# *Exo*‐Selective Intramolecular (4+3) Cycloadditions to *Trans*‐Fused Perhydroazulenes: An Asymmetric Formal Synthesis of (−)‐Pseudolaric Acid B

**DOI:** 10.1002/anie.202509650

**Published:** 2025-08-05

**Authors:** Zengsheng Yin, Yuxuan He, Guo Wei, Yuchen Zhou, Antonio Rizzo, Yun He, Elizabeth H. Krenske, Pauline Chiu

**Affiliations:** ^1^ Department of Chemistry and State Key Laboratory of Synthetic Chemistry The University of Hong Kong Pokfulam Road Hong Kong P.R. China; ^2^ Laboratory for Synthetic Chemistry and Chemical Biology Limited Hong Kong Science Park Shatin Hong Kong P.R. China; ^3^ School of Chemistry and Molecular Biosciences The University of Queensland St Lucia QLD 4072 Australia

**Keywords:** (4+3) cycloaddition, Natural products, One‐pot reactions, Pseudolaric acids, Total synthesis

## Abstract

We present an asymmetric formal total synthesis of pseudolaric acid B (PAB), based on the intramolecular (4+3) cycloaddition of a new class of epoxy enolsilane substrates. This substrate type has the epoxide geminally‐substituted on the tether to the furan, which reacts to yield the *trans*‐fused perhydroazulene core of PAB containing the quaternary stereocenter. The reaction is highly diastereoselective, as opposed to cycloadditions of previous substrate types that generate the perhydroazulene framework. The role of the geminal tether in controlling the transition state conformation and the *exo*‐selectivity is revealed by density functional theory calculations. Using this intramolecular cycloaddition as the key reaction, together with several one‐pot sequences, a key intermediate in Trost's synthesis of PAB was accomplished, representing the shortest asymmetric synthesis of this anti‐tumor natural product thus far.

## Graphical abstract

We present an asymmetric formal synthesis of pseudolaric acid B, for which we developed a new family of substrates with an epoxide geminally tethered to a sidechain bearing a furan, which undergoes a TESOTf‐catalyzed intramolecular (4+3) cycloaddition to yield the *trans*‐fused perhydroazulene core and the requisite quaternary stereocenter of the natural product. The cycloaddition shows exquisite *exo* diastereoselectivity, as opposed to previous related cycloadditions of substrates with vicinally tethered epoxides.

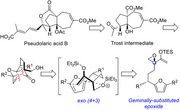

## Introduction

**Figure 1:**
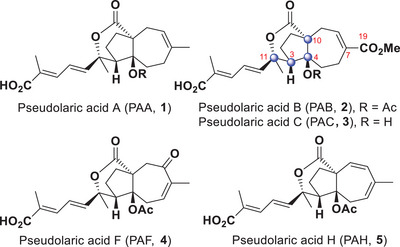
Representative structures of the pseudolaric acid family, highlighting the four contiguous stereocenters of PAB.

Pseudolaric acid B (PAB) is a diterpenoid isolated from the root bark of *Pseudolarix kaempferi*, also known as “*tujingpi*”, which has been used in traditional Chinese medicine to treat eczema and fungal skin infections since the 17^th^ century (Figure 1).^[^ ^1^, ^2^, ^3^, ^4^, ^5^, ^6^ ^]^ To date, more than 20 members of the pseudolaric acid family which share the unusual *trans‐fused* perhydroazulene core structure have been isolated. Among these, pseudolaric acid B (**2**) is the most potent bioactive congener, showing antifungal, antimicrobial, antifertility, anti‐angiogenic, and anticancer activity.^[^ ^7^, ^8^, ^9^, ^10^, ^11^, ^12^, ^13^ ^]^ The bioactive properties of **2** may be further optimized for the treatment of immune disorders, cancer, and other related diseases.^[^ ^14^, ^15^, ^16^, ^17^, ^18^, ^19^, ^20^, ^21^, ^22^, ^23^ ^]^

Structurally, aside from the *trans‐*fused perhydroazulene framework, the presence of four contiguous stereocenters which are either tertiary or quaternary, poses a challenging problem for the synthesis of pseudolaric acid B. The synthetic community has explored various avenues to tackle the synthesis of the pseudolaric acids.^[^ ^24^, ^25^, ^26^, ^27^, ^28^, ^29^, ^30^, ^31^ ^]^ However, since the first asymmetric total synthesis of pseudolaric acid A (PAA, **1**) by Chiu and coworkers in 2006,^[^ ^32^ ^]^ only one asymmetric synthesis of PAB has been accomplished by the Trost group (28 steps).^[^ ^33^, ^34^ ^]^ The groups of Yang (16 steps),^[^ ^35^ ^]^ and then Mori (23 steps)^[^ ^36^ ^]^ subsequently completed racemic total/formal total syntheses of PAA and PAB, respectively.

**Scheme 1:**
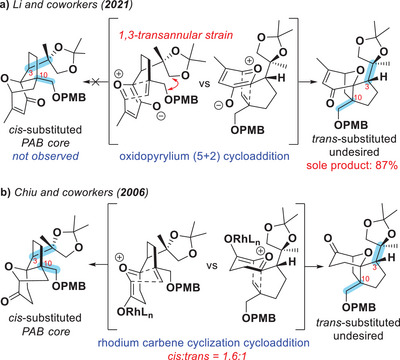
a) Li and coworkers reported an oxidopyrylium (5+2) cycloaddition strategy to the PAB core, but obtained only the undesired diastereomeric cycloadduct.^[^ ^30^ ^]^ b) In the synthesis of PAA by Chiu and coworkers, the cycloaddition was not highly selective for the desired diastereomeric product even after extensive catalyst optimizations.^[^ ^32^ ^].^

Other efforts to solve this synthetic problem were arrested at various stages.^[^ ^4^ ^]^ Recently, Li and coworkers^[^ ^30^ ^]^ reported an approach to PAB construction by applying an intramolecular cycloaddition, merging Wender‐type oxidopyrylium^[^ ^37^, ^38^, ^39^ ^]^ (5+2) cycloaddition chemistry and our approach to PAA^[^ ^32^ ^]^ (Scheme 1a). However, the undesired diastereomeric perhydroazulene was observed as the sole cycloadduct, likely because of unfavorable transannular interactions in the densely packed cycloaddition transition state. This is not entirely surprising, as in our own PAA synthesis, the selectivity of the key step under achiral rhodium catalysis was initially 1:3 in favor of the undesired diastereomeric cycloadduct, and only after a thorough optimization campaign could we increase it to 1.6:1 for the desired diastereomer (Scheme 1b). To expand the structure–activity relationship (SAR) profile towards its therapeutic development, a more efficient synthetic route to provide PAB and its derivatives is still highly desirable.

As (4+3) cycloadditions are a direct and convergent strategy to construct functionally‐endowed cycloheptanoids, they have been widely applied in the syntheses of complex polycyclic natural products.^[^ ^40^, ^41^, ^42^ ^]^ Our group has been exploring the chemistry of epoxy enolsilanes, which engage in successful intramolecular cycloadditions with dienes such as furans, pyrroles, arenes, and thiophenes.^[^ ^43^, ^44^, ^45^, ^46^, ^47^, ^48^, ^49^, ^50^, ^51^, ^52^, ^53^ ^]^

**Scheme 2:**
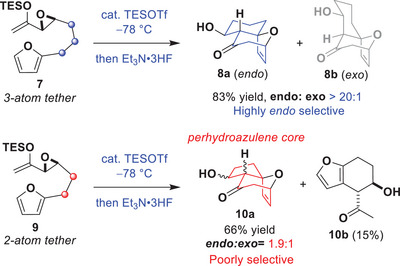
Previous reports of intramolecular (4+3) cycloadditions of vicinally‐tethered epoxy enolsilanes: **7** reacted to provide *endo* product **8a** with excellent yield and dr; while **9** reacted to generate perhydroazulene **10a** with low dr, along with a relevant yield of Friedel–Crafts product **10b**.

The first intramolecular (4+3) cycloadditions of epoxy enolsilanes with furans were reported by our group in 2009.^[^ ^43^ ^]^ We found that cycloaddition substrates such as **7**, bearing a 3‐atom tether between the epoxide and the furan, reacted under silylium catalysis to provide *endo*‐**8a** having a fused [6,7]‐carbobicyclic skeleton in a good yield (83%) and with>20:1 *endo* diastereoselectivity (Scheme 2). However, the intramolecular (4+3) cycloaddition of epoxy enolsilane **9** with a shorter, 2‐atom tether, delivered cycloadduct **10a** having the fused [5,7]‐carbobicyclic framework as a mixture of diastereomers in a reduced 66% yield, along with a substantial amount of Friedel–Crafts product (**10b**,15%).^[^ ^54^ ^]^ Clearly, a simple adjustment of the chain length of these cycloaddition substrates is not a feasible approach to assemble perhydroazulenes, due to the complications from diastereocontrol and side reactions.

**Scheme 3:**
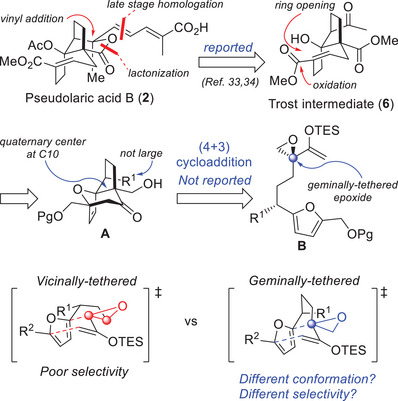
Retrosynthetic analysis of PAB to a new geminally‐tethered epoxide substrate **B** for the intramolecular (4+3) cycloaddition.^[^ ^33^, ^34^ ^].^

Retrosynthetic analysis of PAB led us to first disconnect the diene to provide a methyl ketone; the concomitant deconvolution of the lactone suggests ketoester **6**—which is an intermediate in the Trost synthesis of PAB,^[^ ^33^, ^34^ ^]^ (Scheme 3)—as a formal total synthesis target. Compound **6** can be obtained by functional group transformations, including oxygen‐bridge opening, of oxapolycycle **A**. While the extent of the negative impact of a similar 1,3‐transannular interaction in this context is uncertain, selecting a small R^1^ substituent destined to become the methyl ketone, would be judicious.^[^ ^30^, ^32^ ^]^ In turn, **A** would arise from the intramolecular (4+3) cycloaddition of a furan‐tethered epoxy enolsilane **B**, which could concomitantly generate the requisite quaternary stereocenter at C10.

All the epoxy enolsilanes explored in our cycloaddition studies thus far, including substrates **7** and **9**, have been designed with both carbon atoms of the oxirane as part of the tether to the furan, (ie. vicinally‐substituted on the tether, or vicinally‐tethered with respect to the oxirane, Scheme 3). However, the reactions of epoxy enolsilanes such as **B**, whose epoxide has only one of the carbons as part of the tether (ie. geminally‐tethered with respect to the oxirane, Scheme 3), have not been explored. This represents a new class of epoxy enolsilane substrate for intramolecular (4+3) cycloaddition reactions.

Herein, we report a concise formal synthesis of PAB, which features as the key step a highly diastereoselective intramolecular (4+3) cycloaddition of an epoxy enolsilane whose epoxide is geminally‐tethered. This permits the assembly of the perhydroazulene structure early in the synthetic sequence and thus offers an approach for the structural modifications of the PAB core to fuel SAR studies.

### Intramolecular (4+3) Cycloaddition of Geminally‐Tethered Epoxy Enolsilanes: Model Studies for the Synthesis of PAB

**Scheme 4:**
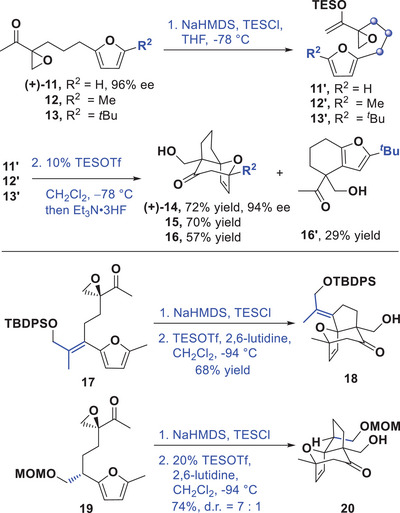
Intramolecular (4+3) cycloaddition reactions of epoxy enolsilanes geminally‐tethered to furans.

To investigate this intramolecular (4+3) cycloaddition more generally, we first designed and synthesized a series of cycloaddition substrates analogous to **B** (Schemes 3, 4). The synthesis of epoxy methyl ketones **11**–**13** was achieved starting from commercially available furan, 2‐methylfuran, and 2‐*tert*‐butylfuran respectively (See Supporting Information). In the event, ketone **11** was converted to its corresponding enolsilane **11**′ by treatment with NaHMDS/TESCl and isolated.^[^ ^44^ ^]^ However, enolsilane **11**′ was observed to be somewhat unstable during chromatographical purification on silica gel. Thus, the crude enolsilane product, without purification, was subjected to the (4+3) cycloaddition conditions directly. Gratifyingly, cycloadduct **14**, was isolated in good yield and as a single *exo*‐diastereomer, having the fused [5,7] bicyclic framework and the correct relative stereochemistry between the oxygen bridge and the quaternary center for pseudolaric acid. Moreover, (+)‐**14** was obtained with a high retention of ee starting from enantiomerically‐enriched (+)‐**11** (72% yield over 2 steps from (+)‐**11**). The relative stereochemistry of **14** was determined by NOE NMR analysis (see Supporting Information). For methyl‐substituted furan **12**, reaction under the standard conditions generated cycloadduct **15** also diastereoselectively, and in a good yield. Notably, even **13** bearing an extremely bulky R^2^ substituent underwent cycloaddition to generate **16** in a moderate yield, accompanied by a Friedel–Crafts side product **16′**.

**Figure 2:**
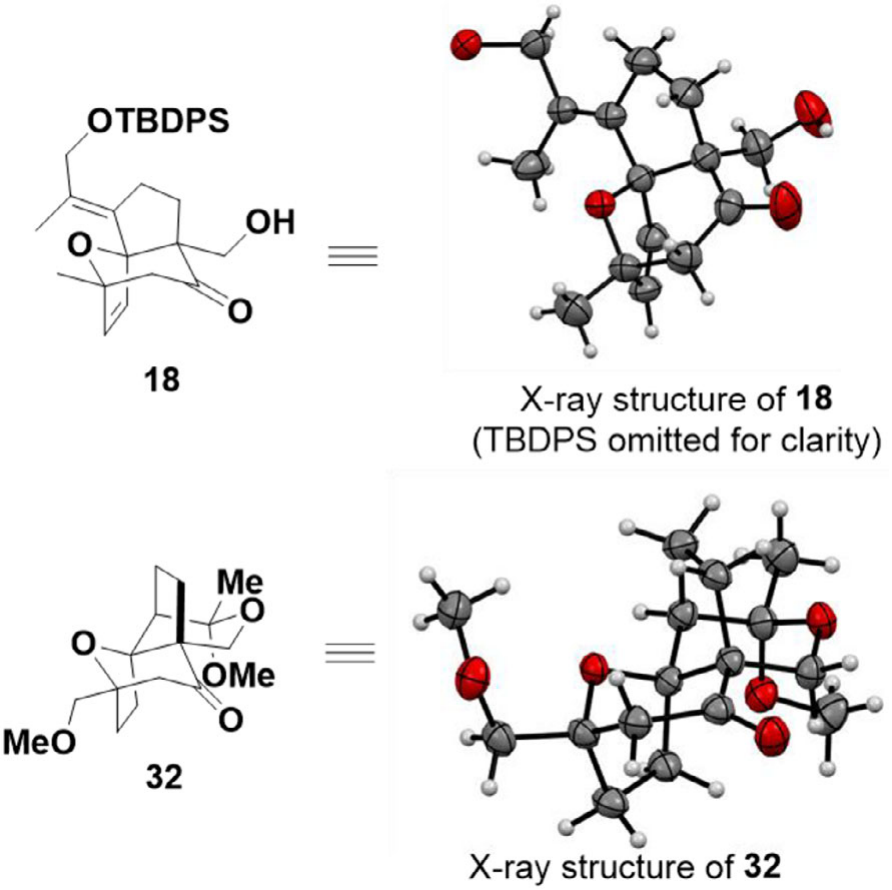
X‐ray crystallographic analyses of compounds **18** and **32**.

After having validated the diastereoselective intramolecular (4+3) cycloaddition of these geminally‐tethered epoxy enolsilanes, we turned our attention to study this reaction for application to the synthesis of the PAB core. To investigate the effect of a substituent R^1^ (Scheme 3), which is a necessary handle to furnish C3 of PAB, we designed and synthesized compound **17** (Scheme 4, see Supporting Information for synthesis). The alkylidene R^1^ minimizes the negative 1,3‐transannular interaction with the epoxide, but also has the potential to be transformed to the required C3 substituent after the (4+3) cycloaddition. Ketone **17** is converted to its enolsilane **17′**, then subjected to catalysis with TESOTf to afford cycloadduct **18** in ∼30% yield. The addition of 2,6‐lutidine as additive and reacting at a lower temperature (−94 °C) improved the yield slightly to 40%. Further optimizations of the loading of TESOTf found that a stoichiometric amount substantially improved the yield to 68% diastereoselectively. The absolute configuration of **18** was determined by X‐ray diffraction analysis (Figure 2).^[^ ^121^ ^]^

We also designed and synthesized compound **19** (see Supporting Information), having an alkoxymethylene substituent as R^1^ with the correct stereochemistry as the C3 side chain in PAB. Treatment of ketone **19** with NaHMDS and TESCl afforded the corresponding enolsilane, and the isolated crude product was subjected to the TESOTf‐promoted (4+3) cycloaddition reaction conditions (Scheme 4), to provide cycloadduct **20** in 74% yield and with a diminished, but still good dr of 7:1. These results suggested that the cycloadditions of this new class of epoxy enolsilane substrates could tolerate small substituents on the tether, and that it was possible to incorporate a stereochemically‐defined substituent present in pseudolaric acid B at an early stage of the synthesis.

**Scheme 5:**
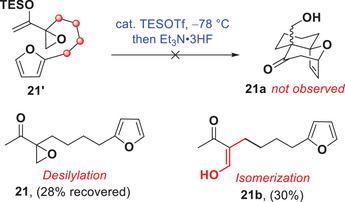
Attempted intramolecular (4+3) cycloaddition reaction of epoxy enolsilane **21′**.

To complete our study, we also examined the cycloaddition of geminally‐substituted epoxy enolsilanes that would provide fused [6,7]‐bicyclic platforms. We prepared and characterized enolsilane **21′** from the corresponding ketone **21**, which bears one additional carbon on the tether between the epoxide and the furan compared to **11** (Scheme 5). Surprisingly, the cycloaddition of **21′** failed to produce **21a** under the same reaction conditions. Instead, parent ketone **21** was recovered after the reaction, along with substantial amounts of isomerized product **21b** arising from epoxide cleavage (see Supporting Information).

### Mechanistic Explanation for the Observed Reactivity and Selectivity

**Figure 3:**
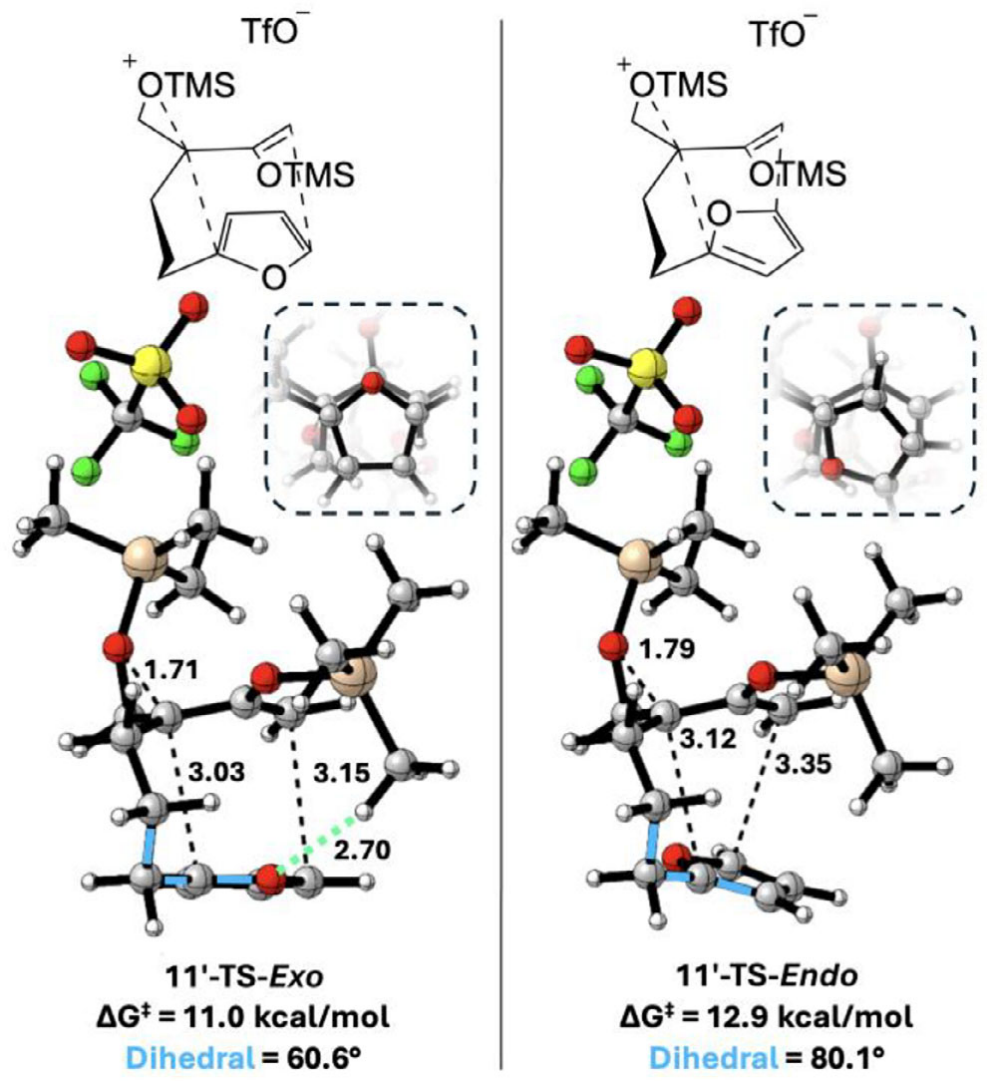
Transition states for the *exo* and *endo* cycloadditions of geminally‐tethered epoxy enolsilane **11′** in CH_2_Cl_2_, calculated at M06‐2X/def2‐TZVPP/CH_2_Cl_2_(SMD)//B3LYP‐D3(BJ)/6–31G(d,p)/CH_2_Cl_2_(CPCM) level of theory. The TES groups have been modelled as TMS groups. Distances in Å. The insets at the top right of each structure show a bottom‐up view of the two reacting moieties.^[^ ^56^, ^57^, ^58^, ^59^, ^60^, ^61^, ^62^, ^63^, ^64^, ^65^ ^].^

We conducted density functional theory calculations to determine the origin of the excellent diastereoselectivity observed in the intramolecular (4+3) cycloaddition reaction of **11**. Previous experimental results and computational calculations^[^ ^55^ ^]^ suggest that (4+3) cycloadditions of vicinally‐tethered epoxy enolsilanes generally follow a mechanism that combines an S_N_2‐like but highly asynchronous backside attack by the diene on the silylium‐activated epoxide and the formation of the second C─C bond between the remote termini of the diene and allyl group, resulting in *endo* cycloadducts. Calculations on the geminally‐tethered substrate **11′** (modelling the TES groups as TMS groups) are shown in Figure 3. Consistent with the retention of optical purity in generating **14,** an *exo* transition state (TS) featuring S_N_2‐like backside attack is found; moreover, this TS is favored by 1.9 kcal mol^−1^ (ΔΔ*G* ^‡^), corresponding to>100:1 selectivity at −78°C, aligning well with the experimental dr. In the *exo* TS, the furan aligns almost perfectly with the reacting moiety of the epoxy enolsilane (as illustrated by the bottom‐up view in the inset of Figure 3). There is a weak stabilizing CH···O interaction between one of the SiMe groups and the furan. In the *endo* TS, in contrast, the SiMe group is close to a furan CH group. To minimize the H···H clash, the reacting moieties move away from each other and the furan tilts by about 20°, weakening the bond‐forming interactions and increasing torsional strain.

Interestingly, these transition states display a significant conformational difference compared to those calculated previously for vicinally‐tethered epoxy enolsilanes. For **11′**, the epoxide and the enol C═C groups are *s‐cis* to each other, whereas in other reactions of this class, the two groups are *s‐trans*. For substrates with the geminally‐tethered epoxide as **11′**, the *s‐cis* arrangement allows better alignment of the bond‐forming centers and permits the tether to adopt its preferred half‐chair conformation. Further details of the TS conformers and an analysis of the interactions within them are provided in the Supporting Information.

Computations also reveal that tether constraint effects are responsible for the failure of the cycloaddition of substrate **21′** which has a 4‐carbon tether between the epoxide and the furan. The longer tether cannot easily position the reacting moieties in an ideal alignment for reaction, leading to a much higher reaction barrier (see the Supporting Information for details).

### Formal Total Synthesis of PAB

**Scheme 6:**
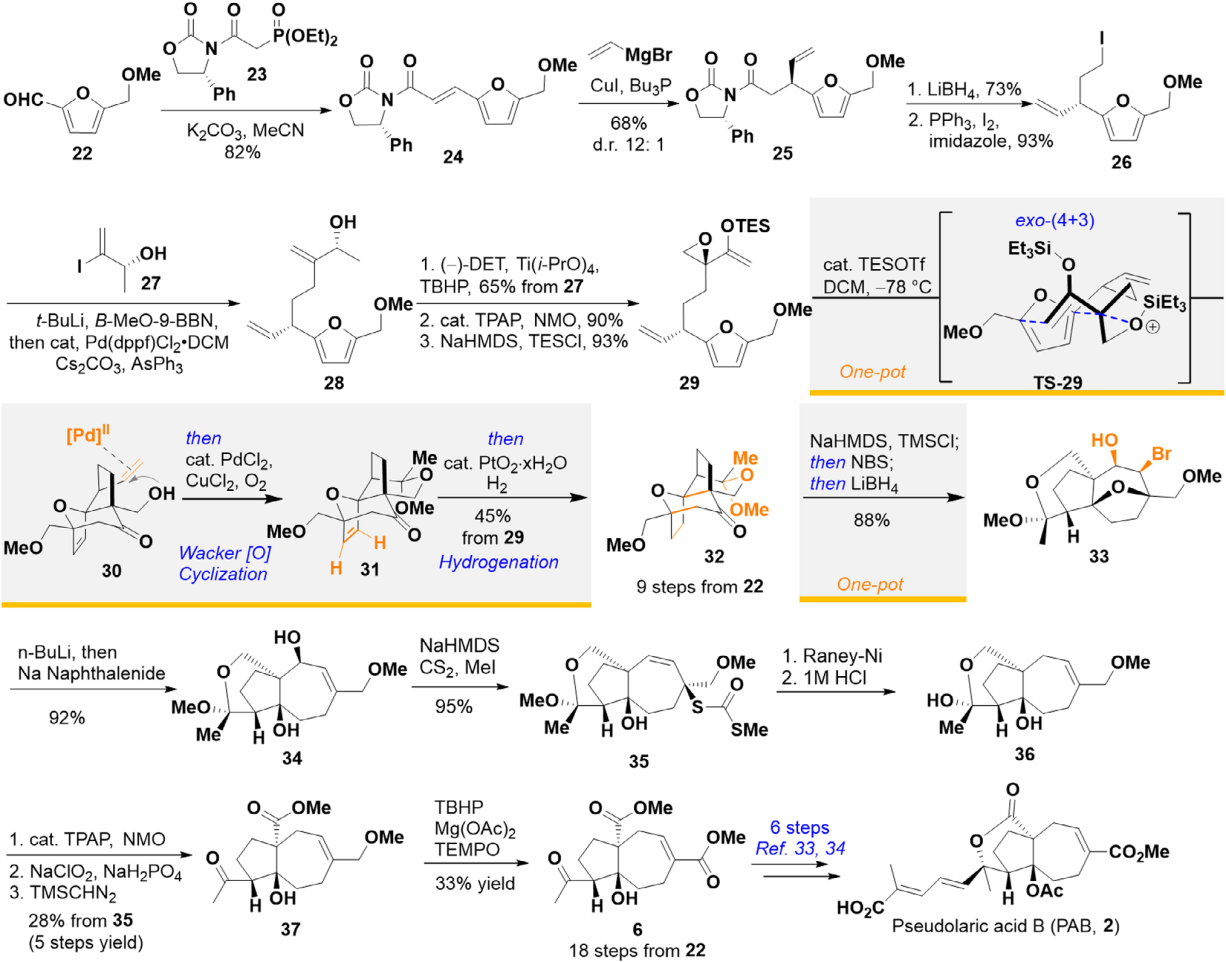
Formal total synthesis of PAB. D‐DET=D‐diethyl tartrate; TBHP=*tert*‐Butyl hydroperoxide; TPAP=Tetrapropylammonium perruthenate; NMO=*N*‐Methylmorpholine *N*‐oxide.

According to our retrosynthetic analysis (Scheme 3) and the model studies (Scheme 4), the key intramolecular (4+3) cycloaddition reaction can accommodate a small R^1^ without substantial loss in diastereoselectivity. Therefore, a vinyl group was selected to be a surrogate of the acetyl moiety, which would be installed as the C3 substituent on the cycloaddition precursor (Scheme 6).

Our formal synthesis of PAB started with commercially available aldehyde **22**. Phosphonate **23** ^[^ ^66^ ^]^ was deprotonated with NaHMDS or NaH, and then treated with aldehyde **22**. This Horner–Wadsworth–Emmons reaction was not only a means to homologate **22**, but also to introduce the Evans auxiliary to enamide **24**.^[^ ^66^, ^67^ ^]^ However, the requirements of anhydrous solvents and an inert atmosphere are unfriendly for operations on a large scale as is the case for the first step in this synthetic route. Upon further exploration, we found that K_2_CO_3_ can promote this reaction with acetonitrile as solvent in an open flask, to afford **24** in 82% yield routinely on>20‐gram scales.

Enamide **24** sets us up to introduce the requisite vinyl group diastereoselectively at the β‐position. The use of vinyl Grignard reagents alone resulted mainly in 1,2‐additions,^[^ ^68^, ^69^ ^]^ but in the presence of CuBr•DMS, conjugate addition occurred to generate the desired product **25** in 72% yield.^[^ ^70^ ^]^ However, when this reaction was conducted on a 10‐gram scale, precipitates formed in the low‐temperature reaction posed significant challenges for effective stirring and further scaling up. Thus, we replaced CuBr•DMS with a more soluble mixture of CuI and Bu_3_P.^[^ ^71^ ^]^ In the event, the reaction remained homogeneous even on a 30‐gram scale, and the desired vinylated product **25** was obtained in 68% yield and with a dr of 12:1. Reductive removal of the chiral auxiliary,^[^ ^72^ ^]^ followed by an Appel reaction^[^ ^73^ ^]^ furnished iodide **26**.

We proceeded to induce a one‐pot sp^2^‐sp^3^ cross‐coupling of **26** and **27**. Compound **26** was converted to an alkylborane via a lithium‐iodide exchange and quenching with *B*‐MeO‐9‐BBN. To this was added Pd catalyst and vinyl iodide **27**.^[^ ^74^ ^]^ This *B*‐alkyl Suzuki reaction^[^ ^75^ ^]^ furnished allylic alcohol **28**, which was subjected to a Sharpless asymmetric epoxidation,^[^ ^76^ ^]^ to afford optically‐enriched epoxy alcohol in 65% yield over two steps. The epoxy alcohol was oxidized^[^ ^77^, ^78^ ^]^ to the corresponding methyl ketone (90% yield), which was readily transformed to epoxy enolsilane **29** using NaHMDS/TESCl (93% yield).

With the precursor of the key cycloaddition step in hand, we first treated **29** under the optimized reaction conditions (stoichiometric TESOTf and 2,6‐lutidine at −94 °C) found for obtaining cycloadduct **18**. The reaction proceeded, but cycloadduct **30** was obtained in low yield. Further optimizations of solvent, temperature, time, TESOTf:2,6‐lutidine ratio and stoichiometry (See Supporting Information) finally found that 20% TESOTf in DCM at −78 °C provided reproducible yields of **30** (48% yield, dr = 7:1). We surmise that **29** bearing a methoxymethylenated furan, was less stable than enolsilane **17′** or **19′** under the Lewis acidic conditions.

Cycloadduct **30** was then subjected to a Wacker oxidation.^[^ ^79^ ^]^ Instead of the expected methyl ketone, ketal **31** was obtained in 75% yield as one diastereomer. Fortuitously, the in situ reaction of the carbonyl group obviated additional steps to protect this functional group. Hydrogenation of **31** under catalytic Pd/C and H_2_ (1 atm) generated the desired product **32** in 93% yield. To maximize pot‐economy,^[^ ^80^ ^]^ we also developed a protocol that circumvented the purification of intermediates **30** and **31**. In the event, quenching the TESOTf‐induced (4+3) cycloaddition with anhydrous MeOH, and then subjecting the resulting mixture to Wacker oxidation directly furnished **31**. When we tried to incorporate the hydrogenation step into the one‐pot protocol, we found that employing Pd/C as catalyst necessitated, after the Wacker oxidation, filtration through celite in order for the hydrogenation to proceed efficiently. However, when PtO_2_•xH_2_O was employed instead of Pd/C, hydrogenation proceeded simply by adding the catalyst to the reaction mixture. In this manner, ketal **32** was isolated as a single diastereomer in 45% yield over three reactions in one pot from **29**. The absolute stereochemistry of **32** was confirmed by X‐ray diffraction analysis (Figure 2).^[^ ^121^ ^]^ The success of this one‐pot protocol demonstrated that the individual reactions must have been rather clean; and not only resulted in economies in terms of time and chemicals but also provided **32** in a higher overall yield than the sequence of individual reactions with workup and purifications. These transformations generated three new rings stereoselectively to increase the complexity of a sp^2^‐carbon‐rich substrate to a completely sp^3^‐carbon framework. Moreover, this core has been achieved in nine steps from aldehyde **22** and is amenable to be derivatized for SAR studies, as the core carbon framework of the pseudolaric acids is already formed.

To complete the formal synthesis of Trost's intermediate (**6**), cleavage of the oxygen bridge in **32** was accomplished by a reductive elimination strategy.^[^ ^81^ ^]^ α‐Bromination of ketone **32** was achieved by silyl enol ether formation followed by treatment with NBS. Bromination was diastereoselective from halogenation occurring *syn* with respect to the oxygen bridge. Reduction with LiBH_4_ provided alcohol **33**, also diastereoselectively, from hydride delivery from the less hindered face with respect to the α‐bromo‐substituent. These three transformations were also realized in one pot by the sequential addition of reagents to **32**, and alcohol **33** was isolated in 88% yield as a single diastereomer. Deprotonation of **33** with *n*‐BuLi, and treatment of the resulting lithium alkoxide with sodium naphthalenide furnished ring‐opened diol **34** in 92% yield.

To achieve the deoxygenation of **34**, our first approach was to activate the hydroxyl group, then reduction. However, tosylation or converting the −OH group to a halide leaving group promptly led to transannular *O*‐cyclization via S_N_2' displacement from attack by the C4 tertiary hydroxyl group. We then explored a Barton–McCombie reaction. Treatment with CS_2_ to generate the xanthate ester proceeded to undergo an unexpected but known [3,3]‐sigmatropic rearrangement^[^ ^82^, ^83^ ^]^ to generate the isomeric carbonodithionate **35** (95% yield). Fortunately, Raney‐Ni cleaved the C─S bond with concomitant isomerization to the desired trisubstituted cycloheptene. The crude material was subjected to an acidic hydrolysis to yield hemiketal **36** as a mixture of isomers which was further oxidized, firstly to the ketoaldehyde, then under Pinnick conditions to give the corresponding carboxylic acid.^[^ ^84^ ^]^ Methylation with TMSCHN_2_^[^ ^85^ ^]^ furnished methyl ester **37** in 28% yield over five steps from **35** (ca. 80% yield per step on average). Indeed, each reaction in this sequence provided crude products that successfully proceed to subsequent reactions without protracted and time‐consuming chromatographic purifications.

Conventionally, conversion of a methyl ether to methyl ester would follow a sequence of demethylation, oxidation to the acid, then methyl ester formation. Early on in our synthetic plans, we incorporated the design of a late‐stage selective oxidation of the ether at the allylic position to provide the unsaturated ester efficiently. Indeed, treatment of **37** with a combination of Mg(OAc)_2_·4H_2_O‐TBHP‐TEMPO^[^ ^86^ ^]^ provided the key intermediate **6** in Trost's total synthesis of pseudolaric acid B in 33% yield (unoptimized). The characterization data of **6** we synthesized are consistent with those reported in the literature.^[^ ^33^, ^34^ ^]^

## Conclusion

In summary, we accomplished a formal asymmetric synthesis of pseudolaric acid B by assembling the advanced intermediate **6** in Trost's total synthesis of PAB in 18 steps, which represents the most concise synthesis of PAB thus far. To construct its *trans*‐fused perhydroazulene scaffold, we designed a new family of substrates for intramolecular (4+3) cycloaddition reactions: epoxy enolsilanes geminally tethered to furans. These cycloaddition reactions have exquisite *exo* selectivity, in contrast to the previous analogous but vicinally‐tethered substrates. The selectivity is a result of tether‐induced conformational preferences and alignment in the transition state. This method represents an efficient solution for the synthesis of *trans*‐fused perhydroazulene frameworks with the concomitant establishment of a quaternary stereocenter. Moreover, we integrated the intramolecular (4+3) cycloaddition with a Wacker oxidation and a hydrogenation in a one‐pot protocol, delivering in high efficiency a polycyclic compound which features the core of the pseudolaric acids in nine steps. The synthetic route features a reductive cleavage of the oxygen‐bridge and a late‐stage formation of an ester through an allylic oxidation of a methyl ether. This synthesis demonstrates the potential of (4+3) cycloadditions of epoxy enolsilanes and dienes in complex natural product synthesis. Applications of this strategy to the asymmetric synthesis of the pseudolaric acids and modifications of PAB for SAR studies are on‐going in our lab.

## Supporting Information

Supporting information available: Supporting figures and tables, experimental procedures, computational methods, and data. The authors have cited additional references within the Supporting Information.^[^ ^87^, ^88^, ^89^, ^90^, ^91^, ^92^, ^93^, ^94^, ^95^, ^96^, ^97^, ^98^, ^99^, ^100^, ^101^, ^102^, ^103^, ^104^, ^105^, ^106^, ^107^, ^108^, ^109^, ^110^, ^111^, ^112^, ^113^, ^114^, ^115^, ^116^, ^117^, ^118^, ^119^, ^120^ ^]^ For supplementary crystallographic data see reference.^[^ ^121^ ^]^

## Conflict of Interests

The authors declare no conflict of interest.

## Supporting information



Supporting Information

## Data Availability

The data that support the findings of this study are available in the Supporting Information of this article.
