# Supplementary material for: Exo‐Selective Intramolecular (4+3) Cycloadditions to Trans‐Fused Perhydroazulenes: An Asymmetric Formal Synthesis of (−)‐Pseudolaric Acid B
Source: Angew Chem Int Ed Engl. 2025 Aug 5;64(38):e202509650. doi: 10.1002/anie.202509650 (PMC12435416; doi:10.1002/anie.202509650)

Exo-selective Intramolecular (4+3) Cycloadditions to *trans*-Fused Perhydroazulenes: An Asymmetric Formal Synthesis of (–)-Pseudolaric acid B

Zengsheng Yin,[a,b] Yuxuan He,[a] Guo Wei,[a] Yuchen Zhou,[c] Antonio Rizzo,[a] Yun He, [a] Elizabeth H. Krenske,*[c] and Pauline Chiu*,[a,b]

[a] Dr. Z. Yin, Y. He, Dr. G. Wei, Dr. A. Rizzo, Dr. Y. He, Prof. Dr. P. Chiu*
Department of Chemistry, and State Key Laboratory of Synthetic Chemistry
The University of Hong Kong
Pokfulam Road, Hong Kong
E-mail: pchiu@hku.hk

[b] Dr. Z. Yin, Prof. Dr. P. Chiu
Laboratory for Synthetic Chemistry and Chemical Biology Limited
Hong Kong Science Park, Shatin, Hong Kong

[c] Y. Zhou, Prof. Dr. E. H. Krenske
School of Chemistry and Molecular Biosciences
The University of Queensland
St Lucia, QLD 4072, Australia
E-mail: e.krenske@uq.edu.au

Table of Contents

1. Supporting Schemes and Tables S1

1.1. Scheme S1. Synthetic route for (4+3) cycloaddition substrates-1 ..... S1

1.2. Scheme S2. Synthetic route for (4+3) cycloaddition substrate-2. ..... S2

1.3. Scheme S3. Synthetic route for (4+3) cycloaddition substrate-3. ..... S3

1.4. Scheme S4. Synthetic route for (4+3) cycloaddition substrate-4. ..... S4

1.5. Table S1. Optimization of (4+3) cycloaddition of enolsilane 17′. S4

1.6. Table S2. Optimization of (4+3) cycloaddition of enolsilane 19′. S5

1.7. Table S3. Optimization of (4+3) cycloaddition of enolsilane 29. ..... S5

2. General information S7

3. Synthetic Protocols S8

3.1. Preparation of substrates for intramolecular (4+3) cycloaddition-1 ..... S8

3.2. Preparation of substrates for intramolecular (4+3) cycloaddition-2 ..... S27

3.3. Preparation of substrates for intramolecular (4+3) cycloaddition-3 ..... S34

3.4. Preparation of substrates for intramolecular (4+3) cycloaddition-4 ..... S45

3.5. Formal total synthesis of pseudolaric acid B: Synthesis of 6 ..... S51

4. X-Ray Crystallographic Data S70

5. Computational studies. S79

5.1. Computational results S79

5.2 Computational Methods S83

5.3 Molecular Coordinates Summary S84

6 References for the SI S110

7 NMR spectra S112

1. Supporting Schemes and Tables

1.1. Scheme S1. Synthetic route for (4+3) cycloaddition substrates-1

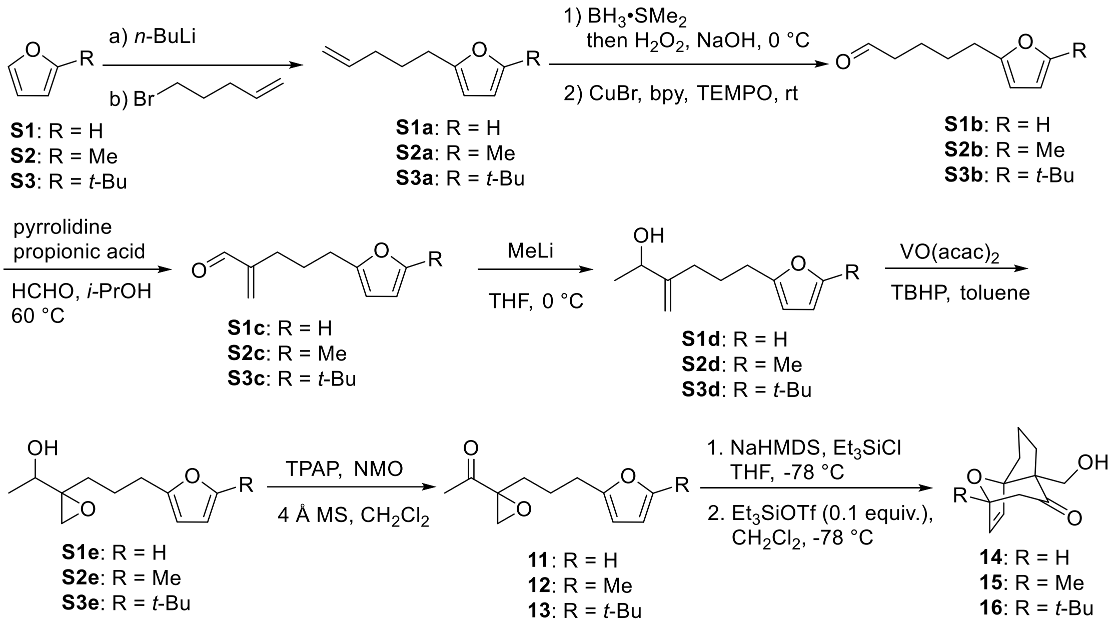

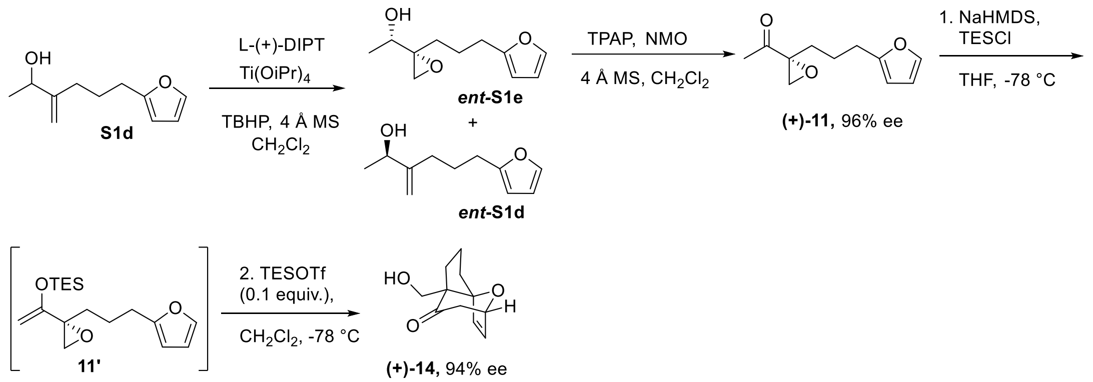

1.2. Scheme S2. Synthetic route for (4+3) cycloaddition substrate-2.

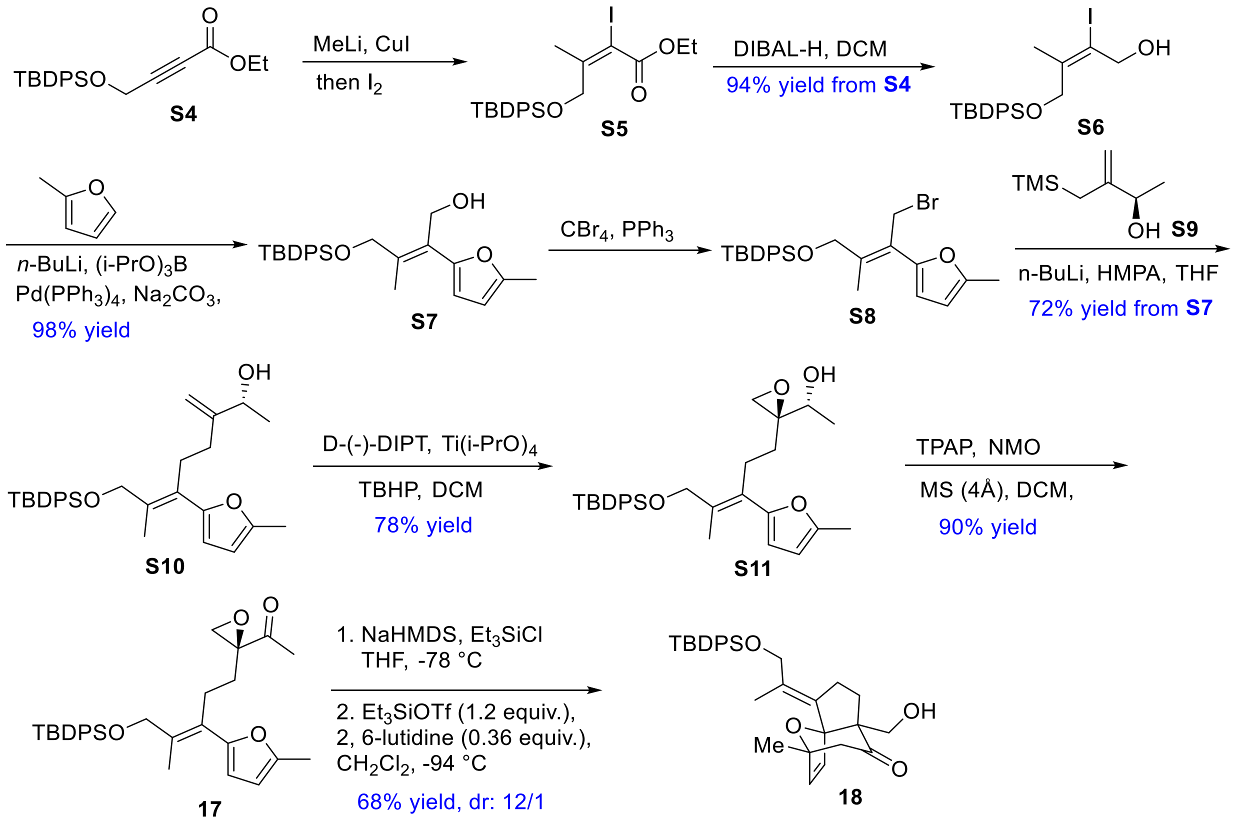

1.3. Scheme S3. Synthetic route for (4+3) cycloaddition substrate-3.

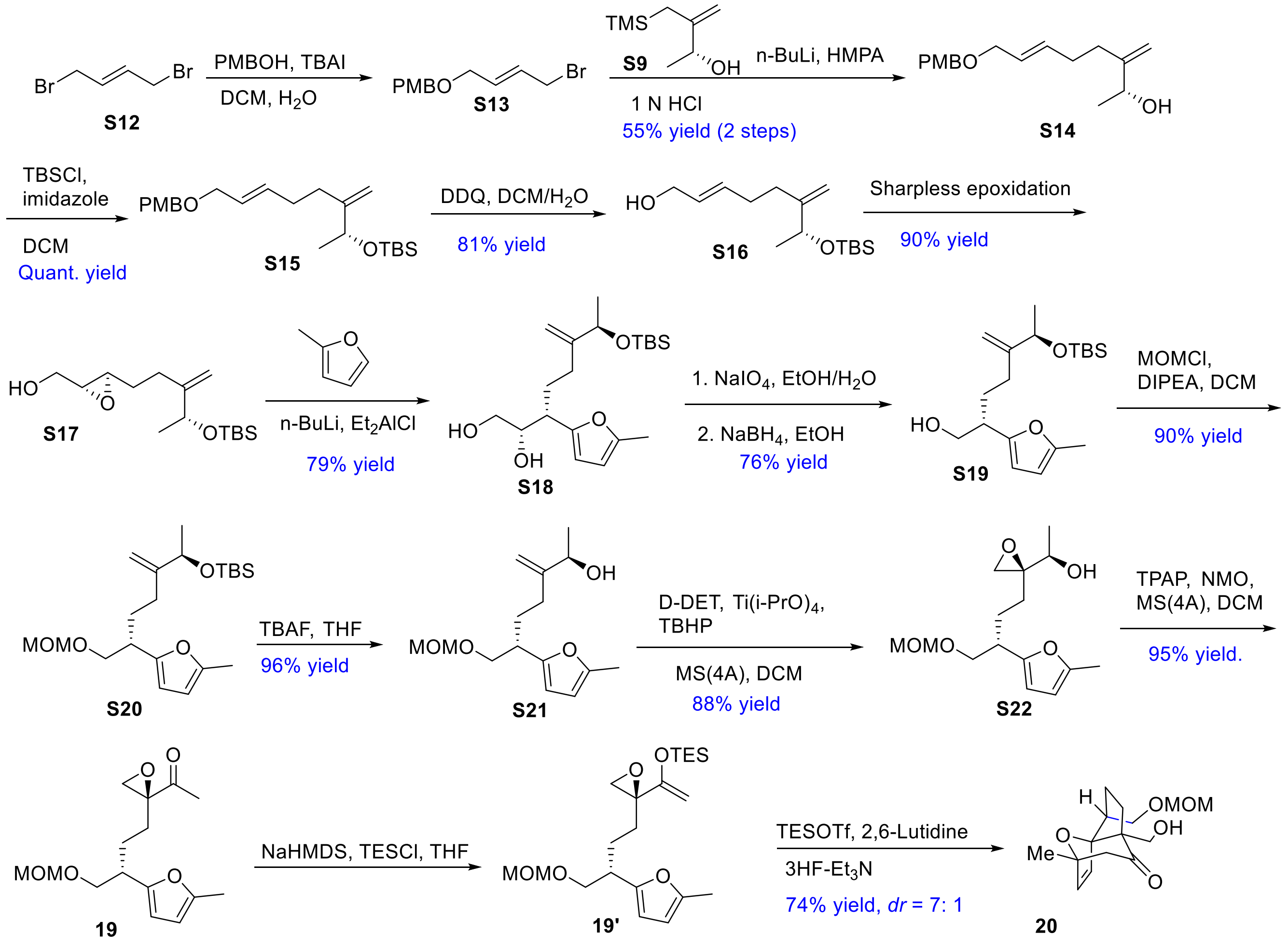

1.4. Scheme S4. Synthetic route for (4+3) cycloaddition substrate-4.

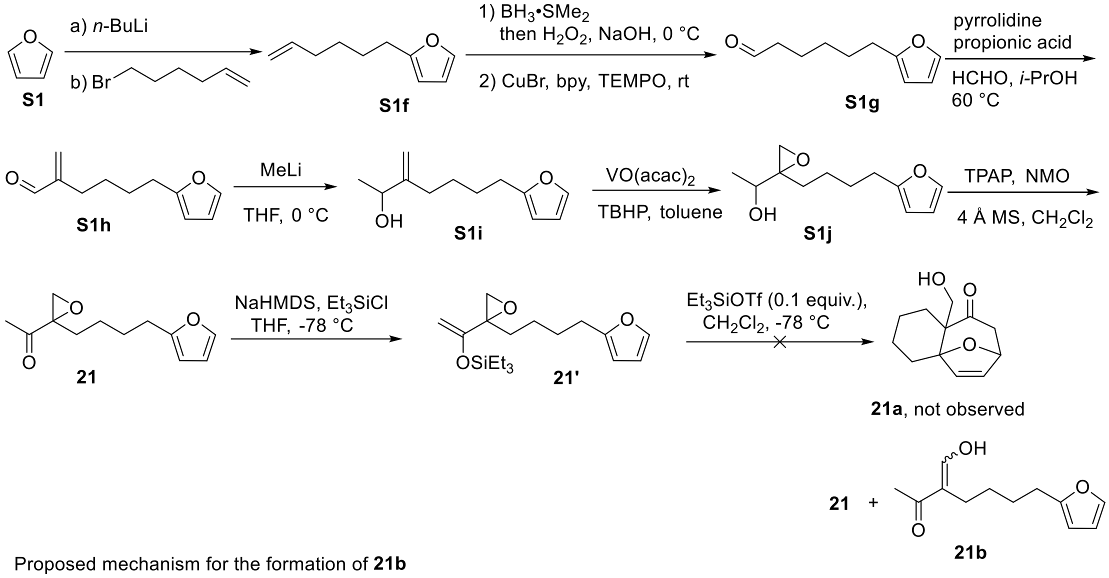

Proposed mechanism for the formation of **21b**

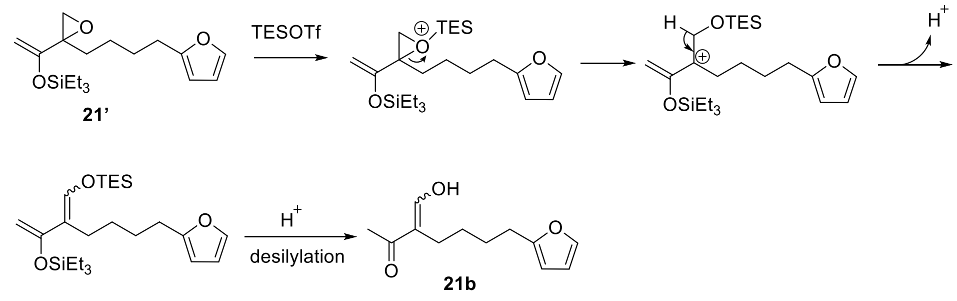

1.5. Table S1. Optimization of (4+3) cycloaddition of enolsilane 17′.

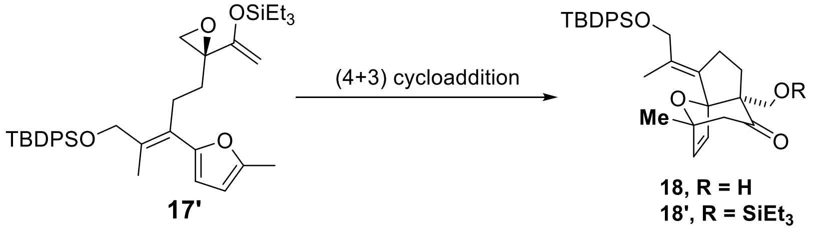

Entry	TESOTf (equiv.)	2,6-lutidine (equiv.)	solvent	T (°C)	Result
1	0.2	----	DCM	–78	**18**, 25%, *d.r.*: 7/1
2	0.5	----	DCM/PhMe	–78	**18**, 33%, *d.r.*: 7/1
3	1.0	----	DCM	–78	**18**, 40%, *d.r.*: 8/1
4	0.5	0.05	DCM	–78	**18**, 36%, *d.r.*: 8/1
5	0.5	0.15	DCM	–78	**18**, 39%, *d.r.*: 8/1
6	1.0	0.1	DCM	–78	**18**, 63%, *d.r.*: 8/1
7	1.0	0.3	DCM	–94	**18,** 60%, *d.r.*: 11/1

8	1.2	0.36	DCM	–78	**18**, 43%, *d.r.*: 8/1
9	1.0	0.3	DCM	–78	**18**, 49%, *d.r.*: 6/1
10	1.0	0.5	DCM	–78	**18**, 52%, *d.r.*: 5/1
11	1.0	0.3	DCM	–78	**18**, 64%, *d.r.*: 7/1
12	1.2	0.36	DCM	–94	**18**, 68%, *d.r.*: 12/1
13	1.5	0.15	DCM	–94	**18**, 46%, *d.r.*: 12/1
14	1.5	0.3	DCM	–94	**18’,** 64%, *d.r.*: 8/1

1.6. Table S2. Optimization of (4+3) cycloaddition of enolsilane 19′.

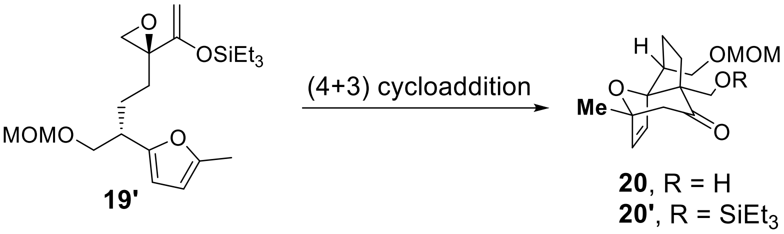

19'

20, R = H
20', R = $SiEt_3$

entry	TESOTf (equiv.)	2,6-lutidine (equiv.)	solvent	T (°C)	Result
1	1.0	0.1	DCM	–94	**20′**, 29%, *d.r.*: 7/1
2	1.2	0.24	DCM	–78	**20**, 39%, *d.r.*: 4/1
3	0.1	----	DCM	–78	**20**, 50%, *d.r.*: 7/1
4	0.2	----	DCM	–78	**20**, 59%, *d.r.*: 8/1
5	0.5	----	DCM	–78	**20**, 52%, *d.r.*: 6/1
6	0.2	0.04	DCM	–78	**20**, 51%, *d.r.*: 6/1
7	0.2	0.02	DCM	–78	**20**, 67%, *d.r.*: 8/1
8	0.2	0.02	DCM	–94	**20**, 74%, *d.r.*: 8/1

1.7. Table S3. Optimization of (4+3) cycloaddition of enolsilane 29.

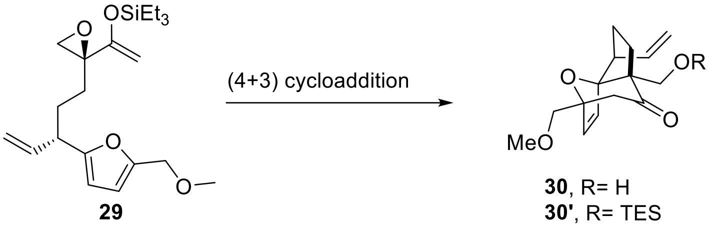

29

30, R= H
30', R= TES

entry	Et_3SiOTf (equiv.)	2,6-lutidine (equiv.)	solvent	T (°C)	Results
1	0.2	----	DCM	–78	**30**, 48%, *d.r.*: 7/1
2	0.1	----	DCM	–94	**30**, 40%, *d.r.*: 10/1
3	0.2	----	DCM	–94	**30**, 42%, *d.r.*: 10/1

4	0.2	0.02	DCM	–94	**30**, 40%, *d.r.*: 9/1
5	0.2	0.02	DCM	–78	**30′**, 48%, *d.r.*: 5/1
6	0.5	0.05	DCM	–78	**30′**, 42%, *d.r.*: 7/1
7	0.2	0.02	DCM	–94	**30′**, 37%, *d.r.*: 6/1
8	1	0.1	DCM	–94	**30′**, 35%, *d.r.*: 5/1
9	1.5	0.15	DCM	–78	**30′**, 50%, *d.r.*: 10/1
10	1.5	0.3	DCM	–78	**30′**, 43%, *d.r.*: 6/1
11	1.2	0.12	DCM	–78	**30′**, 35%, *d.r.*: 5/1
12	1.5	0.15	DCM	–94	**30′**, 40%, *d.r.*: 8/1
13	0.2	----	Tol	–78	**30′**, 40%, *d.r.*: 7/1
14	0.2	0.04	Tol	–78	**30′**, 44%, *d.r.*: 8/1
15	1.1	0.22	Tol	–94	**30′**, 40%, *d.r.*: 6/1

2. General information

All anhydrous reactions were performed in oven-dried round-bottomed flasks under a positive pressure of dry argon. Air and moisture-sensitive compounds were introduced via syringes or cannula using standard inert atmosphere techniques. Reactions were monitored by thin layer chromatography (TLC) using E. Merck silica gel plates, Kieselgel 60 F_{254} with 0.2 mm thickness. Components were visualized by illumination with short-wavelength ultra-violet light and/or staining. Flash column chromatography was performed with E. Merck silica gel 60 (230-400 mesh ASTM).

Solvents and chemicals were purified according to standard procedures. All solvents used for reactions were distilled or dried by passing through drying columns. Tetrahydrofuran (THF), dichloromethane (DCM), toluene, chlorotriethylsilane (TESCl), furan, 1,1,1,3,3,3-hexamethyldisilazane (HMDS), triethylamine (Et_3N) were distilled from CaH_2 under argon. In particular, the solvents used in all (4+3) cycloadditions were distilled from CaH_2 under argon then dried over 4Å MS. Other reagents were used as received.

^1H and ^{13}C NMR nuclear magnetic resonance spectra were recorded in deuteriochloroform ($CDCl_3$), with residual solvent peak as an internal standard, hexadeuterobenzene (C_6D_6), dideuteromethylenechloride (CD_2Cl_2) or toluene-d_8 at ambient temperature (unless otherwise specified) on a Bruker DX 300 spectrometer, Bruker Avance 400 spectrometer, Bruker DX 500 spectrometer, or Bruker Avance 600 operating at 300 MHz, 400 MHz, 500 MHz or 600 MHz respectively for ^1H, and at 75 MHz, 100 MHz, 125 MHz or 150 MHz respectively for ^{13}C. All spectra were calibrated at δ 7.26 or δ 0.00 ppm for ^1H spectra (residual $CHCl_3$ or TMS respectively), and δ 77.16 ppm for ^{13}C spectra, at δ 7.16 ppm for 1H spectra (residual C_6H_6), and δ 128.06 ppm for ^{13}C spectra. Splitting patterns were designated as follows: s = singlet, d = doublet, t = triplet, q = quartet, m = multiplet, br = broad. Electron impact mass spectrometry was recorded on a Finnigan MAT 95 mass spectrometer or API QSTAR PULSAR iLC/MS/TOF System for both low resolution and high resolution, with accurate mass reported for the molecular ion [M^+] or next largest fragment thereof. High-resolution ESI-MS measurements were performed on a Bruker impact II high-resolution LC-QTOF mass spectrometer. Accurate masses from high-resolution mass spectra were reported for the molecular ion $[M+H]^+$ or $[M+Na]^+$. Optical rotations were recorded as solutions in DCM on a Bellingham + Stanley ADP440+ polarimeter.

3. Synthetic Protocols

3.1. Preparation of substrates for intramolecular (4+3) cycloaddition-1

Synthesis of Substrate 11 from S1

Preparation of S1a:

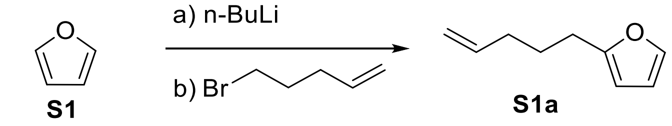

To a stirred solution of furan (29.0 mL, 455 mmol) in anhydrous THF (300 ml), was added *n*-BuLi (2.4 M, 100 mL, 240 mmol) at 0 °C. After stirring at 0 °C for 1 h, 5-bromo-1-pentene (8.95 g, 60.1 mmol) was added dropwise. The reaction mixture was allowed to warm to room temperature and stirred overnight. The reaction was quenched with saturated NH_4Cl (aq.) at 0 °C and the aqueous layer was extracted with Et_2O three times. The combined organic phases were dried over anhydrous MgSO4 and concentrated by rotary evaporation. The crude product **S1a** was used without further purification in the following step.

R_f: 0.62 (hexane);

^{1}H NMR (400 MHz, $CDCl_3$) δ 7.30 (dd, J = 1.9, 0.8 Hz, 1H), 6.28 (dd, J = 3.2, 1.9 Hz, 1H), 5.98 (dd, J = 3.0, 1.0 Hz, 1H), 5.82 (ddt, J = 17.0, 10.2, 6.7 Hz, 1H), 5.11 – 4.93 (m, 2H), 2.64 (t, J = 7.6 Hz, 2H), 2.11 (dtd, J = 7.8, 6.8, 1.4 Hz, 2H), 1.74 (p, J = 7.5 Hz, 2H) ppm; ^{13}C NMR (101 MHz, $CDCl_3$) δ 156.3, 140.9, 138.4, 115.1, 110.2, 104.9, 33.3, 27.5, 27.4 ppm.

The spectral characteristics are consistent with those of **S1a** in the literature. [87]

Preparation of S1b: *[88, 89]*

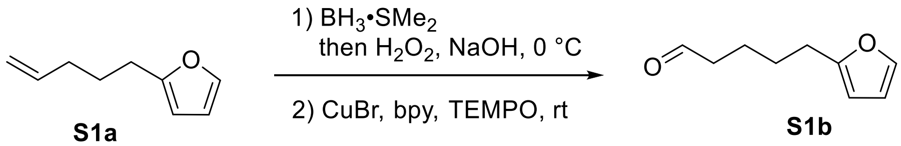

To a stirred solution of crude **S1a** in anhydrous THF (100 mL) was added $BH_3 \cdot SMe_2$ (10 M, 12.2 mL, 122 mmol) at 0 °C. After stirring at 0 °C for 2 h, the reaction was quenched with 5 M NaOH (aq.). H_2O_2 (30% aqueous solution, 40.0 mL, 350 mmol) was added in portions at 0 °C and the reaction was stirred at room temperature overnight. The organic layer was separated, and the aqueous layer was extracted with Et_2O three times. The combined organic phases were dried over anhydrous MgSO4 and concentrated by rotary evaporation. The residue was used in the next step without further purification.

The crude product was dissolved in CH_3CN (100 mL). CuBr (0.86 g, 6.1 mmol), bpy (bpy = 2,2′-bipyridine; 0.94 g, 6.1 mmol), and TEMPO (0.95g, 6.1 mmol) were added sequentially. The reaction was placed under an atmosphere of O_2 using a balloon charged with O_2, and the reaction mixture was stirred at room temperature until a green solution was observed. After this, the mixture was filtered through a pad of silica gel, and the filtrate was concentrated. The residue was purified by flash column chromatography (eluent: 5% EtOAc in hexanes) to afford **S2b** (9.13 g, 68% yield over 3 steps) as a yellow oil.

R_f: 0.52 (10% EtOAc in hexane);

^1H NMR (400 MHz, C_6D_6) δ 9.23 (q, *J* = 1.7 Hz, 1H), 7.12 (d, *J* = 1.8 Hz, 1H), 6.14 – 6.08 (m, 1H), 5.86 – 5.76 (m, 1H), , 2.34 (t, *J* = 7.2 Hz, 2H), 1.78 – 1.65 (m, 2H), , 1.39 – 1.17 (m, 4H) ppm. ^{13}C NMR (101 MHz, C_6D_6) δ 200.4, 156.0, 141.1, 110.5, 105.3, 43.4, 27.9, 27.7, 21.6 ppm.

The spectral characteristics are consistent with those of **S1b** in the literature. [89]

Preparation of S1c: *[90]*

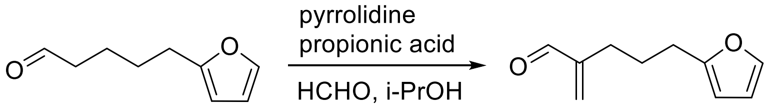

S1b **S1c**

To a stirred solution of **S1b** (3.04 g, 20.0 mmol) in *i*PrOH (10 mL) was added pyrrolidine (0.160 mL, 2.0 mmol), propionic acid (0.160 mL, 2.02 mmol) and formaldehyde (37% aq., 1.60 mL, 20 mmol). The resulting mixture was heated at 60 °C overnight. Et_2O (100 mL) and water (100 mL) were added. The

organic layer was separated, and the aqueous layer was extracted with Et_2O three times. The combined organic phases were dried over anhydrous $MgSO_4$ and concentrated by rotary evaporation. The residue was purified by flash silica gel column chromatography (5% EtOAc in hexanes) to provide aldehyde **S1c** (1.77 g, 54% yield) as a colorless oil. It should be noted that considerable decomposition was observed during silica gel column chromatography.

R_f: 0.57 (10% EtOAc in hexanes).

^{1}H NMR (400 MHz, C_6D_6) *δ* 9.19 (s, 1H), 7.10 (d, *J* = 1.9, 0.8 Hz, 1H), 6.10 (dd, *J* = 3.1, 1.9 Hz, 1H), 5.86 – 5.81 (m, 1H), 5.51 (q, *J* = 1.2 Hz, 1H), 5.21 (t, *J* = 0.9 Hz, 1H), 2.41 – 2.34 (m, 2H), 2.09 (td, *J* = 7.7, 1.3 Hz, 2H), 1.64 – 1.54 (m, 2H) ppm; ^{13}C NMR (100 MHz, C_6D_6) δ 193.0, 155.5, 149.6, 140.7, 132.5, 110.1, 105.1, 27.3, 27.1, 26.1 ppm.

Preparation of S1d: [91]

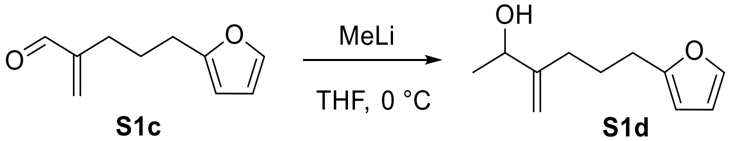

To a stirred solution of **S1c** (1.64 g, 10.0 mmol) in THF (50 mL) was added methyllithium (1.6 M, 9.4 mL, 15 mmol) dropwise at 0 °C. The resulting solution was stirred at 0 °C for one hour, and then quenched with saturated NH_4Cl (aq.). The organic layer was separated, and the aqueous layer was extracted with Et_2O three times. The combined organic extracts were dried over anhydrous $MgSO_4$ and removed of volatiles in vacuo. The residue was purified by flash silica gel column chromatography (5% to 10% EtOAc in hexanes) to afford **S1d** (1.59 g, 89% yield) as a colorless oil.

R_f: 0.33 (20% EtOAc in hexanes)

^{1}H NMR (400 MHz, C_6D_6) *δ* 7.13 (m, 1H), 6.13 (dd, *J* = 3.2, 1.9 Hz, 1H), 5.92 – 5.86 (dd, *J* = 3.1, 0.8 Hz, 1H), 5.01 (s, 1H), 4.74 – 4.67 (m, 1H), 4.02 – 3.90 (m, 1H), 2..49 (t, *J* = 7.5, 2H), 2.04 – 1.93 (m, 1H), 1.92 – 1.81 (m, 1H), 1.77 – 1.67 (m, 2H), 1.10 (d, *J* = 6.4 Hz, 3H) ppm; ^{13}C NMR (100 MHz, C_6D_6) δ 156.0, 152.9, 140.7, 110.1, 107.9, 104.9, 70.4, 30.9, 27.6, 26.48, 22.1 ppm.

HRMS-ESI (*m/z*) $[M+Na]^+$: Calcd for $C_{11}H_{16}O_2Na$: 203.1043; Found, 203.1043.

Preparation of S1e: [92]

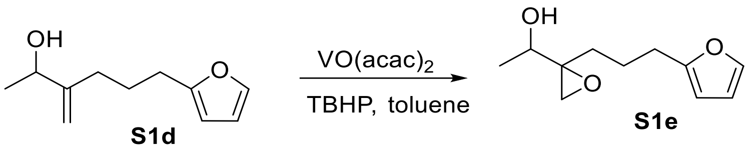

To a stirred solution of **S1d** (1.59 g, 8.9 mmol) in toluene (30 mL) was added $VO(acac)_2$ (0.24 g, 0.89 mmol) in one portion. After 10 min, TBHP (5~6 M, 2 mL) in decane was added. The mixture was stirred at room temperature for 4 h and then filtered through a pad of silica gel. The filtrate was concentrated by rotary evaporation to afford **S1e** which was used in the next step without further purification. R_f: 0.37 (20% EtOAc in hexane).

Preparation of 11: [77]

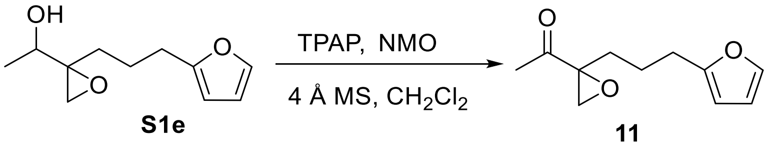

A solution of crude **S1e** in anhydrous CH_2Cl_2 (20 mL) was added to a stirred mixture of 4Å MS (5 g), TPAP (312.8 mg, 0.89 mmol), NMO (3.13 g, 26.7 mmol). The resulting mixture was stirred at room temperature for 2 h and then filtered through a pad of silica gel. The filtrate was concentrated by rotary evaporation. The residue was purified by flash column chromatography (eluent: 5% to 10% EtOAc in hexane) to afford **11** (1.11 g, 65% yield over 2 steps) as a colorless oil.

R_f: 0.48 (20% EtOAc in hexane)

^{1}H NMR (400 MHz, C_6D_6) δ 7.10 (d, *J* = 1.9 Hz, 1H), 6.10 (dd, *J* = 3.1, 1.9 Hz, 1H), 5.87 (dd, *J* = 3.1, 1.0 Hz, 1H), 2.45 (t, *J* = 7.5 Hz, 2H), 2.12 (s, 2H), 2.05 (m, 1H), 1.68 (m, 2H), 1.61 (s, 3H), 1.34 (m,

1H) ppm; ^{13}C NMR (100 MHz, C_6D_6) δ 205.8, 155.5, 140.8, 110.1, 105.1, 62.0, 49.8, 29.4, 27.8, 23.3, 22.8 ppm.

HRMS-ESI (m/z) $[M+Na]^+$: Calcd for $C_{11}H_{14}O_3Na$: 217.0835; Found, 217.0837.

Synthesis of optically enriched Substrate (+)-11 from S1d

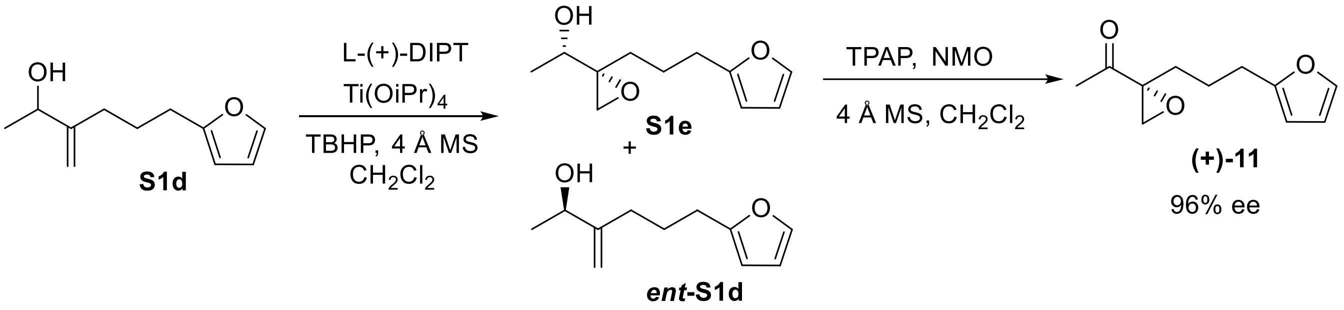

To *L*-(+)-diisopropyl-tartrate (53 μL, 0.250 mmol), molecular sieves (4 Å, 150 mg) in DCM (8.3 mL) at -30 °C, was added Ti(*i*-PrO)$_4$ (49 μL, 0.166 mmol). After 20 minutes, a solution of **S1d** (152 mg, 0.843 mmol) in DCM (2.0 mL) was added. After 20 minutes, TBHP (91 μL, 5.5 M in decane, 0.499 mmol) was added dropwise. The reaction mixture was kept at this temperature for 2 h. TLC analysis confirmed around 50% conversion, consistent with the expected progress of the kinetic resolution. A mixture of ferrous sulfate, tartaric acid and water (1 g/ 1 g/ 50 ml) was added, then the mixture was allowed to warm to rt and stirred for one hour. After filtration through celite, the filtrate was extracted with DCM (3 x 30 mL), and dried over $MgSO_4$. The crude mixture was purified by flash column chromatography provided impure **S1e** (77.9 mg) and recovered **S1d**.

A solution of the above impure **S1e** (77.9 mg) in anhydrous CH_2Cl_2 (5 mL) was added to a stirred mixture of 4Å MS (500 mg), TPAP (14.0 mg, 0.040 mmol), NMO (140 mg, 1.2 mmol). The resulting mixture was stirred at room temperature for 1 h and then filtered through a pad of silica gel. The filtrate was concentrated by rotary evaporation. The residue was purified by flash column chromatography (eluent: 5% to 10% EtOAc in hexane) to afford (+)-**11** (45.0 mg, 29% yield for 2 steps) as a colorless oil.

$[\alpha]_D^{23}$ = + 52.9 (1.96, $CHCl_3$).

HPLC analysis: Daicel Chiralpak® OJ-3, n-Hexane/i-PrOH = 90/10, 1.0 mL/min, 220 nm; tr (major) = 14.575 min, tr (minor) = 15.630 min, 95.5% *ee*.

Other spectral characteristics are consistent with those of **11**.

Sample HY-8-27 & 38
Column: Daicel Chiralcel® OJ-3
Condition: Hexane / iso-Propanol = 90 / 10, flowrate: 1.0 mL / min
Wavelength: 220 nm
Instrument: Agilent 1260 Infinity II

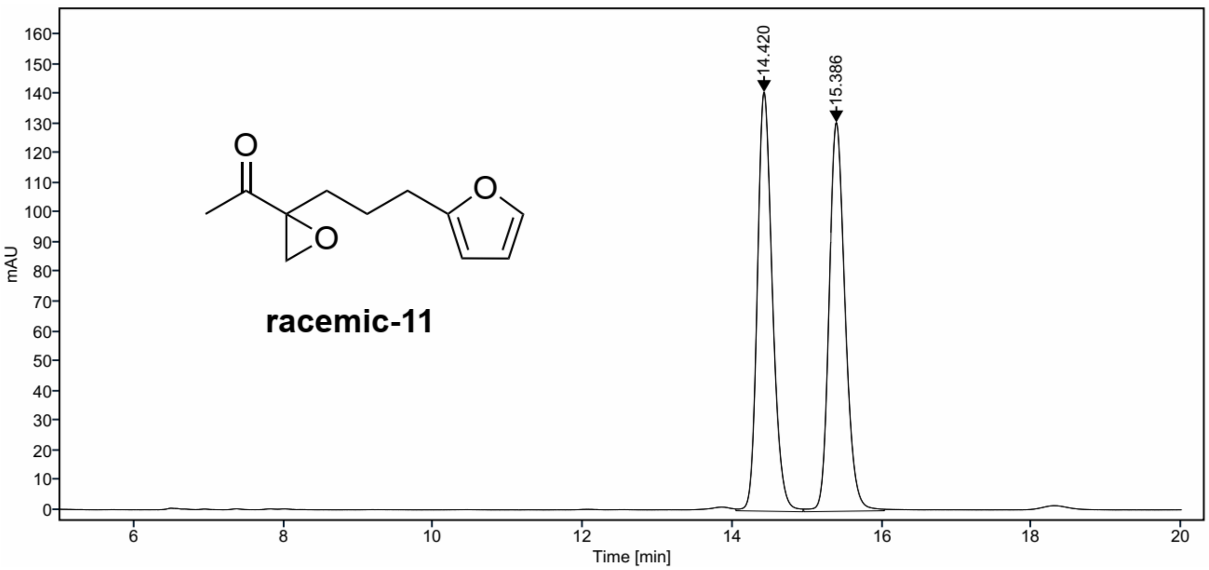

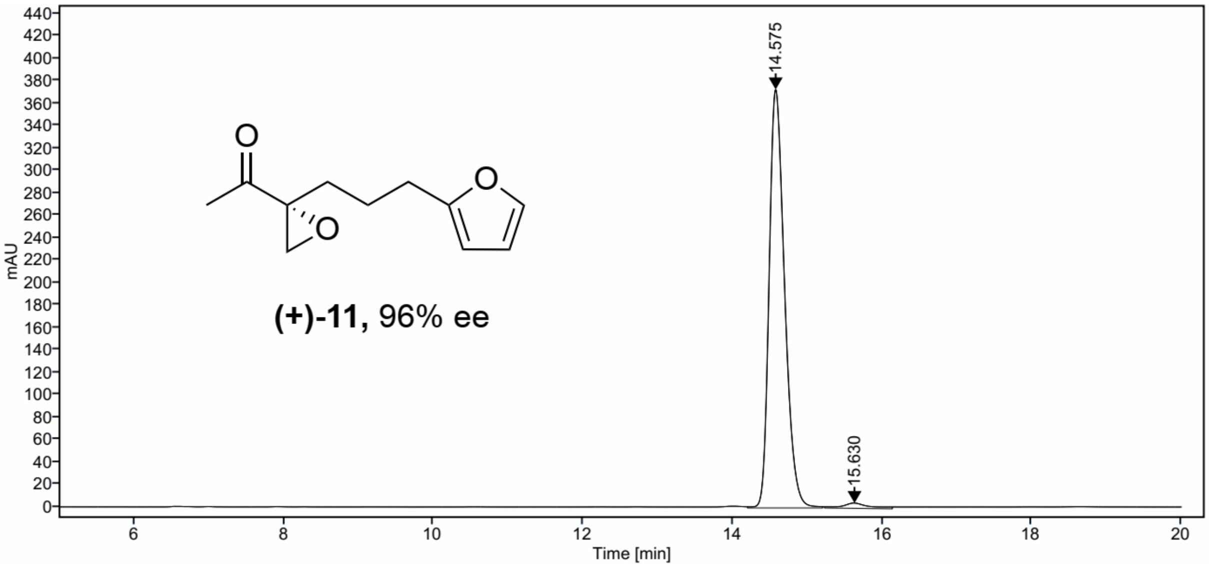

Retention Time [min]	Peak Height	Peak Area	Area%
14.420	140.967	1972.145	50.285
15.386	130.832	1949.776	49.715

Retention Time [min]	Peak Height	Peak Area	Area%
14.575	373.142	5357.105	97.756
15.630	4.731	122.961	2.244

Synthesis of Substrate 12 from S2

Preparation of S2a

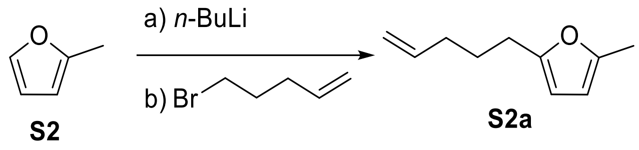

According to the general procedure for the preparation of **S1a**, 2-methylfuran (1.97 g, 24.0 mmol) was treated with *n*-BuLi (2.4 M, 10.0 mL, 24.0 mmol), and 5-bromo-1-pentene (2.98 g, 20.0 mmol) in anhydrous THF (100 mL) and worked up. The crude **S2a** thus obtained was then used in the following step without further purification.

R_f: 0.63 (hexane);

^{1}H NMR (400 MHz, $CDCl_3$) δ 5.89 – 5.73 (m, 3H), 5.09 – 4.91 (m, 2H), 2.58 (t, J = 7.6 Hz, 2H), 2.25 (s, 3H), 2.14 – 2.06 (m, 2H), 1.88 – 1.67 (m, 2H) ppm.

The spectral characteristics are consistent with those of **S2a** in the literature. [93]

Preparation of S2b

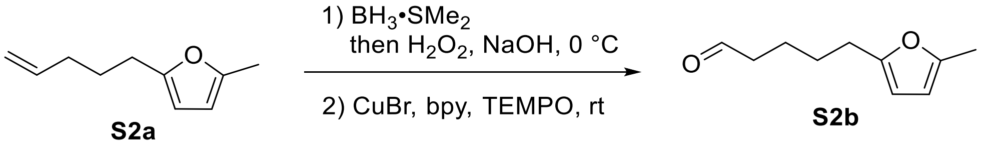

According to the general procedure for the preparation of **S1b**, treatment of crude **S2a** with $BH_3·SMe_2$ (10 M, 4.0 mL, 40 mmol) in anhydrous THF (100 mL) and workup, followed by treatment with CuBr (0.290 g, 2.0 mmol), bpy (0.310 g, 2.0 mmol), and TEMPO (0.320 g, 2.0 mmol) in CH_3CN (50 mL) and workup, provided **S2b**, which was used the following step without further purification.

R_f: 0.53 (10% EtOAc in hexane).

Preparation of S2c

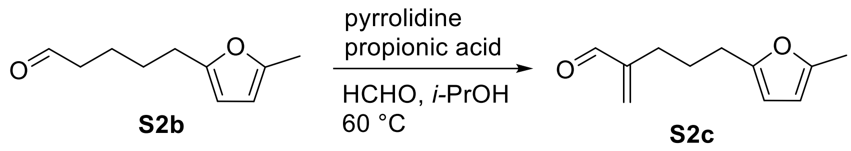

According to the general procedure for the preparation of **S1c**, treatment of crude **S2b**, pyrrolidine (0.16 mL, 2.0 mmol), propionic acid (0.16 mL, 2.02 mmol), and formaldehyde (37% aq., 1.6 mL, 20 mmol) in *i*PrOH (10 mL) followed by workup and purification by flash silica gel column chromatography (5% EtOAc in hexanes) provided aldehyde **S2c** (1.81 g, 51 % yield over 4 steps) as a colorless oil.

R_f: 0.59 (10% EtOAc in hexanes).

^{1}H NMR (400 MHz, $CDCl_3$) δ 9.54 (s, 1H), 6.27 (d, *J* = 1.0 Hz, 1H), 6.01 (d, *J* = 0.8 Hz, 1H), 5.89 – 5.79 (m, 2H), 2.58 (t, *J* = 7.5 Hz, 2H), 2.36 – 2.27 (m, 2H), 2.24 (s, 3H), 1.83 – 1.73 (m, 2H) ppm; ^{13}C NMR (101 MHz, $CDCl_3$) δ 194.8, 153.8, 150.4, 149.9, 134.4, 105.9, 105.8, 27.7, 27.4, 26.3, 13.6 ppm.

Preparation of S2d

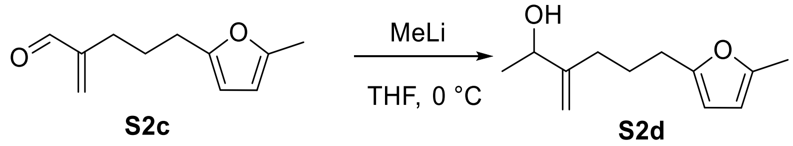

According to the general procedure for the preparation of **S1d**, treatment of **S2c** (1.78 g, 10 mmol) with methyllithium (1.6 M, 7.6 mL, 12.2 mmol) in anhydrous THF (50 mL) followed by workup provided S2d, which was used in the following step without further purification.

R_f: 0.42 (20% EtOAc in hexane).

Preparation of S2e

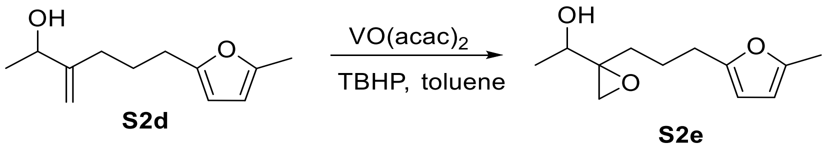

According to the general procedure for the preparation of **S1e**, treatment of crude **S2d** with $VO(acac)_2$ (0.52 g, 2.0 mmol), and TBHP (5~6 M, 3 mL) in toluene (50 mL), followed by workup provided **S2e**, which was used in the next step without further purification.

R_f: 0.38 (20% EtOAc in hexanes).

^1H NMR (400 MHz, $CDCl_3$) δ 5.84 (q, *J* = 3.2 Hz, 2H), 3.93 (qd, *J* = 6.4, 1.7 Hz, 1H), 2.88 (d, *J* = 4.6 Hz, 1H), 2.63 (d, *J* = 4.6 Hz, 1H), 2.60 – 2.51 (m, 2H), 2.24 (s, 3H), 2.18 – 2.04 (m, 1H), 1.80 – 1.61 (m, 4H), 1.22 (d, *J* = 6.3 Hz, 3H) ppm; ^{13}C NMR (101 MHz, $CDCl_3$) δ 153.7, 150.5, 105.9, 105.8, 66.1, 62.5, 48.3, 30.7, 28.2, 22.8, 18.7, 13.6 ppm.

Preparation of 12

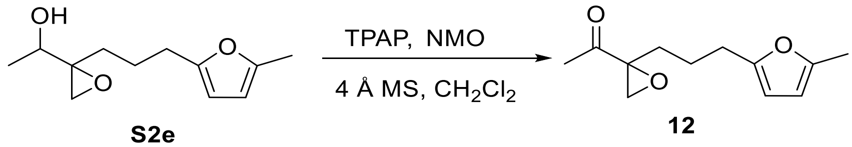

According to the general procedure for preparation of **11**, treatment of crude **S2e** with TPAP (351.4 mg, 1.000 mmol), NMO (3.51 g, 30.0 mmol), and 4Å MS (5 g), in CH_2Cl_2 (50 mL) followed by workup, and purification by flash column chromatography (eluent: 5% to 10% EtOAc in hexane) afforded **12** (1.22 g, 59% yield over 3 steps) as a pale yellow oil.

R_f: 0.48 (10% EtOAc in hexane).

^1H NMR (600 MHz, $CDCl_3$) δ 5.85 (d, *J* = 3.0 Hz, 1H), 5.82 (dd, *J* = 3.0, 1.1 Hz, 1H), 2.93 (d, *J* = 4.9 Hz, 1H), 2.87 (d, *J* = 4.9 Hz, 1H), 2.58 (t, *J* = 7.4 Hz, 2H), 2.23 (d, *J* = 1.0 Hz, 3H), 2.19 (ddd, *J* = 13.9, 10.7, 4.8 Hz, 1H), 2.03 (s, 3H), 1.77 – 1.70 (m, 1H), 1.70 – 1.63 (m, 1H), 1.63 – 1.58 (m, 1H) ppm; ^{13}C NMR (151 MHz, $CDCl_3$) δ 207.8, 153.7, 150.5, 105.9, 105.7, 62.7, 50.9, 29.7, 28.1, 24.0, 23.5, 13.6 ppm.

HRMS-ESI (m/z) $[M+Na]^+$: Calcd for $C_{12}H_{16}O_3Na$: 231.0992; Found, 231.0993.

Synthesis of Substrate 13 from S3

Preparation of S3a

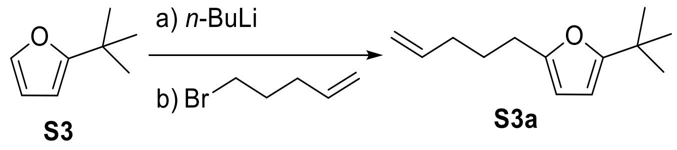

According to the general procedure for the preparation of **S1a**, 2-*tert*-butylfuran (2.48 g, 20.0 mmol) was treated with *n*-BuLi (2.4 M, 8.6 mL, 21 mmol), and 5-bromo-1-pentene (2.98 g, 20.0 mmol) in anhydrous THF (100 mL), followed by workup. The crude **S3a** was used in the next step without further purification. R_f: 0.71 (hexane).

Preparation of S3b

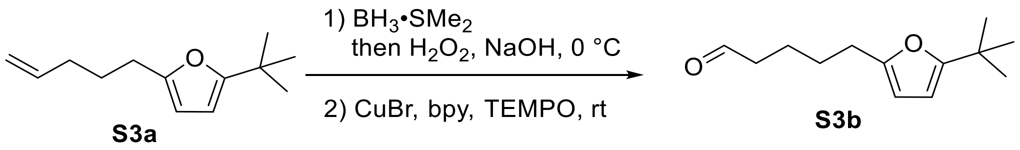

According to the general procedure for the preparation of **S1b**, treatment of crude **S3a** with $BH_3{\cdot}SMe_2$ (4.0 mL, 10 M, 40 mmol) in anhydrous THF (100 mL) followed by workup; and then further treatment with CuBr (0.290 g, 2.0 mmol), bpy (0.310 g, 2.0 mmol), and TEMPO (0.320 g, 2.0 mmol) in CH_3CN (50 mL) followed by workup provided **S3b**, which was used in the next step without further purification.

R_f: 0.63 (10% EtOAc in hexane).

Preparation of S3c

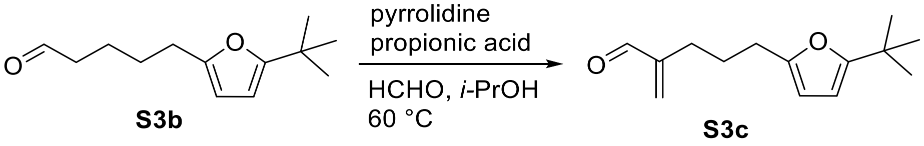

According to the general procedure for the preparation of **S1c**, treatment of crude **S3b** in *i*PrOH (10 mL) with pyrrolidine (0.16 ml, 2.0 mmol), propionic acid (0.16 ml, 2.02 mmol), and formaldehyde (37% aq., 1.6 ml, 20 mmol), followed by workup and purification by flash silica gel column chromatography (5% EtOAc in hexanes) to give aldehyde **S3c** (2.24 g, 51 % yield over 4 steps) as a colorless oil.

R_f: 0.65 (10% EtOAc in hexanes).

^{1}H NMR (400 MHz, C_6D_6) δ 9.20 (s, 1H), 5.86 – 5.80 (m, 2H), 5.54 (s, 1H), 5.22 (s, 1H), 2.42 (t, *J* = 7.5 Hz, 2H), 2.14 (d, *J* = 7.6 Hz, 2H), 1.69 – 1.59 (m, 2H), 1.26 (s, 9H); ^{13}C NMR (101 MHz, C_6D_6) δ 193.0, 162.2, 153.5, 149.7, 132.4, 105.3, 102.4, 32.3, 28.9, 27.5, 27.2, 26.3.

Preparation of S3d

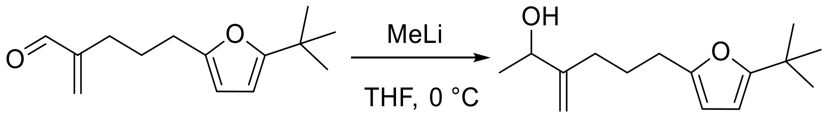

S3c **S3d**

According to the general procedure for the preparation of **S1d**, treatment of **S3c** (2.24 g, 10.1 mmol) with methyllithium (1.6 M, 7.6 mL, 12.2 mmol) in anhydrous THF (50 mL), followed by workup provided **S3d**, which was used in the following step without further purification.

R_f: 0.38 (20% EtOAc in hexane).

Preparation of S3e

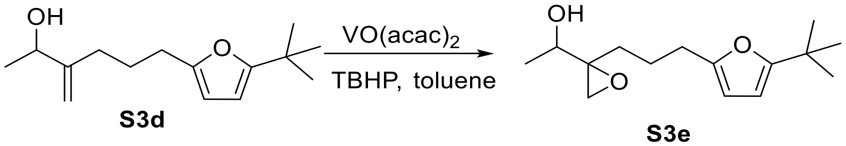

S3d **S3e**

According to the general procedure for preparation of **S1e**, treatment of crude **S3d** in toluene (50 mL) with $VO(acac)_2$ (0.52 g, 2.0 mmol), and TBHP (5~6 M, 3 mL), followed by workup provided **S3e**, which was used in the next step without further purification.

R_f: 0.36 (20% EtOAc in hexanes).

Preparation of 13

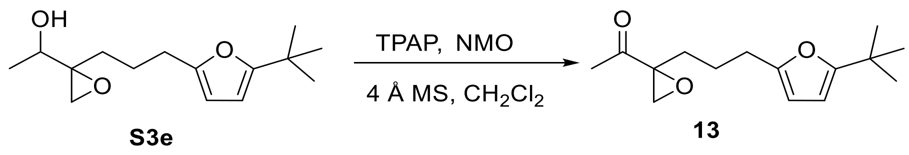

According to the general procedure for preparation of **11**, crude **S3e** in CH_2Cl_2 (50 mL) was treated with TPAP (351.4 mg, 1.000 mmol), NMO (3.51 g, 30.0 mmol), and 4Å MS (5 g). After workup and purification by flash column chromatography (eluent: 5% to 10% EtOAc in hexane), **13** was obtained (2.06 g, 81% yield over 3 steps) as pale yellow oil.

R_f: 0.55 (20% EtOAc in hexane).

^{1}H NMR (400 MHz, C_6D_6) δ 5.85 (d, J = 3.1 Hz, 1H), 5.83 (d, J = 3.0 Hz, 1H), 2.48 (t, J = 7.5 Hz, 2H), 2.16 (s, 2H), 2.14 – 2.04 (m, 1H), 1.80 – 1.64 (m, 2H), 1.62 (s, 3H), 1.43 – 1.32 (m, 1H), 1.25 (s, 9H) ppm; ^{13}C NMR (100 MHz, C_6D_6) δ 205.7, 162.2, 153.4, 105.3, 102.4, 62.0, 49.9, 32.3, 29.5, 28.9, 27.9, 23.4, 22.8 ppm.

HRMS-ESI (m/z) $[M+Na]^+$: Calcd for $C_{15}H_{22}O_3Na$, 273.1461; Found 273.1463.

Reaction of Compound 11: *General Procedure for enolsilane formation and (4+3) cycloaddition*

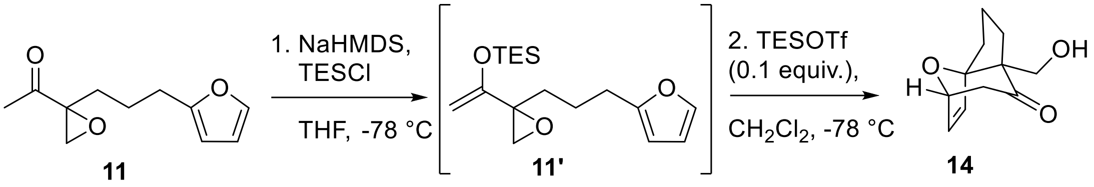

To a stirred solution of **11** (22.0 mg, 0.113 mmol) in anhydrous THF (1 mL) was added NaHMDS (0.113 mL, 2.0 M in THF, 0.226 mmol) at -78 °C. The resulting solution was stirred at -78 °C for 20 min, and then TESCl (57.0 µL, 0.340 mmol) was added. After 1 h, reaction monitoring by TLC showed that the reaction was complete. Saturated $NaHCO_3$ (aq., 1 mL) was added at –78 °C and allowed to warm to room temperature. The reaction mixture was extracted with Et_2O three times. The combined organics were dried over anhydrous $MgSO_4$, and concentrated in vacuo to give the enolsilane **11′**, which was used in next step without further purification. R_f: 0.79 (5% hexanes).

To the solution of crude **11′** in anhydrous CH_2Cl_2 (3 mL), TESOTf (56.6 µL, freshly prepared 0.2 M solution in CH_2Cl_2, 0.0113 mmol) was added at –78 °C. After stirring at –78 °C for 30 min, $Et_3N{\cdot}3HF$ (0.1 mL) was added, and the mixture was warmed to room temperature over 1 hour. Saturated $NaHCO_3$ (aq., 1 mL) was added to the reaction until the reaction mixture ceased bubbling. The mixture was extracted with Et_2O three times; the combined organics were dried over anhydrous $MgSO_4$, and concentrated by in vacuo. The residue was purified by flash silica gel column chromatography (20 % EtOAc in hexanes) to afford **14** (17.0 mg, 77% yield) as a colorless oil.

R_f: 0.39 (30% EtOAc in hexanes).

^{1}H NMR (600 MHz, C_6D_6) δ 5.75 (dd, *J* = 5.9, 0.7 Hz, 1H), 5.59 (dd, *J* = 5.9, 1.9 Hz, 1H), 4.38 (dd, *J* = 6.1, 1.9 Hz, 1H), 3.41 – 3.35 (m, 1H), 3.32 (dd, *J* = 11.4, 8.8 Hz, 1H), 2.89 (dd, *J* = 9.0, 3.9 Hz, 1H), 2.32 (dd, *J* = 17.5, 6.0 Hz, 1H), 2.05 (ddd, *J* = 12.8, 8.7, 2.9 Hz, 1H), 2.02 – 1.95 (m, 1H), 1.86 – 1.68 (m, 4H), 1.65 – 1.54 (m, 1H) ppm; ^{13}C NMR (150 MHz, C_6D_6) δ 213.8, 134.6, 134.3, 95.3, 77.2, 68.3, 63.8, 41.8, 31.4, 29.8, 22.0 ppm.

HRMS-ESI (m/z) $[M+Na]^+$: Calcd for $C_{11}H_{14}NaO_3$, 217.0835; found, 217.0834.

Reaction of (+)-11:

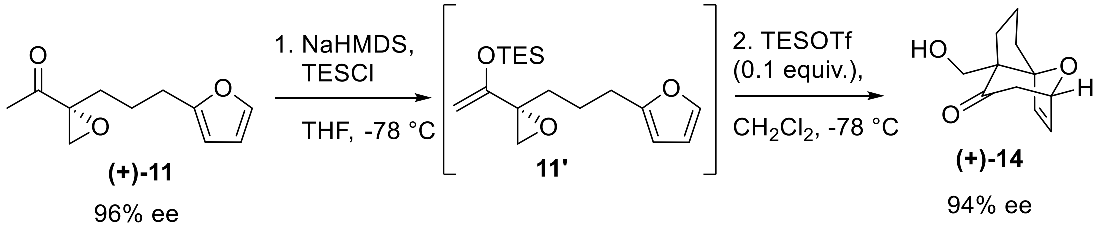

According to the general procedure for reaction of **11**, treatment of (+)-**11** (24.3 mg, 0.125 mmol) in anhydrous THF (2.8 mL) with NaHMDS (0.250 mL, 1.0 M in THF, 0.250 mmol), and TESCl (0.063 mL, 0.375 mmol) followed by workup, provided enolsilane **11′,** which was used in the next step without further purification.

Crude **11′** in CH_2Cl_2 (3.7 mL) was treated with TESOTf (freshly prepared 0.2 M solution in CH_2Cl_2, 0.063 mL, 0.013 mmol). Workup and purification by flash silica gel chromatography (30% EtOAc in hexane) provided (+)-**14** (17.5 mg, 72% yield) as a colorless oil.

$[\alpha]_D^{23}$ = +90.6 (0.63, $CHCl_3$).

HPLC analysis: Daicel Chiralpak® OJ-3, n-Hexane/i-PrOH = 80/20, 1.0 mL/min, 220 nm; tr (major) = 8.123 min, tr (major) = 9.513 min, 94.2% ee.

Other spectral characteristics are the same as those of **14**.

Sample HY-8-31 & 42
Column: Daicel Chiralcel® OJ-3
Condition: Hexane / iso-Propanol = 80 / 20, flowrate: 1.0 mL / min
Wavelength: 220 nm
Instrument: Agilent 1260 Infinity II

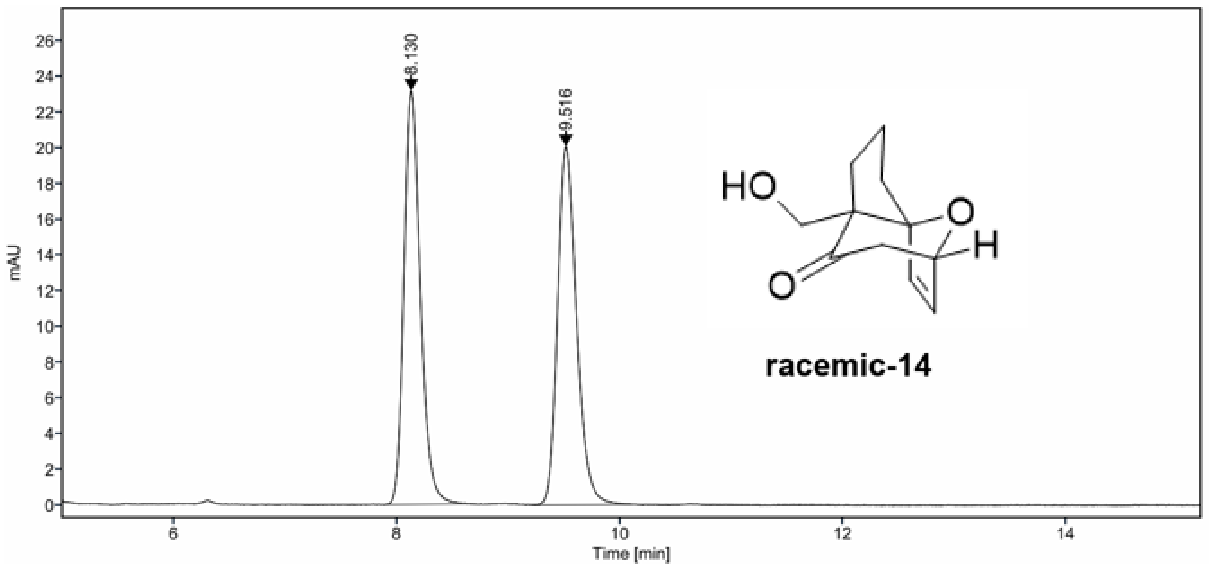

racemic-14

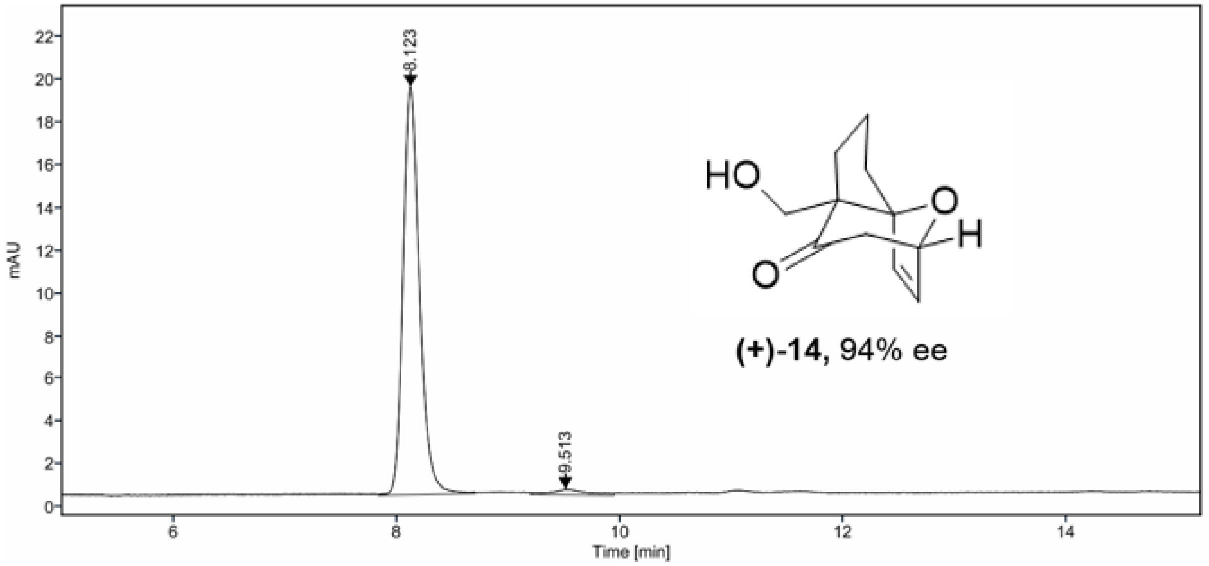

(+)-14, 94% ee

Retention Time [min]	Peak Height	Peak Area	Area%
8.130	23.159	236.901	49.847
9.516	20.073	238.360	50.153

Retention Time [min]	Peak Height	Peak Area	Area%
8.123	19.086	195.687	97.090
9.513	0.265	5.866	2.910

Reaction of 12:

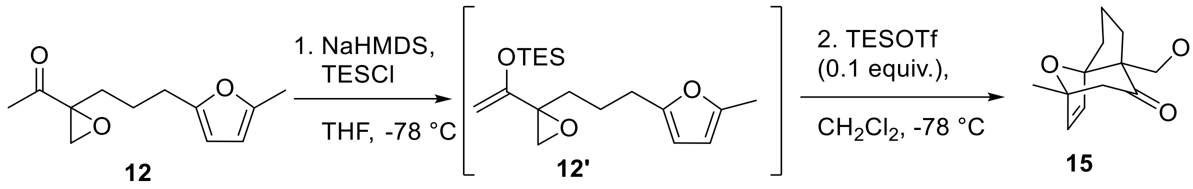

According to the general procedure for reaction of **11**, treatment of **12** (42.3 mg, 0.203 mmol) in anhydrous THF (4.0 mL) with NaHMDS (0.406 mL, 1.0 M in THF, 0.406 mmol), and TESCl (0.102 mL, 0.609 mmol) followed by workup, provided enolsilane **12′,** which was used in the next step without further purification.

Crude **12′** in CH_2Cl_2 (7.0 mL) was treated with TESOTf (freshly prepared 0.2 M solution in CH_2Cl_2, 0.1 mL, 0.0203 mmol). After workup and purification by flash silica gel chromatography (20% EtOAc in hexane) provided **15** (28.0 mg, 71% yield) as a colorless oil.

R_f: 0.30 (30% EtOAc in hexane).

^{1}H NMR (600 MHz, $CDCl_3$) δ 6.09 (d, J = 5.8 Hz, 1H), 6.02 (d, J = 5.8 Hz, 1H), 3.52 (dd, J = 11.5, 2.9 Hz, 1H), 3.41 (dd, J = 11.5, 9.2 Hz, 1H), 2.90 (dd, J = 9.4, 3.6 Hz, 1H), 2.52 (d, J = 17.4 Hz, 1H), 2.31 (d, J = 17.4 Hz, 1H), 2.13 – 2.04 (m, 2H), 2.04 – 1.83 (m, 4H), 1.46 (s, 3H) ppm; ^{13}C NMR (151 MHz, $CDCl_3$) δ 215.5, 137.6, 133.7, 95.7, 83.0, 65.6, 63.3, 47.8, 31.1, 29.0, 24.2, 21.3 ppm.

HRMS-ESI (m/z) $[M+Na]^+$: Calcd for $C_{12}H_{16}NaO_3$, 231.0992; found, 231.0990.

Reaction of 13:

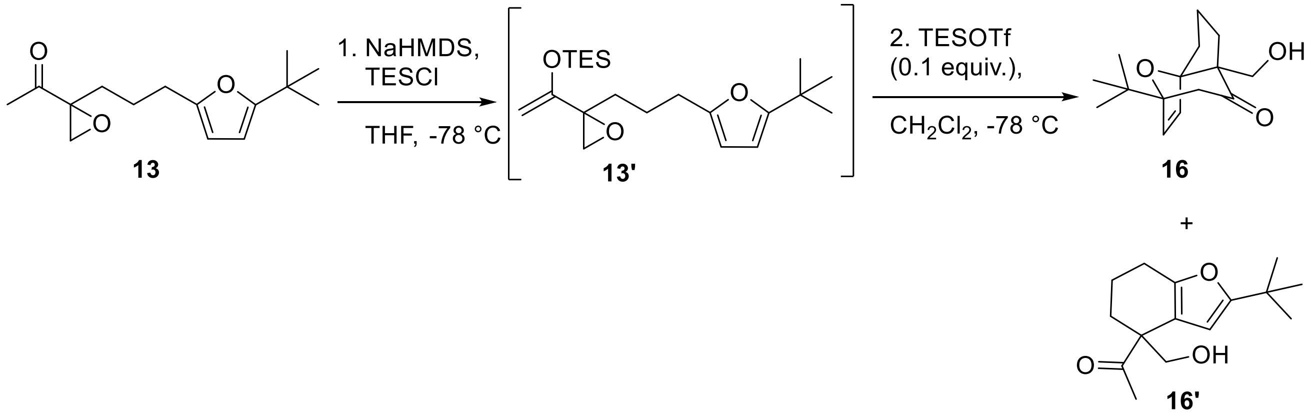

According to the general procedure for reaction of **11**, treatment of **13** (24.2 mg, 0.0967 mmol) in anhydrous THF (1 mL) with NaHMDS (0.193 mL, 1.0 M in THF, 0.193 mmol), and TESCl (48.7 µL, 0.290 mmol) followed by workup, provided enolsilane **13′,** which was used in the next step without further purification.

Crude **13′** in CH_2Cl_2 (3 mL) was treated with TESOTf (freshly prepared 0.2 M solution in CH_2Cl_2, 48.4 µL). After workup and purification by flash silica gel chromatography (20 % EtOAc in hexanes) **16** was obtained as a colorless oil (13.8 mg, 57% yield) and **16’** was obtained as a colorless oil (7.1 mg, 29% yield).

16: R_f: 0.55 (30% EtOAc in hexane).

^{1}H NMR (600 MHz, $CDCl_3$) δ 6.14 (d, J = 5.9 Hz, 1H), 6.04 (d, J = 5.9 Hz, 1H), 3.52 (dd, J = 11.4, 3.4, 1H), 3.39 (dd, J = 11.4, 9.6 Hz, 1H), 2.94 (dd, J = 9.7, 3.5 Hz, 1H), 2.64 (d, J = 17.3 Hz, 1H), 2.23 (d, J = 17.3 Hz, 1H), 2.12 – 2.02 (m, 2H), 2.00 – 1.83 (m, 4H), 0.96 (s, 9H) ppm; ^{13}C NMR (151 MHz, $CDCl_3$) δ 217.0, 135.4, 132.9, 95.1, 90.7, 65.6, 63.3, 41.6, 34.7, 31.0, 29.0, 25.1, 21.5 ppm.

HRMS-ESI (m/z) $[M+Na]^+$: Calcd for $C_{15}H_{22}O_3Na$, 273.1461; Found 273.1461.

16': R_f: 0.52 (20% EtOAc in hexane).

^{1}H NMR (400 MHz, $CDCl_3$) δ 5.05 (d, *J* = 9.3 Hz, 1H), 3.93 (s, 1H), 3.50 (d, *J* = 4.9 Hz, 1H), 2.79 (d, *J* = 9.3 Hz, 1H), 2.07 (s, 3H), 1.87 (d, *J* = 14.0 Hz, 1H), 1.66 (dd, *J* = 14.2, 5.3 Hz, 2H), 1.52-1.22 (m, 2H), 1.05 (s, 9H), 1.02-0.82 (m, 1H) ppm; ^{13}C NMR (100 MHz, $CDCl_3$) δ 204.8, 112.0, 104.0, 89.4, 74.5, 68.2, 37.0, 36.9, 35.4, 32.8, 27.6, 25.1, 23.0 ppm.

HRMS-EI (*m*/*z*) [M^+] Calcd for $C_{15}H_{20}O_3$, 250.1569; Found 250.1569.

3.2. Preparation of substrates for intramolecular (4+3) cycloaddition-2

Preparation of allylic alcohol S6: [94, 95]

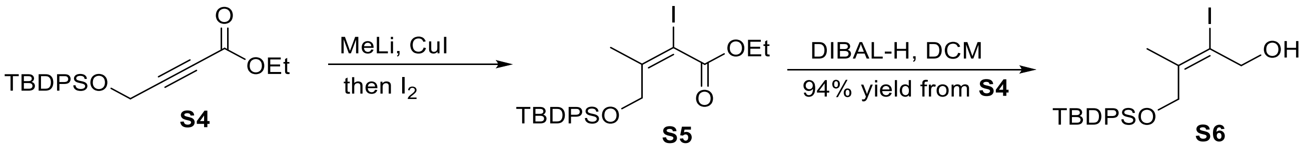

At 0 °C, MeLi (72.6 mL, 3.1 M in diethoxymethane, 225 mmol) was added to a suspension of CuI (21.5 g, 113 mmol) in THF (400 mL). After 30 min, the mixture was cooled to -78 °C, then **S4** [96] (27.5 g, 75 mmol) in THF (50 mL) was added. The reaction was kept at this temperature for 4 h, then I_2 (57.2 g, 225 mmol) in THF (50 mL) was added. After 15 min, the reaction mixture was allowed to warm to 0 °C, quenched with saturated aqueous $Na_2S_2O_3$ and aqueous NH_4Cl. The resulting mixture was extracted with Et_2O (3 x 500 mL). The combined organic extracts were washed with brine, dried over anhydrous $MgSO_4$, and concentrated in vacuo. **S5** thus obtained was used directly in next step without further purification.

To a solution of crude **S5** in DCM (300 mL) at –78 °C was added DIBAL-H (188 mL, 1 M in hexane, 188 mmol). After 30 min, TLC monitoring showed that the reaction was complete. The reaction was quenched by the addition of ethyl acetate (15 mL) carefully. Then saturated potassium sodium tartrate solution (250 mL) was added, the mixture was stirred vigorously overnight. The reaction mixture was separated, and the aqueous layer was extracted with Et_2O (3 x 200 mL). The combined organic extracts were washed with brine, dried over anhydrous $MgSO_4$. The volatiles were removed under reduced pressure and the residue was purified by flash column chromatography (EtOAc/Hexane: 1/30 to 1/20) to give **S6** a colorless oil (32.9 g, 94% yield over 2 steps).

Rf: 0.27 (10% EtOAc in Hexane)

^{1}H NMR (400 MHz, $CDCl_3$) δ 7.71 – 7.59 (m, 4H), 7.50 – 7.34 (m, 6H), 4.29 (s, 2H), 4.12 (d, *J* = 6.5 Hz, 2H), 2.04 (s, 3H), 1.05 (s, 9H) ppm; ^{13}C NMR (101 MHz, $CDCl_3$) *δ* 143.3, 135.8, 135.7, 133.1, 130.1, 128.0, 107.6, 67.5, 62.6, 27.7, 26.8, 19.3 ppm.

HRMS-ESI (*m/z*) $[M+Na]^+$: Calcd for $C_{21}H_{27}NaIO_2Si$, 489.0717; found, 489.0720.

Preparation of allylic alcohol S7: [97]

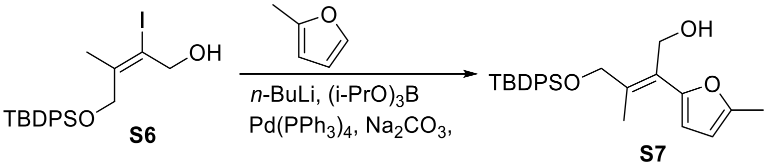

To a solution of 2-methylfuran (7.50 mL, 75.6 mmol) in anhydrous dimethoxyethane (150 mL) at −72 °C (dry ice/ ethanol bath) was added n-BuLi (36.6 mL, 2.08 M in Hexane, 76.2 mmol). The reaction mixture was allowed to stir and warm to rt over 4 h. The reaction mixture was then cooled to −40 °C, to which (i-PrO)$_3$B (18.6 mL, 80.5 mmol) was added. The resulting mixture was allowed to warm to rt and stirred overnight. Water (degassed, 150 mL) was added, the reaction was stirred for another 2 h. Then Na_2CO_3 (12.8 g, 121 mmol), **S6** (28.2 g, 60.5 mmol) in dimethoxyethane (190 mL), $Pd(PPh_3)_4$ (3.5 g, 3.03 mmol) were added sequentially. After addition, the reaction mixture was heated to 80 °C and stirred for 2 h. After the reaction was cooled to rt, water (100 mL) and Et_2O (100 mL) were added. The mixture was separated, and the aqueous layer was back-extracted with Et_2O (3 x 100 mL). The combined organic layers were dried over anhydrous $MgSO_4$. The volatiles were removed under reduced pressure and the residue was purified by flash column chromatography (EtOAc/Hexane: 1/30 to 1/20) to give **S7** as a colorless oil (24.8 g, 98% yield).

Rf: 0.27 (10% EtOAc in Hexane)

^{1}H NMR (400 MHz, $CDCl_3$) *δ* 7.71 (dd, *J* = 7.8, 1.5 Hz, 4H), 7.51 – 7.37 (m, 7H), 6.25 (d, *J* = 3.2 Hz, 1H), 6.06 – 5.98 (m, 1H), 4.39 (s, 2H), 4.31 (s, 2H), 2.32 (s, 3H), 2.02 (s, 3H), 1.07 (s, 9H) ppm; ^{13}C

NMR (101 MHz, $CDCl_3$) δ 152.0, 151.2, 136.0, 135.8, 133.4, 130.0, 127.9, 127.8, 110.9, 107.2, 65.5, 60.5, 27.0, 19.5, 19.3, 13.8 ppm.

HRMS-ESI (*m/z*) $[M+Na]^+$: Calcd for $C_{26}H_{32}NaO_3Si$, 443.2013; found, 443.2011.

Preparation of allylic alcohol S10: [98, 99]

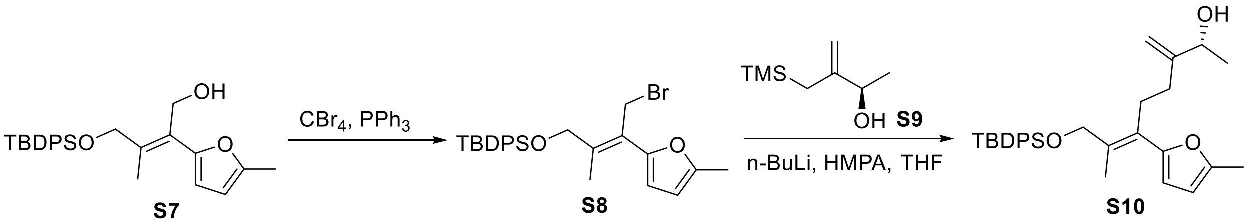

S7 S8 S10

To **S7** (10.5 g, 25.0 mmol), PPh_3 (7.86 g, 30.0 mmol) in DCM (100 mL) at -15 °C (ice/ ethanol bath) was added a solution of CBr_4 (9.95 g, 30.0 mmol) in DCM (15 mL). After 1 h , the reaction mixture was poured into a flask containing hexane (300 mL). A lot of solid precipitates appeared, which were filtered off. Then the filtrate was concentrated under reduced pressure. The residue was treated again with hexane (100 mL), solids appeared which were filtered off. This was repeated three times until no solids precipitated when treated with hexane. The filtrate was evacuated at high vacuum (2 mmHg) for 2 h to remove all the volatile impurities. The resultant crude product **S8** was used for following step directly.

To **S9** [13] (9.20 mL, 50.0 mmol) in anhydrous THF (150 mL) at –78 °C, was added *n*-BuLi (21.5 mL, 2.33 M in hexane, 50.0 mmol). After 30 minutes, crude **S8** in THF (30 mL), HMPA (34.8 mL, 200 mmol) was added to the reaction mixture. After 5 minutes, the reaction mixture was moved to an ice-water bath and allowed to warm to rt overnight. The reaction was quenched with HCl (100 mL, 1 N) and stirred for 10 minutes. The resulting mixture was extracted with Et_2O (3 x 100 mL). The The combined organic extracts were washed with saturated $NaHCO_3$ (aq.), brine, then dried over anhydrous $MgSO_4$. The volatiles were removed under reduced pressure, and the residue was purified by flash column chromatography (EtOAc/Hexane: 1/50 to 1/10) to give **S10** as a colorless oil (8.76 g, 72% yield).

Rf: 0.42 (10% EtOAc in Hexane), $[\alpha]_D^{25}$ = +3.80 (0.527, DCM).

^{1}H NMR (400 MHz, $CDCl_3$) δ 7.72 – 7.69 (m, 4H), 7.50 – 7.31 (m, 6H), 6.15 (d, J = 3.2 Hz, 1H), 6.00 – 5.97 (m, 1H), 4.95 (s, 1H), 4.65 (s, 1H), 4.30 (q, J = 8.33 Hz, 2H), 4.18 – 4.03 (m, 1H), 2.36 (t, J = 8.3 Hz, 2H), 2.30 (s, 3H), 2.04 (s, 3H), 2.01 – 1.85 (m, 2H), 1.35 (d, J = 4.1 Hz, 1H), 1.15 (d, J = 6.4 Hz, 3H), 1.06 (s, 9H) ppm; ^{13}C NMR (101 MHz, $CDCl_3$) δ 153.3, 153.0, 150.6, 135.8, 133.9, 132.5, 129.8, 127.8, 126.5, 110.2, 108.5, 106.9, 32.0, 29.8, 27.0, 22.2, 19.5, 18.5, 13.8 ppm

HRMS-ESI (*m/z*) $[M+Na]^+$: Calcd for $C_{31}H_{40}NaO_3Si$, 511.2639; found, 511.2635.

Preparation of epoxide S11: [76]

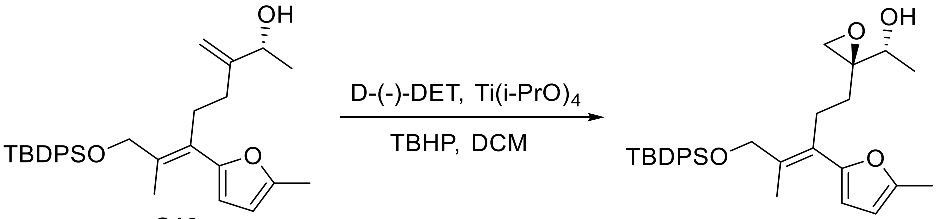

S10 **S11**

To D-(–)-DET (0.443 mL, 2.59 mmol), molecular sieves (4 Å, 2.11 g) in DCM (43 mL) at -30 °C, was added Ti(*i*-PrO)$_4$ (0.632 mL, 2.16 mmol). After 30 minutes, a solution of **S10** (2.11 g, 4.32 mmol) in DCM (15 mL) was added. After 1 h, TBHP (1.57 mL, 5.5 M in decane, 8.64 mmol) was added dropwise. The reaction mixture was kept at this temperature for 3 h. The reaction progress was monitored by TLC. When the reaction was deemed complete, a mixture of ferrous sulfate, tartaric acid and water (24 g/ 24 g/ 400 g, 40 mL) was added, then the mixture was allowed to warm to rt and stirred for 1 h. After filtration through celite, the filtrate was extracted with DCM (3 x 30 mL).

The combined DCM layers were concentrated. The residue was re-dissolved in Et_2O, and cooled to 0 °C. NaOH in brine (10 wt%, 30 mL) was added to the ethereal solution and stirred for 1 h. The organic layer was separated by separatory funnel, and the aqueous layer was back-extracted with Et_2O (3x 30 mL). The The combined organic extracts were washed with brine, and dried over anhydrous

$MgSO_4$. The volatiles were removed under reduced pressure, and the residue was purified by flash column chromatography (EtOAc/Hexane: 1/25 to 1/10) to give **S11** as a colorless oil (1.70 g, 78% yield).

R_f: 0.38 (20% EtOAc in Hexane), $[\alpha]_D^{25}$ = -0.920 (1.31, DCM).

^{1}H NMR (400 MHz, $CDCl_3$) δ 7.76 – 7.68 (m, 4H), 7.49 – 7.36 (m, 6H), 6.14 (d, J = 3.1 Hz, 1H), 6.00 – 5.97 (m, 6H), 4.32 – 4.24 (m, 2H), 3.78 (q, J = 6.3 Hz, 1H), 2.76 (d, J = 4.6 Hz, 1H), 2.42 (d, J = 4.6 Hz, 1H), 2.30 (s, 3H), 2.29 – 2.17 (m, 2H), 2.03 (s, 3H), 2.00 (br, 1H), 1.63 – 1.46 (m, 2H), 1.08 (d, J = 5.2 Hz, 1H), 1.07 (s, 9H) ppm; ^{13}C NMR (101 MHz, $CDCl_3$) δ 152.7, 150.7, 135.8, 133.8, 132.6, 129.8, 127.8, 125.8, 110.3, 106.9, 65.9, 64.8, 62.3, 48.2, 31.3, 27.0, 25.0, 19.5, 18.5, 18.5,13.8 ppm.

HRMS-ESI (*m/z*) $[M+Na]^+$: Calcd for $C_{31}H_{40}NaO_4Si$, 527.2588; found, 527.2584.

Preparation of ketone 17: [77]

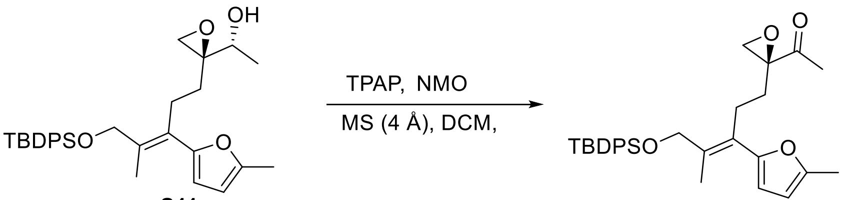

S11 **17**

To a mixture of **S11** (4.28 g, 8.48 mmol) and molecular sieves (4 Å, 17.0 g) in DCM (50 mL), NMO (1.99 g, 17.0 mmol) then TPAP (298 mg, 0.85 mmol) were added sequentially. The reaction progress was monitored by TLC. After 2 h, TLC showed that the reaction was complete. The reaction mixture was filtered through a celite pad, and the pad was washed with DCM. The combined filtrates were concentrated, and the residue was purified by flash column chromatography (EtOAc/Hexane: 1/50) to give a **17** as a colorless oil (3.82 g, 90% yield).

R_f: 0.25 (5% EtOAc in Hexane), $[\alpha]_D^{25}$ = -18.5 (1.57, DCM).

^{1}H NMR (400 MHz, $CDCl_3$) δ 7.73 (ddt, J = 5.9, 3.8, 1.6 Hz, 4H), 7.41 (qd, J = 7.1, 1.8 Hz, 6H), 6.20 (d, J = 3.1 Hz, 1H), 5.99 (d, J = 2.5 Hz, 1H), 4.42 – 4.24 (m, 2H), 2.81 (d, J = 5.0 Hz, 1H), 2.66 (d, J = 5.0 Hz, 1H), 2.43 – 2.23 (m, 5H), 2.04 (s, 3H), 2.03 – 1.96 (m, 1H), 1.94 (s, 3H), 1.61 – 1.52 (m, 1H), 1.09 (s, 9H) ppm; ^{13}C NMR (101 MHz, $CDCl_3$) δ 207.2, 152.8, 150.7, 135.8, 133.9, 132.7, 129.7, 127.8, 125.6, 110.3, 106.9, 64.8, 62.2, 50.8, 30.5, 27.0, 25.8, 23.8, 19.5, 18.4, 13.8 ppm.

HRMS-ESI (m/z) $[M+Na]^+$: Calcd for $C_{31}H_{38}NaO_4Si$, 525.2432; found, 525.2433.

Preparation of the Enolsilane 17' and its intramolecular (4+3) cycloaddition:

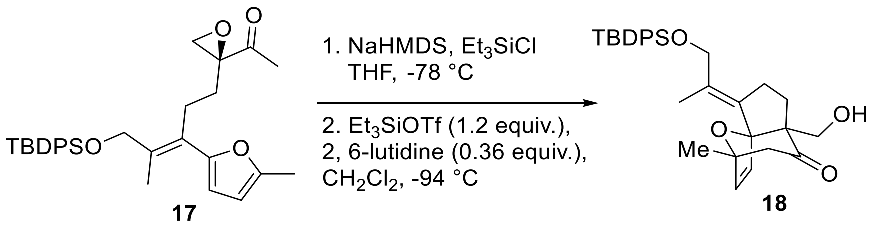

According to the general procedure for the reaction of **11**, the reaction of **17** (0.470 g, 0.930 mmol), NaHMDS (2.05 mL, 1.0 M in THF, 2.05 mmol), and TESCl (0.312 mL, 1.86 mmol) in anhydrous THF (3 mL) was followed by workup. The crude product was purified by passing through a short silica gel plug, eluted with EtOAc/ Et_3N/ hexane (0.5/ 0.5/ 99) and concentrated to give enolsilane **17'**, which was used in following reaction without further purification.

To a solution of enolsilane **17'** (0.283 g, 0.466 mmol) in DCM (10.0 mL) at -94 °C was added a solution of TESOTf (0.20 M, 0.559 mmol) and 2,6-lutidine (0.030 M, 0.0839 mmol) in DCM (2.80 mL). After 1.5 hours, $Et_3N\cdot 3HF$ (0.1 mL) was added, and the mixture was allowed to warm to room temperature over 2 hours. Saturated $NaHCO_3$ (aq., 10 mL) was added to the reaction until the resultant mixture stopped bubbling. The mixture was extracted with EtOAc three times, and the combined organics were dried over anhydrous $MgSO_4$. After concentration by rotary evaporation, the residue was purified by silica gel flash chromatography (EtOAc/ hexane: 20/ 1 to 10/ 1) to afford **18**, as a white solid (0.160 g,

68% yield). The absolute stereochemical structure of **18** was determined by 2D NMR, NOESY, and X-ray diffraction analysis.

R_f: 0.16 (10% EtOAc in Hexane), $[\alpha]_D^{25}$ = -60.0 (1.10, DCM).

^{1}H NMR (600 MHz, $CDCl_3$) δ 7.69 – 7.65 (m, 4H), 7.47 – 7.41 (m, 2H), 7.41 – 7.37 (m, 4H), 6.21 (d, *J* = 5.9 Hz, 1H), 5.99 (d, *J* = 5.8 Hz, 1H), 4.21 (d, *J* = 12.8 Hz, 1H), 4.15 (d, *J* = 12.7, 1H), 3.45 (dd, *J* = 11.5, 2.8, 1H), 3.17 (dd, *J* = 11.6, 10.5 Hz, 1H), 2.94 (dd, *J* = 10.5, 2.7 Hz, 1H), 2.52 (d, *J* = 17.7 Hz, 1H), 2.33 (d, *J* = 17.7 Hz, 1H), 2.27 – 2.15 (m, 2H), 2.15 – 2.09 (m, 1H), 1.90 (s, 3H), 1.89 – 1.83 (m, 1H), 1.49 (s, 3H), 1.06 (s, 9H) ppm; ^{13}C NMR (151 MHz, $CDCl_3$) δ 215.9, 137.5, 135.7, 135.7, 135.1, 135.0, 133.7, 133.6, 131.4, 129.9, 129.8, 127.8, 94.1, 82.6, 65.6, 64.4, 62.1, 47.8, 27.0, 26.9, 26.8, 24.0, 19.4, 15.4 ppm.

HRMS-ESI (*m/z*) $[M+Na]^+$: Calcd for $C_{31}H_{38}NaO_4Si$, 525.2432; found, 525.2428.

3.3. Preparation of substrates for intramolecular (4+3) cycloaddition-3

Preparation of allylic alcohol S14: [98, 100]

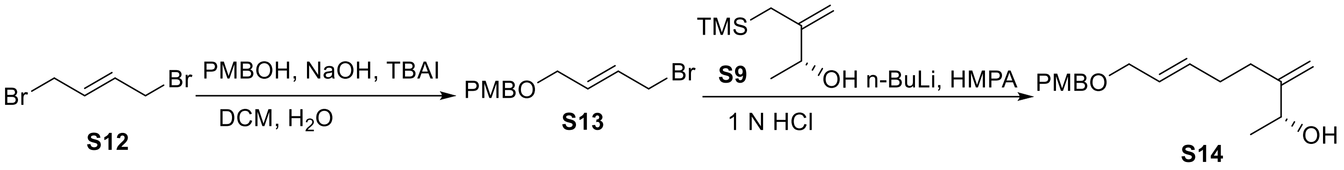

To a mixture of PMBOH (5.52 g, 40 mmol), water (20 mL) and DCM (160 mL) was added NaOH (16.0 g, 400 mmol) and TBAI (1.48 g, 4.00 mmol) sequentially. After 10 minutes, **S12** (17.1 g, 80 mmol) was added. After stirring overnight, TLC showed that the reaction was complete. Et_2O (250 mL) was added and the reaction mixture was separated by a separatory funnel. The organic layer was washed with water and brine, and dried over anhydrous $MgSO_4$. After concentrating by rotary evaporation, the residue was purified by passing through a short plug of silica gel, and eluting with 2% EtOAc in hexane. The filtrate was concentrated to give **S13** as a crude product (7.47 g), which was used in the following step without further purification.

To **S9** (1.84 mL, 10 mmol) in THF (50 mL) at -78 °C was added *n*-BuLi (4.50 mL, 2.20 M in hexane, 10 mmol). After 30 minutes, crude **S13** (5.42 g), HMPA (8.70 mL, 50 mmol) in THF (10 mL) was added. After the addition was finished, the acetone/ dry ice bath was replaced by an ice/ water bath. The reaction mixture was allowed to warm to rt and stirred overnight. The reaction was quenched with 1 N HCl, and extracted with Et_2O (3 x 40 mL). The combined organic phases were washed with water, saturated $NaHCO_3$ (aq.), brine, then dried over anhydrous $MgSO_4$. The volatiles were removed under reduced pressure, and the residue was purified by flash column chromatography (EtOAc/Hexane: 1/10 to 1/5) to give **S14** as a colorless oil (2.18 g, 79% yield).

R_f: 0.31 (20% EtOAc in Hexane), $[\alpha]_D^{25}$ = +7.25 (0.80, DCM).

^{1}H NMR (500 MHz, $CDCl_3$) δ 7.32 – 7.22 (m, 2H), 6.94 – 6.84 (m, 2H), 5.79 – 5.68 (m, 1H), 5.62 (dddd, *J* = 12.3, 7.4, 6.0, 1.2 Hz, 1H), 5.06 (d, *J* = 0.8 Hz, 1H), 4.82 (s, 1H), 4.43 (s, 2H), 4.24 (dd, *J* =

12.4, 6.1 Hz, 1H), 3.99 – 3.90 (m, 2H), 3.89 – 3.74 (m, 3H), 2.25 (dd, *J* = 14.2, 6.9 Hz, 2H), 2.13 (ddd, *J* = 29.4, 15.0, 7.7 Hz, 2H), 1.28 (d, *J* = 6.5 Hz, 3H) ppm; ^{13}C NMR (126 MHz, $CDCl_3$) *δ* 159.3, 152.7, 134.1, 130.6, 129.5, 127.0, 113.9, 108.8, 77.4, 77.2, 76.9, 71.7, 71.0, 70.7, 55.4, 31.1, 30.9, 22.3 ppm.

HRMS-ESI (*m*/*z*) $[M+Na]^+$: Calcd for $C_{17}H_{24}NaO_3$, 299.1618; found, 299.1619

Preparation of silyl ether S15: [101]

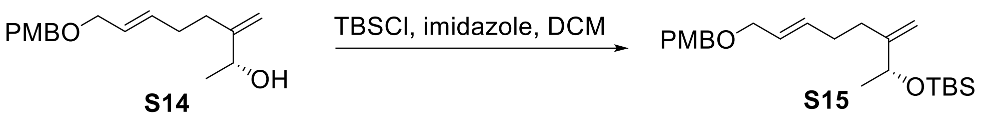

To **S14** (2.40 g, 8.70 mmol) and imidazole (1.18 g, 17.4 mmol) in DCM (20 mL) was added TBSCl (1.97 g, 13.1 mmol). After 4 h, the TLC showed that the reaction was complete. The reaction was quenched by adding saturated NH_4Cl (aq). The mixture was extracted with Et_2O (3 x 20 mL). The combined organic phases were washed with brine, and dried over anhydrous $MgSO_4$. The volatiles were removed under reduced pressure, and the residue was purified by flash column chromatography (EtOAc/Hexane: 1/100) to give **S15** as a colorless oil (3.40 g, quantitative yield).

R_f: 0.37 (5% EtOAc in Hexane), $[\alpha]_D^{25}$ = +8.50 (0.80, DCM).

^1H NMR (500 MHz, $CDCl_3$) *δ* 7.33 – 7.19 (m, 2H), 6.95 – 6.77 (m, 2H), 5.79 – 5.69 (m, 1H), 5.66 – 5.55 (m, 1H), 5.01 (s, 1H), 4.74 (s, 1H), 4.43 (s, 2H), 4.22 (q, *J* = 6.3 Hz, 1H), 4.07 – 3.90 (m, 2H), 3.80 (s, 3H), 2.23 (q, *J* = 7.2 Hz, 2H), 2.20 – 2.13 (m, 1H), 2.11 – 2.02 (m, 1H), 1.21 (d, *J* = 6.4 Hz, 3H), 0.89 (s, 9H), 0.04 (s, 3H), 0.03 (s, 3H) ppm; ^{13}C NMR (126 MHz, $CDCl_3$) δ 159.3, 152.7, 134.4, 130.7, 129.5, 126.7, 113.9, 108.3, 72.0, 71.7, 70.7, 55.4, 30.9, 30.7, 26.0, 23.7, 18.4, -4.7, -4.8 ppm.

HRMS-ESI (*m*/*z*) $[M+Na]^+$: Calcd for $C_{23}H_{38}NaO_3Si$, 413.2482; found, 413.2482.

Preparation of allylic alcohol S16: [102]

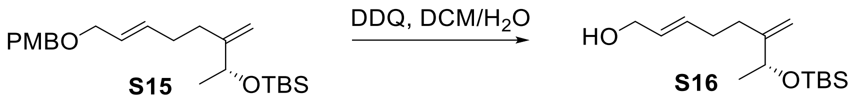

To a mixture of **S15** (3.40 g, 8.70 mmol), water (4.4 mL) and DCM (44 mL) at 0 °C was added DDQ (2.77 g, 12.6 mmol). After 5 h, TLC showed that the reaction was complete. Saturated $NaHCO_3$ (aq., 20 mL) was added, then the mixture was filtered through celite. The filtrate was separated using a separatory funnel, and the aqueous layer was back-extracted with Et_2O (4 x 20 mL). The combined organic phases were washed with saturated NH_4Cl (aq.), saturated $NaHCO_3$(aq.), and brine. The volatiles were removed under reduced pressure and the residue was dissolved in MeOH (10 mL). Saturated $NaHSO_3$ (20 mL) was added, and the reaction mixture was shaken for 1 min. H_2O (30 mL) was then added, then the mixture was extracted with hexane/EtOAc (10/ 1) three times (3 x 30 mL). The combined organic phases were washed with brine then dried over anhydrous $MgSO_4$. The volatiles were removed under reduced pressure, and the residue was purified by flash column chromatography (EtOAc/Hexane: 1/10) to give **S16** as a colorless oil (1.90 g, 81% yield).

R_f: 0.32 (10% EtOAc in Hexane), $[\alpha]_D^{25}$ = +6.81 (1.057, DCM).

^{1}H NMR (500 MHz, $CDCl_3$) δ 5.78 – 5.62 (m, 2H), 5.01 (s, 1H), 4.73 (d, J = 0.90 Hz, 1H), 4.22 (q, J = 6.3 Hz, 1H), 4.09 (t, J = 5.4 Hz, 2H), 2.22 (q, J = 6.7 Hz, 2H), 2.19 – 2.11 (m, 1H), 2.10 – 2.02 (m, 1H), 1.32 (t, J = 5.7 Hz, 1H), 1.21 (d, J = 6.4 Hz, 3H), 0.89 (s, 9H), 0.04 (s, 3H), 0.02 (s, 3H) ppm;. ^{13}C NMR (126 MHz, $CDCl_3$) δ 152.6, 133.1, 129.3, 108.3, 72.0, 63.9, 30.8, 30.6, 26.0, 23.7, 18.4, -4.7, -4.8 ppm.

HRMS-ESI (*m/z*) $[M+H]^+$: Calcd for $C_{15}H_{31}O_2Si$, 271.2088; found, 271.2086

Preparation of epoxide S17: [103]

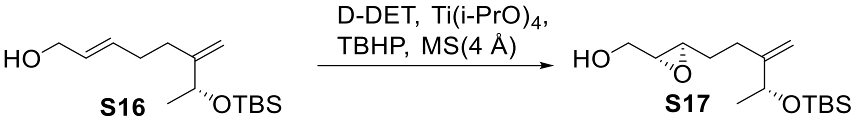

To a mixture of D-(-)-DET (0.699 mL, 4.09 mmol, molecular sieves (4 Å, 1.84 g) and DCM (68 mL). at -35 °C was added Ti(*i*-PrO)$_4$ (0.997 mL, 3.41 mmol). After 30 minutes, a solution of **S16** (1.84 g, 6.81 mmol) in DCM (10 mL) was added. After 1 h, TBHP (2.70 mL, 5.5 M in decane, 15.0 mmol) was added dropwise. The reaction mixture was kept at the same temperature overnight. The reaction progress was monitored by TLC. A mixture of ferrous sulfate, tartaric acid and water (24 g/ 24 g/ 400 g, 50 mL) was added, then the mixture was allowed to warm to rt and stirred for 1 h.

The mixture was filtered through celite, and the filtrate was extracted with DCM (3 x 30 mL). The combined organic layers were concentrated, and the residue was dissolved in Et_2O. After cooling to 0 °C, NaOH in brine (10 wt%, 100 mL) was added and the reaction was stirred for 1 h. The mixture was separated using a separatory funnel, and the aqueous layer was back-extracted with Et_2O (3 x 80 mL). The The combined organic extracts were washed with brine then dried over anhydrous $MgSO_4$. The volatiles were removed under reduced pressure, and the residue was purified by flash column chromatography (EtOAc/Hexane: 1/10) to give **S17** as a colorless oil (1.76 g, 90% yield).

R_f: 0.30 (20% EtOAc in Hexane), $[\alpha]_D^{25}$ = +26.4 (1.68, DCM).

^{1}H NMR (500 MHz, $CDCl_3$) δ 5.01 (s, 1H), 4.73 (d, *J* = 0.95 Hz, 1H), 4.23 (q, *J* = 6.3 Hz, 1H), 3.90 (ddd, *J* = 12.6, 5.1, 2.4 Hz, 1H), 3.59 (ddd, *J* = 12.2, 6.5, 4.6 Hz, 1H), 3.03 – 2.97 (m, 1H), 2.96 – 2.92 (m, 1H), 2.27 – 2.07 (m, 1H), 1.82 – 1.66 (m, 2H), 1.21 (d, *J* = 6.4 Hz, 3H), 0.87 (s, 9H), 0.03 (s, 3H), 0.01 (s, 3H) ppm; ^{13}C NMR (126 MHz, $CDCl_3$) δ 152.2, 108.5, 72.0, 61.8, 58.8, 55.9, 30.2, 27.2, 26.0, 23.7, 18.3, -4.7, -4.8 ppm.

HRMS-ESI (*m/z*) [M+Na]$^+$: Calcd for $C_{15}H_{30}NaO_3Si$, 309.1856; found, 309.1857.

Preparation of diol S18: [104]

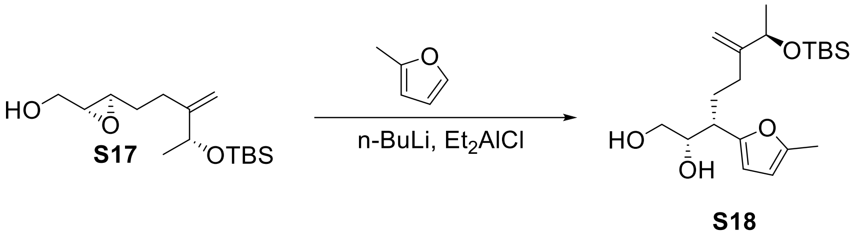

To 2-methylfuran (3.18 mL, 32.1 mmol) in Et_2O (16 mL) at -78 °C, was added n-BuLi (9.96 mL, 2.3 M in hexane, 22.9 mmol). The reaction mixture was allowed to warm to rt over 5 h. The reaction was cooled to 0 °C, then Et_2AlCl (10.3 mL, 2.0 M in hexane, 20.6 mmol) was added dropwise. After 30 minutes, **S17** (1.31 g, 4.58 mmol) in Et_2O (4 mL) was added, then the reaction mixture was allowed to warm to rt and stirred overnight. The reaction was quenched by the careful addition of water (1.0 mL), then sat. potassium sodium tartrate (15 mL). The resulting mixture was stirred at rt for 2 h, then extracted with EtOAc (3 x 15 mL). The The combined organic extracts were washed with brine, and dried over anhydrous $MgSO_4$. The volatiles were removed under reduced pressure, and the residue was purified by flash column chromatography (EtOAc/Hexane: 15%) to give **S18** as a colorless oil (1.34 g, 79% yield), along with recovered unreacted **S17** (0.110 g). This is an 87% BRSM yield for **S18**.

R_f: 0.38 (30% EtOAc in Hexane), $[\alpha]_D^{25}$ = -5.33 (0.938, DCM).

^{1}H NMR (500 MHz, $CDCl_3$) δ 5.95 (d, J = 3.0 Hz, 1H), 5.89 – 5.83 (m, 1H), 4.99 (s, 1H), 4.73 (s, 1H), 4.18 (q, J = 6.3 Hz, 1H), 3.79 (td, J = 7.4, 3.1 Hz, 1H), 3.54 (dd, J = 11.4, 3.0 Hz, 1H), 3.40 (dd, J = 11.4, 7.3 Hz, 1H), 2.85 – 2.72 (m, 1H), 2.24 (s, 3H), 2.05 – 1.97 (m, 2H), 1.90 – 1.76 (m, 2H), 1.16 (d, J = 6.4 Hz, 3H), 0.86 (s, 9H), 0.02 (s, 3H), -0.01 (s, 3H) ppm; ^{13}C NMR (126 MHz, $CDCl_3$) δ 153.0, 152.8, 151.2, 108.1, 108.0, 106.1, 74.6, 72.3, 65.1, 42.8, 28.6, 28.2, 26.0, 23.7, 18.4, 13.7, -4.7, -4.8 ppm.

HRMS-ESI (*m/z*) $[M+Na]^+$: Calcd for $C_{20}H_{36}NaO_4Si$, 391.2275; found, 391.2273.

Preparation of alcohol S19: [105]

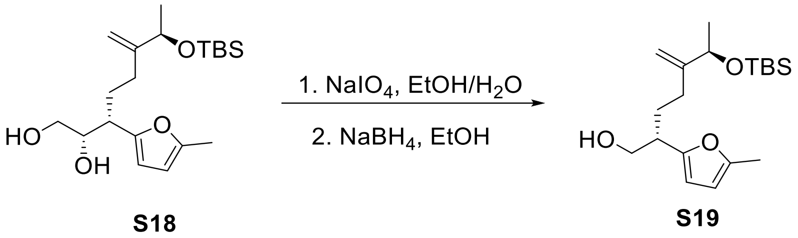

To a solution of **S18** (1.13 g, 3.07 mmol) in water (20 mL) and EtOH (10 mL) at 0 °C was added $NaIO_4$ (1.31 g, 6.14 mmol). The cold bath was removed, and the reaction mixture was stirred at rt for 40 minutes. The reaction progress was monitored by TLC. Water (20 mL) was added to the reaction, and the resulting mixture was extracted with EtOAc (3 x 40 mL). The combined organic phases were washed with brine, then dried over anhydrous $MgSO_4$. The volatiles were removed under reduced pressure.

The residue was dissolved in EtOH (15 mL), then $NaBH_4$ (233 mg, 6.14 mmol) was added. After 30 minutes, the TLC showed that the reaction was complete. The reaction was quenched by the careful addition of saturated NH_4Cl (aq., 15 mL). The resulting mixture was extracted with EtOAc (3 x 20 mL). The combined organic phases were washed with brine, and dried over anhydrous $MgSO_4$. The volatiles were removed under reduced pressure. The residue was purified by flash column chromatography (EtOAc/Hexane: 7%) to give **S19** as a colorless oil (0.805 g, 76% yield).

R_f: 0.35 (10% EtOAc in Hexane), $[\alpha]_D^{25}$ = -4.23 (1.14, DCM).

^{1}H NMR (500 MHz, $CDCl_3$) δ 5.99 (d, J = 2.9 Hz, 1H), 5.93 – 5.82 (m, 1H), 5.00 (s, 1H), 4.77 – 4.68 (m, 1H), 4.21 (q, J = 6.3 Hz, 1H), 3.73 (d, J = 5.2 Hz, 2H), 2.86 (p, J = 6.5 Hz, 1H), 2.25 (s, 3H), 2.06 (dt, J = 15.7, 7.8 Hz, 1H), 1.95 (dt, J = 15.7, 7.4 Hz, 1H), 1.83 – 1.75 (m, 2H), 1.18 (d, J = 6.4 Hz, 3H), 0.87 (s, 9H), 0.03 (s, 3H), 0.00 (s, 3H) ppm; ^{13}C NMR (126 MHz, $CDCl_3$) δ 154.1, 152.9, 151.2, 108.2, 107.4, 106.0, 72.2, 65.3, 42.0, 28.5, 28.4, 26.0, 23.7, 18.4, 13.7, -4.7, -4.8 ppm.

HRMS-ESI (m/z) $[M+Na]^+$: Calcd for $C_{19}H_{34}NaO_3Si$, 361.2169; found, 361.2170.

Preparation of ether S20: [106]

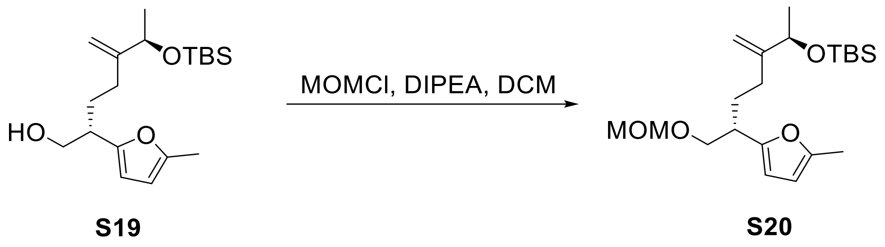

To **S19** (0.766 mg, 2.27 mmol) in DCM (7.0 mL) was added DIPEA (3.85 mL, 22.7 mmol) and MOMCl (0.862 mL, 11.4 mmol). After 40 minutes, TLC showed that the reaction was complete. The reaction was quenched by the addition of saturated NH_4Cl (aq., 10 mL). The resulting mixture was extracted with Et_2O (3 x 15 mL). The combined organic extracts were washed with saturated $NaHCO_3$ (aq.), brine, then dried over anhydrous $MgSO_4$. The volatiles were removed under reduced pressure. The residue was purified by flash column chromatography (EtOAc/Hexane: 1%) to give **S20** as a colorless oil (0.777 g, 90% yield).

R_f: 0.71 (10% EtOAc in Hexane), $[\alpha]_D^{25}$ = -1.51 (0.931, DCM).

^{1}H NMR (500 MHz, $CDCl_3$) δ 5.94 (d, J = 3.0 Hz, 1H), 5.89 – 5.80 (m, 1H), 5.00 (s, 1H), 4.80 – 4.71 (m, 1H), 4.64 – 4.54 (m, 2H), 4.20 (q, J = 6.3 Hz, 1H), 3.73 (dd, J = 9.5, 6.6 Hz, 1H), 3.64 (dd, J = 9.6, 6.6 Hz, 1H), 3.31 (s, 3H), 3.00 – 2.87 (m, 1H), 2.24 (d, J = 0.8 Hz, 3H), 2.12 – 2.02 (m, 1H), 1.99 – 1.85 (m, 2H), 1.81 – 1.71 (m, 1H), 1.18 (d, J = 6.4 Hz, 3H), 0.87 (s, 9H), 0.03 (s, 3H), 0.00 (s, 3H) ppm; ^{13}C NMR (126 MHz, $CDCl_3$) δ 154.5, 153.1, 150.6, 108.0, 106.5, 105.9, 96.6, 72.1, 70.1, 55.2, 39.6, 29.2, 28.5, 26.0, 23.7, 18.4, 13.7, -4.7, -4.8 ppm.

HRMS-ESI (*m/z*) $[M+Na]^+$: Calcd for $C_{21}H_{38}NaO_4Si$, 405.2432; found, 405.2435.

Preparation of allylic alcohol S21: [107]

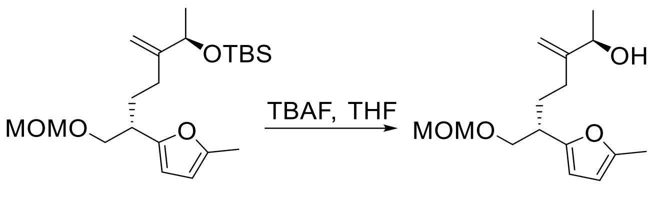

S20 **S21**

To a solution of **S20** (0.722 g, 1.89 mmol) in THF (6 mL) was added TBAF (3.78 mL, 1 M in THF, 3.78 mmol). The mixture was stirred at rt overnight. The volatiles were removed under reduced pressure, and the residue was purified by flash column chromatography (EtOAc/Hexane: 15%) to give **S21** as a colorless oil (0.487 g, 96% yield).

R_f: 0.24 (15% EtOAc in Hexane), $[\alpha]_D^{25}$ = -4.26 (0.891, DCM).

^{1}H NMR (400 MHz, $CDCl_3$) δ 5.95 (d, J = 3.0 Hz, 1H), 5.85 (dd, J = 3.0, 1.2 Hz, 1H), 5.05 (s, 1H), 4.82 (s, 1H), 4.62 – 4.57 (m, 2H), 4.23 (q, J = 6.5 Hz, 1H), 3.73 (dd, J = 9.6, 6.5 Hz, 1H), 3.64 (dd, J = 9.6, 6.8 Hz, 1H), 3.31 (s, 3H), 2.99 – 2.91 (m, 1H), 2.25 (s, 3H), 2.16 – 2.07 (m, 1H), 2.05 – 1.97 (m, 1H), 1.97 – 1.85 (m, 1H), 1.84 – 1.74 (m, 1H), 1.26 (d, J = 6.3 Hz, 3H) ppm; ^{13}C NMR (101 MHz, $CDCl_3$) δ 154.3, 153.0, 150.7, 108.7, 106.5, 106.0, 96.6, 71.2, 70.1, 55.3, 39.4, 29.2, 29.2, 22.3, 13.7 ppm.

HRMS-ESI (m/z) $[M+Na]^+$: Calcd for $C_{15}H_{24}NaO_4$, 291.1568; found, 291.1565.

Preparation of epoxide S22: [76]

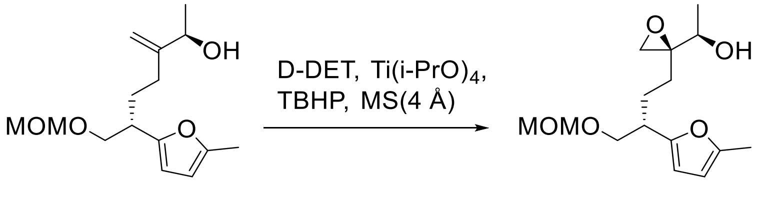

S21 **S22**

To a mixture of D-(−)-DET (0.173 mL, 1.01 mmol, molecular sieves (4 Å, 0.454 g) in DCM (8.0 mL) at -35 °C, was added Ti(*i*-PrO)$_4$ (0.247 mL, 0.850 mmol). After 30 minutes, a solution of **S21** (0.454 g, 1.69 mmol) in DCM (10 mL) was added. After 1 h, TBHP (0.680 mL, 5.5 M in decane, 3.72 mmol) was added dropwise. The reaction mixture was kept at this temperature for 3 h. The reaction progress was monitored by TLC.

A mixture of ferrous sulfate, tartaric acid and water (24 g/ 24 g/ 400 g, 10 mL) was added, and the mixture was allowed to warm to rt and stirred for 1 h. The reaction was filtered through celite, and the filtrate was extracted with DCM (3 x 30 mL). The combined organic layers were concentrated.

The residue was dissolved in DCM, and cooled to 0 °C. NaOH in brine (10 wt%, 100 mL) was added and stirred for 1 h. The mixture was separated using a separatory funnel, and the aqueous layer was back-extracted with DCM (3 x 80 mL). The combined organic extracts were washed with brine, and dried over anhydrous $MgSO_4$. The volatiles were removed under reduced pressure, and the residue was purified by flash column chromatography (EtOAc/Hexane: 20%) to give **S22** as a colorless oil (0.428 g, 88% yield).

R_f: 0.22 (20% EtOAc in Hexane), $[\alpha]_D^{25}$ = -13.8 (0.667, DCM).

^{1}H NMR (500 MHz, $CDCl_3$) δ 5.93 (d, *J* = 3.0 Hz, 1H), 5.88 – 5.82 (m, 1H), 4.58 (s, 2H), 3.89 (q, *J* = 6.2 Hz, 1H), 3.70 (dd, *J* = 9.6, 6.4 Hz, 1H), 3.60 (dd, *J* = 9.6, 6.9 Hz, 1H), 3.30 (s, 3H), 2.88 (ddd, *J* = 10.5, 6.4, 3.1 Hz, 1H), 2.85 (d, *J* = 4.6 Hz, 1H), 2.61 (d, *J* = 4.6 Hz, 1H), 2.26 – 2.21 (m, 3H), 2.11 (d, *J* = 5.1 Hz, 1H), 1.83 – 1.73 (m, 1H), 1.69 – 1.58 (m, 3H), 1.19 (d, *J* = 6.4 Hz, 3H) ppm; ^{13}C NMR (126 MHz, $CDCl_3$) δ 153.8, 150.8, 106.7, 106.0, 96.6, 69.9, 66.3, 62.4, 55.3, 48.3, 39.7, 28.6, 25.1, 18.7, 13.7 ppm.

HRMS-ESI (*m/z*) $[M+Na]^+$: Calcd for $C_{15}H_{24}NaO_5$, 307.1516; found, 307.1516.

Preparation of epoxy ketone 19: [77]

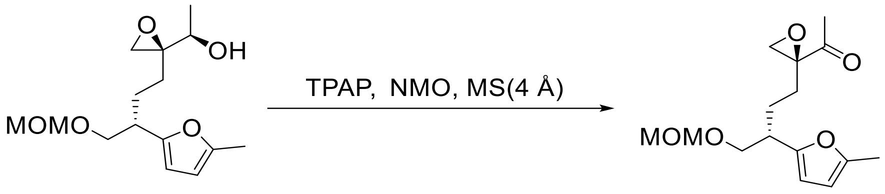

S22 **19**

To a mixture of **S22** (0.400 g, 1.41 mmol), molecular sieves (4 Å, 2.82 g) and DCM (14 mL), was added NMO (0.330 g, 2.82 mmol) then TPAP (49.5 mg, 0.141 mmol). After 3 h, the TLC showed that the reaction was complete. The mixture was filtered through celite, and rinsed with DCM. The filtrate was concentrated, and the residue was purified by flash column chromatography (EtOAc/Hexane: 1/20) to give **19** as a colorless oil (0.376 g, 95% yield).

R_f: 0.44 (20% EtOAc in Hexane), $[\alpha]_D^{25}$ = -47.4 (0.832, DCM).

^{1}H NMR (300 MHz, $CDCl_3$) δ 5.95 (d, J = 3.0 Hz, 1H), 5.90 – 5.78 (m, 1H), 4.57 (s, 2H), 3.69 (dd, J = 9.6, 6.4 Hz, 1H), 3.59 (dd, J = 9.6, 6.7 Hz, 1H), 3.29 (s, 3H), 2.98 – 2.80 (m, 3H), 2.23 (d, J = 0.9 Hz, 3H), 2.16 – 2.05 (m, 1H), 2.01 (s, 3H), 1.81 – 1.67 (m, 2H), 1.62 – 1.50 (m, 1H) ppm; ^{13}C NMR (75 MHz, $CDCl_3$) δ 207.7, 153.8, 150.8, 106.7, 106.0, 96.6, 69.9, 62.6, 55.3, 50.9, 39.6, 27.9, 25.7, 23.9, 13.7 ppm.

HRMS-ESI (m/z) $[M+Na]^+$: Calcd for $C_{15}H_{22}NaO_5$, 305.1359; found, 305.1360.

Preparation of 19′ (enolsilane derivative of 19) and its intramolecular (4+3) cycloaddition:

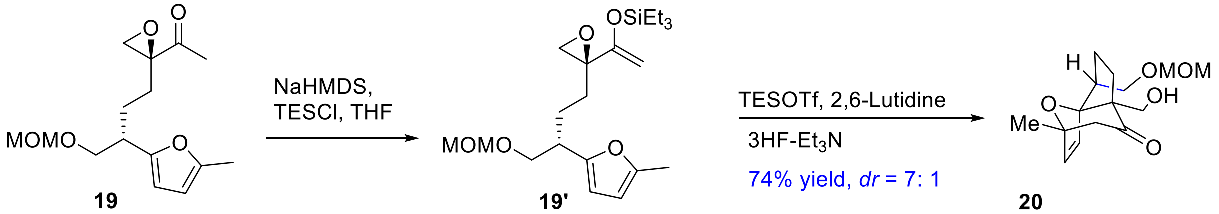

19 **19'** **20**

According to the general procedure for the reaction of **11**, the reaction of **19** (328 mg, 1.16 mmol), NaHMDS (1.16 mL, 2.0 M in THF, 2.32 mmol), and Et_3SiCl (0.487 mL, 2.90 mmol) in anhydrous THF (6 mL) was followed by workup. The reaction mixture was passed through a short silica gel plug, rinsed (EA/ Et_3N/ hexane: 0.5/ 0.5/ 99) to give crude **19'** (492 mg, around 85% purity), which was used in the following step without further purification.

To crude **19'** (44.5 mg, 0.11 mmol) in DCM (2.2 mL) at -94 °C was added a solution of TESOTf (0.20 M, 0.022 mmol), and 2,6-lutidine (0.020 M, 0.0022 mmol) in DCM (0.11 mL). After 40 minutes, $Et_3N{\cdot}3HF$ (0.1 mL) was added, and the mixture was allowed to warm to room temperature over 2 hours. Then saturated $NaHCO_3$ (aq., 1 mL) was added to the reaction until the reaction mixture stopped bubbling. The mixture was extracted with EtOAc three times, and the combined organic layers were dried over anhydrous $MgSO_4$, and concentrated by rotary evaporation. The residue was purified by flash chromatography on silica gel (25% to 30% EtOAc in hexanes) to afford **20** and its endo- isomer **20′** (22.8 mg, 74% yield, dr= 7:1) as a colorless oil.

R_f: 0.45 (40% EtOAc in hexane), $[\alpha]_D^{25}$ = -64.7 (0.985, DCM).

^{1}H NMR (600 MHz, $CDCl_3$) δ 6.15 (d, J = 5.9 Hz, 1H, major), 6.12 (d, J = 5.8 Hz, 1H, minor), 6.07 (d, J = 5.9 Hz, 1H, major), 5.98 (d, J = 5.8 Hz, 1H, minor),.4.64 (q, J = 6.6 Hz, 2H, major), 4.58 (d, J = 1.1 Hz, 2H, minor), 3.75 (dd, J = 9.6, 7.1 Hz, 1H, minor), 3.67 – 3.59 (m, 3H, major), 3.58 – 3.48 (m, 3H, minor), 3.45 (d, J = 11.6 Hz, 1H, major), 3.37 (s, 3H, major), 3.34 (s, 3H, minor), 2.93 (d, J = 10.0 Hz, 1H, major& minor), 2.63 – 2.56 (m, 1H, minor), 2.53 (d, J = 17.5 Hz, 1H, minor), 2.52 – 2.47 (m, 1H, major), 2.45 (d, J = 18.3 Hz, 1H, major), 2.33 (d, J = 18.3 Hz, 1H, major), 2.33 (d, J = 17.4 Hz, 2H, minor) 2.22 (dd, J = 12.2, 7.0 Hz, 1H, major), 2.14 – 2.03 (m, 2H, minor). 2.03 – 1.95 (m, 1H, major& minor), 1.88 (tdd, J = 12.3, 7.8, 1.0 Hz, 1H, major), 1.75 (tdd, J = 12.6, 9.0, 7.0 Hz, 1H, major& minor), 1.45 (s, 3H, major), 1.44 (s, 3H, minor) ppm; ^{13}C NMR (151 MHz, $CDCl_3$) δ 216.2 (major), 215.2 (minor), 138.12 (major), 136.92 (minor), 133.62 (minor), 132.32 (major), 96.82 (major), 96. 82 (minor), 96.02 (major), 95.52 (minor), 82.82 (minor), 82.02 (major), 68.52 (major), 67.42 (minor), 65.82 (minor), 65.42 (major),

63.72 (minor), 61.52 (major), 55.72 (major), 55.32 (minor), 48.12 (minor), 47.22 (major), 46.52 (major), 41.82 (minor), 29.12 (major), 28.02 (minor), 26.12 (minor), 25.52 (major), 24.32 (major), 24.12 (minor) ppm.

HRMS-ESI (m/z) $[M+Na]^+$: Calcd for $C_{15}H_{22}NaO_5$, 305.1359; found, 305.1354.

3.4. Preparation of substrates for intramolecular (4+3) cycloaddition-4

Synthesis of Substrate 21 from S1

Preparation of S1f

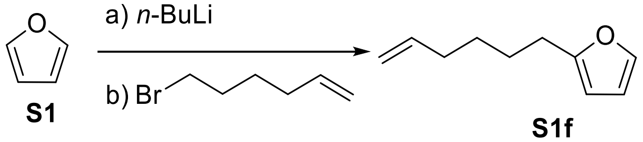

According to the general procedure for the preparation of **S1a**, furan (10.0 mL, 0.157 mol) was treated with *n*-BuLi (2.4 M, 34.0 mL, 80 mmol), and 6-bromo-1-hexene (3.26 g, 20.0 mmol) in anhydrous THF (150 mL), followed by workup and concentration. The residue was purified by flash column chromatography (eluent: hexanes) to afford **S1f** (2.73 g, 91% yield) as a colorless oil.

R_f: 0.62 (hexane);

^1H NMR (400 MHz, C6D6) δ 6.84 (dd, *J* = 5.1, 1.2 Hz, 1H), 6.76 (dd, *J* = 5.1, 3.4 Hz, 1H), 6.65 – 6.59 (m, 1H), 5.68 (ddt, *J* = 17.0, 10.1, 6.7 Hz, 1H), 5.02 – 4.91 (m, 2H), 2.58 (d, *J* = 7.5 Hz, 2H), 1.88 (t, *J* = 7.4 Hz, 2H), 1.56 – 1.46 (m, 2H), 1.29 – 1.20 (m, 2H) ppm; ^{13}C NMR (101 MHz, C_6D_6) δ 145.2, 138.5, 126.6, 124.0, 122.8, 114.4, 33.4, 31.2, 29.6, 28.2 ppm.

The spectral characteristics are consistent with those of **S1f** in the literature. [108]

Preparation of S1g

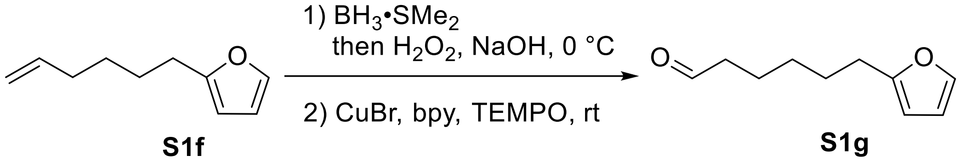

According to the general procedure for the preparation of **S1b**, treatment of **S1f** with $BH_3{\cdot}SMe_2$ (10 M, 4.0 mL, 40 mmol) in anhydrous THF (50 mL) and workup, followed by treatment with CuBr (0.290 g, 2.0 mmol), bpy (0.310 g, 2.0 mmol), and TEMPO (0.320 g, 2.0 mmol) in CH_3CN (50 mL) and workup, provided a crude material which was purified by flash column chromatography (eluent: 5% EtOAc in hexanes) to afford **S1g** (3.32 g, 89% yield over two steps) as a colorless oil.

R_f: 0.52 (10% EtOAc in hexane).

^{1}H NMR (400 MHz, C_6D_6) δ 9.27 (t, *J* = 1.7 Hz, 1H), 7.14 (d, *J* = 1.9 Hz, 1H), 6.13 (dd, *J* = 3.1, 1.9 Hz, 1H), 5.85 (d, *J* = 3.0 Hz, 1H), 2.40 (t, *J* = 7.5 Hz, 2H), 1.72 (td, *J* = 7.3, 1.7 Hz, 2H), 1.38 (p, *J* = 7.6 Hz, 2H), 1.21 (dt, *J* = 15.1, 7.4 Hz, 2H), 1.02 – 0.92 (m, 2H) ppm; ^{13}C NMR (101 MHz, C_6D_6) δ 200.3, 156.0, 140.7, 110.1, 104.8, 43.3, 28.4, 27.7, 27.63, 21.5 ppm.

The spectral characteristics are consistent with those of **S1g** in the literature. [109]

Preparation of S1h

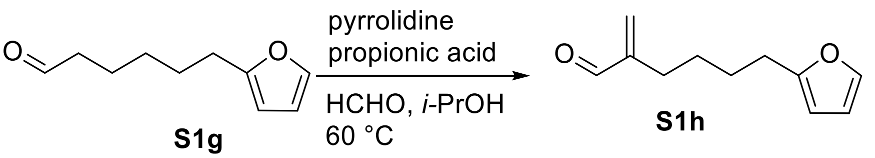

According to the general procedure for the preparation of **S1c**, treatment of **S1g** (1.11 g, 6.65 mmol) in *i*PrOH (3.0 mL) with pyrrolidine (54.0 μL, 0.67 mmol), propionic acid (50.0 μL, 0.66 mmol), and

formaldehyde (37% aq., 0.990 mL, 9.98 mmol), followed by workup provided **S1h**, which was used in the following step without further purification.

R_f: 0.57 (10% EtOAc in hexanes).

Preparation of S1i

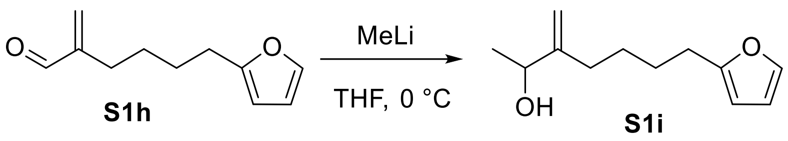

According to the general procedure for the preparation of **S1d**, treatment of crude **S1h** with methyllithium (1.0 M, 5.12 mL, 5.12 mmol) in anhydrous THF (20 mL), followed by workup, provided **S1i**, which was used in the following step without further purification.

R_f: 0.33 (20% EtOAc in hexane).

Preparation of S1j

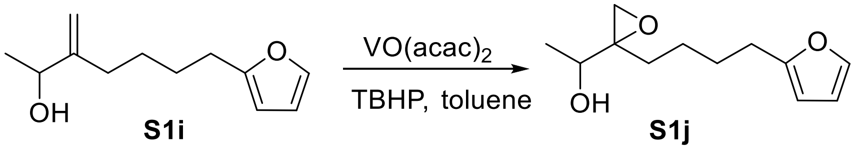

According to the general procedure for the preparation of **S1e**, treatment of crude **S1i** with $VO(acac)_2$ (93.0 mg, 0.35 mmol), and TBHP (5~6 M, 1.05 mL) in toluene (17.0 mL), followed by workup and flash chromatography provided **S1j** (0.381 g, 27% yield over 3 steps).

R_f: 0.37 (20% EtOAc in hexanes).

^{1}H NMR (400 MHz, $CDCl_3$) δ 7.28 (d, *J* = 1.8 Hz, 1H), 6.26 (dd, *J* = 3.1, 1.9 Hz, 1H), 5.96 (d, *J* = 3.1 Hz, 1H), 3.90 (q, *J* = 6.4 Hz, 1H), 2.85 (d, *J* = 4.7 Hz, 1H), 2.67 – 2.58 (m, 3H), 2.19 (dd, *J* = 6.9, 3.3 Hz, 1H), 1.79 – 1.69 (m, 1H), 1.63 (ddt, *J* = 14.4, 10.9, 7.8 Hz, 3H), 1.40 (ddd, *J* = 9.5, 8.0, 4.2 Hz, 2H), 1.21 (d, *J* = 6.3 Hz, 3H). ^{13}C NMR (101 MHz, $CDCl_3$) δ 156.0, 140.9, 110.2, 104.9, 66.0, 62.5, 48.3, 30.9, 28.3, 27.9, 23.5, 18.7.

HRMS-ESI *m/z* $[M+H]^+$ Calcd for $C_{12}H_{19}O_3$: 211.1329; Found 323.1328.

Preparation of 21

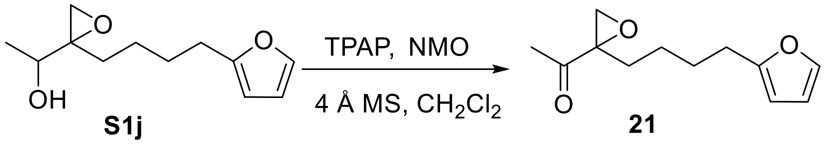

S1j **21**

According to the general procedure for preparation of **11**, treatment of **S1j** (381 mg, 1.81 mmol) with TPAP (64.0 mg, 0.18 mmol), NMO (0.637 g, 5.44 mmol), and 4Å MS (1.0 g), in CH_2Cl_2 (5 mL) followed by workup and concentration, provided a residue which was purified by flash column chromatography (eluent: 5% to 10% EtOAc in hexane) to afford **21** (0.307 g, 81% yield) as a pale yellow oil.

R_f: 0.50 (20% EtOAc in hexane).

^{1}H NMR (400 MHz, $CDCl_3$) δ 7.29 – 7.27 (m, 1H), 6.26 (dd, *J* = 3.2, 1.8 Hz, 1H), 6.00 – 5.93 (m, 1H), 2.92 (d, *J* = 4.9 Hz, 1H), 2.86 (d, *J* = 5.0 Hz, 1H), 2.61 (t, *J* = 7.5 Hz, 2H), 2.23 – 2.11 (m, 1H), 2.02 (s, 3H), 1.75 – 1.35 (m, 5H) ppm; ^{13}C NMR (101 MHz, $CDCl_3$) δ 207.8, 156.0, 140.8, 110.1, 104.8, 62.7, 50.8, 29.9, 28.0, 27.7, 24.2, 23.9 ppm.

HRMS-EI *m/z* $[M^+]$ Calcd for $C_{11}H_{14}O_3$: 208.1099; Found 208.1093.

Preparation of 21′ (enolsilane derivative of 21) and its attempted intramolecular cycloaddition:

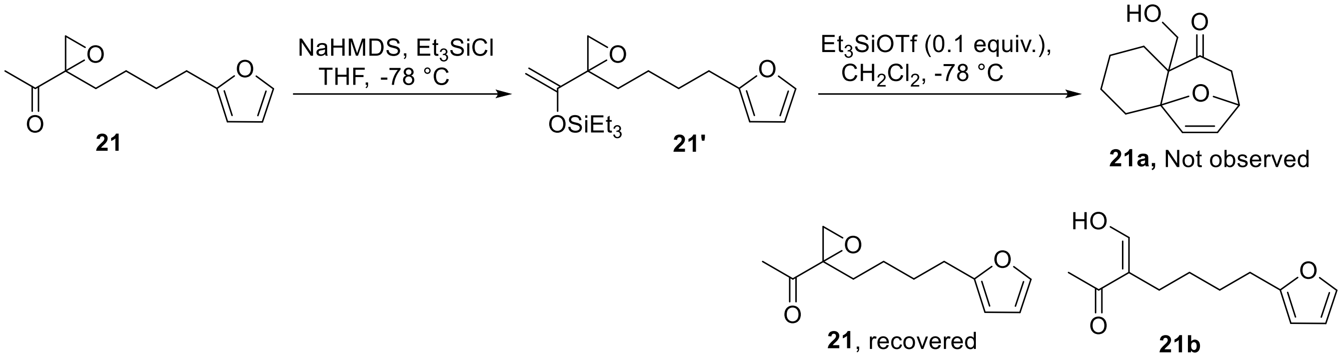

According to the general procedure for the preparation of **11′**, treatment of **21** (0.254 g, 1.22 mmol) with NaHMDS (2.0 M, 1.22 ml, 2.44 mmol) and Et_3SiCl (0.610 mL, 3.66 mmol) in anhydrous THF (15 mL) followed by workup provided crude **21′**, which was which was purified by flash column chromatography (eluent: 1% to 3% EtOAc and 1% triethylamine in hexane) to give pure **21′** (0.382 g, 97% yield) as a colorless oil.

R_f: 0.90 (10% EtOAc in hexanes).

^{1}H NMR (500 MHz, C_6D_6) δ 7.64 – 7.59 (m, 1H), 6.62 (dd, *J* = 3.0, 2.0 Hz, 1H), 6.41 – 6.34 (m, 1H), 5.07 (d, *J* = 1.2 Hz, 1H), 4.74 (d, *J* = 1.3 Hz, 1H), 3.12 (d, *J* = 6.0 Hz, 1H), 2.99 (t, *J* = 7.4 Hz, 2H), 2.85 (d, *J* = 6.0 Hz, 1H), 2.47 – 2.38 (m, 1H), 2.14 – 2.05 (m, 2H), 2.05 – 1.91 (m, 3H), 1.47 (t, *J* = 8.0 Hz, 9H), 1.15 (q, *J* = 7.6 Hz, 6H). ^{13}C NMR (126 MHz, C_6D_6) δ 157.5, 156.4, 140.9, 110.4, 105.1, 90.0, 59.0, 53.4, 32.3, 28.5, 28.1, 24.9, 6.8, 5.1.

HRMS-ESI *m/z* $[M+H]^+$ Calcd for $C_{18}H_{31}O_3Si$: 323.2042; Found 323.2037.

According to the general procedure for the (4+3) cycloadditions of **11′**, **21′** (93.3 mg, 0.289 mmol) in CH_2Cl_2 (6.0 mL) was treated with Et_3SiOTf (65 µL, 0.289 mmol) at −78 °C. After workup, 25.3 mg ethylene carbonate was added as internal standard. The resultant ^{1}H NMR of the crude product

mixture·did not show the presence of any desired cycloadduct **21a**, but a 35% yield of **21** and 44% yield of isomerized product **21b**.· Purification by flash chromatography on silica gel (10 % EtOAc in hexanes) provided recovered **21** (17.0 mg, 28% yield) as a colorless oil and isomerized **21b** (18.2 mg, 30% yield) as a colorless oil.

Compound **21b**: R_f: 0.40 (10% EtOAc in hexanes).

^{1}H NMR (500 MHz, $CDCl_3$) δ 14.96 (d, *J* = 7.0 Hz, 1H), 7.94 (d, *J* = 6.9 Hz, 1H), 7.30 (dd, *J* = 1.8, 0.9 Hz, 1H), 6.28 (dd, *J* = 3.1, 1.9 Hz, 1H), 5.98 (dd, *J* = 3.1, 1.0 Hz, 1H), 2.68 – 2.59 (m, 2H), 2.21 – 2.15 (m, 2H), 2.13 (s, 3H), 1.71 – 1.64 (m, 2H), 1.46 (t, *J* = 7.8 Hz, 2H).^{13}C NMR (126 MHz, $CDCl_3$) δ 194.9, 177.3, 155.9, 141.0, 112.8, 110.2, 105.1, 30.5, 27.8, 27.7, 27.6, 24.0.

HRMS-ESI *m/z* $[M+H]^+$ Calcd for $C_{12}H_{17}O_3$: 209.1178; Found 209.1170.

3.5. Formal total synthesis of pseudolaric acid B: Synthesis of **6**

Preparation of α,β-unsaturated amide 24:

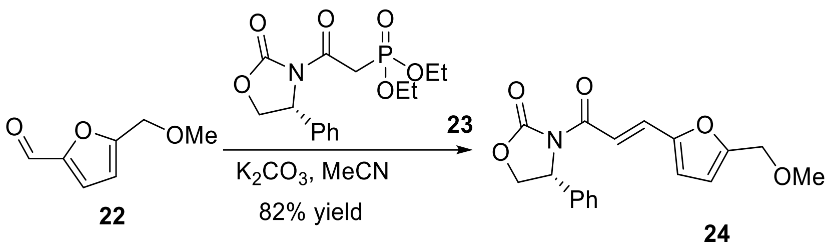

To a solution of aldehyde **22** (21.4 g, 153 mmol) and **23** (61.6 g, 181 mmol) in MeCN (600 mL) was added K_2CO_3 (63.3 g, 459 mmol). The reaction was stirred at rt for 18 h. Water (300 mL) was added to the reaction mixture. After removing most of MeCN under reduced pressure by rotoevaporation, the remaining mixture was extracted with EtOAc (3 x 150 mL). The combined organic extracts were washed with brine, and dried over anhydrous $MgSO_4$. The filtrate was concentrated in *vacuo*. The resulting oil was recrystallized from EtOAc and hexane to give a first crop (31.3 g) of **24** as a white solid. The mother liquor was concentrated and further purified by Combiflash to give a semi-purified product, which was recrystallized from EtOAc and hexane to provide another crop (9.70 g) of **24** as a white solid. A total of 41.0 g of **24** was obtained (82% yield).

mp.: 110 -111 °C. R_f: 0.44 (20% EtOAc in Hexane), $[\alpha]_D^{25}$ = +93.0 (1.0, DCM).

^{1}H NMR (400 MHz, $CDCl_3$) δ 7.74 (d, J = 15.4 Hz, 1H), 7.49 (d, J = 15.4 Hz, 1H), 7.44 – 7.29 (m, 5H), 6.63 (d, J = 3.4 Hz, 1H), 6.40 (d, J = 3.4 Hz, 1H), 5.54 (dd, J = 8.7, 3.9 Hz, 1H), 4.73 (t, J = 8.8 Hz, 1H), 4.43 (s, 2H), 4.29 (dd, J = 8.9, 4.0 Hz, 1H), 3.41 (s, 3H) ppm; ^{13}C NMR (101 MHz, $CDCl_3$) δ 164.9, 155.3, 153.8, 151.5, 139.2, 132.7, 129.3, 128.8, 126.1, 117.1, 114.5, 111.8, 70.1, 66.6, 58.5, 58.0 ppm.

HRMS-EI (m/z) M^+: Calcd for $C_{18}H_{17}NO_5$, 327.1107; found, 327.1108.

Preparation of amide 25:

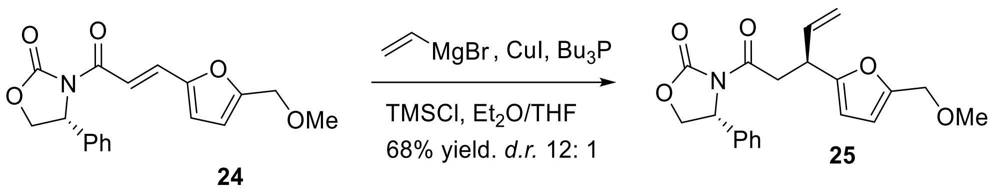

Procedure I: To a suspension of CuBr-DMS (2.64 g, 12.8 mmol) in Et_2O (75 mL) at -40 °C was added vinylmagnesium bromide (25.7 mL, 1 M in THF, 25.7 mmol). After stirring at this temperature for 1 h, the reaction mixture was cooled to -78 °C, then TMSCl (3.30 mL, 25.7 mmol) was added. After 20 min, **24** (2.80 g, 8.6 mmol) in Et_2O/THF (15 mL/ 15 mL) was added. The reaction was stirred at – 78 °C for 5 h, then quenched by the addition of a mixture of saturated NH_4Cl (aq.) / NH_3-H_2O (60 mL / 30 mL). The mixture was allowed to warm to rt. The reaction mixture was separated, and the aqueous layer was back-extracted with EtOAc (3 x 100 mL). The combined organic extracts were washed with saturated $NaHCO_3$ (aq.) and brine, and dried over anhydrous $MgSO_4$. The volatiles were removed under reduced pressure and the residue was purified by flash column chromatography (EtOAc in hexane: 15% to 20%) to give **25** as a yellow oil (2.20 g, 72% yield).

Procedure II: Bu_3P (63.4 mL, 0.254 mol) was added to a suspension of CuI (24.3 g, 0.127 mol) in Et_2O (400 mL). The mixture was stirred for 10 min to obtain a clear solution, then it was cooled to – 78 °C. Vinylmagnesium bromide (0.127 L, 1 M in THF, 0.127 mol) was added via cannula. After 30 min, **24** (27.7 g, 84.9 mmol) in THF (100 mL) was added, then the reaction mixture was allowed to warm to -10 °C over 3 h. A mixture of saturated NH_4Cl (aq.) and NH_3-H_2O (100 mL/ 50 mL) was added. The resulting mixture was stirred for 1 h and filtered through celite. The filtrate was separated, and the aqueous layer was extracted with EtOAc (3 x 150 mL). The combined organic extracts were washed with saturated $NaHCO_3$ (aq.) and brine, and dried over anhydrous $MgSO_4$. The volatiles were removed under reduced pressure and the residue was purified by flash column chromatography (EtOAc / Hexane: 15% to 20%) to give **25** as a yellow oil (20.5 g, 68% yield).

R_f: 0.33 (30% EtOAc in Hexane), $[\alpha]_D^{25}$ = -56.1 (1.40, DCM).

^{1}H NMR (400 MHz, $CDCl_3$) δ 7.42 – 7.19 (m, 6H), 6.20 (d, J = 3.2 Hz, 1H), 5.98 (d, J = 3.2 Hz, 1H), 5.89 (ddd, J = 17.4, 10.3, 7.4 Hz, 1H), 5.40 (dd, J = 8.7, 3.7 Hz, 1H), 5.06 – 4.95 (m, 2H), 4.67 (t, J = 8.8 Hz, 1H), 4.32 (s, 2H), 4.26 (dd, J = 8.9, 3.7 Hz, 1H), 4.00 (q, J = 7.3 Hz, 1H), 3.48 (dd, J = 16.8, 7.4 Hz, 1H), 3.37 – 3.28 (m, 4H), 1.61 (s, 1H) ppm; ^{13}C NMR (101 MHz, $CDCl_3$) δ 170.6, 155.9, 153.8, 150.7, 139.0, 137.2, 129.2, 128.8, 126.1, 116.5, 110.3, 106.4, 70.1, 66.5, 57.8, 57.8, 39.0, 38.8 ppm.

HRMS-EI (m/z) M^+: Calcd for $C_{20}H_{21}NO_5$, 355.1420; found, 355.1413.

Preparation of alcohol S23:

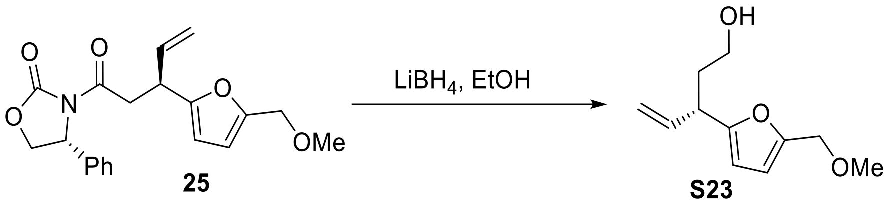

To $LiBH_4$ (5.2 g, 225 mmol) in THF (130 mL) at -30 °C was added **25** (16.1 g, 45 mmol) in THF (20 mL). EtOH (13.1 mL, 225 mmol) was then added. After being stirred for 2.5 h at the same temperature, the reaction was carefully quenched with saturated NH_4Cl aqueous solution (50 mL). The mixture was extracted with EtOAc (3 x 50 mL). The combined organic extracts were washed with $NaHCO_3$ (sat.) and brine, and dried over anhydrous $MgSO_4$. The volatiles were removed under reduced pressure and the residue was purified by flash column chromatography (EtOAc/Hexane: 30%) to give **S23** as a yellow oil (6.40 g, 73% yield).

R_f: 0.28 (30% EtOAc in Hexane), $[\alpha]_D^{25}$ = +41.2 (0.79, DCM).

^{1}H NMR (400 MHz, $CDCl_3$) δ 6.23 (d, J = 3.1 Hz, 1H), 5.99 (d, J = 3.1 Hz, 1H), 5.88 (ddd, J = 17.4, 10.1, 8.0 Hz, 1H), 5.13 (d, J = 10.4 Hz, 1H), 5.11 – 5.06 (m, 1H), 4.34 (s, 2H), 3.66 (t, J = 6.3 Hz, 2H), 3.57 (q, J = 7.6 Hz, 1H), 3.34 (s, 3H), 2.09 – 2.00 (m, 1H), 1.93 – 1.84 (m, 1H), 1.72 (brs, 1H) ppm; ^{13}C NMR (101 MHz, $CDCl_3$) δ 157.4, 150.5, 138.6, 116.2, 110.3, 105.8, 66.5, 60.7, 57.8, 40.2, 36.2 ppm.

HRMS-EI (*m*/*z*) M$^+$: Calcd for $C_{11}H_{16}O_3$, 196.1099; found, 196.1096.

Preparation of iodide 26:

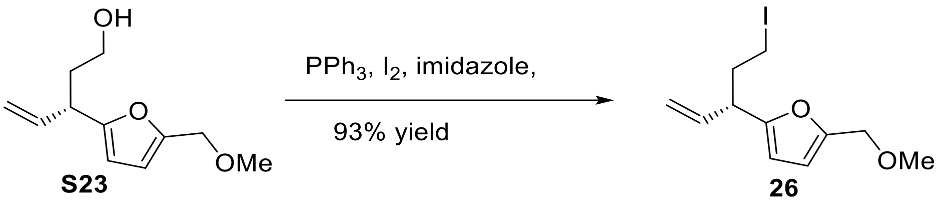

To **S23** (4.12 g, 21.0 mmol) in anhydrous DCM (70 mL) at 0 °C was added sequentially PPh_3 (8.26 g, 31.5 mmol), imidazole (2.86 g, 42.0 mmol) and I_2 (8.00 g, 31.5 mmol). The cool bath was removed and the reaction mixture was stirred for 2 h at rt. Saturated aqueous $Na_2S_2O_3$ solution (40 mL) was added and the reaction mixture was stirred for 10 minutes. The mixture was separated, and the aqueous layer was back-extracted with Et_2O (3 x 50 mL). The combined organic extracts were washed with $Na_2S_2O_3$ (sat.) and brine, then dried over anhydrous $MgSO_4$. The volatiles were removed under reduced pressure and the residue was purified by flash column chromatography (EtOAc/Hexane: 5%) to give **26** as a colorless oil (5.96 g, 93% yield).

R_f: 0.27 (10% EtOAc in Hexane), $[\alpha]_D^{25}$ = +29.9 (1.48, DCM).

^{1}H NMR (400 MHz, $CDCl_3$) δ 6.23 (d, *J* = 3.1 Hz, 1H), 6.02 (d, *J* = 3.1 Hz, 1H), 5.81 (ddd, *J* = 17.2, 10.1, 8.1 Hz, 1H), 5.22 – 5.12 (m, 2H), 4.34 (s, 2H), 3.54 (q, *J* = 7.6 Hz, 1H), 3.35 (s, 3H), 3.19 – 3.10 (m, 2H), 2.31 (dq, *J* = 14.2, 7.2 Hz, 1H), 2.10 (dq, *J* = 14.4, 6.9 Hz, 1H) ppm; ^{13}C NMR (101 MHz, $CDCl_3$) δ 156.1, 150.8, 137.2, 117.0, 110.2, 106.3, 66.5, 57.9, 44.2, 36.7, 4.0 ppm.

HRMS-EI (*m*/*z*) M$^+$: Calcd for $C_{11}H_{15}IO_2$, 306.0117; found, 306.0108.

Preparation of vinyl iodide 27: [74, 110]

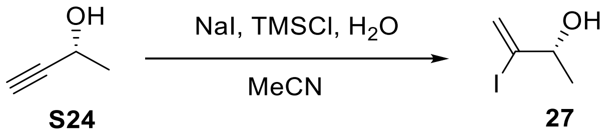

To a stirred solution of NaI (20.1 g, 134.2 mmol) in acetonitrile (160 mL) was added TMSCl (17.0 mL, 134.2 mmol) and water (1.45 mL, 80.5 mmol) sequentially at room temperature. After 10 min, alcohol **S24** (4.95 g, 85.9 mmol) was added in one portion. After a further 90 min at room temperature, the reaction mixture was diluted with water (100 mL), and partitioned between Et_2O (500 mL) and 5% aq. $Na_2S_2O_3$ (50 mL). The layers were separated, and the aqueous layer was back-extracted with Et_2O (3 × 100 mL). The combined organic extracts were washed with $NaHCO_3$ (sat.), water (3 times) and brine, and dried over anhydrous $MgSO_4$. The volatiles were removed under reduced pressure and the residue was purified by flash column chromatography (EtOAc/Hexane: 2% to 10%) to give **27** as an orange oil (6.86 g, 52% yield).

R_f: 0.48 (20% EtOAc in Hexane), $[\alpha]_D^{25}$ = +1.94 (1.44, DCM).

^{1}H NMR (500 MHz, $CDCl_3$) δ 6.36 (dd, J = 1.8, 1.1 Hz, 1H), 5.82 (d, J = 1.8 Hz, 1H), 3.98 – 3.87 (m, 1H), 1.92 (d, J = 5.3 Hz, 1H), 1.31 (d, J = 6.3 Hz, 3H) ppm; ^{13}C NMR (126 MHz, $CDCl_3$) δ 124.5, 119.7, 74.4, 23.3 ppm

HRMS-EI (m/z) M^+: Calcd for C_4H_7IO, 197.9542; found, 197.9537.

Preparation of allylic alcohol 28:

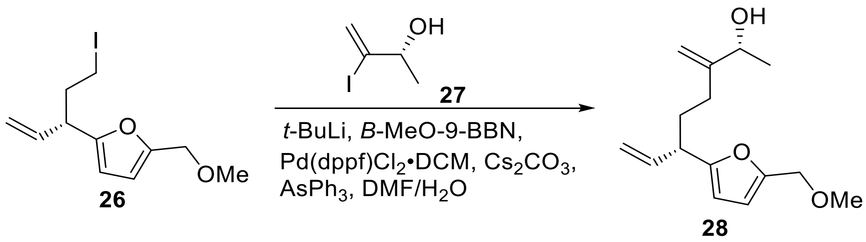

To **26** (4.40 g, 14.4 mmol) in Et_2O (60 mL) at -78 °C was added t-BuLi (23.0 mL, 1.3 M in pentane, 16.5 mmol). After 10 min, *B*-MeO-9-BBN (33.8 mL, 1.0 M in hexane, 33.8 mmol) was added. After the addition was finished, THF (60 mL) was added and the cold bath was removed. The reaction mixture was stirred at rt for 4 h. In another flask, **27** (2.37 g, 12.0 mmol) in DMF (120 mL) and Cs_2CO_3 (24.0 mL, 2.5 M in H_2O, 59.9 mmol) were added, then the mixture above was added to **27** via cannula. To the reaction mixture was added $AsPh_3$ (0.550 g, 1.80 mmol) and Pd(dppf)Cl_2-DCM (0.489 g, 0.598 mmol). The reaction mixture was stirred for 18 h in the dark. The reaction was quenched with saturated aqueous NH_4Cl solution (50 mL), then the mixture was extracted with Et_2O (3 x 100 mL). The combined organic extracts were washed with $NaHCO_3$ (sat.), H_2O (2 x) and brine, then dried over anhydrous $MgSO_4$. The volatiles were removed under reduced pressure and the residue was purified by flash chromatography on a short silica gel column (EtOAc / Hexane: 30%) to give **28** as a yellow oil 3.06 g which was pure enough to be used directly in next step. An analytically pure sample of **28** was obtained by further purification by flash column chromatography (EtOAc / Hexane: 10% to 15%) as a yellow oil with the following characteristics:

R_f: 0.27 (20% EtOAc in Hexane), $[\alpha]_D^{25}$ = +45.2 (1.98, DCM).

^{1}H NMR (500 MHz, $CDCl_3$) δ 6.23 (d, *J* = 3.1 Hz, 1H), 5.98 (d, *J* = 3.1 Hz, 1H), 5.85 (ddd, *J* = 16.6, 10.7, 8.1 Hz, 1H), 5.11 (s, 1H), 5.08 (d, *J* = 7.4 Hz, 1H), 5.05 (s, 1H), 4.81 (s, 1H), 4.34 (s, 2H), 4.24 (q, *J* = 6.4 Hz, 1H), 3.38 (dd, *J* = 14.6, 7.5 Hz, 1H), 3.34 (s, 3H), 2.06 (q, *J* = 7.5 Hz, 2H), 2.03 – 1.93 (m, 1H), 1.84 – 1.74 (m, 1H), 1.68 (d, *J* = 24.9 Hz, 2H), 1.26 (d, *J* = 6.5 Hz, 3H) ppm; ^{13}C NMR (126 MHz, $CDCl_3$) δ 157.8, 152.9, 150.3, 138.9, 116.1, 110.2, 108.7, 105.7, 70.9, 66.5, 57.8, 43.4, 31.8, 29.3, 22.3 ppm.

HRMS-ESI (*m*/*z*) $[M+Na]^+$: Calcd for $C_{15}H_{22}NaO_3$, 273.1461; found, 273.1464.

Preparation of epoxide S25:

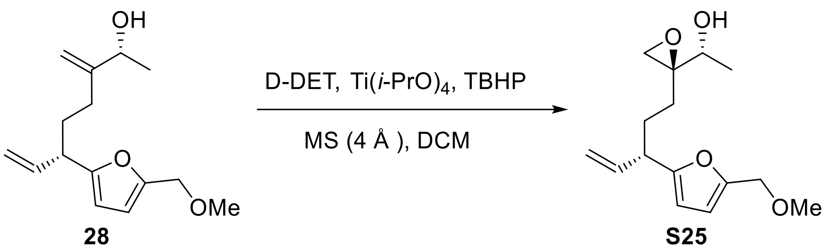

To D-DET (0.492 mL, 2.88 mmol), MS (4Å, 3.0 g) in DCM (40 mL) at -20 °C was added Ti(i-PrO)$_4$ (0.702 mL, 2.40 mmol). After 30 min, **28** (3.00 g, 12.0 mL) in DCM (2 mL) was added. After stirring for 30 min, TBHP (4.79 mL, 5.5 M in decane, 26.4 mmol) was added. The reaction mixture was stirred overnight, then treated with a mixture of ferrous sulfate, tartaric acid and H_2O (24g/24g/400g, 10 mL). The mixture was stirred at rt for 1 h and extracted with DCM (3 x 10 mL). The combined organic extracts were concentrated to ~ 15 mL, and cooled to 0 °C. To this was added NaOH (10 wt% in brine, 10 mL). This reaction mixture was stirred for 1 h, then extracted with DCM (3 x 20 mL). The combined organic extracts were washed with brine, then dried over anhydrous $MgSO_4$. The volatiles were removed under reduced pressure and the residue was purified by flash column chromatography (EtOAc / Hexane: 10% to 20%) to give **S25** as a colorless oil (2.06 g, 65% yield over 2 steps from **27**).

R_f: 0.30 (30% EtOAc in Hexane), $[\alpha]_D^{25}$ = +36.3 (0.60, DCM).

^{1}H NMR (400 MHz, $CDCl_3$) δ 6.22 (d, J = 2.9 Hz, 1H), 5.96 (d, J = 2.9 Hz, 1H), 5.89 – 5.72 (m, 1H), 5.09 (dd, J = 12.7, 5.5 Hz, 2H), 4.33 (s, 2H), 3.89 (q, J = 6.0 Hz, 1H), 3.40 – 3.23 (m, 4H), 2.86 (d, J = 4.5 Hz, 1H), 2.61 (d, J = 4.5 Hz, 1H), 2.18 (s, 1H), 1.82 (ddd, J = 21.5, 14.7, 8.9 Hz, 3H), 1.70 – 1.53 (m, 2H), 1.19 (d, J = 6.3 Hz, 3H) ppm; ^{13}C NMR (101 MHz, $CDCl_3$) δ 157.2, 150.3, 138.2, 116.3, 110.1, 105.7, 66.4, 66.1, 62.3, 57.7, 48.2, 43.4, 28.5, 27.5, 18.5 ppm.

HRMS-ESI (m/z) $[M+Na]^+$: Calcd for $C_{15}H_{22}NaO_4$, 289.1410; found, 289.1417.

Preparation of epoxy ketone S26:

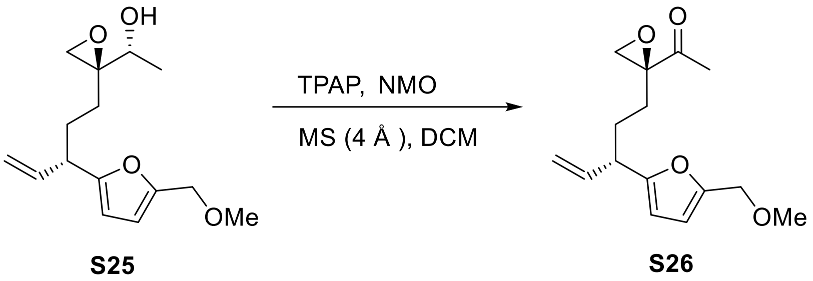

To a mixture of **S25** (1.70 g, 6.39 mmol), MS (4Å) (6.40 g) in DCM (30 mL) was added NMO (1.50 g, 12.8 mmoL) and TPAP (0.225 g, 0.640 mmol). The mixture was stirred for 2.5 h. The reaction was filtered through celite, and the celite plug was washed with DCM. The filtrates were collected and concentrated, and the residue was purified by flash column chromatography on silica gel (EtOAc /Hexane: 5% to 10%) to give **S26** as a yellow oil (1.53 g, 90% yield).

R_f: 0.40 (20% EtOAc in Hexane), $[\alpha]_D^{25}$ = +6.89 (1.51, DCM).

^{1}H NMR (400 MHz, $CDCl_3$) δ 6.21 (d, J = 3.0 Hz, 1H), 5.97 (d, J = 3.1 Hz, 1H), 5.81 (ddd, J = 16.7, 10.6, 8.2 Hz, 1H), 5.11 (s, 1H), 5.07 (d, J = 7.1 Hz, 1H), 4.32 (s, 2H), 3.38 – 3.26 (m, 4H), 2.92 (d, J = 4.9 Hz, 1H), 2.85 (d, J = 4.9 Hz, 1H), 2.19 (ddd, J = 14.1, 11.7, 4.4 Hz, 1H), 2.01 (s, 3H), 1.90 (ddd, J = 17.8, 12.0, 5.6 Hz, 1H), 1.71 – 1.59 (m, 1H), 1.50 (ddd, J = 14.0, 11.6, 4.9 Hz, 1H) ppm; ^{13}C NMR (101 MHz, $CDCl_3$) δ 207.6, 157.3, 150.5, 138.3, 116.4, 110.2, 105.8, 66.5, 62.6, 57.8, 50.9, 43.4, 28.3, 27.9, 23.9 ppm.

HRMS-ESI (*m/z*) [M+Na]$^+$: Calcd for $C_{15}H_{20}NaO_4$, 287.1254; found 287.1249.

Preparation of enolsilane 29:

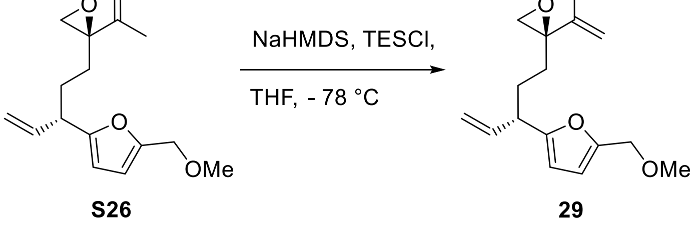

To **S26** (1.49 g, 5.8 mmol) in THF (15 mL) at -78 °C was added NaHMDS (5.64 mL, 2.0 M in THF, 11.3 mmol). After 30 min, TESCl (2.84 mL, 16.9 mmol) was added, and the reaction mixture was stirred at the same temperature about 1 hour. After the reaction was judged to be completed as monitored by TLC, the reaction was quenched by the addition of saturated aqueous $NaHCO_3$. The mixture was allowed to warm to rt, then extracted with Et_2O (3 x 10 mL). The combined organic extracts were washed with brine, then dried over anhydrous $MgSO_4$. The volatiles were removed under reduced pressure, and the residue was purified by flash column chromatography (EtOAc/ Et_3N/ Hexane: 1/ 0.5/ 98.5) to give **29** as a colorless oil (1.99 g, 93% yield).

R_f: 0.53 (5% EtOAc in Hexane), $[\alpha]_D^{25}$ = -2.13 (3.38, DCM).

^{1}H NMR (400 MHz, $CDCl_3$) δ 6.22 (d, J = 3.1 Hz, 1H), 5.97 (d, J = 3.1 Hz, 1H), 5.81 (ddd, J = 14.3, 9.7, 8.1 Hz, 1H), 5.10 (s, 1H), 5.07 (d, J = 3.7 Hz, 1H), 4.39 (d, J = 1.3 Hz, 1H), 4.33 (s, 2H), 4.20 (d, J = 1.3 Hz, 1H), 3.38 – 3.28 (m, 4H), 2.77 (d, J = 5.8 Hz, 1H), 2.66 (d, J = 5.8 Hz, 1H), 2.03 – 1.89 (m, 2H), 1.79 – 1.66 (m, 1H), 1.55 – 1.44 (m, 1H), 0.97 (t, J = 7.9 Hz, 9H), 0.69 (q, J = 8.0 Hz, 6H) ppm; ^{13}C NMR (101 MHz, $CDCl_3$) δ 157.7, 156.5, 150.4, 138.7, 116.1, 110.2, 105.6, 90.2, 66.5, 59.1, 57.8, 53.9, 43.5, 30.1, 28.7, 6.8, 4.9 ppm.

HRMS-ESI (*m/z*) $[M+Na]^+$: Calcd for $C_{21}H_{34}NaO_4Si$, 401.2119; found, 401.2125.

Synthetic Protocols to Prepare 31:

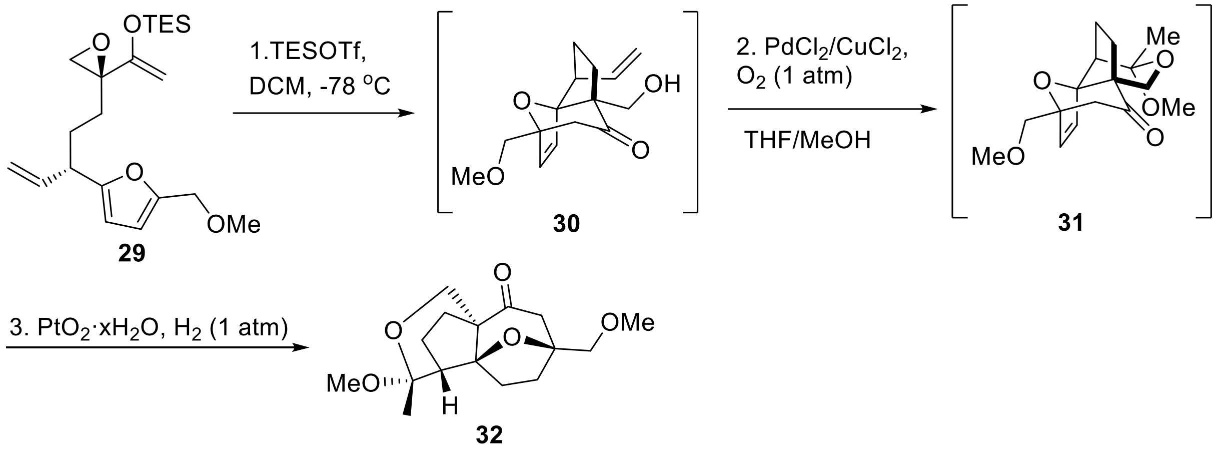

Stepwise procedure:

Step 1: To a solution of enolsilane **29** (75.6 mg, 0.2 mmol) in anhydrous CH_2Cl_2 (4 mL) at –78 °C was added Et_3SiOTf (0.2 mL, freshly prepared 0.2 M in CH_2Cl_2, 0.04 mmol). After stirring at –78 °C for 30 min, $Et_3N{\cdot}3HF$ (0.1 mL) was added, and the mixture was warmed to room temperature over 1 hour. Saturated aqueous $NaHCO_3$ (1 mL) was added to the reaction until the reaction mixture stopped bubbling. The mixture was extracted with Et_2O three times. The combined organic extracts were dried over anhydrous $MgSO_4$, and concentrated by rotary evaporation. The residue was purified by flash chromatography on silica gel (20 % EtOAc in hexanes) to afford **30** (25.3 mg, 48% yield) as a colorless oil.

30: R_f: 0.22 (30% EtOAc in Hexane), $[\alpha]_D^{25}$ = -77.2 (0.707, DCM).

^{1}H NMR (500 MHz, $CDCl_3$) δ 6.17 (d, J = 6.0 Hz, 1H), 6.11 (d, J = 5.9 Hz, 1H), 5.88 (ddd, J = 17.0, 10.3, 7.6 Hz, 1H), 5.18 – 5.07 (m, 2H), 3.61 – 3.52 (m, 2H), 3.47 (d, J = 6.4 Hz, 2H), 3.44 (s, 3H), 3.01 – 2.93 (m, 1H), 2.84 (t, J = 6.6 Hz, 1H), 2.72 (d, J = 18.0 Hz, 1H), 2.23 (d, J = 18.0 Hz, 1H), 2.21 – 2.11 (m, 2H), 2.04 – 1.95 (m, 1H), 1.88 – 1.78 (m, 1H) ppm; ^{13}C NMR (126 MHz, $CDCl_3$) δ 215.4, 138.9, 134.9, 133.3, 116.2, 97.8, 85.2, 75.3, 66.4, 61.9, 59.9, 49.4, 42.5, 29.2, 27.8 ppm.

HRMS-ESI (m/z) $[M+Na]^+$: Calcd for $C_{15}H_{20}NaO_4$, 287.1254; found, 287.1253.

Step 2: To a solution of **30** (67.1 mg, 0.23 mmol) in THF/ MeOH (4 mL/ 0.4 mL), was added $PdCl_2$ (4.10 mg, 0.023 mmol) and $CuCl_2$ (47.3 mg, 0.35 mmol). The mixture was bubbled with O_2 via a balloon for 3 h, and stirred overnight. The reaction was quenched by the addition of saturated $NaHCO_3$ (aq.). The resultant mixture was filtered through celite, and the celite plug was washed with Et_2O. The filtrates were pooled then separated, and the aqueous layer was back-extracted with Et_2O (3 x 5 mL). The combined organic extracts were dried over anhydrous $MgSO_4$, and concentrated by rotary evaporation. The residue was purified by flash chromatography on silica gel (20 % EtOAc in hexanes) to afford **31** (50.5 mg, 75% yield) as a colorless oil.

31: R_f: 0.37 (20% EtOAc in Hexane), $[\alpha]_D^{25}$ = -149.2 (1.18, DCM).

^{1}H NMR (500 MHz, $CDCl_3$) δ 6.90 (d, J = 6.0 Hz, 1H), 6.15 (d, J = 6.0 Hz, 1H), 3.79 (d, J = 11.4 Hz, 1H), 3.61 – 3.50 (m, 2H), 3.41 (s, 3H), 3.32 (d, J = 11.4 Hz, 1H), 3.23 (s, 3H), 2.84 (d, J = 15.8 Hz, 1H), 2.33 – 2.26 (m, 1H), 2.17 (d, J = 15.8 Hz, 1H), 2.14 – 2.06 (m, 2H), 1.91 - 1.86 (m, 1H), 1.85 – 1.75 (m, 1H), 1.34 (s, 3H) ppm; ^{13}C NMR (126 MHz, $CDCl_3$) δ 208.2, 135.7, 135.4, 103.6, 94.4, 84.9, 75.2, 66.1, 59.9, 59.5, 48.6, 47.5, 43.7, 29.5, 25.6, 21.2 ppm.

HRMS-ESI (*m/z*) $[M+Na]^+$: Calcd for $C_{16}H_{22}NaO_5$, 317.1359; found, 317.1352.

Step 3**: Pd/C-catalyzed hydrogenation:** To **31** (200 mg, 0.68 mmol) in hexane (3.5 mL) was added Pd/C (10.0 mg). The reaction mixture was stirred under 1 atm H_2 (via a balloon) for 2.5 h, and the reaction progress was monitored by TLC. The reaction mixture was filtered through celite, and the celite plug was washed with EtOAc. The filtrates were pooled, and concentrated under reduced pressure. The residue was purified by flash column chromatography (EtOAc / Hexane: 10%) to give **32** as a white solid (187 mg, 93% yield). **$PtO_2 \cdot xH_2O$-catalyzed hydrogenation**: To **31** (78.3 mg, 0.266 mmol) in hexane (5 mL) was added $PtO_2 \cdot xH_2O$ (3.9 mg). The reaction mixture was stirred under 1 atm H_2 (via a balloon) for 2 h, and the reaction progress was monitored by TLC. The reaction mixture was filtered through celite, and

the celite plug was washed with EtOAc. The filtrates were pooled and concentrated under reduced pressure. The residue was purified by flash column chromatography (EtOAc / Hexane: 10%) to give **32** as a white solid (68.0 mg, 87% yield).

32: R_f: 0.38 (20% EtOAc in Hexane), $[\alpha]_D^{25}$ = -178.2 (1.33, DCM). mp: 39 – 40 °C.

^{1}H NMR (500 MHz, $CDCl_3$) δ 3.79 (d, J = 11.6 Hz, 1H), 3.55 (d, J = 11.6 Hz, 1H), 3.45 – 3.39 (m, 5H), 3.15 (s, 3H), 3.00 (d, J = 15.3 Hz, 1H), 2.23 (ddd, J = 14.1, 9.7, 4.1 Hz, 1H), 2.19 – 2.07 (m, 4H), 2.05 (d, J = 15.3 Hz, 1H), 1.86 – 1.73 (m, 3H), 1.64 – 1.57 (m, 3H), 1.27 (s, 3H) ppm; ^{13}C NMR (126 MHz, $CDCl_3$) δ 210.5, 103.2, 93.2, 82.1, 77.1, 65.9, 59.8, 57.5, 51.4, 47.3, 46.9, 32.1, 32.0, 28.3, 25.6, 21.0 ppm.

HRMS-ESI (m/z) $[M+Na]^+$: Calcd for $C_{16}H_{24}NaO_5$, 319.1516; found, 319.1515.

One-pot Procedure: To **29** (0.415 g, 1.10 mmol) in DCM (20 mL) at -78 °C was added TESOTf that was precooled to -78 °C (1.10 mL, 0.2 M in DCM, 0.4 mmol). The reaction was stirred -78 °C for 2.5 h, while the reaction progress was monitored by TLC. When the reaction was deemed complete, MeOH (2 mL) was added.

This reaction mixture was added via cannula to another flask containing a mixture of $PdCl_2$ (19.4 mg, 0.110 mmol), $CuCl_2$ (22.1 mg, 0.164 mmol) in THF/ MeOH (10 mL/ 2 mL) at 0 °C. The first flask was rinsed with THF (5 mL), and the rinsing was also added to the reaction by cannula. The reaction mixture was bubbled through with O_2 via a balloon for 3 h, then further stirred for 16 h.

$PtO_2 \cdot xH_2O$ (93.0 mg) was added, and the reaction mixture was stirred under 1 atm H_2 (via a balloon) for 3 h, while the reaction was monitored by TLC. When the reaction was complete, saturated aqueous $NaHCO_3$ (30 mL) was added, and the resultant mixture was filtered through celite. The celite plug was washed with EtOAc. The filtrates were pooled then separated, and the aqueous layer was back-extracted

with EtOAc (3 x 50 mL). The combined organic extracts were washed with brine, then dried over anhydrous $MgSO_4$. The volatiles were removed under reduced pressure and the residue was purified by flash column chromatography (EtOAc / Hexane: 5% to 8% to 10%) to give **32** as a white solid (146 mg, 45% yield over 3 steps).

Preparation of bromoalcohol 33:

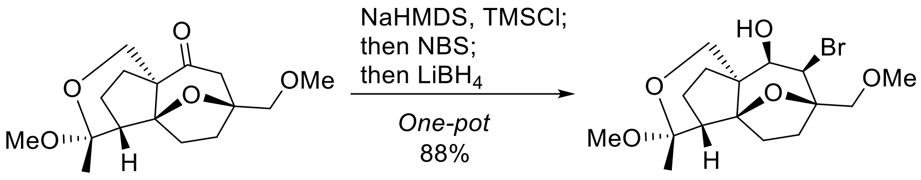

32 **33**

To **32** (0.145 g, 0.490 mmol) in THF (2 mL) at -78 °C was added NaHMDS (0.490 mL, 2.0 M in THF, 0.980 mmol). After 20 min, TMSCl (0.190 mL, 1.47 mmol) was added.

After stirring for another 35 min at -78 °C, $NaHCO_3$ (28.8 mg, 0.340 mmol) and then NBS (0.105 g, 0.590 mmol) were added sequentially. The reaction mixture was allowed to warm to 0 °C, while the reaction was monitored by TLC.

Then $LiBH_4$ (0.74 mL, 2 M in THF, 1.5 mmol) was added, and the reaction was stirred at 0 °C for 1 h. Saturated NH_4Cl aqueous solution (3 mL) was added carefully, and the resultant mixture was extracted with EtOAc (3 x 5 mL). The combined organic extracts were washed with saturated $NaHCO_3$ solution and brine, then dried over anhydrous $MgSO_4$. The volatiles were removed under reduced pressure and the residue was purified by flash column chromatography (EtOAc / Hexane: 15%) to give **33** as a sticky oil (0.163 g, 88% yield).

R_f: 0.21 (20% EtOAc in Hexane), $[\alpha]_D^{25}$ = -58.8 (0.673, DCM).

^{1}H NMR (400 MHz, $CDCl_3$) δ 6.22 (d, J = 3.1 Hz, 1H), 5.97 (d, J = 3.1 Hz, 1H), 5.81 (ddd, J = 14.3, 9.7, 8.1 Hz, 1H), 5.10 (s, 1H), 5.07 (d, J = 3.7 Hz, 1H), 4.39 (d, J = 1.3 Hz, 1H), 4.33 (s, 2H), 4.20

(d, J = 1.3 Hz, 1H), 3.38 – 3.28 (m, 4H), 2.77 (d, J = 5.8 Hz, 1H), 2.66 (d, J = 5.8 Hz, 1H), 2.03 – 1.89 (m, 2H), 1.79 – 1.66 (m, 1H), 1.55 – 1.44 (m, 1H), 0.97 (t, J = 7.9 Hz, 9H), 0.69 (q, J = 8.0 Hz, 6H) ppm; ^{13}C NMR (101 MHz, $CDCl_3$) δ 157.7, 156.5, 150.4, 138.7, 116.1, 110.2, 105.6, 90.2, 66.5, 59.1, 57.8, 53.9, 43.5, 30.1, 28.7, 6.8, 4.9 ppm.

HRMS-ESI (m/z) $[M+Na]^+$: Calcd for $C_{16}H_{25}NaO_5Br$, 399.0778, 401.0759; found, 399.0772, 401.0755.

Preparation of allylic alcohol 34:

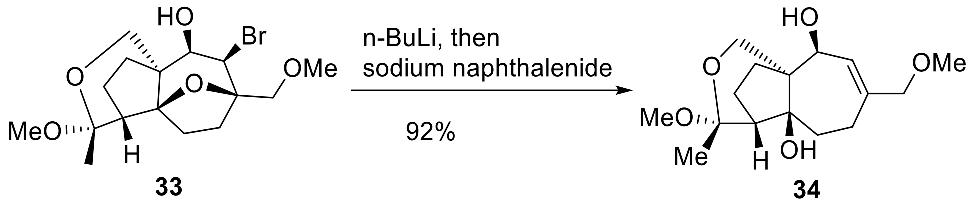

Preparation of sodium naphthalenide: An oven-dried round bottom flask was equipped with a stir bar, and charged with argon. The total weight of the flask was recorded. Dry hexane (3 mL) was added, followed by the addition of freshly cut sodium metal. The sodium metal was cut into small pieces using a spatula working in the hexane. After stirring for 2 min, the hexane supernatant was removed by syringe. Fresh dry hexanes was added, stirred and removed by syringe. This hexane washing procedure was repeated 3 times. In the final washing, the remaining hexane was removed by a dry argon stream, then the flask containing the washed sodium metal was weighed. The flask was found to contain 95.5 mg (4.15 mmol) of sodium metal. Dry THF (4 mL) was added, followed by the addition of a solution of naphthalene (638 mg, 4.98 mmol) in THF (2.93 mL). The mixture was stirred for 2.5 h to generate a dark blue solution of sodium naphthalenide.

To a solution of **33** (163 mg, 0.43 mmol) in THF (4 mL) at -78 °C was added n-BuLi (0.27 mL, 2.4 M in hexane, 0.65 mmol). After 10 min, sodium naphthalenide (3.58 mL, 0.6 M in THF, 2.15 mmol) was added. The reaction was kept at this temperature for 1 h, while the reaction progress was monitored by

TLC. When the reaction was deemed complete, aqueous NaH_2PO_4 (5 mL, 1 M in H_2O) was added to quench the reaction, resulting in a colorless mixture. The reaction mixture was extracted with EtOAc (3 x 8 mL). The combined organic extracts were washed with saturated $NaHCO_3$ solution and brine, then dried over anhydrous $MgSO_4$. The volatiles were removed under reduced pressure and the residue was purified by flash column chromatography (EtOAc / Hexane: 40%) to give **34** as a semi-solid (118 mg, 92% yield).

R_f: 0.23 (50% EtOAc in Hexane), $[\alpha]_D^{25}$ = -30.0 (1.24, DCM).

^{1}H NMR (500 MHz, $CDCl_3$) δ 5.82 (d, J = 7.5 Hz, 1H), 4.46 (brs, 1H), 4.09 (d, J = 7.4 Hz, 1H), 3.88 (dd, J = 13.3, 1.7 Hz, 1H), 3.79 (d, J = 13.6 Hz, 1H), 3.55 (d, J = 11.0 Hz, 1H), 3.35 (s, 3H), 3.15 (s, 3H), 2.97 (d, J = 11.0 Hz, 1H), 2.90 – 2.72 (m, 2H), 2.41 – 2.30 (m, 1H), 2.12 – 2.00 (m, 1H), 1.93 – 1.83 (m, 2H), 1.81 – 1.75 (m, 1H), 1.68 – 1.56 (m, 2H), 1.31 (s, 3H) ppm; ^{13}C NMR (126 MHz, $CDCl_3$) δ 145.9, 124.4, 103.7, 84.0, 77.5, 71.3, 69.1, 58.3, 55.4, 47.8, 47.2, 32.2, 28.7, 24.1, 23.5, 22.1 ppm.

HRMS-ESI (m/z) $[M+Na]^+$: Calcd for $C_{16}H_{26}NaO_5$, 321.1672; found, 321.1671.

Preparation of thiourea 35:

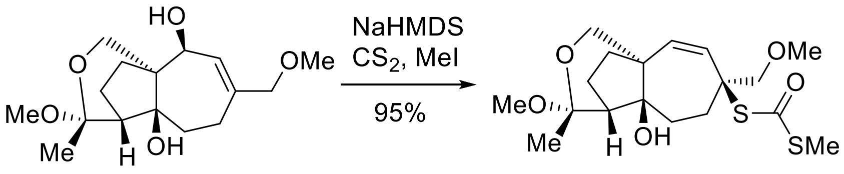

34 **35**

To a solution of **34** (31.6 mg, 0.110 mmol) in THF (1.5 mL) at -78 °C was added NaHMDS (0.11 mL, 2 M in THF, 0.22 mmol). After 20 min, CS_2 (27 µL, 0.44 mmol) was added. The reaction temperature was allowed to warm to –60 °C over 1 h. MeI (27 µL, 0.44 mmol) was added, the reaction was kept at –60 °C for 1 h, before the cold bath was removed. After stirring for 1 h at rt, the reaction was quenched by the addition of saturated NH_4Cl (aq.). The reaction mixture was extracted with EtOAc (3 x 5 mL). The

combined organic extracts were washed with saturated $NaHCO_3$ solution and brine, then dried over anhydrous $MgSO_4$. The volatiles were removed under reduced pressure and the residue was purified by flash column chromatography (EtOAc/Hexane: 7%) to give **35** (39.1 mg, 95% yield).

R_f: 0.47 (20% EtOAc in Hexane), $[\alpha]_D^{25}$ = -160.5 (0.987, DCM).

^{1}H NMR (500 MHz, $CDCl_3$) δ 5.63 (dd, J = 12.7, 1.4 Hz, 1H), 5.29 (d, J = 12.6 Hz, 1H), 3.55 (s, 1H), 3.43 – 3.38 (m, 5H), 3.29 (d, J = 10.0 Hz, 1H), 3.23 (d, J = 11.1 Hz, 1H), 3.20 (s, 3H), 2.66 – 2.53 (m, 2H), 2.37 (s, 3H), 2.34 – 2.27 (m, 1H), 2.15 – 2.08 (m, 1H), 2.04 – 1.95 (m, 2H), 1.93 – 1.86 (m, 1H), 1.81 – 1.75 (m, 1H), 1.62 – 1.56 (m, 1H), 1.33 (s, 3H) ppm; ^{13}C NMR (126 MHz, $CDCl_3$) δ 191.0, 133.6, 129.0, 104.5, 79.8, 76.7, 70.7, 62.9, 59.8, 54.0, 52.0, 47.3, 33.1, 29.6, 29.5, 24.4, 22.0, 13.1 ppm.

HRMS-ESI (m/z) $[M+Na]^+$: Calcd for $C_{18}H_{28}NaO_5S$, 411.1270; found, 411.1267.

Preparation of methyl ester 37:

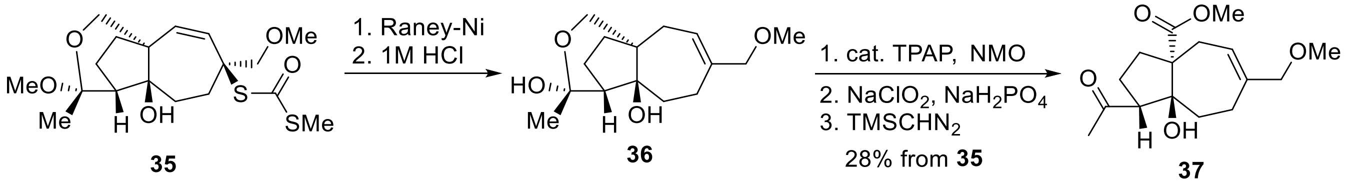

To **35** (43.9 mg, 0.11 mmol) in EtOH (2.0 mL) at 0 °C was added Raney Ni (suspension in water, excess). After 30 min, TLC monitoring suggested the reaction was complete. The reaction mixture was filtered through celite, and the celite plug was washed with EtOAc (3 mL x 2). The filtrates were pooled and concentrated under reduced pressure. The residue was dissolved in THF (2.0 mL) and cooled to 0 °C. HCl (1.0 N, 0.15 mL) was added, then the mixture was stirred for 30 min. The reaction progress was monitored by TLC. When the reaction was complete, it was quenched by the addition of saturated $NaHCO_3$ (aq., 3.0 mL). The reaction mixture was extracted with EtOAc (3 mL x 3). The extracts were pooled, washed with brine, then dried over anhydrous $MgSO_4$. After concentrating under reduced

pressure, the residue was filtered through a short silica gel plug using DCM/MeOH (100/ 1 mixture) to give a crude product **36** (21.6 mg), which was used directly in the next step without further purification.

To the crude **36** (21.6 mg) in MeCN (1.60 mL) at 0 °C was added NMO (94.8 mg, 0.81 mmol), and TPAP (2.80 mg, 0.0081 mmol) to sequentially. After 4 h, the reaction mixture was filtered through a short silica gel plug using with DCM/MeOH (50/ 1 mixture). The filtrates were pooled, and concentrated to give a crude product, which was used directly in next step.

To the above crude product in *t*-BuOH (1.60 mL) was added NaH_2PO_4 (0.28 mL, 1.0 M in H_2O, 0.28 mmol), 2-methyl-2-butene (0.26 mL, 90% purity, 2.43 mmol) and $NaClO_2$ (45.8 mg, 80% purity, 0.41 mmol) sequentially. The reaction mixture was stirred for 14 h, then H_2O (3.0 mL) was added, followed by EtOAc (3.0 mL) and HCl (1 N, 4 drops). The mixture was separated and the aqueous layer was extracted with EtOAc (3.0 mL x 3). The combined organic extracts were washed with brine, then dried over anhydrous $MgSO_4$. After concentrating under reduced pressure, the residue was used directly in next step without further purification.

The above residue was dissolved in DCM/ MeOH (0.8 mL/ 0.8 mL). $TMSCHN_2$ (0.12 mL, 2.0 M in hexane, 0.24 mmol) was added and stirred. The volatiles were removed under reduced pressure, and the residue was purified by flash column chromatography on silica gel (DCM/ MeOH: 100/1) to give **37** as a colorless oil (9.4 mg, 28% yield for 5 steps from **35)**.

R_f: 0.33 (DCM/ MeOH: 20/ 1), $[\alpha]_D^{25}$ = -72.5 (0.331, DCM).

^{1}H NMR (500 MHz, $CDCl_3$) δ 5.63 – 5.54 (m, 1H), 3.82 – 3.69 (m, 2H), 3.56 (s, 3H), 3.22 (s, 3H), 2.87 (dd, J = 9.3, 5.2 Hz, 1H), 2.61 – 2.55 (m, 1H), 2.55 – 2.43 (m, 2H), 2.39 (dd, J = 15.0, 8.2 Hz, 1H), 2.25 – 2.16 (m, 4H), 2.13 – 2.03 (m, 3H), 1.98 - 1.93 (m, 1H), 1.87 - 1.80 (m, 1H), 1.72 (s, 1H) ppm; ^{13}C NMR (126 MHz, $CDCl_3$) δ 206.6, 174.8, 141.0, 125.5, 84.8, 78.3, 66.1, 57.8, 57.4, 51.7, 34.3, 32.6, 31.0, 29.5, 22.9, 22.0 ppm.

HRMS-ESI (*m/z*) $[M+Na]^+$: Calcd for $C_{16}H_{24}NaO_5$, 319.1516; found, 319.1517.

Preparation of 6:

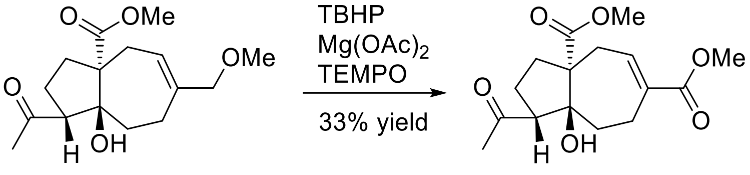

37 **6**

To **37** (4.00 mg, 0.0135 mmol) in EtOAc (0.4 mL) at 0 °C was added sequentially $Mg(OAc)_2 \cdot 4H_2O$ (4.34 mg, 0.0203 mmol), $PhI(OAc)_2$ (13.0 mg, 0.0405 mmol) and TBHP (17.2 μL, 5.5 M in decane, 0.0945 mmol). The reaction mixture was stirred for 22 h at 0 °C, then concentrated under reduced pressure. The residue was purified by flash column chromatography on silica gel (EtOAc/ hexane: 30%) to give **6** as a colorless oil (1.9 mg, 33% yield).

R_f: 0.40 (50% EtOAc in Hexane), $[\alpha]_D^{25}$ = -35.2 (0.233, DCM).

^{1}H NMR (600 MHz, $CDCl_3$) δ 6.97 – 6.89 (m, 1H), 3.70 (s, 3H), 3.56 (s, 3H), 2.86 (dd, J = 9.3, 5.1 Hz), 2.84-2.81 (m, 1H), 2.75 (ddd, J = 14.8, 4.8, 1.5 Hz, 1H), 2.54 (dd, J = 14.7, 8.6 Hz, 1H), 2.51 – 2.44 (m, 2H), 2.28 – 2.18 (m, 1H), 2.21 (s, 3H), 2.16 – 2.08 (m, 2H), 2.00 (dt, J = 12.9, 9.8 Hz, 1H), 1.90 – 1.83 (m, 1H), 1.59 (brs, 1H) ppm; ^{13}C NMR (151 MHz, $CDCl_3$) δ 206.2, 174.3, 168.3, 140.4, 135.7, 84.4, 66.3, 57.2, 52.1, 51.9, 34.4, 32.6, 30.9, 29.9, 21.9, 20.4 ppm.

HRMS-ESI (m/z) $[M+Na]^+$: Calcd for $C_{16}H_{22}NaO_6$, 333.1309; found, 333.1312.

A Comparison of Characterization data for Chiu's (–)-**6** and Trost's (–)-**6**

^{1}H NMR (in $CDCl_3$)		^{13}C NMR (in $CDCl_3$)	
Chiu's **6** (600 MHz)	**6** (400 MHz)[33, 34]	Trost's **6** (126 MHz)	**6** (100 MHz)[33, 34]

6.97 – 6.89 (m, 1H)	6.97-6.89 (m, 1H)	206.2	206.2
3.70 (s, 3H)	3.69 (s, 3H)	174.3	174.1
3.56 (s, 3H)	3.55 (s, 3H)	168.3	168.1
2.86 (dd, *J* = 9.3, 5.1 Hz)	2.87 (dd, *J* = 9.3, 4.9 Hz),	140.4	140.3
2.84-2.81 (m, 1H)	2.84-2.79 (m, 1H)	135.7	135.5
2.75 (ddd, J = 14.8, 4.8, 1.5 Hz, 1H)	2.76 (dd, *J* = 14.8, 4.9 Hz)	84.4	84.2
2.54 (dd, J = 14.7, 8.6 Hz, 1H), 2.52 – 2.44 (m, 2H)	2.58-2.39 (m, 3H)	66.3	66.1
2.28 – 2.18 (m, 1H)	2.27-2.16 (m, 1H)	57.2	57.0
2.21 (s, 3H)	2.21 (s, 1H)	52.1	51.9
2.16 – 2.08 (m, 2H)	2.16-2.06 (m, 2H)	51.9	51.7
2.00 (dt, J = 12.9, 9.8 Hz, 1H)	2.00 (dt, *J* = 12.6, 9.6 Hz, 1H)	34.4	34.2
1.90 – 1.83 (m, 1H)	1.92-1.83 (m, 1H)	32.6	32.4
1.59 (brs, 1H)	1.81 (brs, 1H)	30.9	30.7
		29.9	29.7
		21.9	21.7,
		20.4	20.2

Optical rotation of Chiu's sample: $[\alpha]_D^{25}$ = -35.2 (0.233, DCM)

Optical rotation of Trost's sample reported: $[\alpha]_D^{25}$ = -51.2 (1.0, $CHCl_3$)

4. X-Ray Crystallographic Data

Crystal Structure Determination

Crystals suitable for X-ray diffraction were mounted on a MiTeGen dual-thickness micro-mount and placed under a cold stream of nitrogen (Oxford). Single-crystal X-ray diffraction measurements were recorded on a Bruker D8 VENTURE Duo FIXED-CHI X-Ray Diffractometer using a IμS micro-focus Mo-Kα (λ = 0.71073 Å) or Cu-Kα (λ = 1.54178 Å) radiations with a Quazar multilayer optics. Data collection was conducted with the APEX3 v2015.5.2 or v2019.3-2click4 (Bruker AXS 2015, Nano 2019) program. Cell refinement and data reduction were performed with the SAINT V8.38A (Bruker AXS Inc., 2017) program. The structure was solved using XT 2014/5[1a] in the APEX3 suite and refined with SHELXL2014/7 or 2018/3[1b]. Hydrogen atoms were placed in idealized positions and were set riding on the respective parent atoms. All non-hydrogen atoms were refined with anisotropic thermal parameters. The structure was refined by weighted least squares refinement on F^2 to convergence. All e.s.d.'s are estimated using the full covariance matrix. The X-ray crystallographic data of XX have been deposited at the Cambridge Crystallographic Data Centre (CCDC), under the deposition number 2380345 for compound **19** and CCDC 2017565 for compound **32**. The data can be obtained free of charge from the Cambridge Crystallographic Data Center (https://www.ccdc.cam.ac.uk/structures/).

[1] a) G. M. Sheldrick, Acta Cryst. 2015, A71, 3-8. ; b) G. M. Sheldrick, Acta Cryst. 2015, C71, 3-8.

Structure determination of compound 18

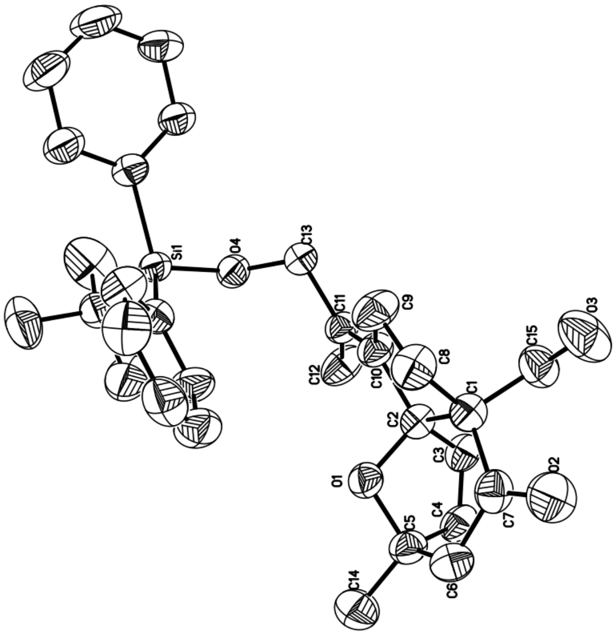

Figure S1. ORTEP of the molecular structure of **18** showing thermal ellipsoids at the 50% probability level. **CCDC** 2380345

Experimental details

Crystal data	
Chemical formula	$C_{31}H_{38}O_4Si$
M_r	502.70
Crystal system, space group	Monoclinic, $P2_1$
Temperature (K)	299
a, b, c (Å)	7.9112 (3), 39.0524 (12), 9.7891 (3)
β (°)	112.705 (1)
V (Å^3)	2789.98 (16)
Z	4
Radiation type	Cu $K\alpha$
μ (mm^{-1})	1.00
Crystal size (mm)	0.74 × 0.46 × 0.36
Data collection	
Diffractometer	Bruker D8 VENTURE FIXED-CHI PHOTON 100 CMOS
Absorption correction	Multi-scan<br>*SADABS* 2014/5 (Sheldrick, 2014)
T_{min}, T_{max}	0.526, 0.753
No. of measured, independent and observed [$I > 2\sigma(I)$] reflections	38190, 9669, 9238
R_{int}	0.046
$(\sin \theta/\lambda)_{max}$ (Å^{-1})	0.595
Refinement	
$R[F^2 > 2\sigma(F^2)]$, $wR(F^2)$, S	0.042, 0.108, 1.06
No. of reflections	9669
No. of parameters	673
No. of restraints	32
H-atom treatment	H atoms treated by a mixture of independent and constrained refinement
$\Delta\rho_{max}$, $\Delta\rho_{min}$ (e Å^{-3})	0.22, −0.22
Absolute structure	Flack x determined using 4105 quotients [(I+)-(I-)]/[(I+)+(I-)] (Parsons, Flack and Wagner, Acta Cryst. B69 (2013) 249-259).
Absolute structure parameter	−0.003 (11)

Computer programs: *APEX3* v2015.5-2 (Bruker-AXS, 2015), *SAINT* v8.34A (Bruker-AXS, 2007), *SHELXT2014*/5 (Sheldrick, 2015), *SHELXL2014*/7 (Sheldrick, 2014), Bruker *SHELXTL*.

Datablock: cu_CHEM0193

Bond precision: C-C = 0.0062 A Wavelength=1.54178

Cell:	a=7.9112(3)	b=39.0524(12)	c=9.7891(3)
	alpha=90	beta=112.705(1)	gamma=90
Temperature:	299 K		

	Calculated	Reported
Volume	2789.98(16)	2789.98(16)
Space group	P 21	P 21
Hall group	P 2yb	P 2yb
Moiety formula	C31 H38 O4 Si	C31 H38 O4 Si
Sum formula	C31 H38 O4 Si	C31 H38 O4 Si
Mr	502.70	502.70
Dx,g cm-3	1.197	1.197
Z	4	4
Mu (mm-1)	1.003	1.003
F000	1080.0	1080.0
F000’	1083.93	
h,k,lmax	9,46,11	9,46,11
Nref	9890[5024]	9669
Tmin,Tmax	0.601,0.696	0.526,0.753
Tmin’	0.453	

Correction method= # Reported T Limits: Tmin=0.526 Tmax=0.753
AbsCorr = MULTI-SCAN

Data completeness= 1.92/0.98 Theta(max)= 66.647

R(reflections)= 0.0423(9238) wR2(reflections)= 0.1077(9669)

S = 1.060 Npar= 673

The following ALERTS were generated. Each ALERT has the format
test-name_ALERT_alert-type_alert-level.
Click on the hyperlinks for more details of the test.

Alert level C

```
PLAT242_ALERT_2_C Low    'MainMol' Ueq as Compared to Neighbors of       C16B Check
PLAT242_ALERT_2_C Low    'MainMol' Ueq as Compared to Neighbors of        C16 Check
PLAT340_ALERT_3_C Low Bond Precision on  C-C Bonds ...............     0.0062 Ang.
PLAT480_ALERT_4_C Long H...A H-Bond Reported H4      ..  O3B     ..      2.61 Ang.
PLAT911_ALERT_3_C Missing # FCF Refl Between THmin & STh/L=  0.595         30 Report
PLAT978_ALERT_2_C Number C-C Bonds with Positive Residual Density           0 Note
```

Alert level G

```
PLAT002_ALERT_2_G Number of Distance or Angle Restraints on AtSite          8 Note
PLAT003_ALERT_2_G Number of Uiso or Uij Restrained non-H Atoms ...          5 Report
PLAT007_ALERT_5_G Number of Unrefined Donor-H Atoms ..............          1 Report
PLAT063_ALERT_4_G Crystal Size Likely too Large for Beam Size ....       0.74 mm
PLAT171_ALERT_4_G The CIF-Embedded .res File Contains EADP Records          2 Report
PLAT172_ALERT_4_G The CIF-Embedded .res File Contains DFIX Records          5 Report
PLAT173_ALERT_4_G The CIF-Embedded .res File Contains DANG Records          2 Report
PLAT176_ALERT_4_G The CIF-Embedded .res File Contains SADI Records          2 Report
PLAT177_ALERT_4_G The CIF-Embedded .res File Contains DELU Records          1 Report
PLAT186_ALERT_4_G The CIF-Embedded .res File Contains ISOR Records          2 Report
PLAT230_ALERT_2_G Hirshfeld Test Diff for    O3C    --  C15C    ..        6.3 s.u.
PLAT301_ALERT_3_G Main Residue  Disorder ............ Percentage =          3 Note
PLAT343_ALERT_2_G Unusual sp3      Angle Range in Main Residue for     C2B    Check
PLAT380_ALERT_4_G Incorrectly? Oriented X(sp2)-Methyl Moiety .....       C12B Check
PLAT791_ALERT_4_G The Model has Chirality at C1      (Chiral SPGR)          S Verify
PLAT791_ALERT_4_G The Model has Chirality at C2      (Chiral SPGR)          S Verify
PLAT791_ALERT_4_G The Model has Chirality at C2B     (Chiral SPGR)          S Verify
PLAT791_ALERT_4_G The Model has Chirality at C5      (Chiral SPGR)          R Verify
PLAT791_ALERT_4_G The Model has Chirality at C5B     (Chiral SPGR)          R Verify
PLAT860_ALERT_3_G Number of Least-Squares Restraints .............         32 Note
PLAT909_ALERT_3_G Percentage of Observed Data at Theta(Max) Still          90 %
PLAT933_ALERT_2_G Number of OMIT records in Embedded RES .........          1 Note
```

```
 0 ALERT level A = Most likely a serious problem - resolve or explain
 0 ALERT level B = A potentially serious problem, consider carefully
 6 ALERT level C = Check. Ensure it is not caused by an omission or oversight
22 ALERT level G = General information/check it is not something unexpected

 0 ALERT type 1 CIF construction/syntax error, inconsistent or missing data
 8 ALERT type 2 Indicator that the structure model may be wrong or deficient
 5 ALERT type 3 Indicator that the structure quality may be low
14 ALERT type 4 Improvement, methodology, query or suggestion
 1 ALERT type 5 Informative message, check
```

Structure determination of compound 32

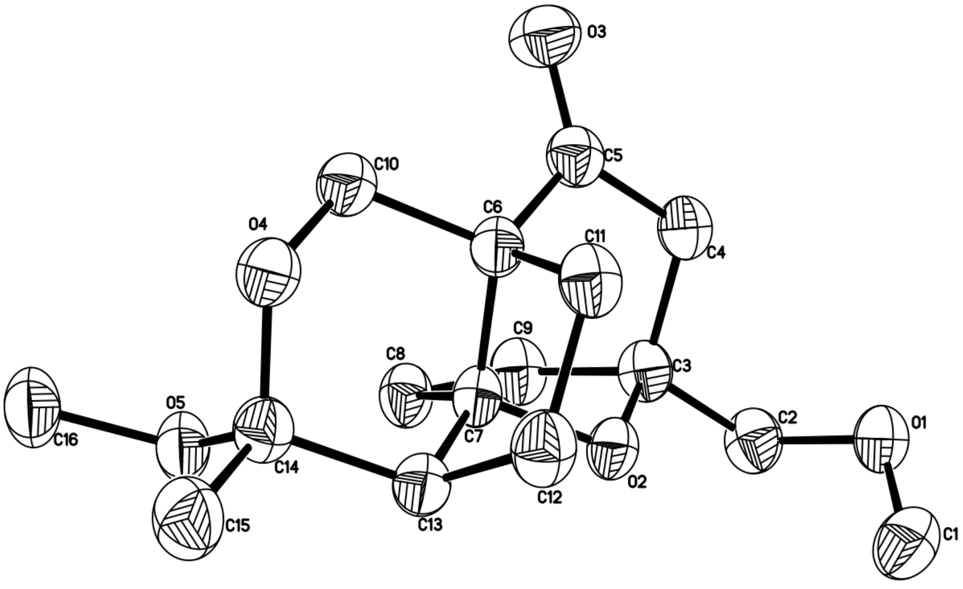

Figure S2. ORTEP of the molecular structure of **32** showing thermal ellipsoids at the 50% probability level. **CCDC** 2017565.

Crystal data

$C_{16}H_{24}O_5$
M_r = 296.35
Monoclinic, $P2_1$
a = 7.7772 (5) Å
b = 11.4840 (7) Å
c = 8.5260 (5) Å
β = 101.087 (3)°
V = 747.27 (8) Å^3
Z = 2

$F(000)$ = 320
D_x = 1.317 Mg m^{-3}
Ga $K\alpha$ radiation, λ = 1.34138 Å
Cell parameters from 9054 reflections
θ = 4.6–63.5°
μ = 0.51 mm^{-1}
T = 199 K
Plate, colorless
0.24 × 0.12 × 0.04 mm

Data collection

′Bruker D8 VENTURE KAPPA goniometer PHOTON II CPAD′ diffractometer
Radiation source: Excillum MetalJet D2
Detector resolution: 7.3910 pixels mm^{-1}
φ and ω scans
Absorption correction: multi-scan
SADABS2014/5 - Bruker AXS area detector scaling and absorption correction

T_{min} = 0.625, T_{max} = 0.752
15450 measured reflections
3631 independent reflections
3137 reflections with $I > 2\sigma(I)$
R_{int} = 0.047
θ_{max} = 63.5°, θ_{min} = 4.6°
h = −10→10
k = −14→15
l = −11→11

Refinement

Refinement on F^2
Least-squares matrix: full
$R[F^2 > 2\sigma(F^2)] = 0.041$
$wR(F^2) = 0.106$
$S = 1.09$
3631 reflections
193 parameters
1 restraint
Primary atom site location: dual
Secondary atom site location: difference Fourier map

Hydrogen site location: inferred from neighbouring sites
H-atom parameters constrained
$w = 1/[\sigma^2(F_o^2) + (0.0428P)^2 + 0.1674P]$
where $P = (F_o^2 + 2F_c^2)/3$
$(\Delta/\sigma)_{max} < 0.001$
$\Delta\rho_{max} = 0.16$ e Å^{-3}
$\Delta\rho_{min} = -0.22$ e Å^{-3}
Absolute structure: Flack x determined using 1272 quotients [(I+)-(I-)]/[(I+)+(I-)] (Parsons, Flack and Wagner, Acta Cryst. B69 (2013) 249-259).
Absolute structure parameter: −0.13 (12)

Datablock: ga_CHEM0869

Bond precision: C-C = 0.0036 A   Wavelength=1.34138

Cell:	a=7.7772(5)	b=11.4840(7)	c=8.5260(5)
	alpha=90	beta=101.087(3)	gamma=90
Temperature:	199 K		

	Calculated	Reported
Volume	747.27(8)	747.27(8)
Space group	P 21	P 21
Hall group	P 2yb	P 2yb
Moiety formula	C16 H24 O5	C16 H24 O5
Sum formula	C16 H24 O5	C16 H24 O5
Mr	296.35	296.35
Dx,g cm-3	1.317	1.317
Z	2	2
Mu (mm-1)	0.507	0.507
F000	320.0	320.0
F000’	320.79	
h,k,lmax	10,15,11	10,15,11
Nref	3740[1961]	3631
Tmin,Tmax	0.932,0.979	0.624,0.752
Tmin’	0.887	

Correction method= # Reported T Limits: Tmin=0.624 Tmax=0.752
AbsCorr = MULTI-SCAN

Data completeness= 1.85/0.97   Theta(max)= 63.507

R(reflections)= 0.0409(3137)   wR2(reflections)= 0.1057(3631)

S = 1.092   Npar= 193

The following ALERTS were generated. Each ALERT has the format
test-name_ALERT_alert-type_alert-level.
Click on the hyperlinks for more details of the test.

Alert level G

```
ABSMU01 ALERT 1 G  Calculation of _exptl_absorpt_correction_mu
                 not performed for this radiation type.
PLAT398 ALERT 2 G Deviating  C-O-C    Angle From 120 for O2             104.7 Degree
PLAT720 ALERT 4 G Number of Unusual/Non-Standard Labels ..........          4 Note
PLAT791 ALERT 4 G Model has Chirality at C3          (Sohnke SpGr)          S Verify
PLAT791 ALERT 4 G Model has Chirality at C6          (Sohnke SpGr)          S Verify
PLAT791 ALERT 4 G Model has Chirality at C7          (Sohnke SpGr)          S Verify
PLAT791 ALERT 4 G Model has Chirality at C13         (Sohnke SpGr)          R Verify
PLAT791 ALERT 4 G Model has Chirality at C14         (Sohnke SpGr)          R Verify
PLAT912 ALERT 4 G Missing # of FCF Reflections Above STh/L=  0.600         18 Note
PLAT978 ALERT 2 G Number C-C Bonds with Positive Residual Density.          1 Info
PLAT992 ALERT 5 G Repd & Actual _reflns_number_gt Values Differ by          1 Check
```

```
  0 ALERT level A = Most likely a serious problem - resolve or explain
  0 ALERT level B = A potentially serious problem, consider carefully
  0 ALERT level C = Check. Ensure it is not caused by an omission or oversight
 11 ALERT level G = General information/check it is not something unexpected

  1 ALERT type 1 CIF construction/syntax error, inconsistent or missing data
  2 ALERT type 2 Indicator that the structure model may be wrong or deficient
  0 ALERT type 3 Indicator that the structure quality may be low
  7 ALERT type 4 Improvement, methodology, query or suggestion
  1 ALERT type 5 Informative message, check
```

5. Computational studies.

5.1. Computational results

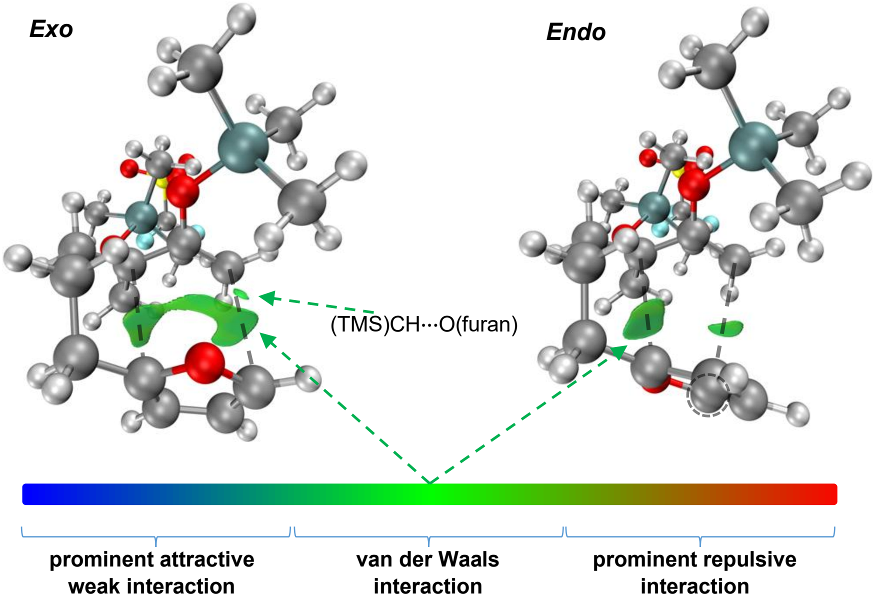

Scheme S5. IGMH weak interaction analyses showing the important weak interactions between the furan ring and epoxy enosilane in the (4+3) cycloaddition transition states for compound **11′**. The green surfaces represent regions in which stabilizing van der Waals interactions occur. More extensive van der Waals interactions are observed in the *exo* geometry, as well as a (TMS)CH···O(furan) interaction.

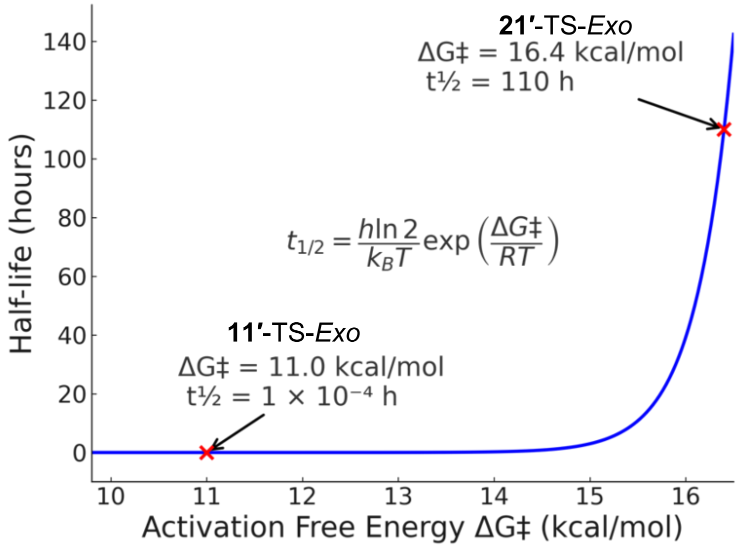

Scheme S6. Plot showing the dependence of the half-life of reactant at –78° C on the activation free energy, with crosses indicating the reaction barriers for cycloadditions of **11′** and **21′**. The calculated half-life for **21′** is too long for product to be detectable under the standard reaction conditions.

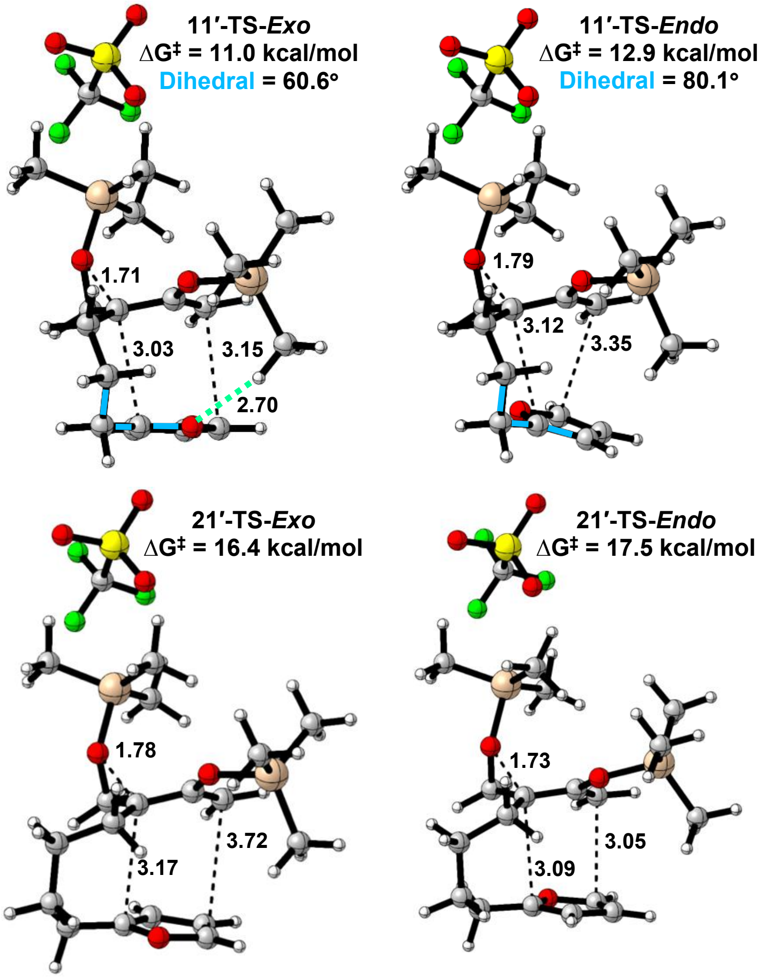

Scheme S7. Geometries of the lowest-energy *exo* and *endo* transition states of compounds **11′** and **21′**. Distances in Å.

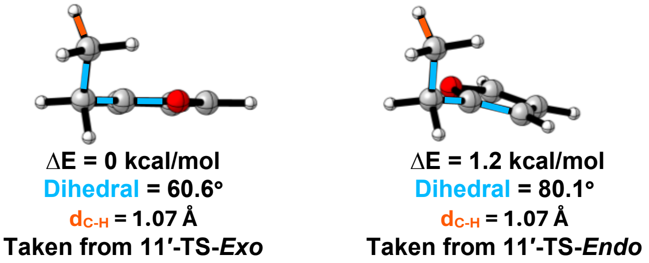

Scheme S8. Truncated fragments of the *exo* and *endo* transition states for cycloaddition of **11′** showing the effect of furan tilting (taken directly from the TS coordinates by adding a hydrogen with a bond length (d_{C-H}) of 1.07 Å at the truncated point). The *endo* fragment has a higher energy due to torsional strain associated with the highlighted dihedral.

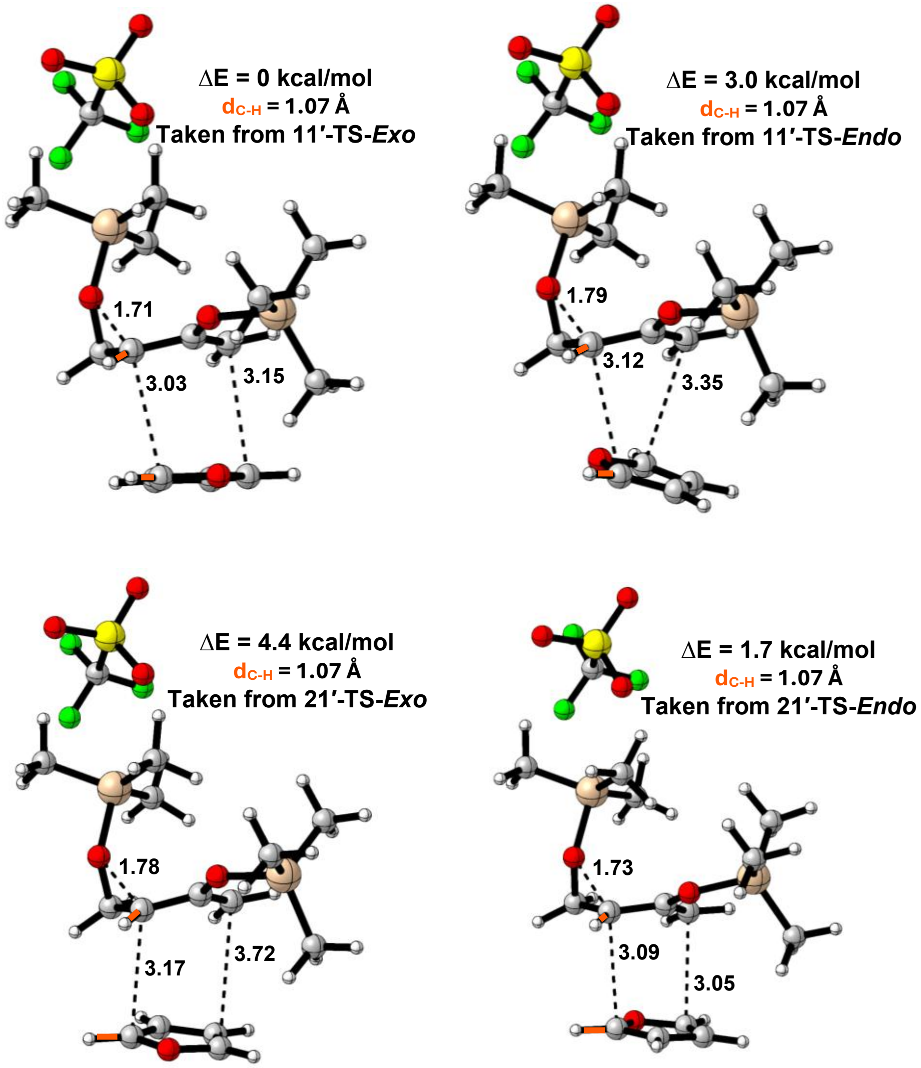

Scheme S9. Truncated fragments of the *exo* and *endo* transition states for cycloaddition of **11′** and **21′** showing the effect of the relative orientation of the epoxy enolsilane and furan moieties (taken by replacing the –$(CH_2)_3$- tether by hydrogen atoms with $d_{C\text{-}H}$ = 1.07 Å). The structure taken from **11′**-TS-*Exo* has lowest energy due to the better alignment of the reacting moieties and the less steric clashing. Distances in Å.

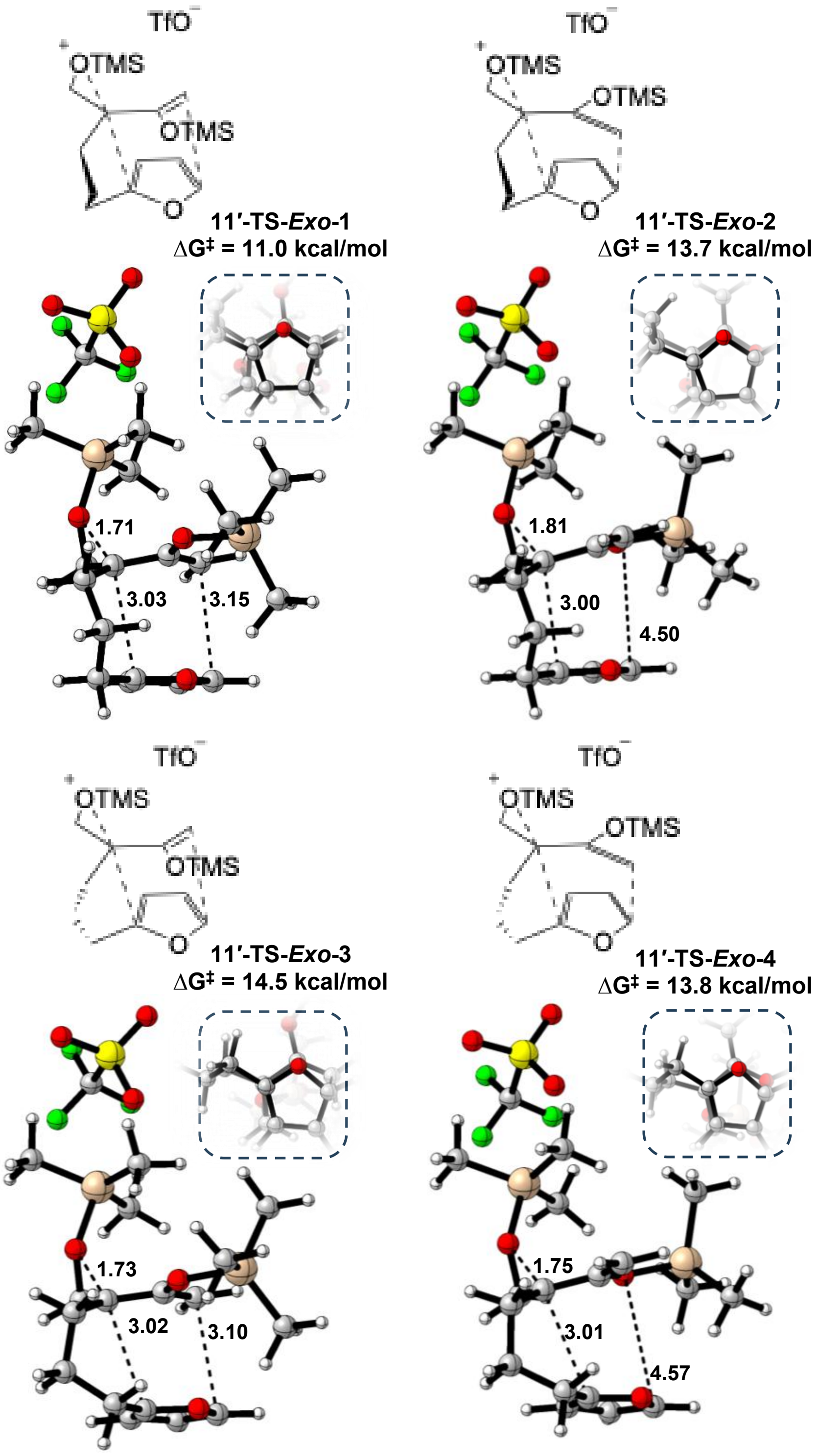

Scheme S10. Four *exo* transition states for compound **11′**, which differ with respect to the epoxy enolsilane conformation (*syn* or *anti*) and the tether conformation (two different half-chairs). The most stable conformer has the best alignment of the reacting moieties, as well as less steric clashing between the tethered chain and the epoxide ring. Distances in Å. The insets at the top right of each structure show a bottom-up view of the two reacting moieties.

5.2 Computational Methods

Density functional theory computations were performed in Gaussian 16. [28] Transition state geometries were optimized with B3LYP-D3(BJ)/6-31G(d,p) using the CPCM implicit solvent model of dichloromethane[56-63, 112, 113] Vibrational frequency calculations at this level gave thermochemical quantities. Entropies and vibrational energies are calculated using Shermo 2.6 with quasi-RRHO model developed by Grimme and Minenkov. [114-116] Single-point energies were calculated with M06-2X/def2-TZVPP using the SMD implicit solvent model of dichloromethane.[64, 65, 117] Gibbs free energies were calculated by adding the quasi-harmonically corrected B3LYP thermochemical quantities to the M06-2X single-point energy, and are reported at a standard state of 298.15 K and 1 mol/L. IGMH weak interaction analysis was conducted with Multiwfn 3.8 dev. [118-120]

The coordinates for each optimized TS are listed below, along with the following energies (all in Hartree):

E_{B3LYP} = B3LYP electronic potential energy in CPCM implicit dichloromethane

G_{B3LYP} = B3LYP Gibbs free energy (quasi-harmonically corrected) in CPCM implicit dichloromethane at 298.15 K

$E_{M06\text{-}2X}$ = M06-2X single-point electronic potential energy in SMD implicit dichloromethane

G_{tot} = total M06-2X//B3LYP Gibbs free energy in dichloromethane at 298.15 K and 1 mol/L

5.3 Molecular Coordinates Summary

Model Reactant **11′**

Charge = 0 Multiplicity = 1

C	-8.810141	-0.325447	0.264466
C	-7.751120	0.006549	-0.648632
C	-6.805865	-0.969694	-0.529791
O	-7.214192	-1.888541	0.406913
C	-5.475497	-1.199559	-1.157488
C	-4.309654	-1.080253	-0.160812
C	-2.952102	-1.304687	-0.832224
O	-0.539683	-1.711072	-0.351818
C	-1.796108	-1.171287	0.138254
C	-1.665345	0.166937	0.800374
O	-1.520145	1.143128	-0.134334
Si	-1.497091	2.838459	0.044956
C	-3.090526	3.388180	0.870805
C	-1.722934	0.340590	2.126234
C	-1.404785	3.446807	-1.722065
C	0.023267	3.346206	1.021635
H	-1.865212	-0.512535	2.776027
H	-1.638223	1.313853	2.592580
H	-2.280599	3.123540	-2.293509
H	-1.367385	4.540834	-1.751028
H	-0.510804	3.063558	-2.222882
H	0.934868	3.023888	0.509415
H	0.033393	2.918605	2.027948
H	0.060656	4.436643	1.118791
H	-3.956894	3.027290	0.306626
H	-3.140203	4.482094	0.898823

H	-3.179481	3.021588	1.896770
H	-2.809600	-0.571092	-1.633665
H	-9.724737	0.223598	0.435496
H	-7.699527	0.860343	-1.308797
H	-4.448027	-1.805675	0.649005
H	-4.335509	-0.087251	0.301471
H	-5.451481	-2.192328	-1.625958
H	-5.350744	-0.467101	-1.961800
C	-8.433553	-1.479671	0.876955
H	-8.886561	-2.111303	1.624698
H	-2.908787	-2.299076	-1.289059
C	-1.195182	-2.358108	0.758052
H	-1.616128	-3.336968	0.536383
H	-0.675276	-2.274790	1.709097
Si	2.429777	-0.349829	-0.522395
C	2.033119	-0.010791	1.269073
C	2.592500	-2.154153	-0.965249
C	1.451594	0.666141	-1.739512
H	0.989550	-0.258004	1.476864
H	2.182111	1.048041	1.500396
H	2.673130	-0.599379	1.931797
H	3.237130	-2.685756	-0.261248
H	1.595770	-2.601522	-0.941015
H	3.011106	-2.275146	-1.967340
H	1.724666	0.413199	-2.768283
H	1.642493	1.732636	-1.586146
H	0.383894	0.486042	-1.596261
O	5.597196	-1.399980	-1.522813
S	5.509774	-0.148754	-0.776447
O	4.046891	0.398429	-0.695541

O	6.400034	0.961386	-1.092183
C	5.816210	-0.577542	1.011630
F	5.549840	0.475586	1.782533
F	5.024923	-1.596295	1.373107
F	7.089499	-0.935588	1.161036

0 imaginary frequencies

E_{B3LYP}= -2432.812637

G_{B3LYP}= -2432.412849

E_{M06-2X}= -2432.724665

G_{tot}= -2432.321858

Model Reactant **21′**

Charge = 0 Multiplicity = 1

C	-9.694886	-1.430135	-0.961987
C	-8.817755	-1.709935	0.141658
C	-7.745578	-0.875866	0.016624
O	-7.903900	-0.094976	-1.103249
C	-5.223096	-1.080756	0.024859
C	-3.947854	-0.842931	0.834222
C	-2.682441	-1.221844	0.058272
O	-0.236524	-1.632821	0.320032
C	-1.413991	-0.962546	0.845370
C	-1.175900	0.466567	1.229560
O	-1.128991	1.251111	0.120449
Si	-1.028650	2.947140	-0.020484
C	-2.483039	3.724733	0.875993
C	-1.063037	0.879282	2.497870
C	-1.146321	3.223920	-1.867227
C	0.621192	3.535579	0.653830
H	-1.139857	0.164429	3.306178

H	-0.900553	1.916218	2.763217
H	-2.098883	2.852962	-2.258500
H	-1.077510	4.291401	-2.101663
H	-0.337777	2.708308	-2.393744
H	1.447157	3.082174	0.097559
H	0.750872	3.287321	1.710752
H	0.703608	4.622928	0.550315
H	-3.426462	3.319440	0.495509
H	-2.491027	4.807217	0.708338
H	-2.452349	3.552289	1.954993
H	-2.630882	-0.642089	-0.870070
H	-10.642672	-1.899793	-1.181871
H	-8.965777	-2.436635	0.927431
H	-3.989041	-1.417522	1.768965
H	-3.893827	0.213438	1.125556
H	-5.295194	-2.137997	-0.259027
H	-5.177139	-0.508969	-0.909774
C	-9.093536	-0.446498	-1.683053
H	-9.357606	0.090503	-2.580393
H	-2.705797	-2.280701	-0.220290
C	-0.764054	-2.041657	1.598442
H	-1.227213	-3.026512	1.610992
H	-0.130255	-1.810184	2.450797
Si	2.702429	-0.477243	-0.460248
C	2.551272	0.202093	1.271216
C	2.770354	-2.337634	-0.574408
C	1.598054	0.343023	-1.716524
H	3.250250	-0.293267	1.950355
H	1.535127	0.057280	1.644978
H	2.765865	1.274718	1.281416

H	3.486350	-2.759227	0.135158
H	1.775389	-2.729519	-0.349519
H	3.058968	-2.657601	-1.578611
H	1.726857	-0.109354	-2.704296
H	1.835108	1.408475	-1.793864
H	0.554277	0.246323	-1.410081
O	5.700114	-1.834307	-1.611227
S	5.729506	-0.460703	-1.118541
O	4.298672	0.152861	-0.971757
O	6.593106	0.529814	-1.749277
C	6.249886	-0.551860	0.669176
F	6.093034	0.637248	1.248731
F	5.497603	-1.455058	1.311673
F	7.527731	-0.919256	0.732171
C	-6.492650	-0.679515	0.796902
H	-6.408473	0.372118	1.102336
H	-6.571358	-1.269480	1.716040

0 imaginary frequencies

E_{B3LYP}= -2472.134087

G_{B3LYP}= -2471.708356

E_{M06-2X}= -2472.033787

G_{tot}= -2471.605037

Model TS-**21'**-*Exo* 1

Charge = 0 Multiplicity = 1

C	-4.421196	-1.173880	2.518446
C	-3.815105	-2.320822	1.901355
C	-4.323303	-2.407859	0.635710
O	-5.207458	-1.375381	0.432851
C	-4.193730	-3.418174	-0.457221

C	-4.135832	-2.859485	-1.890034
C	-2.886683	-2.059185	-2.288756
C	-2.721389	-0.700062	-1.592913
O	-0.256002	-1.140035	-0.816261
Si	1.352547	-0.340195	-0.571822
C	1.657204	-0.415362	1.269914
C	-1.891572	-0.621868	-0.348546
C	-1.910388	0.664672	0.369723
O	-2.144786	1.710714	-0.450500
Si	-2.785724	3.262287	-0.098855
C	-4.383850	3.024615	0.852312
C	-1.792152	0.720778	1.707931
C	-3.070427	3.979514	-1.800048
C	-1.513806	4.253583	0.857775
C	2.367026	-1.475488	-1.649962
C	1.058867	1.359682	-1.279871
O	4.009809	1.107247	-0.223743
S	5.382757	0.552893	-0.362983
O	6.464273	1.462977	0.068970
C	5.417311	-0.792455	0.917898
F	5.118288	-0.298902	2.133899
O	5.627089	-0.176764	-1.629044
F	4.520875	-1.756911	0.634998
F	6.630385	-1.361780	0.984799
H	-1.647208	-0.172740	2.295404
H	-1.879541	1.654464	2.247347
H	-3.774783	3.367429	-2.371626
H	-3.482004	4.991390	-1.725620
H	-2.132531	4.037091	-2.361089
H	-0.557283	4.269822	0.325506

H	-1.334548	3.863341	1.863578
H	-1.856152	5.289155	0.960701
H	-5.102050	2.454583	0.254497
H	-4.831186	3.997375	1.082382
H	-4.231598	2.493056	1.795514
H	-3.699710	-0.284861	-1.321094
H	2.415646	-2.479055	-1.217156
H	1.900907	-1.550060	-2.637646
H	3.385753	-1.100032	-1.768722
H	0.426144	1.964099	-0.626539
H	0.571857	1.291481	-2.257462
H	2.024243	1.856081	-1.396416
H	1.708417	-1.445909	1.633836
H	0.875989	0.116345	1.819624
H	2.612460	0.066486	1.481002
H	-4.248812	-0.799949	3.516805
H	-3.108853	-3.006007	2.348065
H	-1.982797	-2.666677	-2.173845
H	-2.962355	-1.859173	-3.361846
H	-5.023024	-2.238909	-2.060137
H	-4.221954	-3.710586	-2.573367
H	-3.314064	-4.035714	-0.246623
H	-5.054017	-4.099115	-0.402694
C	-5.251452	-0.635851	1.586212
H	-5.897642	0.226678	1.568113
H	-2.293837	0.040981	-2.275190
C	-1.117314	-1.752746	0.169450
H	-1.408351	-2.756276	-0.120829
H	-0.739344	-1.699738	1.185030

1 imaginary frequency

E_{B3LYP}= -2472.116342

G_{B3LYP}= -2471.686837

$E_{M06\text{-}2X}$= -2472.011350

G_{tot}= -2471.578826

Model TS-**21′**-*Exo*-2

Charge = 0 Multiplicity = 1

C	-4.772997	1.114897	1.222343
C	-4.256301	-0.077360	1.832193
C	-4.730431	-1.135116	1.106846
O	-5.507011	-0.662184	0.076201
C	-4.660807	-2.616501	1.288204
C	-4.464321	-3.448353	0.007888
C	-3.123578	-3.314665	-0.730211
C	-2.865279	-1.939608	-1.366912
O	-0.530861	-1.496569	-0.274764
Si	1.123095	-0.784528	-0.494410
C	1.384195	0.266062	1.026463
C	-2.161599	-0.895645	-0.540393
C	-2.151798	0.511597	-0.991968
O	-1.585939	1.348577	-0.098355
Si	-1.478019	3.056668	0.005922
C	0.057881	3.597971	-0.918750
C	-2.667744	0.869764	-2.180175
C	-1.319743	3.331334	1.849487
C	-3.023725	3.868580	-0.685538
C	2.078960	-2.385107	-0.578329
C	0.986286	0.097014	-2.131930
O	3.854764	0.506351	-0.936711
S	5.189041	-0.068917	-0.619461

O	6.318415	0.874282	-0.759603
C	5.092877	-0.352347	1.214372
F	4.787852	0.789980	1.856902
O	5.418589	-1.428037	-1.162561
F	4.145663	-1.260200	1.518167
F	6.263560	-0.801116	1.692710
H	-3.094319	0.140487	-2.852356
H	-2.671014	1.901037	-2.504507
H	-0.442207	2.818359	2.254760
H	-1.215313	4.398987	2.068962
H	-2.203354	2.961037	2.378419
H	-3.915013	3.266060	-0.494164
H	-2.955648	4.048690	-1.762198
H	-3.161993	4.839512	-0.197640
H	0.953074	3.099145	-0.535753
H	0.197173	4.678469	-0.802335
H	-0.019165	3.387448	-1.989888
H	-3.820556	-1.506772	-1.674885
H	2.040274	-2.911562	0.379840
H	1.640447	-3.032722	-1.344312
H	3.126260	-2.202715	-0.830292
H	0.387781	1.005613	-2.059347
H	0.530341	-0.557278	-2.881389
H	1.992545	0.366335	-2.458659
H	1.457717	-0.351284	1.926319
H	0.567048	0.977680	1.151246
H	2.317365	0.819357	0.914588
H	-4.609023	2.133608	1.539303
H	-3.631927	-0.141398	2.711567
H	-2.288983	-3.612669	-0.087276

H	-3.131822	-4.043749	-1.546035
H	-5.274369	-3.209359	-0.690800
H	-4.596270	-4.500380	0.280946
H	-3.870455	-2.830218	2.015125
H	-5.595403	-2.951263	1.758284
C	-5.517168	0.705979	0.160080
H	-6.083172	1.220280	-0.599782
H	-2.277109	-2.054855	-2.282536
C	-1.522667	-1.211553	0.740910
H	-1.822148	-2.107919	1.271382
H	-1.276044	-0.374231	1.382469

1 imaginary frequency

E_{B3LYP}= -2472.119350

G_{B3LYP}= -2471.687114

$E_{M06\text{-}2X}$= -2472.013640

G_{tot}= -2471.578385

Model TS-**21′**-*Endo*

Charge = 0 Multiplicity = 1

C	-5.278316	0.228961	1.665115
C	-5.312378	-0.541781	0.458305
C	-4.663626	-1.718863	0.716962
O	-4.228775	-1.716118	2.020241
C	-4.493217	-3.001193	-0.035031
C	-4.450994	-2.848284	-1.564905
C	-3.135305	-2.347103	-2.180616
C	-2.678393	-0.951382	-1.739312
O	-0.326304	-1.373723	-0.783882
Si	1.315507	-0.587418	-0.602691
C	1.438501	-0.109901	1.199916

C	-1.929590	-0.840497	-0.432484
C	-1.975367	0.457237	0.263154
O	-2.146450	1.487892	-0.596501
Si	-2.187147	3.174504	-0.293967
C	-3.717748	3.571615	0.714270
C	-1.924992	0.545403	1.603282
C	-2.286480	3.889016	-2.017704
C	-0.598743	3.670616	0.571316
C	2.327522	-2.053221	-1.161846
C	1.064132	0.792779	-1.833082
O	3.832905	0.791907	-0.555396
S	5.235651	0.299409	-0.480433
O	6.252352	1.361116	-0.334969
C	5.277809	-0.568637	1.161116
F	4.919502	0.270914	2.150695
O	5.561637	-0.776486	-1.444364
F	4.425289	-1.610526	1.179998
F	6.507262	-1.031993	1.429879
H	-1.797338	-0.331308	2.218658
H	-2.028820	1.490073	2.118380
H	-3.175877	3.526333	-2.542222
H	-2.339114	4.981916	-1.977617
H	-1.405662	3.614340	-2.606220
H	0.272729	3.345038	-0.005293
H	-0.513162	3.254080	1.578493
H	-0.553627	4.761581	0.658807
H	-4.618056	3.238802	0.188204
H	-3.795210	4.653844	0.864540
H	-3.708053	3.096294	1.698768
H	-3.530227	-0.272084	-1.675086

H	2.304392	-2.844474	-0.406179
H	1.902646	-2.452295	-2.088235
H	3.367947	-1.776693	-1.341822
H	0.128947	1.319169	-1.627050
H	1.011153	0.388732	-2.848996
H	1.897483	1.494139	-1.780954
H	1.225141	-0.957694	1.858528
H	0.743065	0.696856	1.439575
H	2.451961	0.232146	1.406610
H	-5.685495	1.216599	1.821617
H	-5.775807	-0.260331	-0.475494
H	-2.336794	-3.080488	-2.021837
H	-3.277502	-2.308212	-3.265188
H	-5.268383	-2.188940	-1.880289
H	-4.670031	-3.826837	-2.003764
H	-3.600012	-3.519212	0.329926
H	-5.332573	-3.661050	0.222751
C	-4.607529	-0.524851	2.579056
H	-4.322869	-0.374217	3.607927
H	-2.020494	-0.513555	-2.496486
C	-1.216668	-1.972469	0.178226
H	-1.491917	-2.983847	-0.099502
H	-0.889010	-1.885372	1.208985

1 imaginary frequency

E_{B3LYP}= -2472.115726

G_{B3LYP}= -2471.684759

E_{M06-2X}= -2472.011197

G_{tot}= -2471.577211

Model TS-**11'**-*Exo* 1

Charge = 0 Multiplicity = 1

C	3.946993	-2.697291	-2.132274
C	3.819620	-3.117346	-0.767169
C	4.439821	-2.169479	-0.000204
O	4.950675	-1.190920	-0.817010
C	4.695820	-2.016725	1.458697
C	4.106490	-0.727809	2.055857
C	2.570019	-0.721151	2.169271
O	0.210478	-1.085439	1.274472
C	1.851142	-0.845127	0.854532
C	2.054145	0.205411	-0.162905
O	2.403633	1.385734	0.396241
Si	3.115717	2.769515	-0.319455
C	4.581125	2.230495	-1.359555
C	1.928224	-0.036762	-1.479098
C	3.640860	3.766231	1.171841
C	1.818300	3.667099	-1.332601
H	1.689537	-1.021439	-1.848119
H	2.093457	0.735594	-2.218096
H	4.382850	3.223313	1.765565
H	4.084511	4.718044	0.862020
H	2.783075	3.986557	1.814858
H	0.969533	3.959557	-0.706728
H	1.436494	3.056811	-2.156520
H	2.246776	4.577986	-1.764839
H	5.195405	1.502176	-0.822322
H	5.203888	3.101615	-1.589859
H	4.277181	1.778631	-2.307384
H	2.266448	0.228494	2.616129
H	3.572130	-3.201169	-3.010960

H	3.342824	-4.014851	-0.400275
H	4.436060	0.132084	1.467730
H	4.500695	-0.590967	3.067104
H	4.292164	-2.895318	1.971716
H	5.778491	-2.016278	1.634988
C	4.633538	-1.520745	-2.104445
H	4.968327	-0.832929	-2.863945
H	2.239348	-1.528049	2.827831
C	1.052061	-2.037989	0.572303
H	1.236169	-2.929238	1.165235
H	0.739376	-2.244721	-0.445144
Si	-1.327775	-0.264718	0.707732
C	-1.585439	-0.953410	-1.009652
C	-2.425164	-0.901660	2.074948
C	-0.858566	1.536292	0.833671
H	-1.692493	-2.042245	-0.995794
H	-0.751156	-0.688350	-1.664634
H	-2.496514	-0.526731	-1.428446
H	-2.549941	-1.985644	1.996198
H	-1.960203	-0.680170	3.040961
H	-3.411692	-0.434961	2.043645
H	-0.233383	1.714282	1.713128
H	-1.771022	2.129411	0.912795
H	-0.305381	1.864751	-0.048616
O	-5.505837	0.607177	1.495454
S	-5.168717	0.835793	0.071359
O	-3.736638	1.152220	-0.181435
O	-6.126326	1.665617	-0.688459
C	-5.359066	-0.848647	-0.688886
F	-5.007429	-0.825662	-1.987923

F	-4.577231	-1.752566	-0.068193
F	-6.628907	-1.272832	-0.606768

1 imaginary frequency

E_{B3LYP}= -2432.802720

G_{B3LYP}= -2432.398453

E_{M06-2X}= -2432.711654

G_{tot}= -2432.304368

Model TS-**11′**-*Exo* 2

Charge = 0 Multiplicity = 1

C	-4.246727	0.359623	2.624060
C	-4.187794	-1.045553	2.339203
C	-4.724414	-1.223607	1.094206
O	-5.114201	-0.003802	0.591376
C	-5.024456	-2.429306	0.269367
C	-4.450196	-2.400594	-1.155508
C	-2.905386	-2.484445	-1.234084
O	-0.525960	-1.847076	-0.249088
C	-2.229588	-1.331872	-0.561587
C	-2.283614	0.027124	-1.118082
O	-1.871062	0.972946	-0.250067
Si	-1.886967	2.686830	-0.365331
C	-0.450578	3.216422	-1.444502
C	-2.705608	0.246807	-2.378128
C	-1.655598	3.207993	1.413816
C	-3.535859	3.249909	-1.073951
H	-3.001847	-0.569031	-3.020520
H	-2.758240	1.243812	-2.794139
H	-0.701073	2.848780	1.810274
H	-1.664293	4.299799	1.495569

H	-2.457509	2.809483	2.041358
H	-4.321576	2.507931	-0.908102
H	-3.476681	3.442219	-2.149246
H	-3.844617	4.181395	-0.588128
H	0.501692	2.857398	-1.042938
H	-0.407291	4.310341	-1.488879
H	-0.550137	2.848524	-2.470286
H	-2.608856	-2.525989	-2.284703
H	-3.912082	0.861145	3.520000
H	-3.812265	-1.827273	2.983553
H	-4.795343	-1.499758	-1.669219
H	-4.841878	-3.257553	-1.710628
H	-4.654363	-3.305924	0.810245
H	-6.112587	-2.547874	0.190829
C	-4.817108	0.941929	1.534904
H	-5.070396	1.958264	1.281140
H	-2.564064	-3.408856	-0.763008
C	-1.555573	-1.554033	0.725037
H	-1.830546	-2.440942	1.288104
H	-1.360779	-0.682819	1.339907
Si	1.059432	-1.015835	-0.483726
C	1.185357	0.208066	0.920669
C	2.171568	-2.511004	-0.380125
C	0.925688	-0.303104	-2.202083
H	1.296079	-0.299048	1.883330
H	0.304486	0.850556	0.957772
H	2.066798	0.830436	0.759633
H	2.143310	-2.949168	0.621869
H	1.837724	-3.268269	-1.096663
H	3.205432	-2.241875	-0.610313

H	0.545641	-1.053356	-2.902172
H	1.924051	0.006711	-2.517782
H	0.265644	0.564615	-2.237301
O	5.433318	-1.306960	-0.961547
S	5.080321	0.078126	-0.571065
O	3.721547	0.511848	-0.990760
O	6.141917	1.087522	-0.770462
C	4.930785	-0.026149	1.278023
F	4.501880	1.143037	1.787639
F	4.053240	-0.980303	1.642976
F	6.114442	-0.317478	1.839525

1 imaginary frequency

E_{B3LYP}= -2432.802438

G_{B3LYP}= -2432.397747

$E_{M06\text{-}2X}$= -2432.707762

G_{tot}= -2432.300052

Model TS-**11′**-*Exo* 3

Charge = 0 Multiplicity = 1

C	4.049193	-2.427776	-2.205495
C	4.066764	-2.739926	-0.808808
C	4.614661	-1.659186	-0.168093
O	4.953268	-0.710413	-1.093699
C	4.916137	-1.349103	1.259057
C	3.819027	-1.848734	2.206981
C	2.503471	-1.044134	2.153205
O	0.147095	-1.269927	1.148376
C	1.826008	-1.035856	0.807776
C	2.067917	0.078811	-0.134311
O	2.541661	1.179395	0.495129

Si	3.030249	2.704833	-0.111613
C	4.372319	2.495585	-1.404364
C	1.848981	-0.026308	-1.457395
C	3.686294	3.541060	1.426713
C	1.538473	3.598751	-0.812598
H	1.513299	-0.948802	-1.902634
H	2.026795	0.801761	-2.129452
H	4.541995	2.993815	1.834685
H	4.013566	4.561173	1.200589
H	2.915159	3.596958	2.201249
H	0.789549	3.783565	-0.037686
H	1.059402	3.036063	-1.619317
H	1.849598	4.566302	-1.221557
H	5.163665	1.834273	-1.043870
H	4.810638	3.475530	-1.623817
H	3.993146	2.083872	-2.343667
H	2.688125	-0.013442	2.456924
H	3.675413	-3.044211	-3.009752
H	3.723738	-3.651984	-0.342673
H	4.181914	-1.790383	3.237215
H	3.616211	-2.907310	2.012602
H	5.870717	-1.806008	1.549044
H	5.042000	-0.266461	1.361423
C	4.587289	-1.180579	-2.323540
H	4.789709	-0.528518	-3.157641
H	1.805456	-1.489214	2.865357
C	1.002602	-2.188695	0.435426
H	1.152788	-3.117848	0.980241
H	0.737187	-2.339527	-0.605291
Si	-1.404150	-0.406057	0.617432

C	-1.600125	-1.044939	-1.131993
C	-2.461232	-1.176453	1.952703
C	-0.870850	1.366104	0.888293
H	-1.526966	-2.136887	-1.161871
H	-0.828125	-0.627855	-1.784110
H	-2.571309	-0.758428	-1.530675
H	-2.586518	-2.247826	1.768927
H	-1.956116	-1.055473	2.916366
H	-3.446620	-0.712433	2.010778
H	0.094962	1.418662	1.395779
H	-1.621860	1.885398	1.485917
H	-0.787066	1.888403	-0.066962
O	-5.357301	0.629338	1.535652
S	-4.984254	0.881016	0.126100
O	-3.517099	0.999688	-0.114106
O	-5.796173	1.885976	-0.587629
C	-5.409109	-0.713429	-0.726243
F	-5.066550	-0.660392	-2.026865
F	-4.750599	-1.746770	-0.170025
F	-6.723551	-0.964607	-0.648540

1 imaginary frequency

E_{B3LYP}= -2432.799221

G_{B3LYP}= -2432.394722

E_{M06-2X}= -2432.706267

G_{tot}= -2432.298749

Model TS-**11'**-*Exo* 4

Charge = 0 Multiplicity = 1

C	-4.372683	0.480326	2.524353
C	-4.337638	-0.857987	2.007138

C	-4.859234	-0.810577	0.742676
O	-5.217386	0.480769	0.451955
C	-5.164014	-1.846913	-0.289079
C	-4.178989	-3.023481	-0.256678
C	-2.791315	-2.746140	-0.888195
O	-0.457474	-1.891971	-0.056117
C	-2.140131	-1.509203	-0.348982
C	-2.289698	-0.194500	-1.002878
O	-1.899954	0.831779	-0.219220
Si	-1.899786	2.528503	-0.496553
C	-0.456733	2.944235	-1.617712
C	-2.764490	-0.081986	-2.257197
C	-1.661115	3.212452	1.226275
C	-3.534120	3.059667	-1.258841
H	-3.038471	-0.947145	-2.840434
H	-2.885157	0.883659	-2.728780
H	-0.707923	2.889230	1.655333
H	-1.664732	4.307193	1.202672
H	-2.465026	2.879987	1.888484
H	-4.338335	2.362304	-1.013136
H	-3.470878	3.137930	-2.348339
H	-3.810930	4.046653	-0.873244
H	0.493368	2.606882	-1.193509
H	-0.400419	4.030732	-1.748675
H	-0.566149	2.498985	-2.611217
H	-2.882100	-2.693694	-1.972917
H	-4.037105	0.815262	3.494647
H	-3.996256	-1.741011	2.526559
H	-4.607125	-3.867733	-0.803283
H	-4.047425	-3.362243	0.775448

H	-6.178099	-2.234861	-0.130239
H	-5.168304	-1.371352	-1.275393
C	-4.915754	1.250471	1.544255
H	-5.141883	2.300845	1.457533
H	-2.136603	-3.590452	-0.661193
C	-1.459835	-1.593147	0.947596
H	-1.664804	-2.454768	1.577364
H	-1.295884	-0.667715	1.487200
Si	1.121803	-1.053339	-0.385508
C	1.212741	0.293936	0.903423
C	2.246337	-2.522091	-0.138910
C	0.921067	-0.538559	-2.166245
H	1.374174	-0.125931	1.900256
H	0.296836	0.885505	0.913023
H	2.053722	0.947934	0.670285
H	2.226543	-2.854871	0.903089
H	1.910887	-3.350381	-0.771047
H	3.277007	-2.273737	-0.402596
H	0.592055	-1.389720	-2.770473
H	1.887836	-0.191982	-2.535929
H	0.192350	0.264281	-2.283011
O	5.472960	-1.317961	-0.860343
S	5.062140	0.083213	-0.610011
O	3.678074	0.408393	-1.047575
O	6.070890	1.113588	-0.934244
C	4.942235	0.168350	1.242045
F	4.479306	1.369078	1.634707
F	4.104182	-0.771962	1.718856
F	6.143143	-0.022602	1.809398

1 imaginary frequency

E_{B3LYP}= -2432.801193

G_{B3LYP}= -2432.396095

E_{M06-2X}= -2432.707920

G_{tot}= -2432.299803

Model TS-**11′**-*Endo* 1

Charge = 0 Multiplicity = 1

C	4.586111	-2.078288	-2.265147
C	5.240145	-1.530877	-1.113539
C	4.620642	-2.054360	-0.015582
O	3.617330	-2.905349	-0.425163
C	4.815569	-1.874447	1.452524
C	4.178262	-0.594389	2.026239
C	2.640350	-0.625562	2.159602
O	0.215129	-1.064065	1.343607
C	1.913172	-0.780880	0.859653
C	2.070762	0.257252	-0.166977
O	2.414067	1.453195	0.357639
Si	3.141118	2.814447	-0.390268
C	4.690115	2.232849	-1.272868
C	1.930524	-0.022485	-1.476800
C	3.527211	3.917563	1.067164
C	1.905950	3.617941	-1.549588
H	1.684954	-1.016133	-1.816387
H	2.100328	0.724996	-2.239932
H	4.206124	3.419636	1.766296
H	4.004675	4.844049	0.731801
H	2.613836	4.184747	1.607583
H	0.978629	3.856117	-1.018933
H	1.652106	2.991625	-2.409393

H	2.320759	4.555971	-1.934609
H	5.418421	1.842451	-0.555167
H	5.154910	3.070900	-1.802985
H	4.484077	1.445360	-2.002691
H	2.325831	0.325505	2.596820
H	4.817474	-1.887428	-3.302789
H	6.065436	-0.834285	-1.101372
H	4.477403	0.264615	1.420845
H	4.570183	-0.427110	3.034097
H	4.431826	-2.755802	1.977360
H	5.891411	-1.832018	1.649345
C	3.612278	-2.906053	-1.794202
H	2.879500	-3.543281	-2.263181
H	2.339715	-1.430263	2.834207
C	1.088265	-1.971282	0.630372
H	1.316263	-2.856914	1.216352
H	0.759435	-2.201504	-0.377441
Si	-1.300051	-0.263114	0.758133
C	-1.579650	-0.957775	-0.953564
C	-2.437641	-0.853052	2.113796
C	-0.874494	1.551744	0.838942
H	-1.690821	-2.046142	-0.938716
H	-0.757512	-0.695414	-1.624913
H	-2.496641	-0.526540	-1.356087
H	-2.578783	-1.936406	2.058704
H	-1.996682	-0.613638	3.086784
H	-3.415720	-0.371945	2.043142
H	-0.326794	1.783331	1.757110
H	-1.804887	2.122686	0.821148
H	-0.261278	1.858897	-0.010745

O	-5.552548	0.631966	1.474840
S	-5.227123	0.835262	0.043757
O	-3.806866	1.185495	-0.223730
O	-6.213888	1.616765	-0.731079
C	-5.373582	-0.872373	-0.673625
F	-5.015115	-0.874837	-1.971089
F	-4.574928	-1.742393	-0.026340
F	-6.633831	-1.324562	-0.587435

1 imaginary frequency

E_{B3LYP}= -2432.799048

G_{B3LYP}= -2432.396832

$E_{M06\text{-}2X}$= -2432.706541

G_{tot}= -2432.301306

Model TS-**11'**-*Endo* 2

Charge = 0 Multiplicity = 1

C	-4.336935	0.442660	2.146372
C	-4.805054	-0.037980	0.877771
C	-4.680454	-1.399637	0.898611
O	-4.158461	-1.787298	2.109451
C	-4.988969	-2.486441	-0.078791
C	-4.464389	-2.203573	-1.493493
C	-2.920927	-2.201183	-1.609990
O	-0.582024	-1.662940	-0.530874
C	-2.266477	-1.128721	-0.793910
C	-2.306698	0.287916	-1.194223
O	-1.836280	1.121221	-0.242737
Si	-1.959567	2.825253	-0.079813
C	-0.828150	3.640268	-1.331958
C	-2.765252	0.656677	-2.404272

C	-1.378666	3.118006	1.672035
C	-3.746652	3.345615	-0.312843
H	-3.104587	-0.073560	-3.123712
H	-2.806591	1.695191	-2.703543
H	-0.313096	2.897804	1.783557
H	-1.535865	4.164822	1.952144
H	-1.933392	2.490242	2.375616
H	-4.385364	2.941296	0.476753
H	-4.159223	3.034599	-1.276277
H	-3.808815	4.438218	-0.264170
H	0.209787	3.328931	-1.179667
H	-0.871967	4.728152	-1.210262
H	-1.104067	3.411428	-2.365544
H	-2.643699	-2.085282	-2.659974
H	-4.293688	1.467481	2.483434
H	-5.184414	0.552987	0.057987
H	-4.862277	-1.250906	-1.854891
H	-4.834190	-2.978172	-2.171266
H	-4.566343	-3.422199	0.300882
H	-6.073827	-2.637442	-0.136505
C	-3.957324	-0.655486	2.854120
H	-3.545406	-0.805669	3.839110
H	-2.531164	-3.166001	-1.277716
C	-1.614466	-1.495726	0.469591
H	-1.890198	-2.442363	0.924264
H	-1.429089	-0.699541	1.181558
Si	1.019062	-0.827293	-0.639306
C	1.168096	0.116622	0.963421
C	2.088502	-2.345371	-0.824911
C	0.892806	0.192969	-2.195300

H	1.233051	-0.558563	1.821358
H	0.314959	0.783271	1.099388
H	2.078649	0.716408	0.930623
H	2.063327	-2.952867	0.084436
H	1.718072	-2.954514	-1.655782
H	3.126220	-2.069282	-1.027310
H	0.478781	-0.399268	-3.016852
H	1.899119	0.521425	-2.463866
H	0.265155	1.074186	-2.055682
O	5.374154	-1.172204	-1.180044
S	5.063931	0.127862	-0.540237
O	3.721069	0.674308	-0.869841
O	6.158078	1.121858	-0.552708
C	4.904795	-0.309192	1.258753
F	4.518641	0.761863	1.976033
F	3.990746	-1.280326	1.446207
F	6.074982	-0.742887	1.752371

1 imaginary frequency

E_{B3LYP}= -2432.802449

G_{B3LYP}= -2432.397320

E_{M06-2X}= -2432.708681

G_{tot}= -2432.300533

6 References for the SI

[74] N. Kamiya, Y. Chikami, Y. Ishii, Stereoselective synthesis of internal alkenyl iodides from alkynes via addition of hydrogen iodide generated in situ from a chlorotrimethylsilane/sodium iodide/water system, *Synlett* **1990**, *1990*, 675.

[76] A. X. Xiang, D. A. Watson, T. Ling, E. A. Theodorakis, Total Synthesis of Clerocidin via a Novel, Enantioselective Homoallenylboration Methodology, *J. Org. Chem.* **1998**, *63*, 6774.

[77] N. S. Mani, C. A. Townsend, A concise synthesis of (+)-cerulenin from a chiral oxiranyllithium, *J. Org. Chem.* **1997**, *62*, 636.

[87] D. Kalaitzakis, E. Antonatou, G. Vassilikogiannakis, One-pot synthesis of 1-azaspiro frameworks initiated by photooxidation of simple furans, *Chem. Commun.* **2014**, *50*, 400.

[88] J. M. Hoover, J. E. Steves, S. S. Stahl, Copper (I)/TEMPO-catalyzed aerobic oxidation of primary alcohols to aldehydes with ambient air, *Nat. Protoc.* **2012**, *7*, 1161.

[89] R. R. Cesati, G. Dwyer, R. C. Jones, M. P. Hayes, P. Yalamanchili, D. S. Casebier, Amino acid derived enamides: Synthesis and aminopeptidase activity, *Org. Lett.* **2007**, *9*, 5617.

[90] A. Basante-Avendaño, V. E. Guerra-Ayala, A. Sánchez-Eleuterio, A. Cordero-Vargas, A Free-Radical and Protecting-Group-Free Approach to (–)-Boschnialactone and γ-Lycorane, *Synthesis* **2019**, *51*, 2207.

[91] Y. Mu, F. W. Hartrampf, E. C. Yu, K. E. Lounsbury, R. R. Schrock, F. Romiti, A. H. Hoveyda, E- and Z-trisubstituted macrocyclic alkenes for natural product synthesis and skeletal editing, *Nat. Chem.* **2022**, *14*, 640.

[92] C. Chapuis, M. Barthe, B. L. Muller, K. H. Schulte-Elte, Preparation and absolute configuration of (−)-(e)-α-trans-bergamotenone, *Helv. Chim. Acta* **1998**, *81*, 153.

[93] D. Kalaitzakis, M. Triantafyllakis, I. Alexopoulou, M. Sofiadis, G. Vassilikogiannakis, One-Pot Transformation of Simple Furans into 4-Hydroxy-2-cyclopentenones in Water, *Angew. Chem. Int. Ed.* **2014**, *53*, 13201.

[94] T. Kuranaga, Y. Sesoko, K. Sakata, N. Maeda, A. Hayata, M. Inoue, Total synthesis and complete structural assignment of yaku'amide A, *J. Am. Chem. Soc.* **2013**, *135*, 5467.

[95] F. Bilodeau, L. Dubé, P. Deslongchamps, New approach toward the total synthesis of (+)-aphidicolin by tandem transannular Diels–Alder/aldol strategy, *Tetrahedron* **2003**, *59*, 2781.

[96] I. Larrosa, M. I. Da Silva, P. M. Gómez, P. Hannen, E. Ko, S. R. Lenger, S. R. Linke, A. J. White, D. Wilton, A. G. Barrett, Highly convergent three component benzyne coupling: The total synthesis of ent-clavilactone B, *J. Am. Chem. Soc.* **2006**, *128*, 14042.

[97] M. Nakano, H. Uchino, N. Iwama, M. Kashimoto, T. Kato, US Patent 8,461,365, **2013**.

[98] H. Chen, Z. Li, P. Shao, H. Yuan, S.-C. Chen, T. Luo, Total Synthesis of (+)-Mutilin: A Transannular [2+ 2] Cycloaddition/Fragmentation Approach, *J. Am. Chem. Soc.* **2022**, *144*, 15462.

[99] S. Aoyagi, T. C. Wang, C. Kibayashi, Highly stereoselective total syntheses of (+)-allopumiliotoxins 267A and 339A via intramolecular nickel (II)/chromium (II)-mediated cyclization, *J. Am. Chem. Soc.* **1993**, *115*, 11393.

[100] B. M. Trost, C. A. Kalnmals, Stereoselective synthesis of exocyclic tetrasubstituted vinyl halides via ru-catalyzed halotropic cycloisomerization of 1, 6-haloenynes, *Org. Lett.* **2017**, *19*, 2346.

[101] B. M. Trost, M. R. Machacek, H. C. Tsui, Development of aliphatic alcohols as nucleophiles for palladium-catalyzed DYKAT reactions: Total synthesis of (+)-hippospongic acid A, *J. Am. Chem. Soc.* **2005**, *127*, 7014.

[102] G. R. Heintzelman, W.-K. Fang, S. P. Keen, G. A. Wallace, S. M. Weinreb, Stereoselective total synthesis of the cyanobacterial hepatotoxin 7-Epicylindrospermopsin: revision of the stereochemistry of cylindrospermopsin, *J. Am. Chem. Soc.* **2001**, *123*, 8851.

[103] R. G. Reddy, R. Venkateshwarlu, K. V. Ramakrishna, J. S. Yadav, D. K. Mohapatra, Asymmetric Total Syntheses of Two Possible Diastereomers of Gliomasolide E and Its Structural Elucidation, *J. Org. Chem.* **2017**, *82*, 1053.

[104] P. A. Allegretti, E. M. Ferreira, Generation of α, β-unsaturated platinum carbenes from homopropargylic alcohols: rearrangements to polysubstituted furans, *Org. Lett.* **2011**, *13*, 5924.
[105] G. Biswas, J. Sengupta, M. Nath, A. Bhattacharjya, Expeditious synthesis of enantiopure symmetrical macroheterocycles by ring-closing metathesis of ether and tether-linked 1, 2-O-isopropylidenefuranosides, *Carbohydr. Res.* **2005**, *340*, 567.
[106] H. Jinnouchi, H. Nambu, T. Fujiwara, T. Yakura, Divergent synthesis of (+)-tanikolide and its analogues employing stereoselective rhodium (II)-catalyzed reaction, *Tetrahedron* **2018**, *74*, 1059.
[107] D. L. Clive, M. Yu, M. Sannigrahi, Synthesis of optically pure (+)-puraquinonic acid and assignment of absolute configuration to natural (−)-puraquinonic acid. Use of radical cyclization for asymmetric generation of a quaternary center, *J. Org. Chem.* **2004**, *69*, 4116.
[108] H. Clavier, J. Broggi, S. P. Nolan, Ring-Rearrangement Metathesis (RRM) Mediated by Ruthenium-Indenylidene Complexes, *Eur. J. Org. Chem.* **2010**, *2010*, 937.
[109] Y. Wang, T. R. Hoye, Intramolecular Capture of HDDA-Derived Benzynes: (i) 6- to 12-Membered Ring Formation, (ii) Internally (vis-à-vis Remotely) Tethered Traps, and (iii) Role of the Rate of Trapping by the Benzynophile, *Org. Lett.* **2018**, *20*, 88.
[110] K. Nicolaou, W. E. Brenzovich, P. G. Bulger, T. M. Francis, Synthesis of iso-epoxy-amphidinolide N and des-epoxy-caribenolide I structures. Initial forays, *Org. Biomol. Chem.* **2006**, *4*, 2119.
[111] M. J. Frisch, G. W. Trucks, H. B. Schlegel, G. E. Scuseria, M. A. Robb, J. R. Cheeseman, G. Scalmani, V. Barone, G. A. Petersson, H. Nakatsuji, X. Li, M. Caricato, A. V. Marenich, J. Bloino, B. G. Janesko, R. Gomperts, B. Mennucci, H. P. Hratchian, J. V. Ortiz, A. F. Izmaylov, J. L. Sonnenberg, Williams, F. Ding, F. Lipparini, F. Egidi, J. Goings, B. Peng, A. Petrone, T. Henderson, D. Ranasinghe, V. G. Zakrzewski, J. Gao, N. Rega, G. Zheng, W. Liang, M. Hada, M. Ehara, K. Toyota, R. Fukuda, J. Hasegawa, M. Ishida, T. Nakajima, Y. Honda, O. Kitao, H. Nakai, T. Vreven, K. Throssell, J. A. Montgomery Jr., J. E. Peralta, F. Ogliaro, M. J. Bearpark, J. J. Heyd, E. N. Brothers, K. N. Kudin, V. N. Staroverov, T. A. Keith, R. Kobayashi, J. Normand, K. Raghavachari, A. P. Rendell, J. C. Burant, S. S. Iyengar, J. Tomasi, M. Cossi, J. M. Millam, M. Klene, C. Adamo, R. Cammi, J. W. Ochterski, R. L. Martin, K. Morokuma, O. Farkas, J. B. Foresman, D. J. Fox, Wallingford, CT, **2016**.
[112] V. Barone, M. Cossi, Quantum calculation of molecular energies and energy gradients in solution by a conductor solvent model, *J. Phys. Chem. A* **1998**, *102*, 1995.
[113] M. Cossi, N. Rega, G. Scalmani, V. Barone, Energies, structures, and electronic properties of molecules in solution with the C-PCM solvation model, *J. Comput. Chem.* **2003**, *24*, 669.
[114] T. Lu, Q. Chen, Shermo: A general code for calculating molecular thermochemistry properties, *Computational and Theoretical Chemistry* **2021**, *1200*.
[115] A. A. Otlyotov, Y. Minenkov, Gas-phase thermochemistry of noncovalent ligand-alkali metal ion clusters: An impact of low frequencies, *J. Comput. Chem.* **2023**, *44*, 1807.
[116] S. Grimme, Supramolecular Binding Thermodynamics by Dispersion-Corrected Density Functional Theory, *Chemistry-a European Journal* **2012**, *18*, 9955.
[117] A. V. Marenich, C. J. Cramer, D. G. Truhlar, Universal Solvation Model Based on Solute Electron Density and on a Continuum Model of the Solvent Defined by the Bulk Dielectric Constant and Atomic Surface Tensions, *J. Phys. Chem. B* **2009**, *113*, 6378.
[118] T. Lu, F. Chen, Multiwfn: A multifunctional wavefunction analyzer, *J. Comput. Chem.* **2012**, *33*, 580.
[119] T. Lu, Q. Chen, Independent gradient model based on Hirshfeld partition: A new method for visual study of interactions in chemical systems, *J. Comput. Chem.* **2022**, *43*, 539.
[120] T. Lu, A comprehensive electron wavefunction analysis toolbox for chemists, Multiwfn, *J. Chem. Phys.* **2024**, *161*.

S1a, $CDCl_3$, 400 MHz

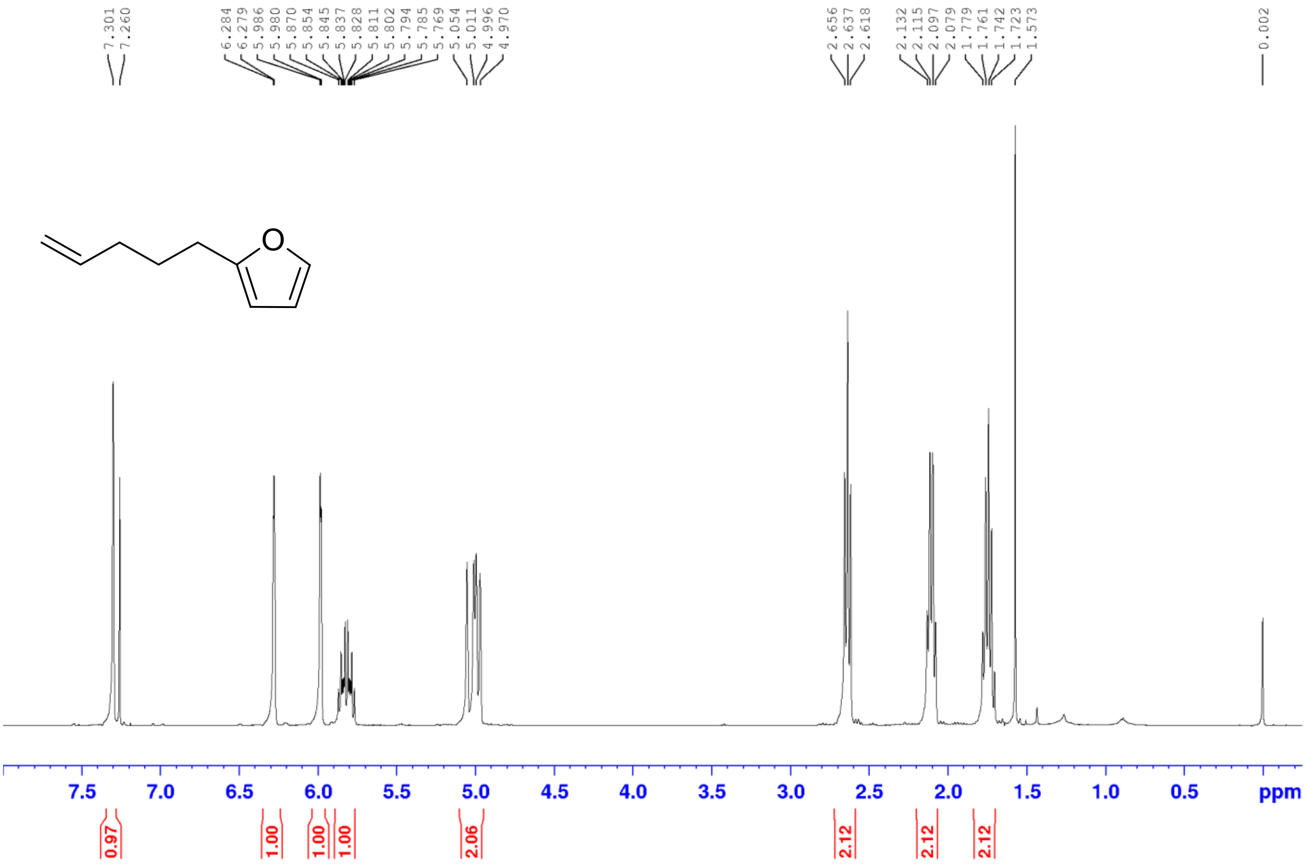

S1a, $CDCl_3$, 100 MHz

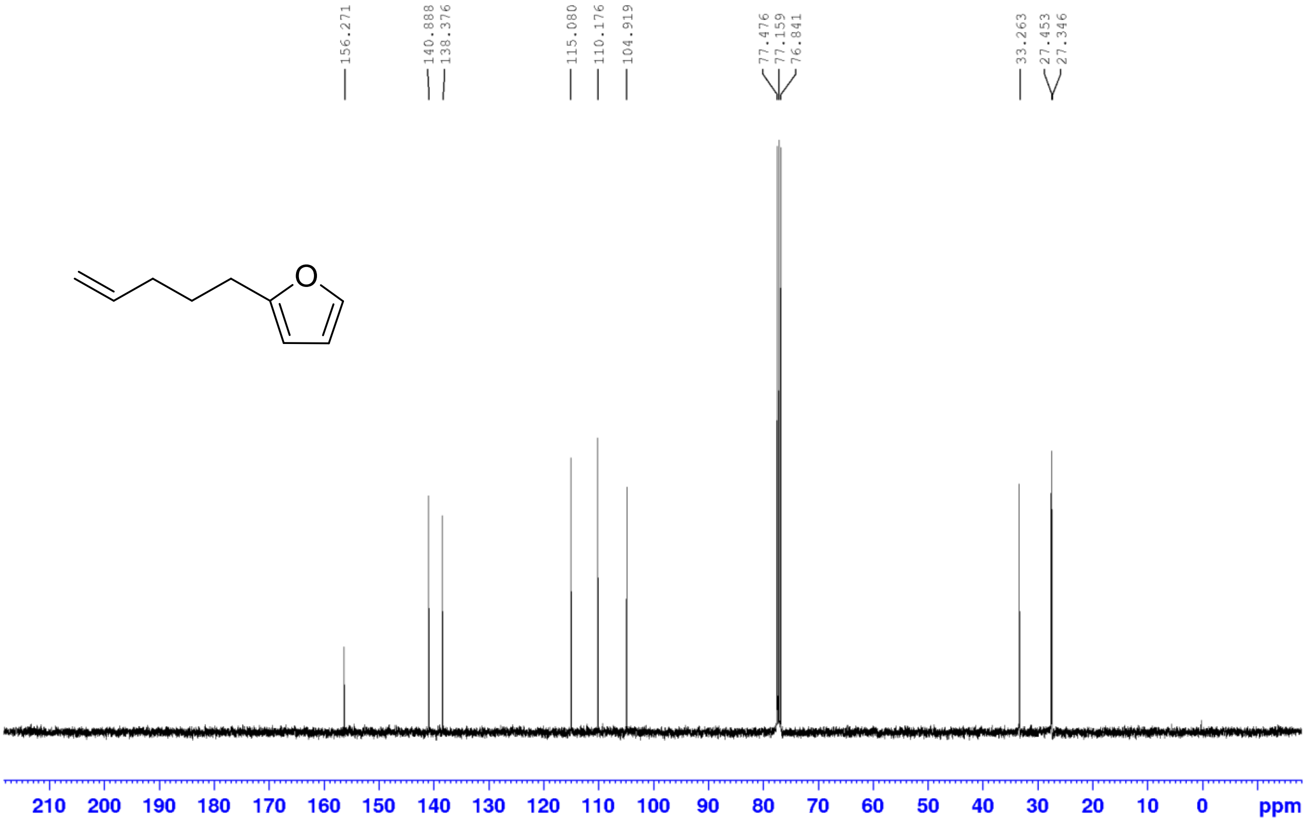

S2a, $CDCl_3$, 400 MHz

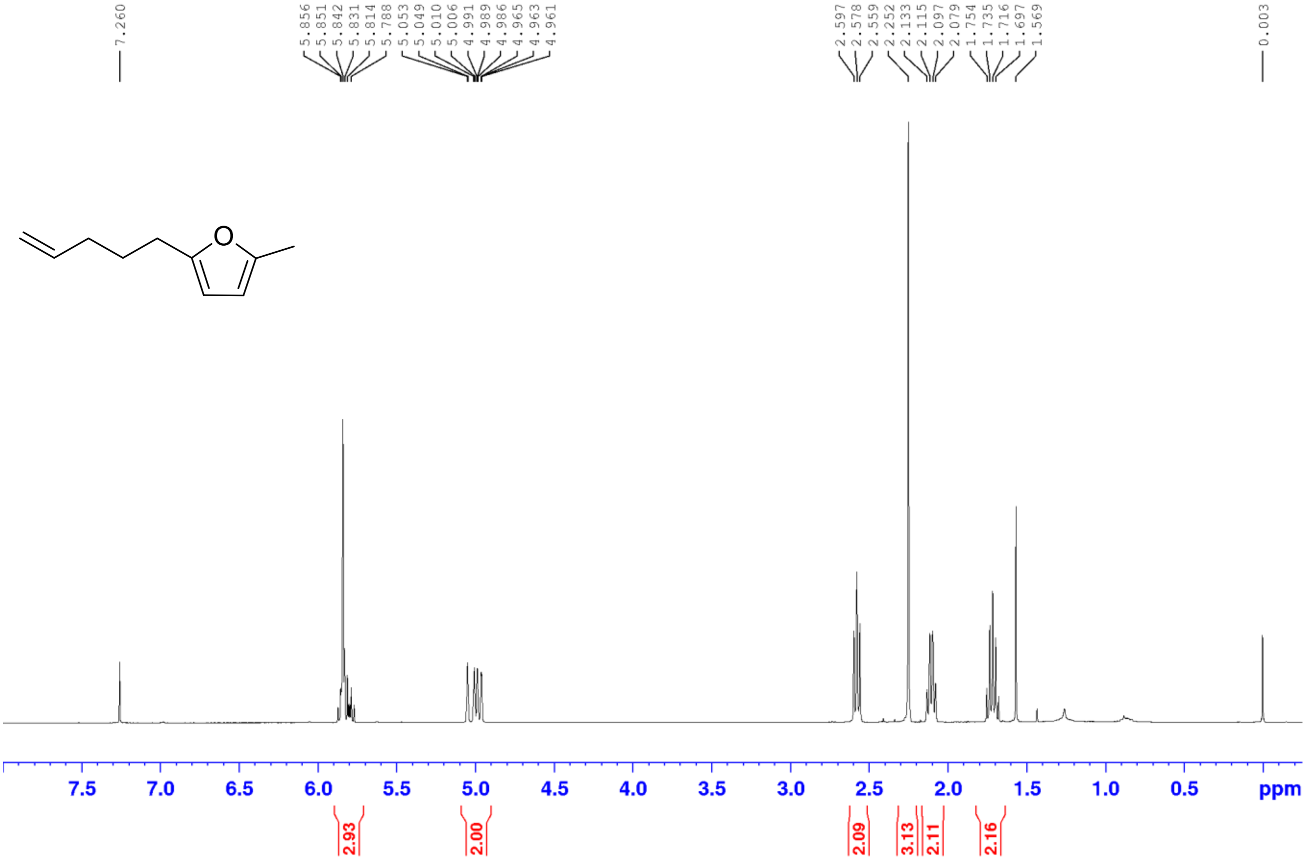

S1f, C_6D_6, 400 MHz

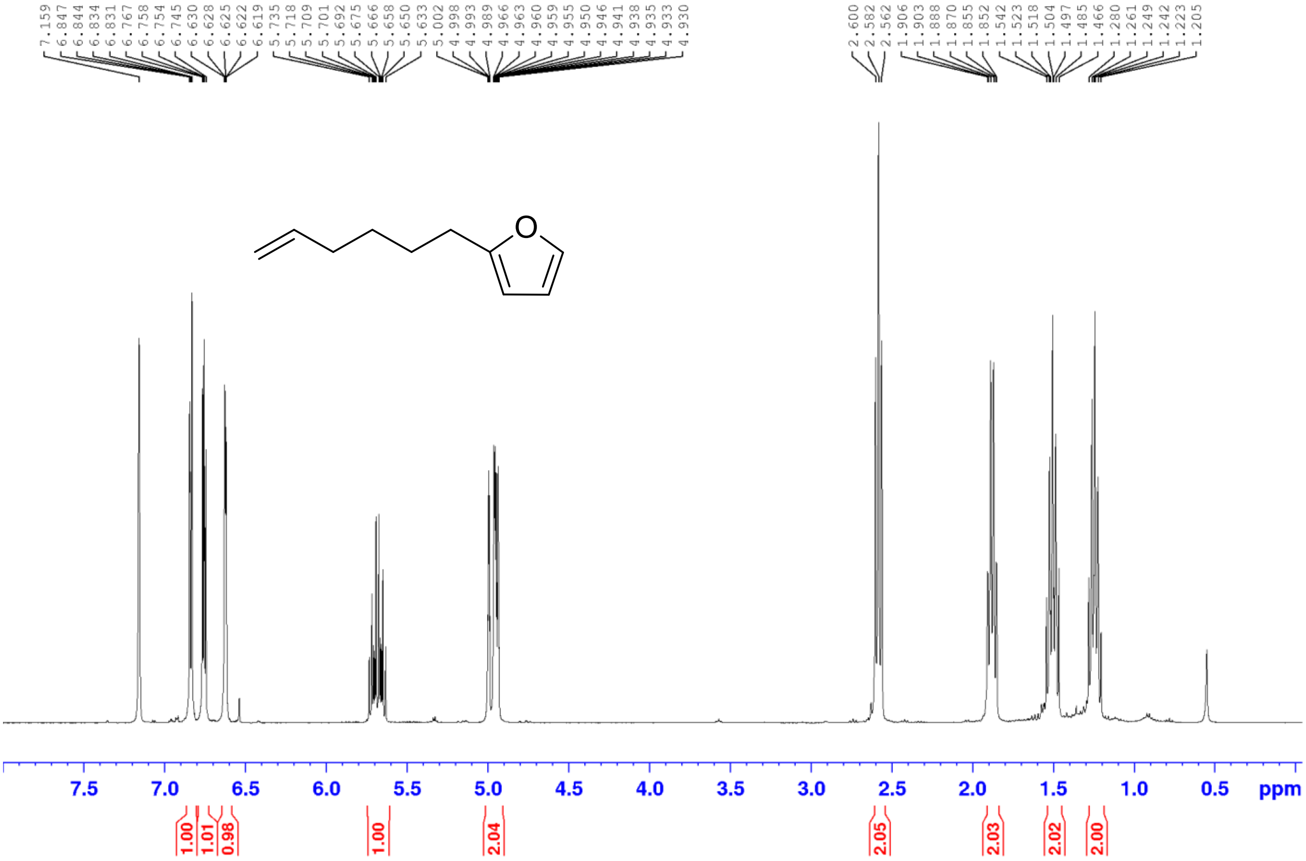

S1f, C_6D_6, 100 MHz

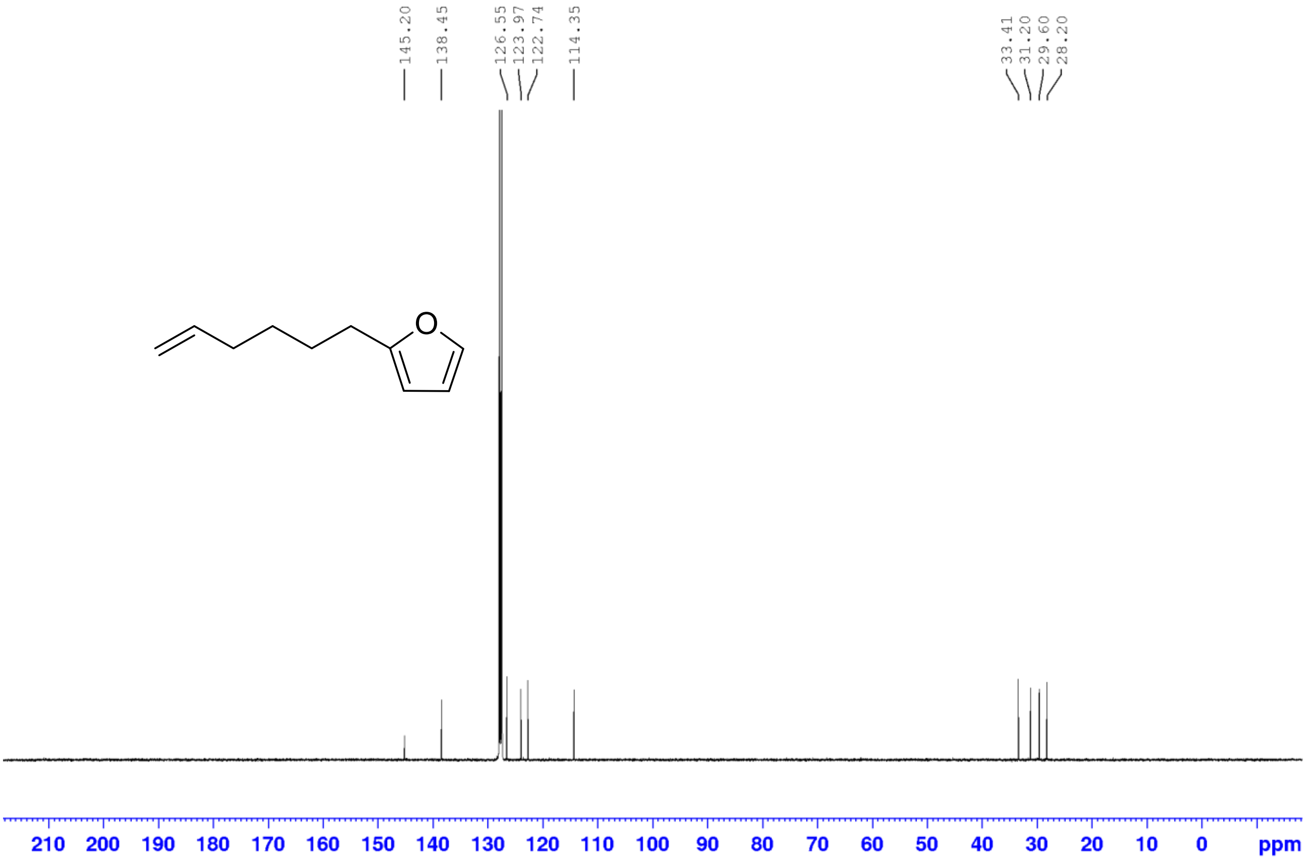

S1b, C_6D_6, 400 MHz

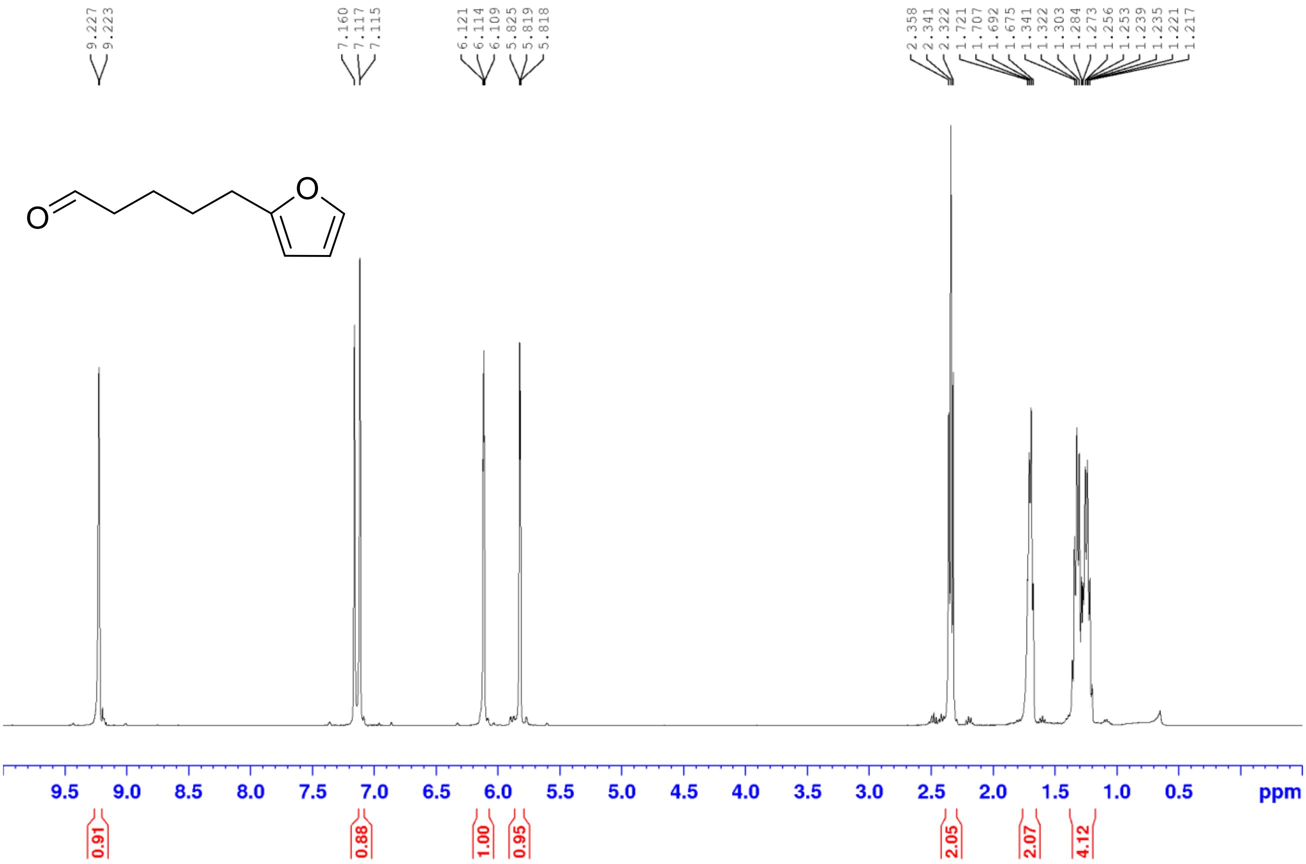

S1b, C_6D_6, 100 MHz

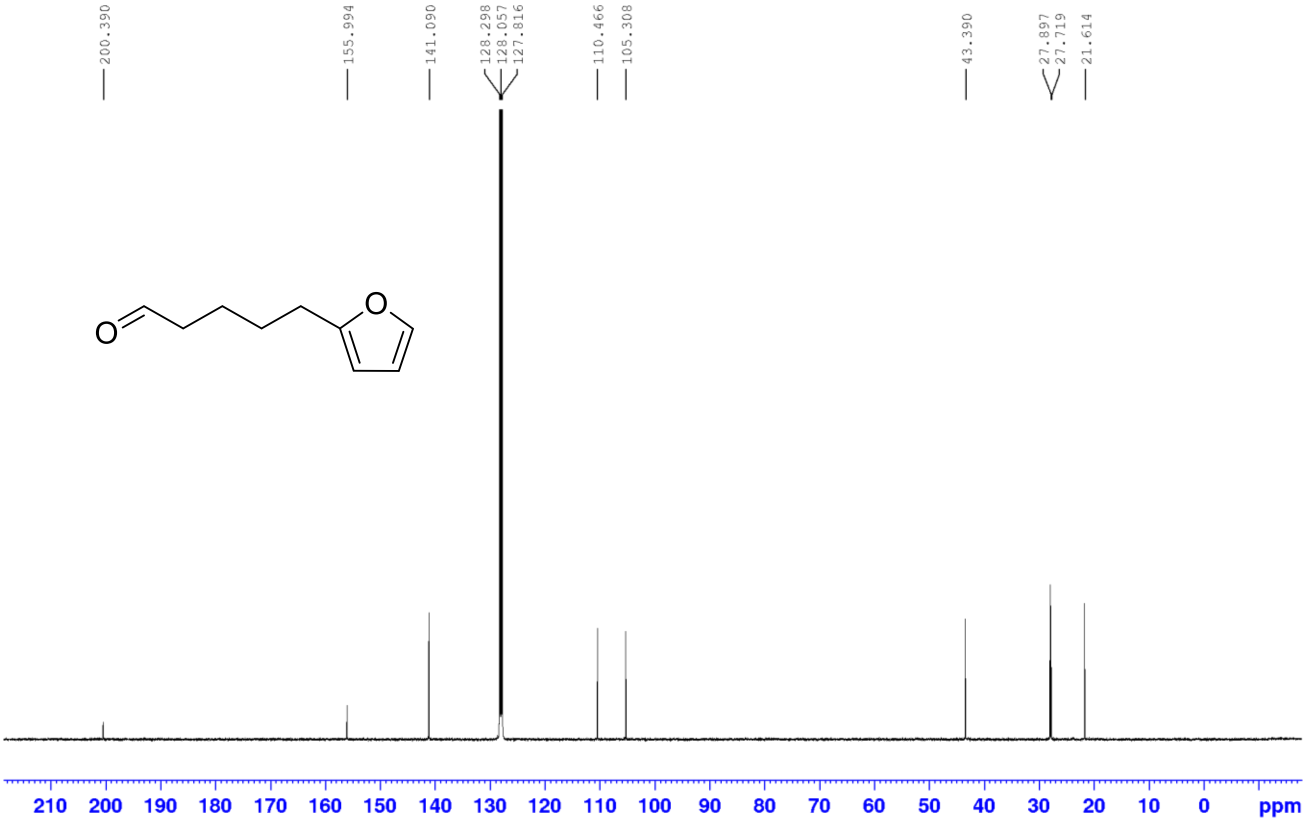

S1g, C_6D_6, 400 MHz

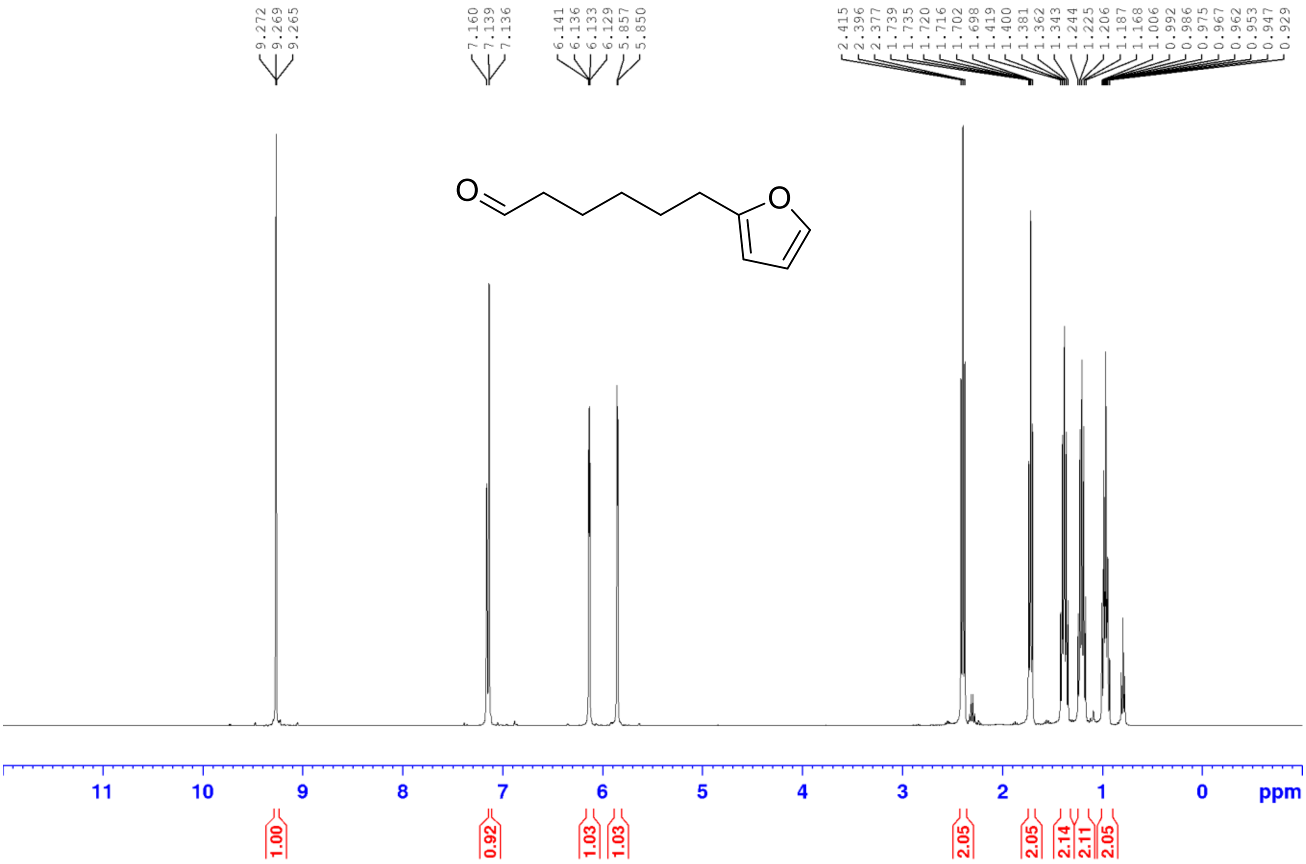

S1g, C_6D_6, 100 MHz

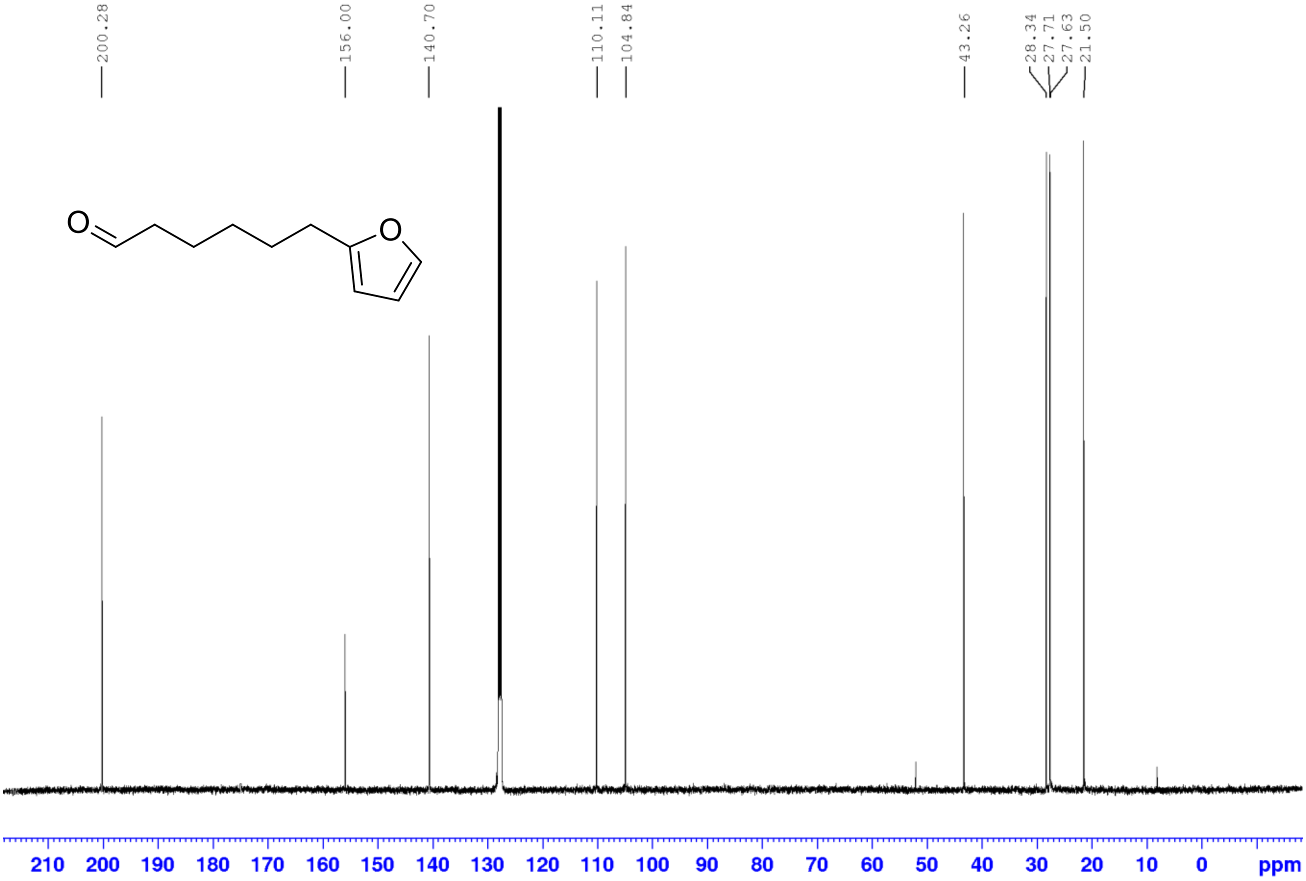

S1c, C_6D_6, 400 MHz

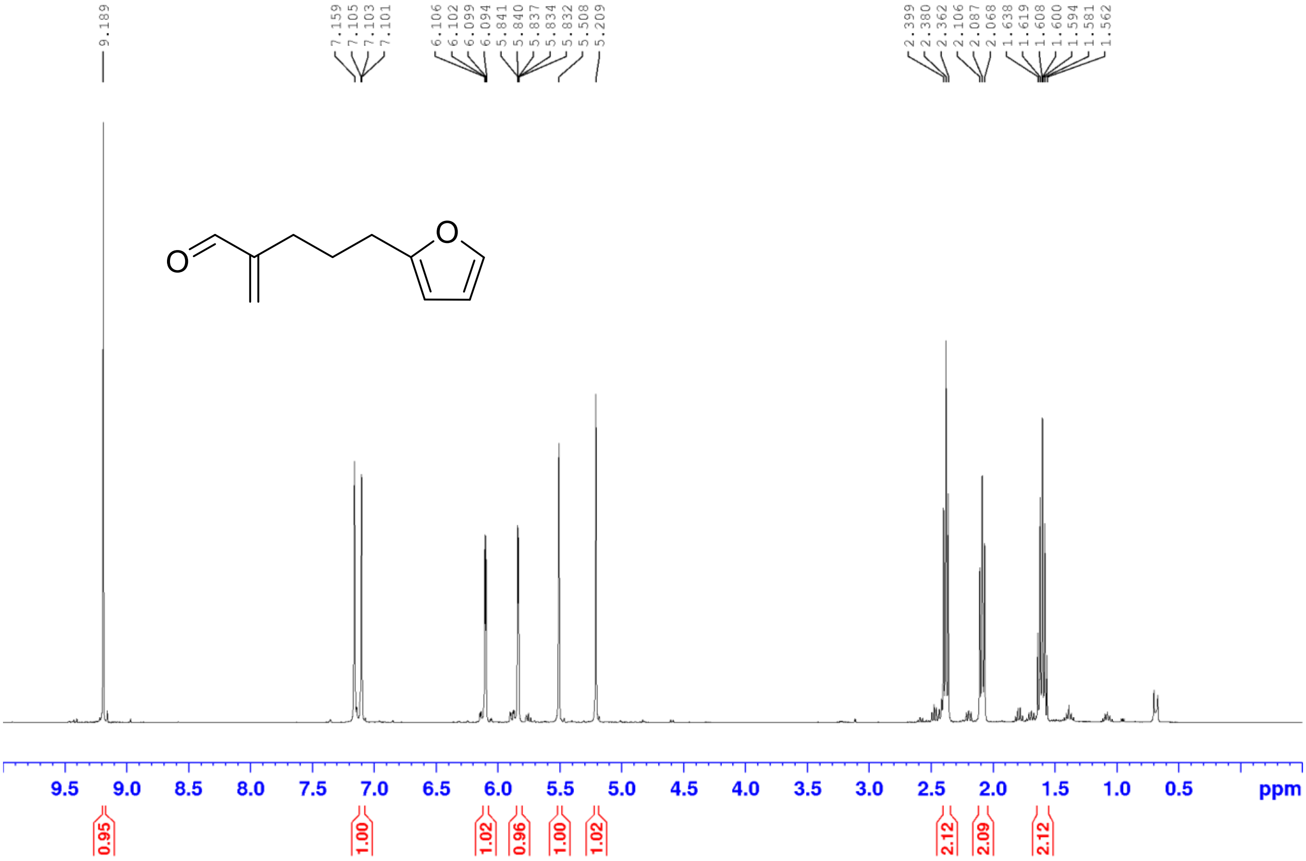

S1c, C_6D_6, 100 MHz

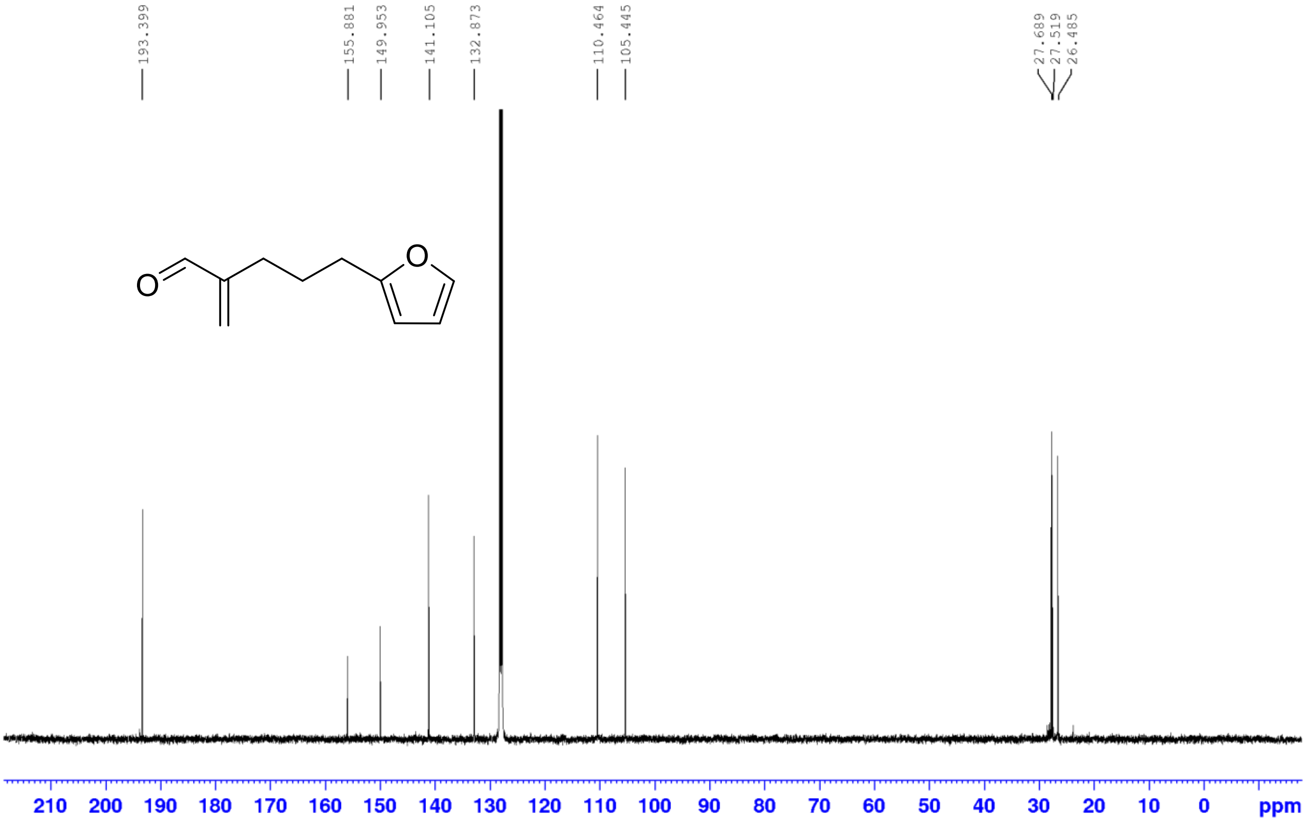

S2c, $CDCl_3$, 400 MHz

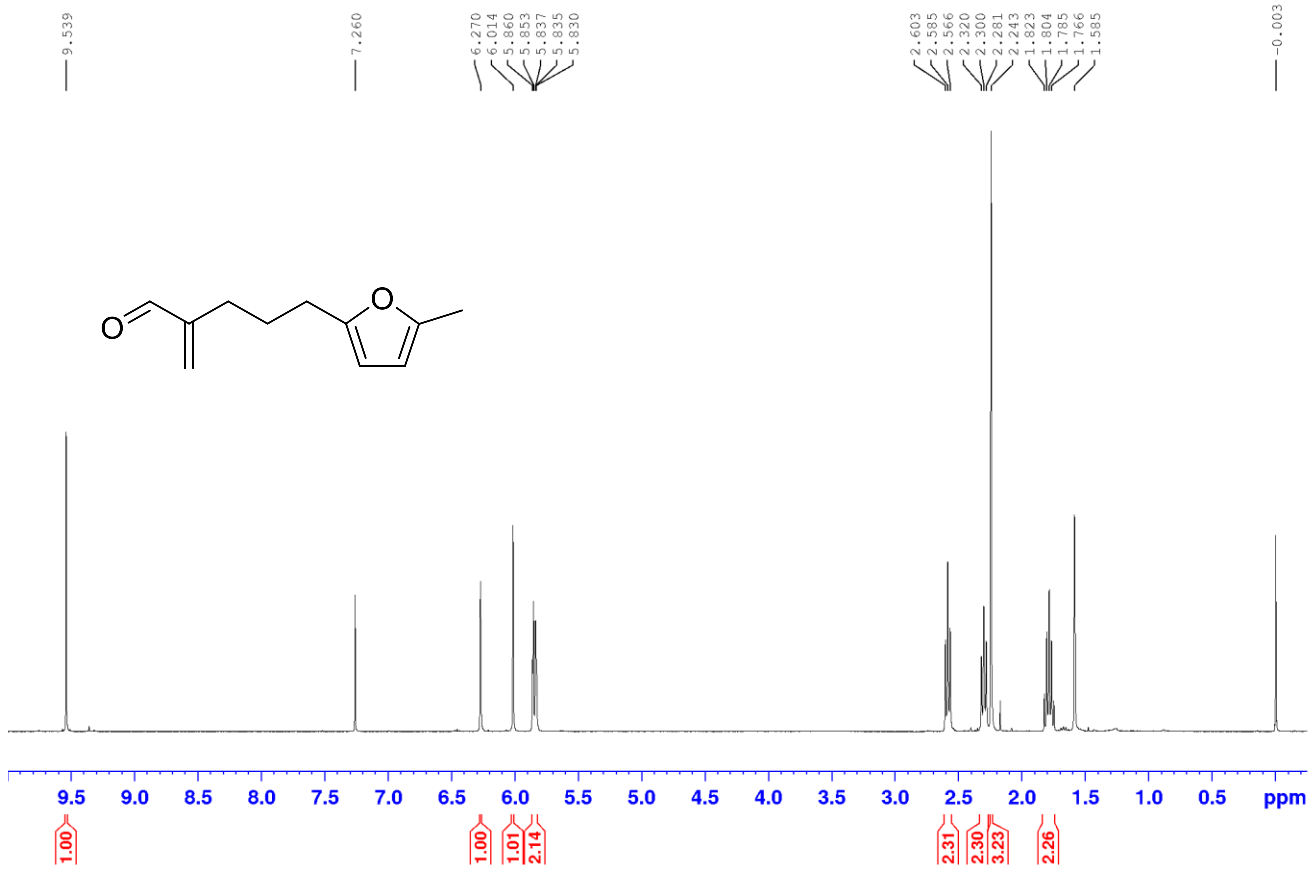

S2c, $CDCl_3$, 100 MHz

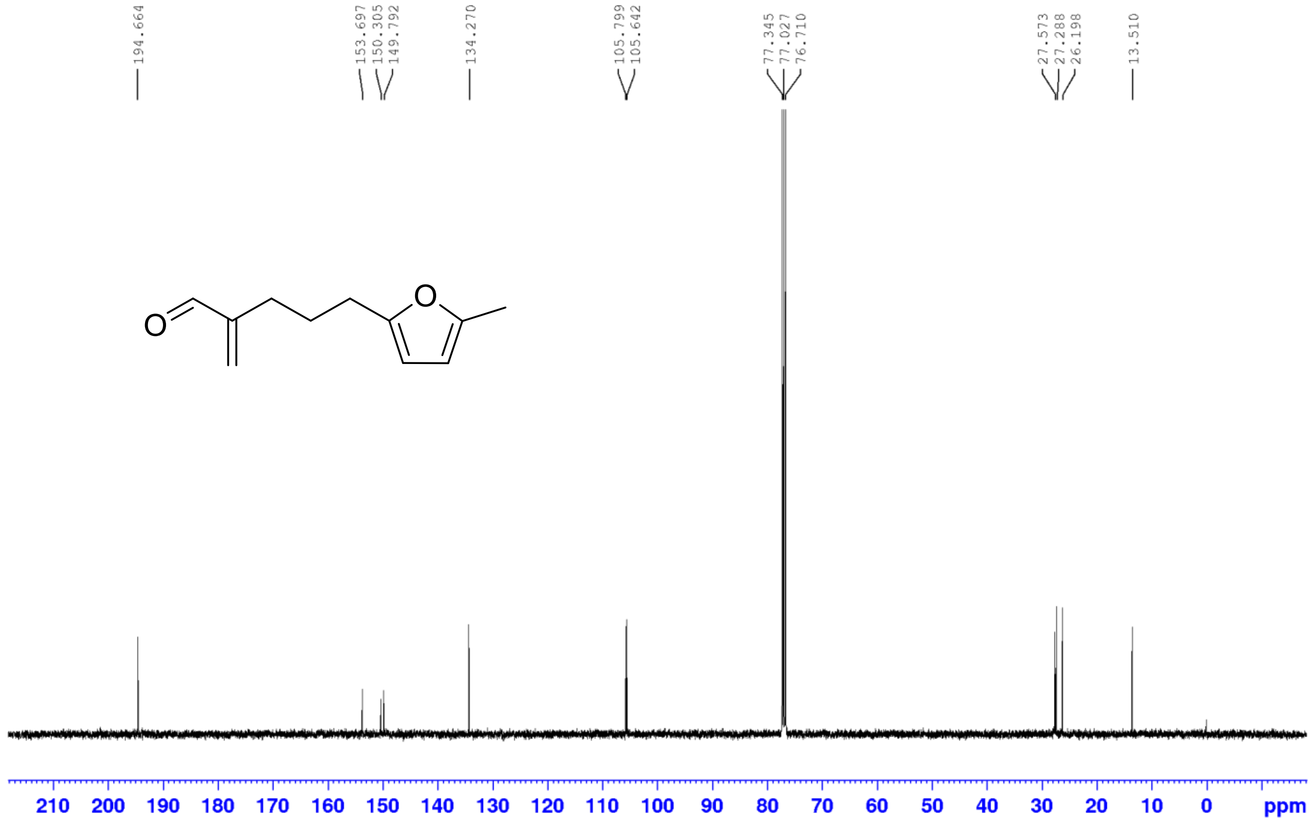

S3c, C_6D_6, 400 MHz

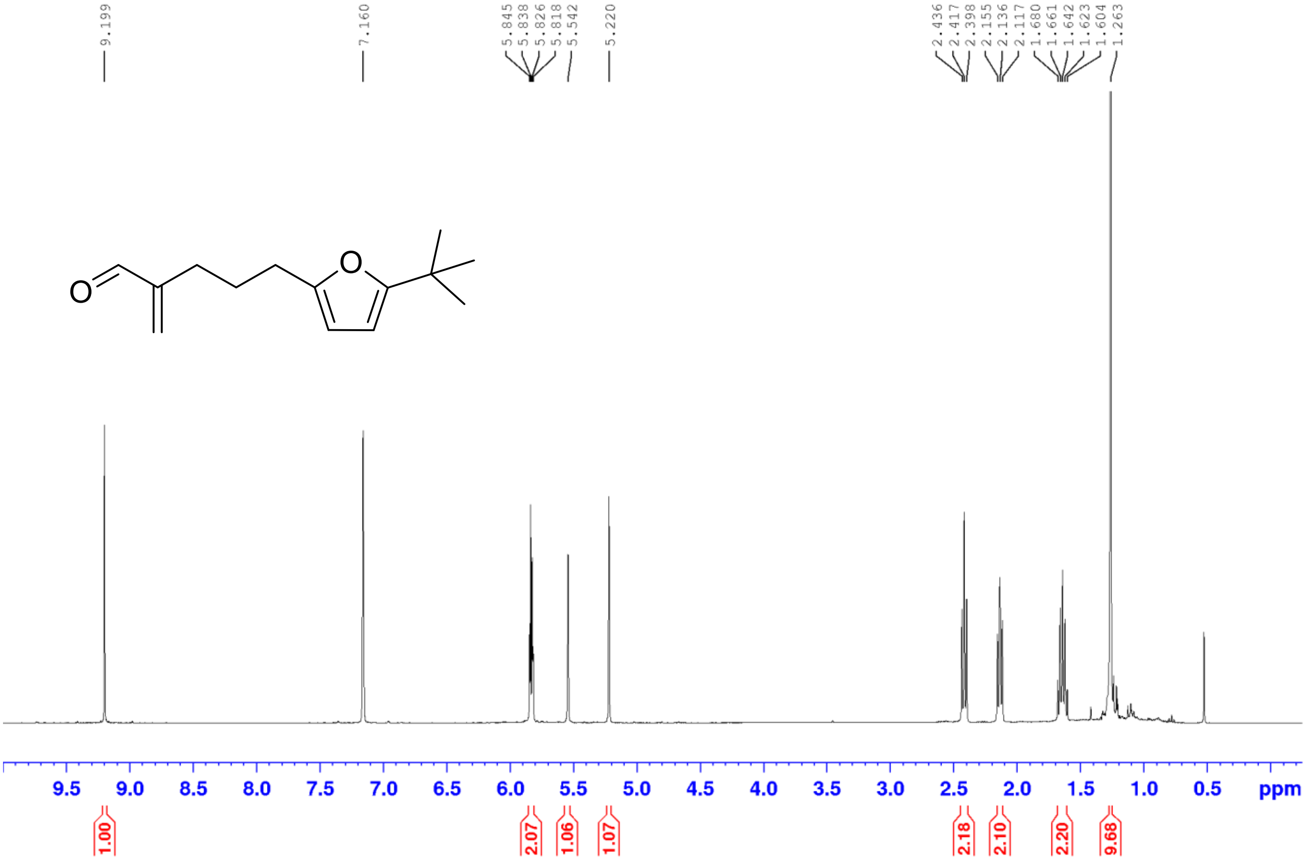

S3c, C_6D_6, 100 MHz

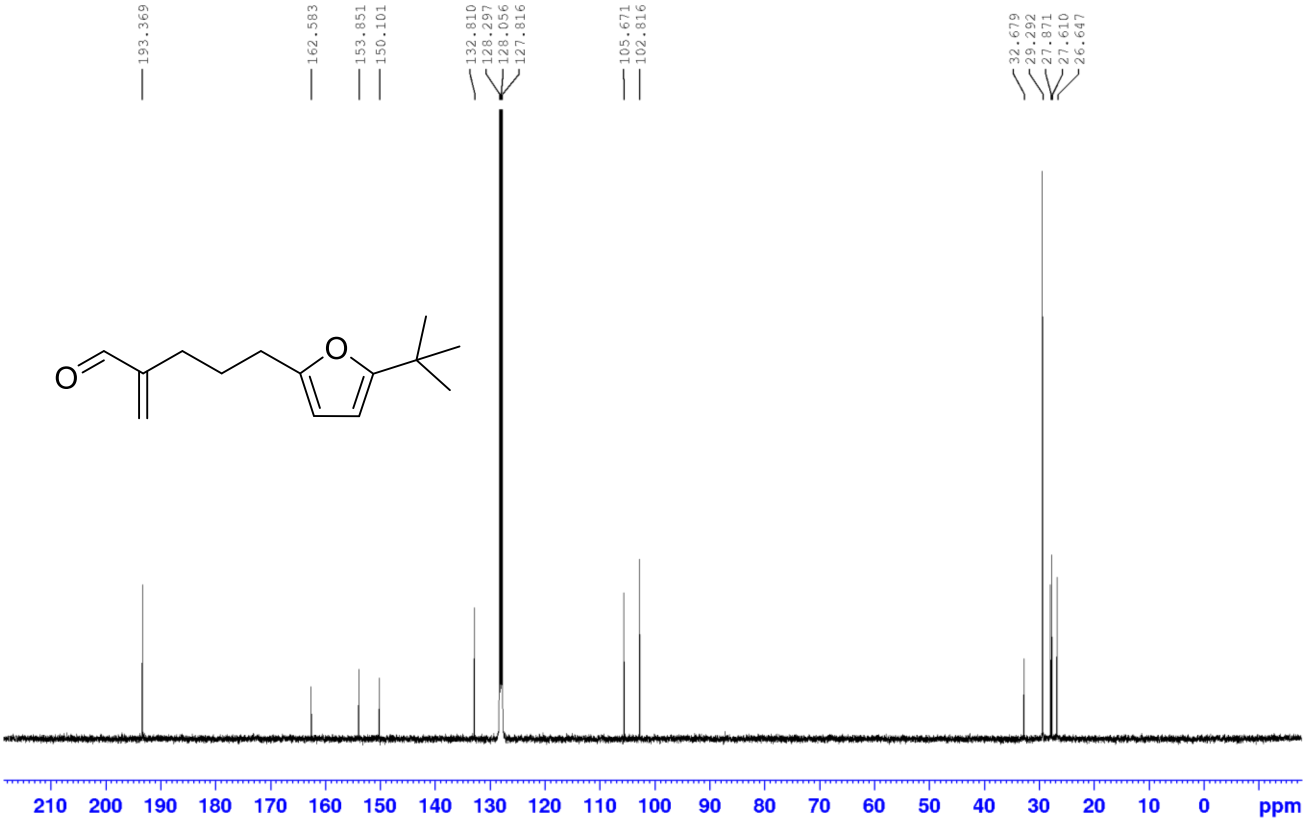

S1d, C_6D_6, 400 MHz

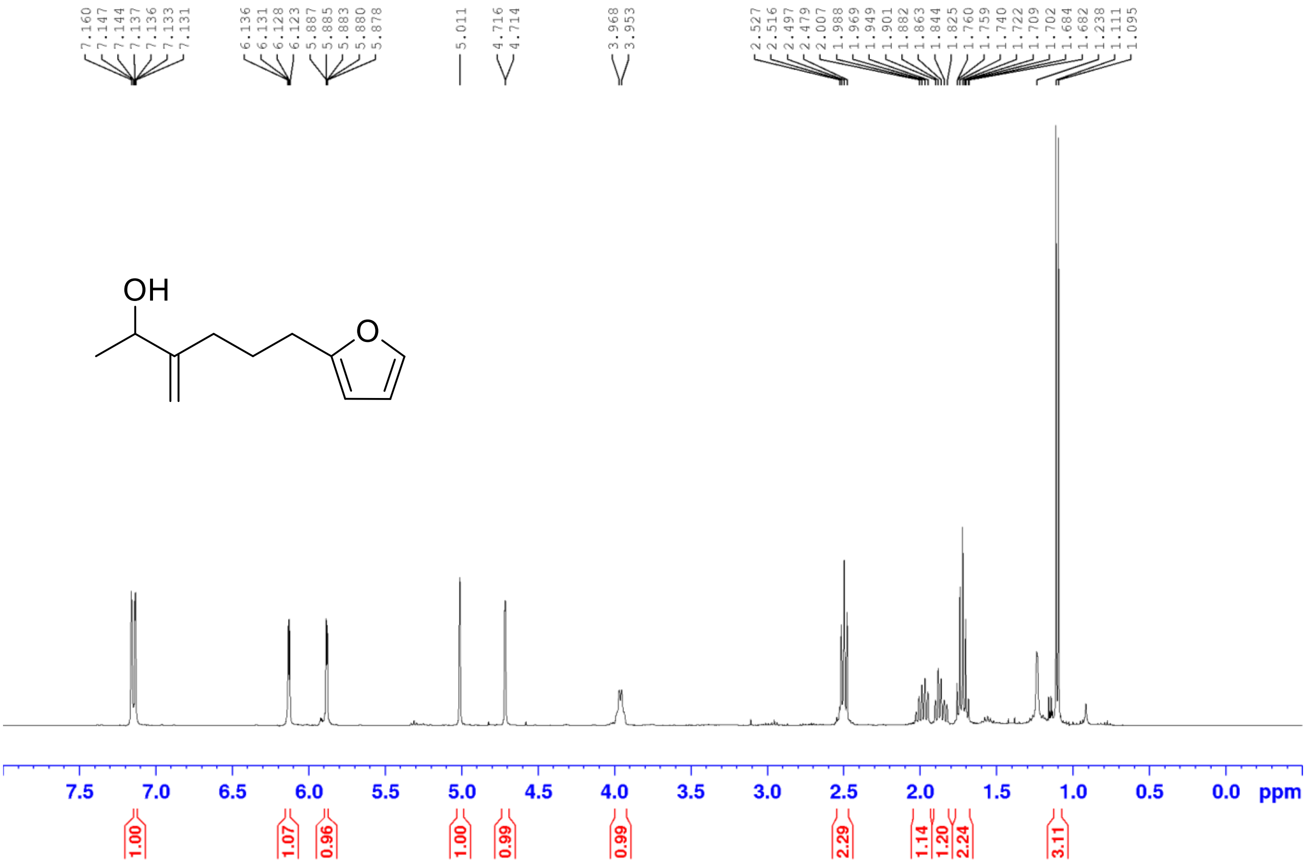

S1d, C_6D_6, 100 MHz

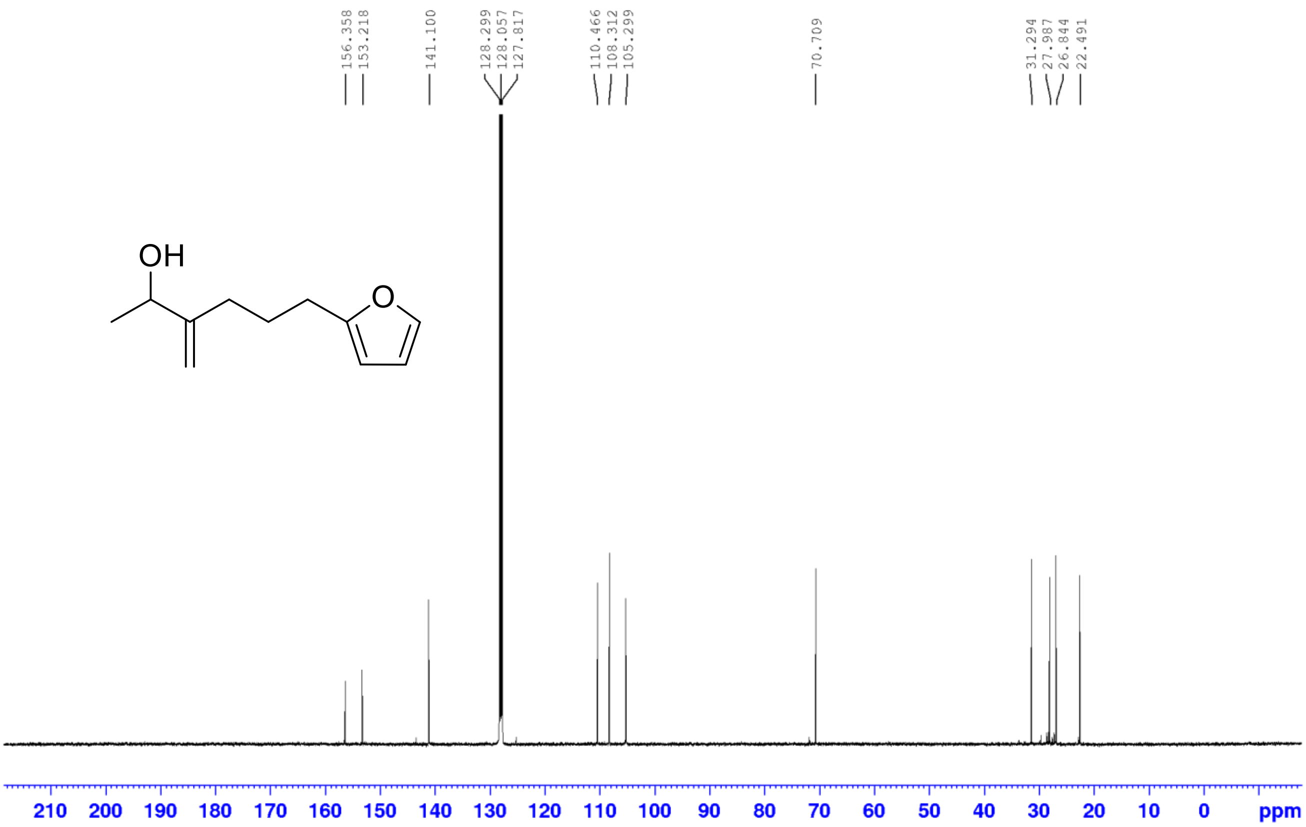

S2d, $CDCl_3$, 400 MHz

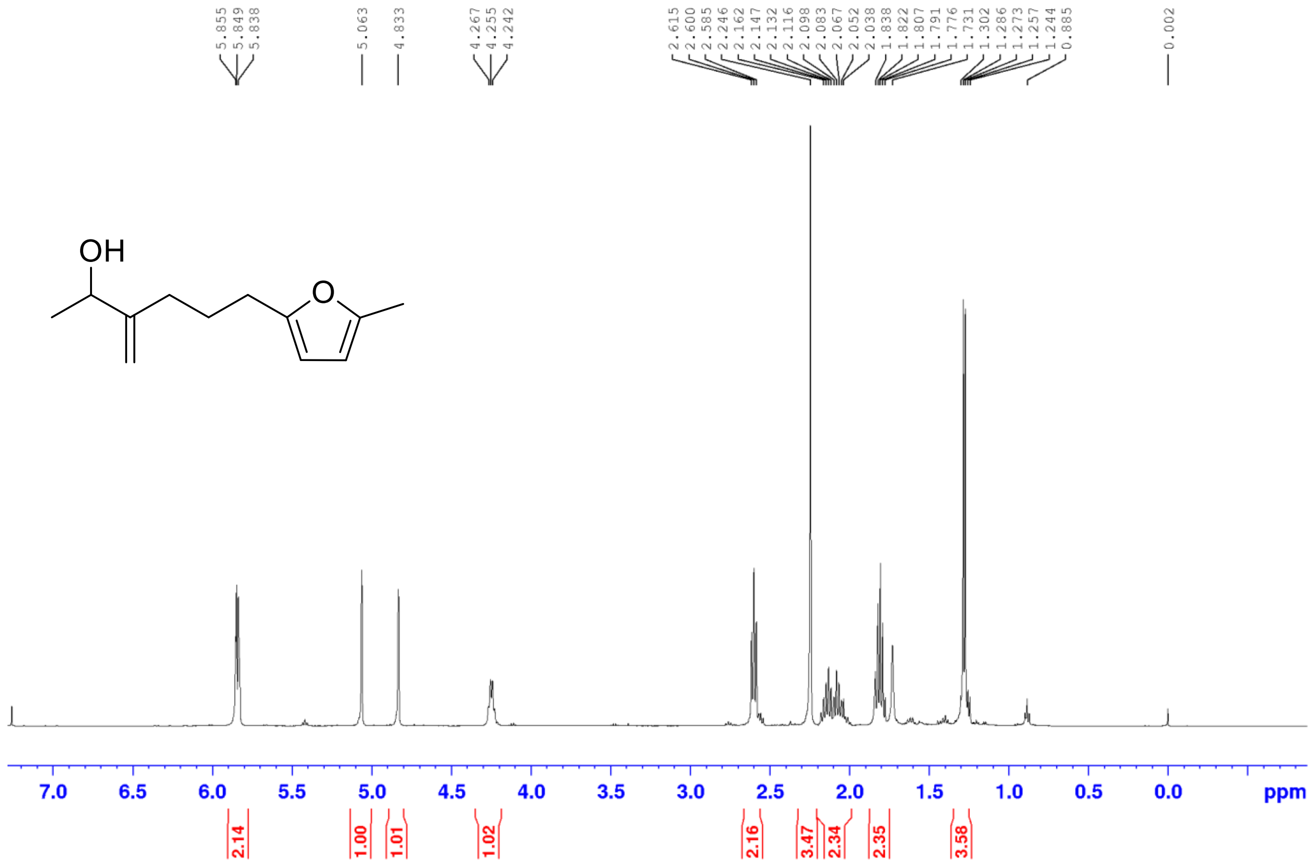

S2d, $CDCl_3$, 100 MHz

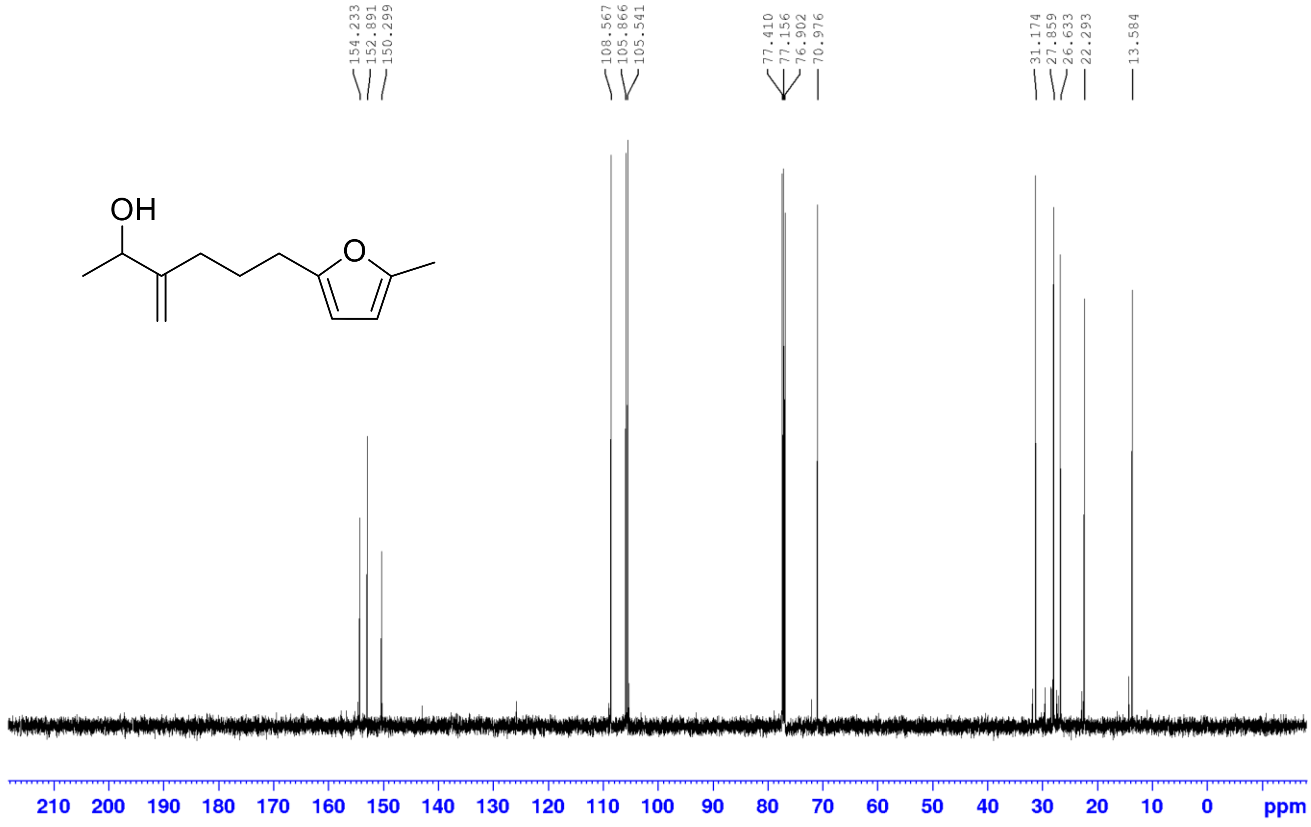

S2e, $CDCl_3$, 400 MHz

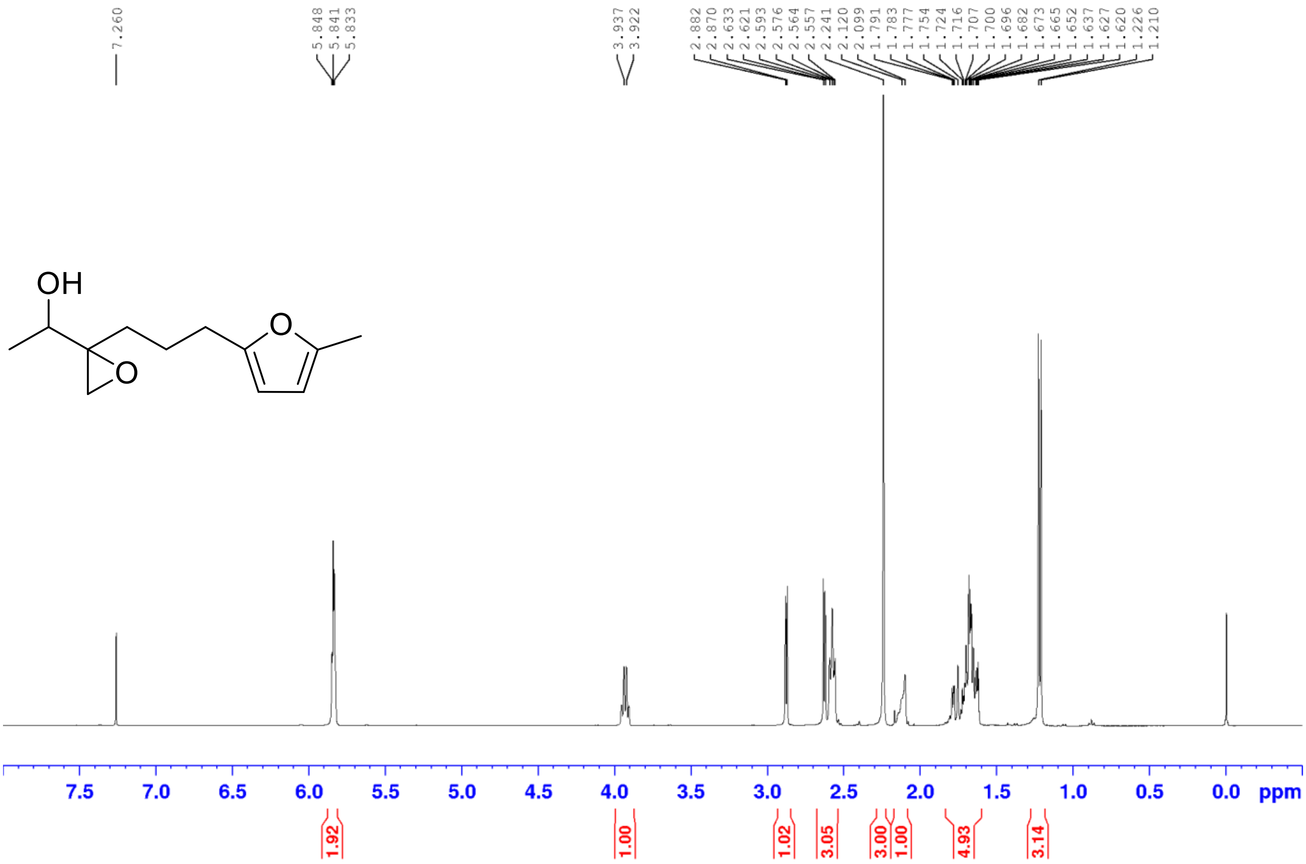

S2e, $CDCl_3$, 100 MHz

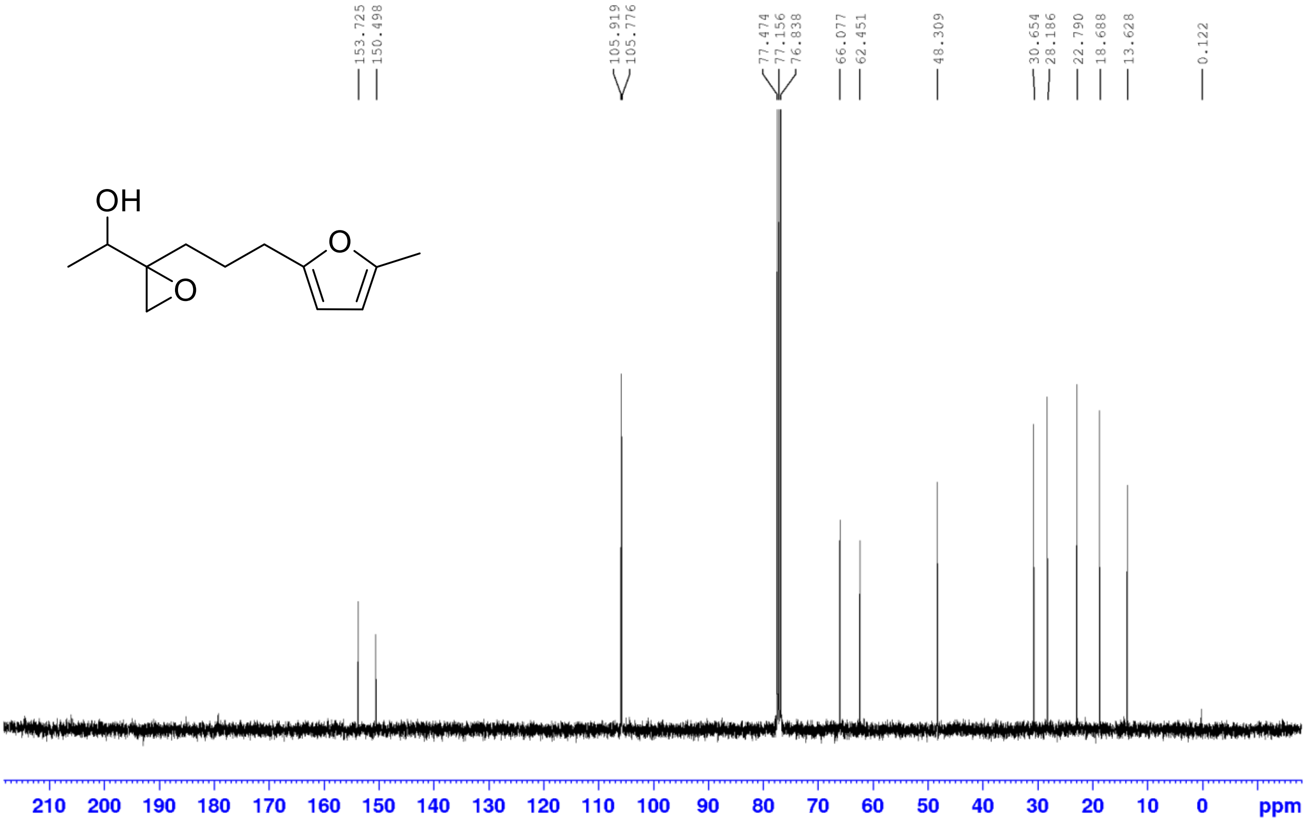

S1j, $CDCl_3$, 400 MHz

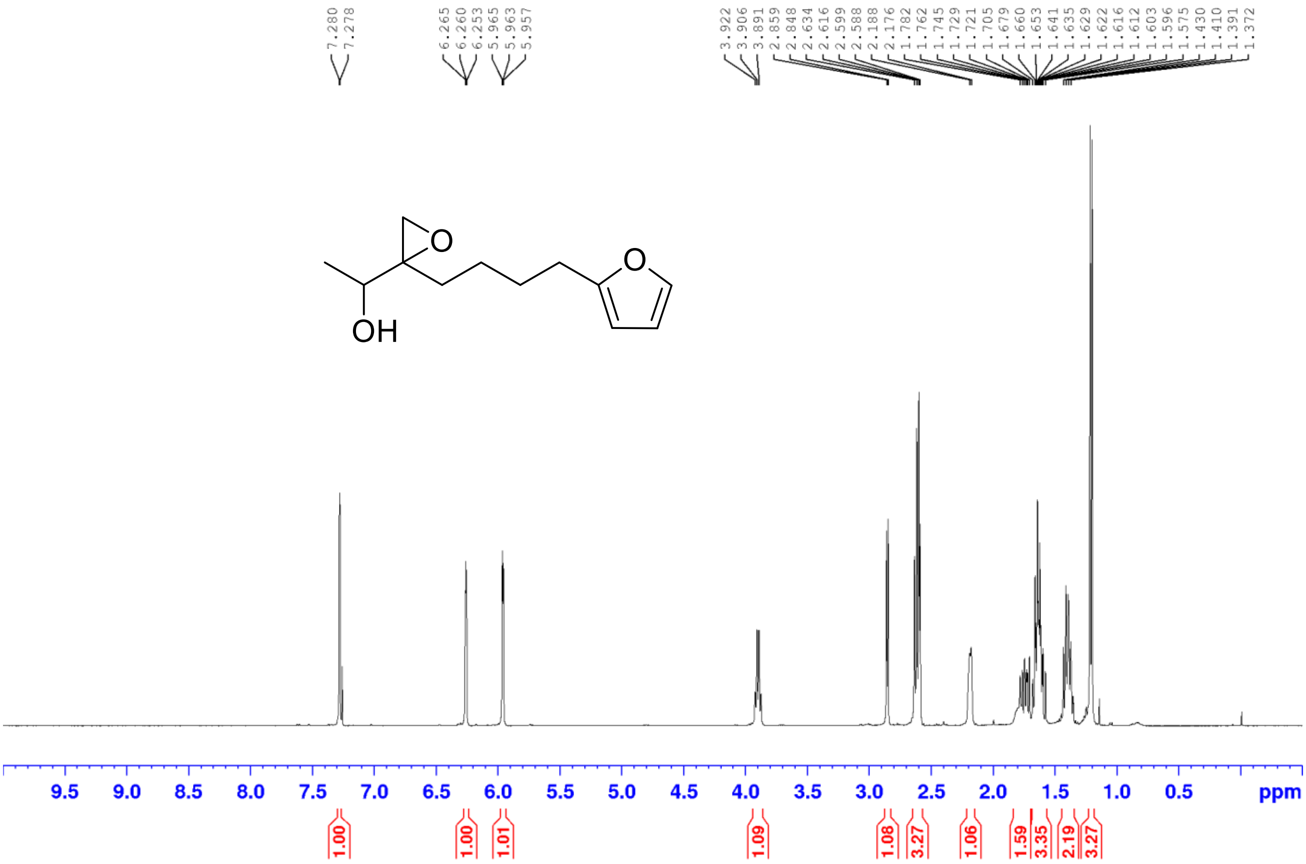

S1j, $CDCl_3$, 100 MHz

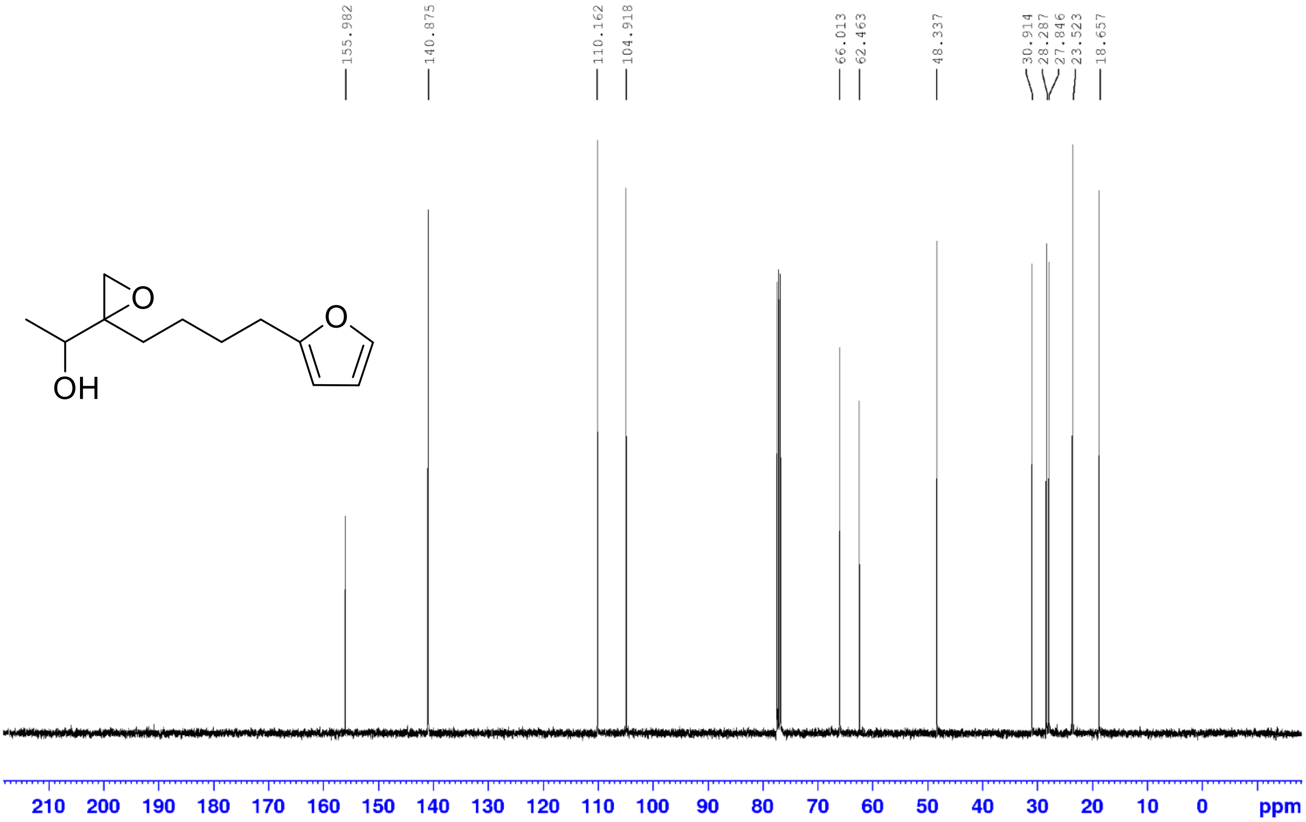

11, C_6D_6, 400 MHz

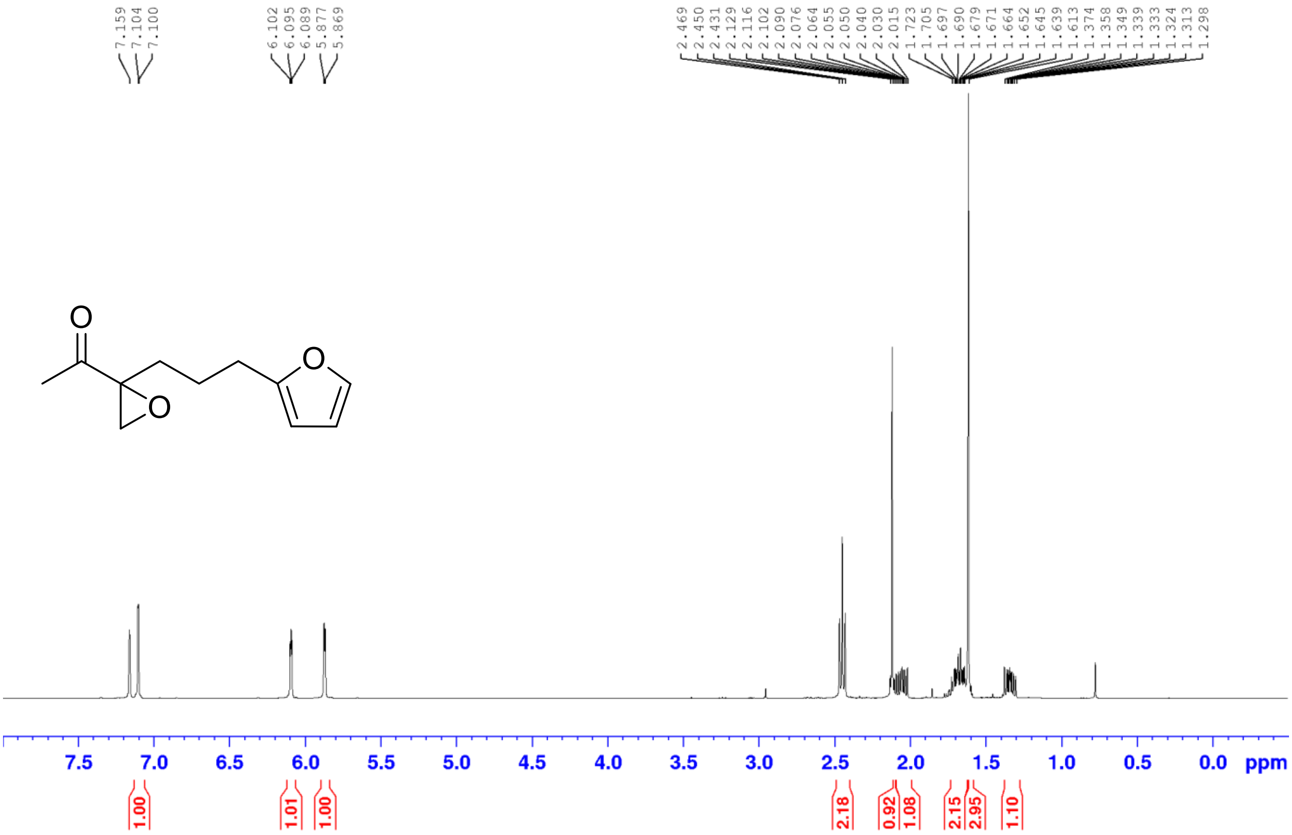

11, C_6D_6, 100 MHz

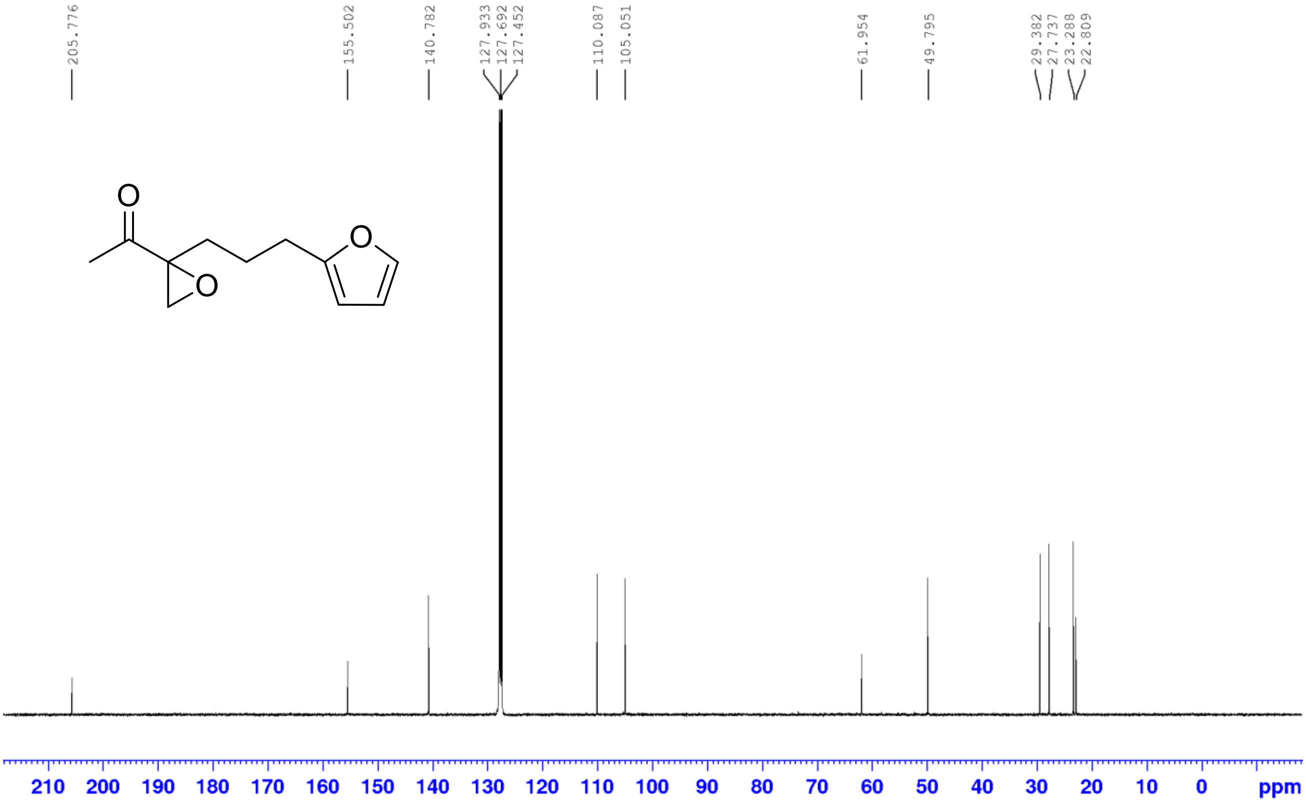

12, $CDCl_3$, 600 MHz

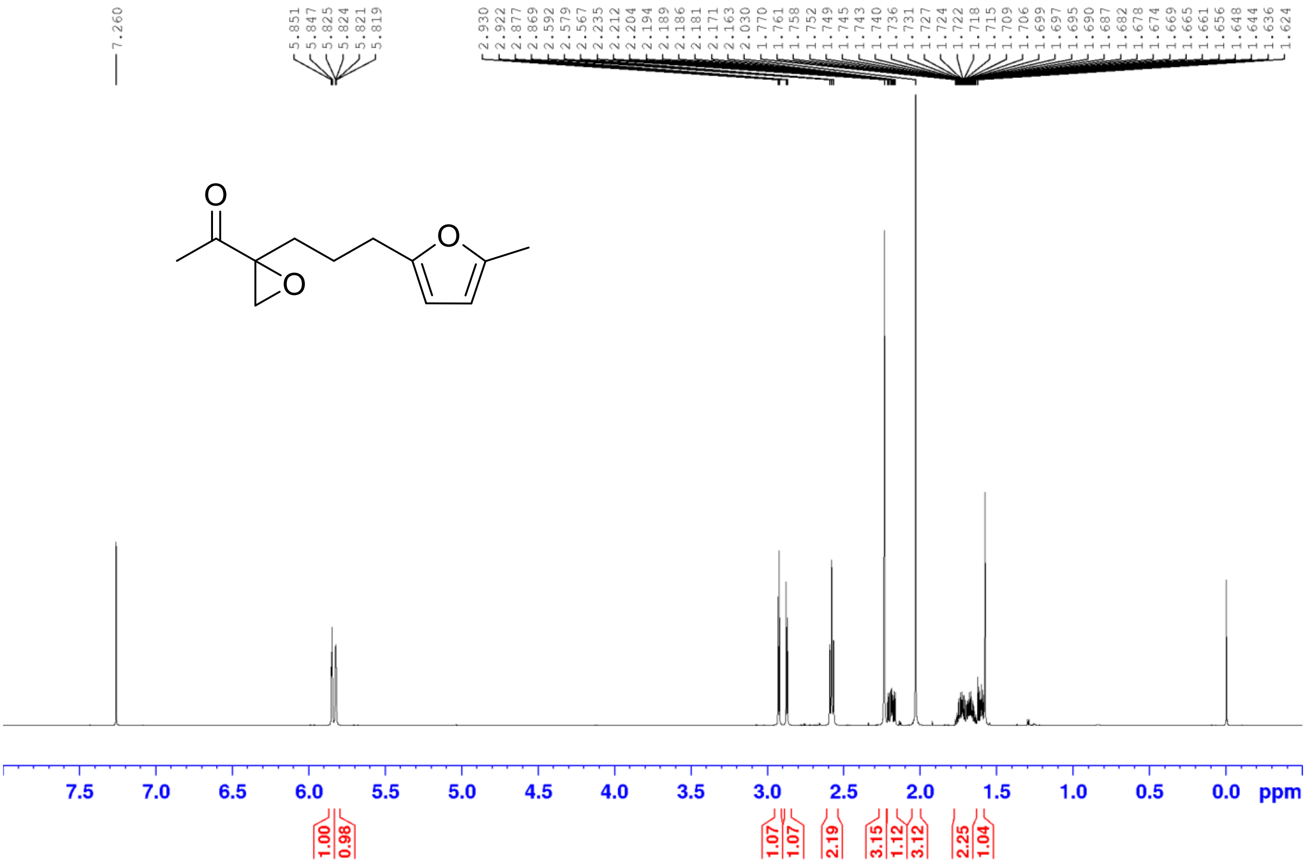

12, $CDCl_3$, 151 MHz

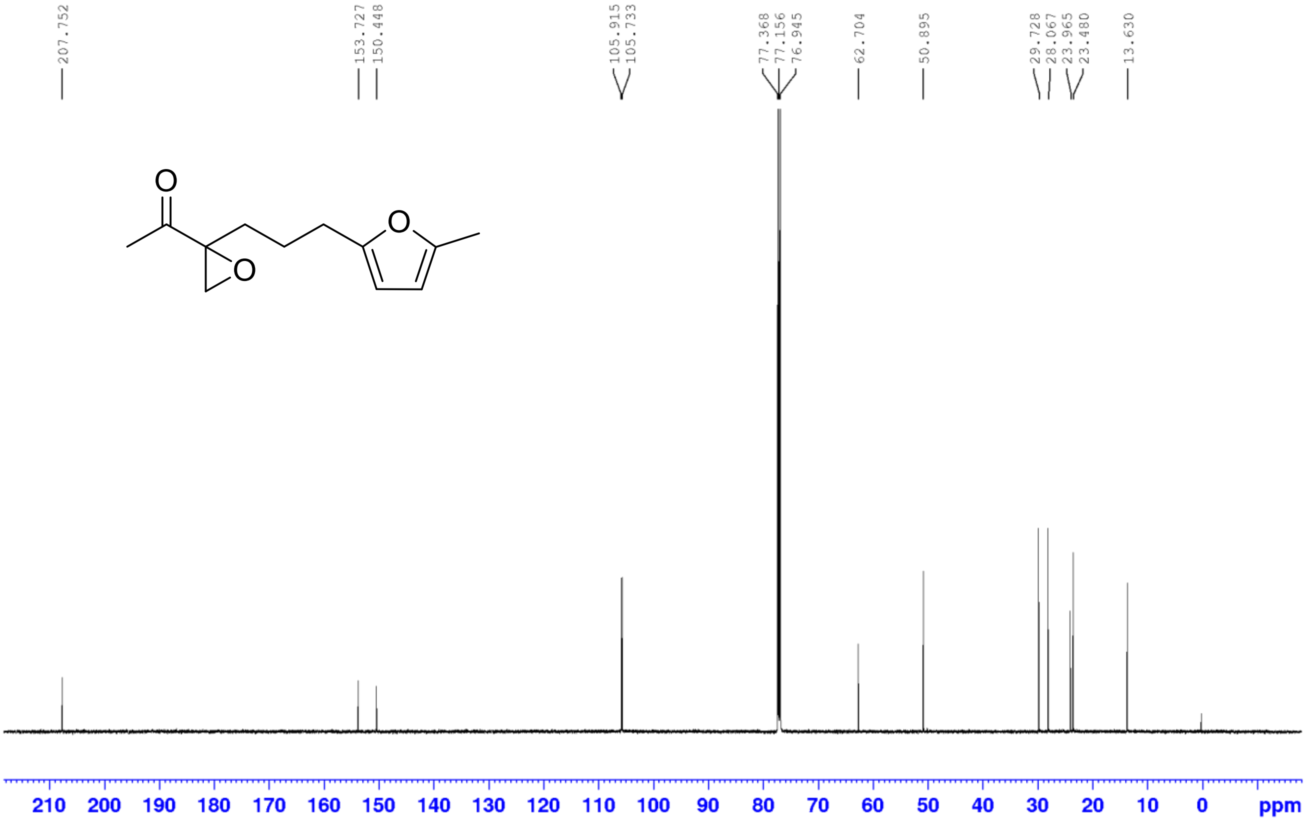

13, C_6D_6, 400 MHz

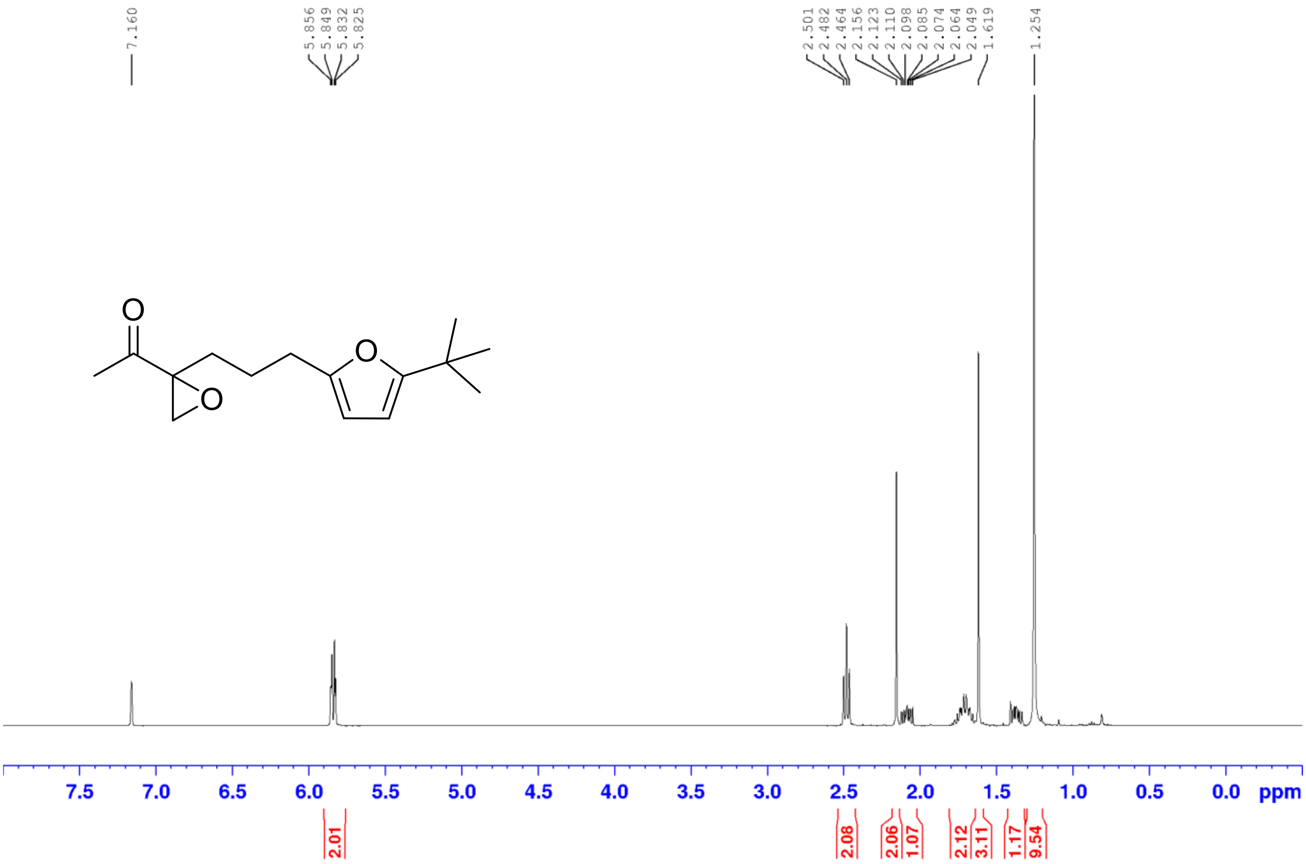

13, C_6D_6, 100 MHz

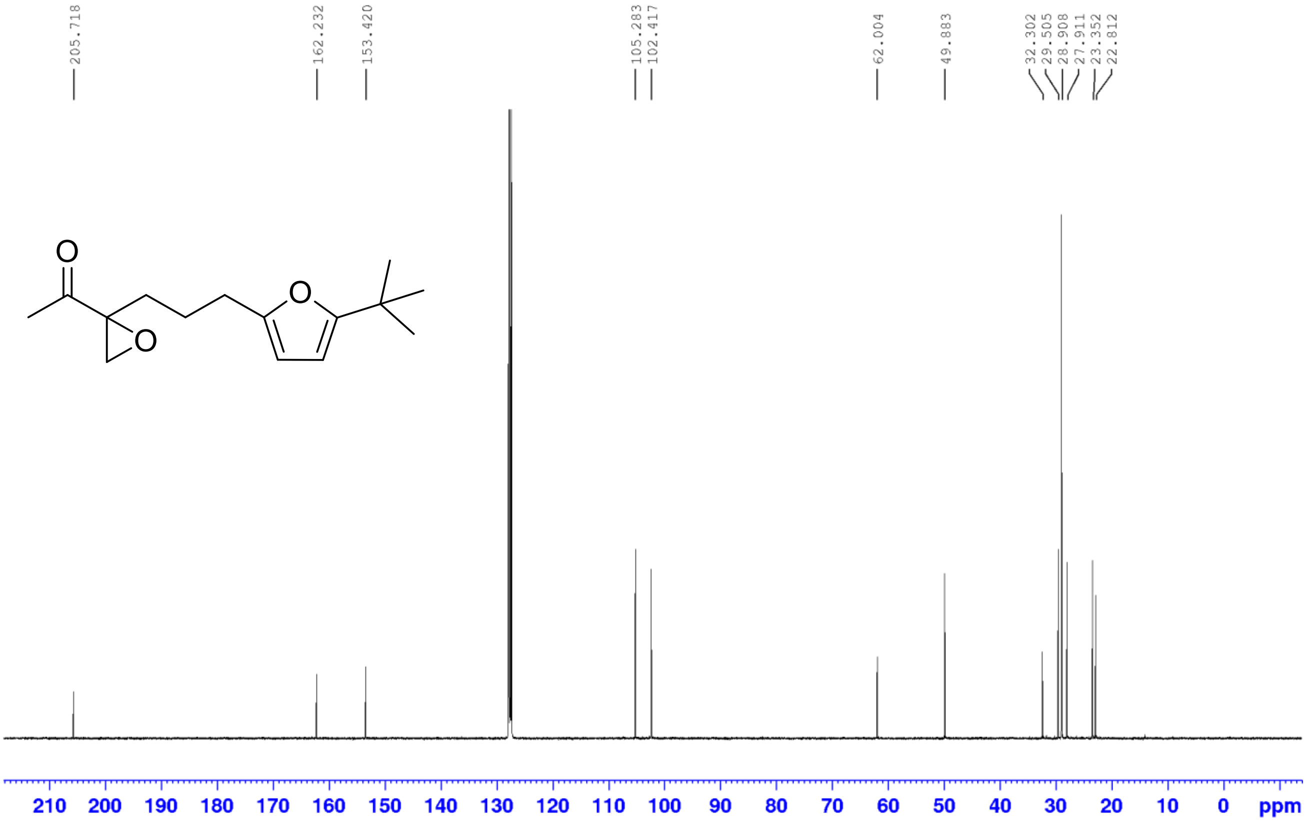

21, $CDCl_3$, 400 MHz

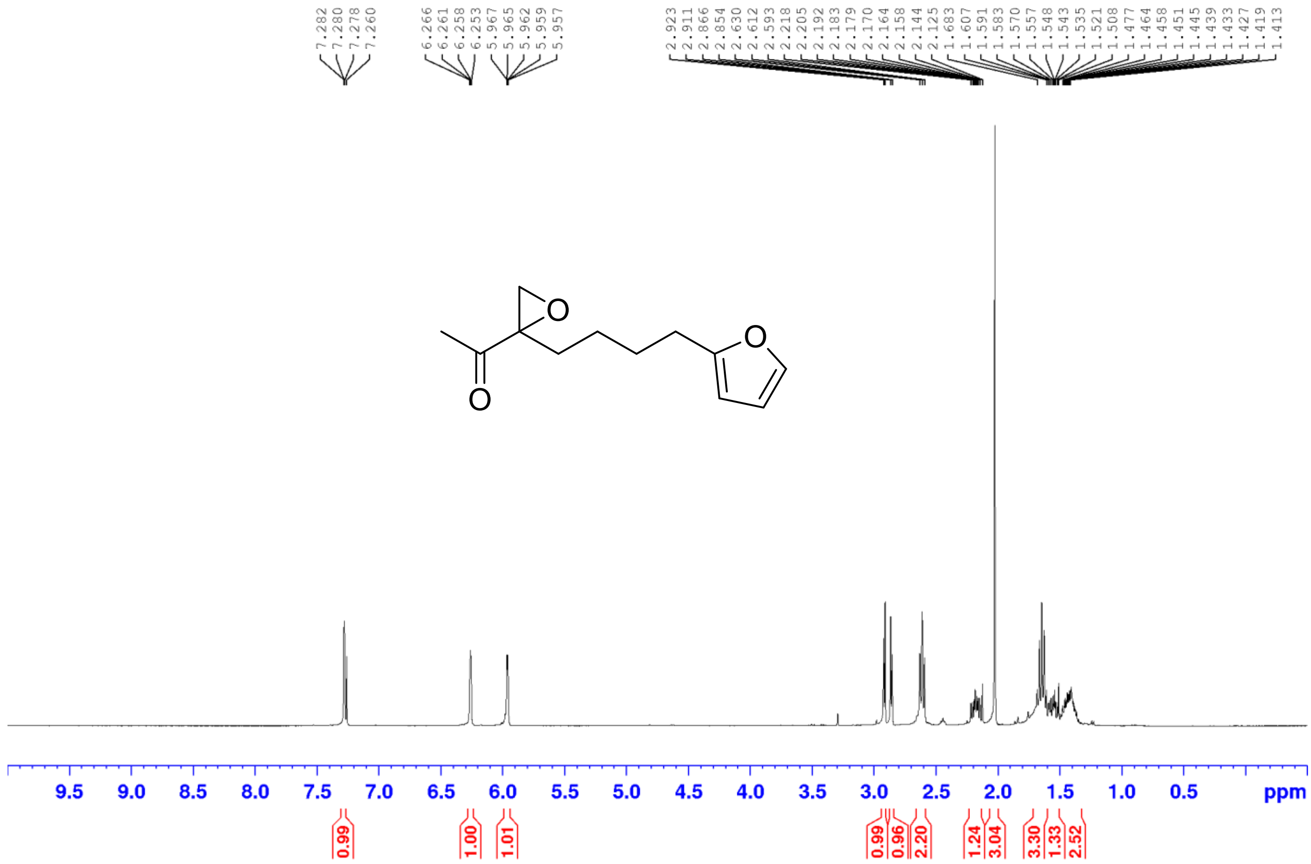

21, $CDCl_3$, 100 MHz

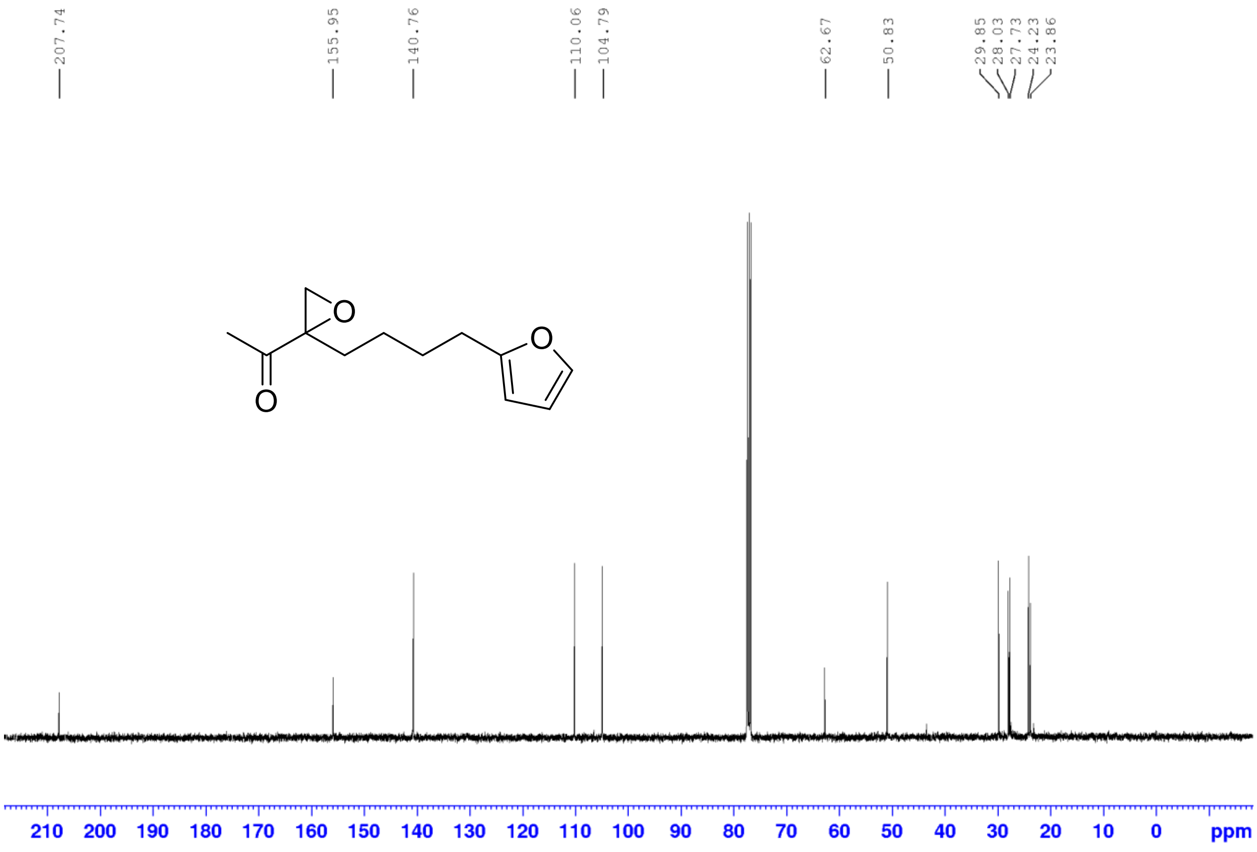

21’, C_6D_6, 400 MHz

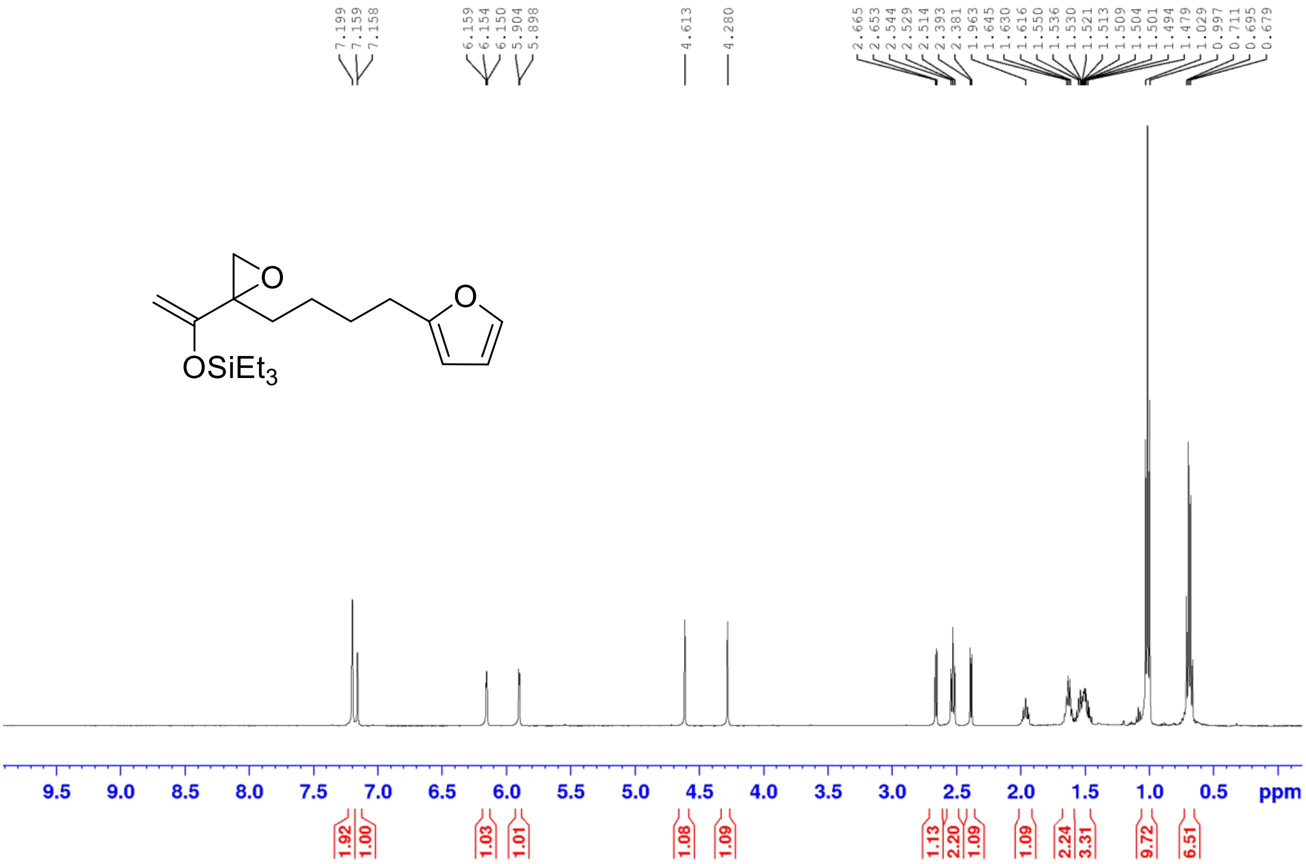

21’, C_6D_6, 100 MHz

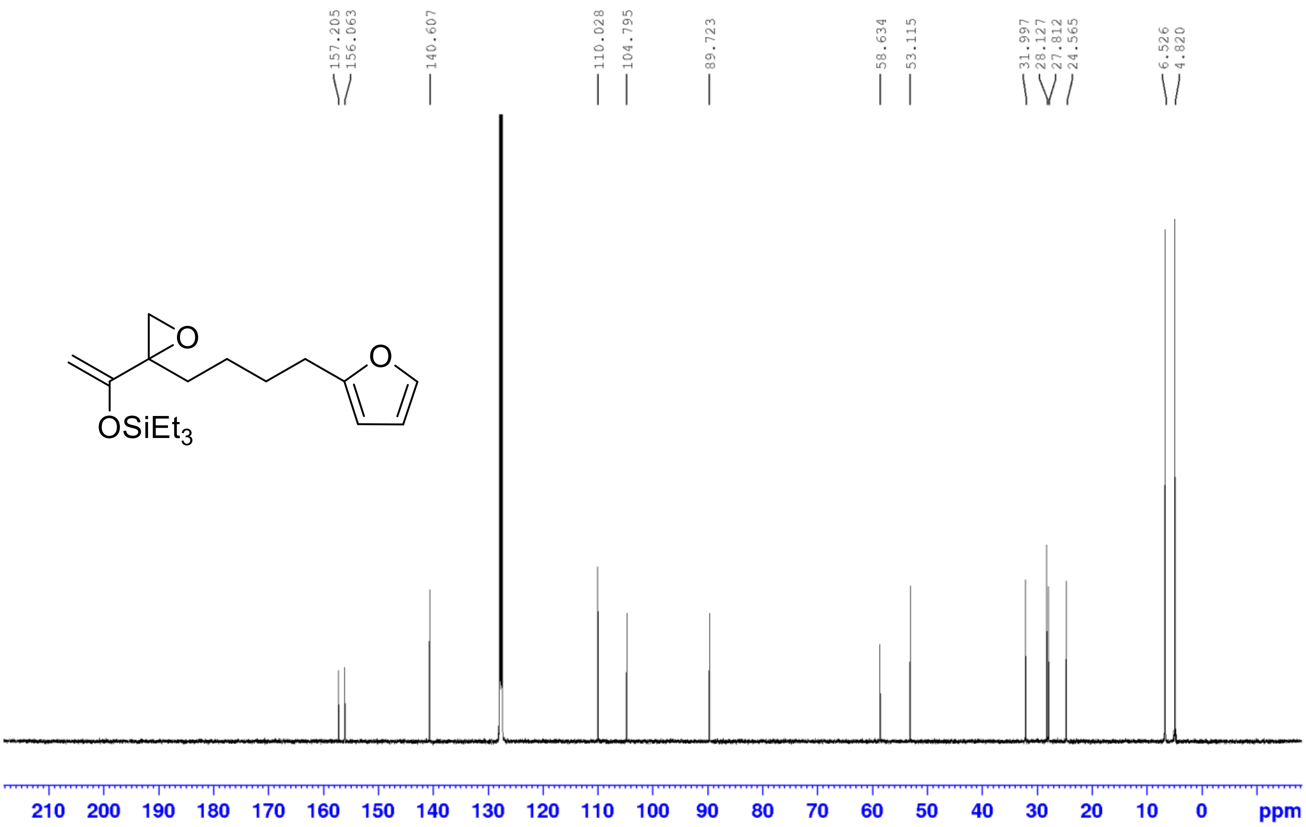

14, C_6D_6, 600 MHz

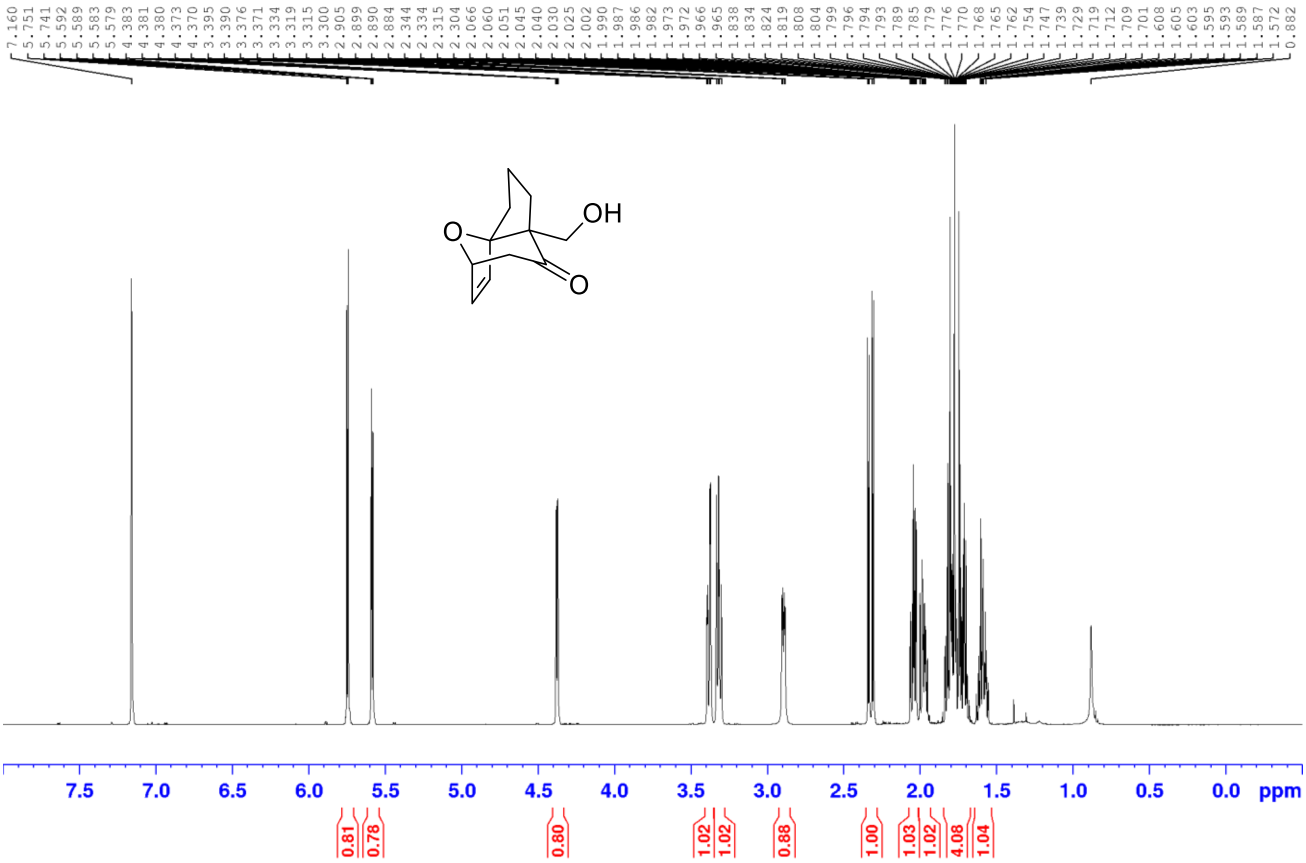

14, C_6D_6, 151 MHz

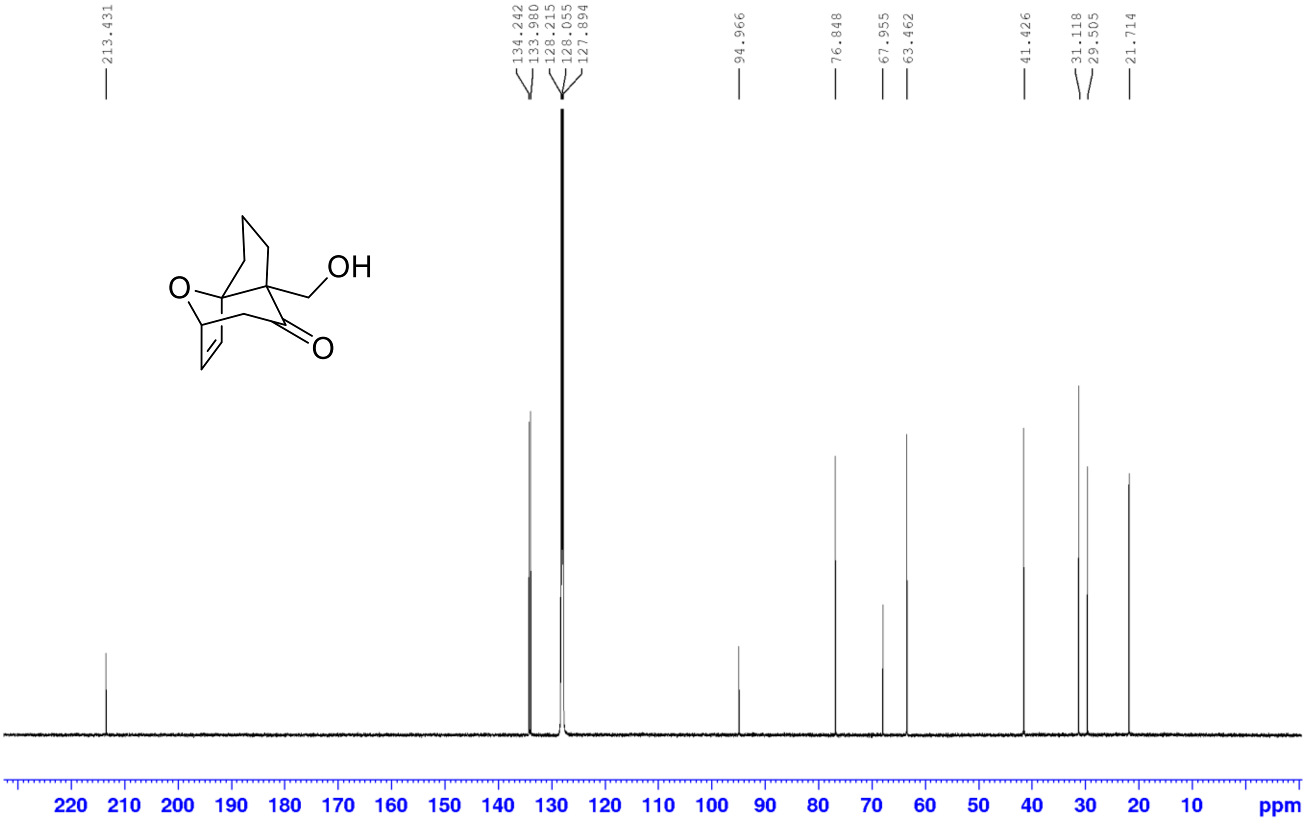

14, NOESY

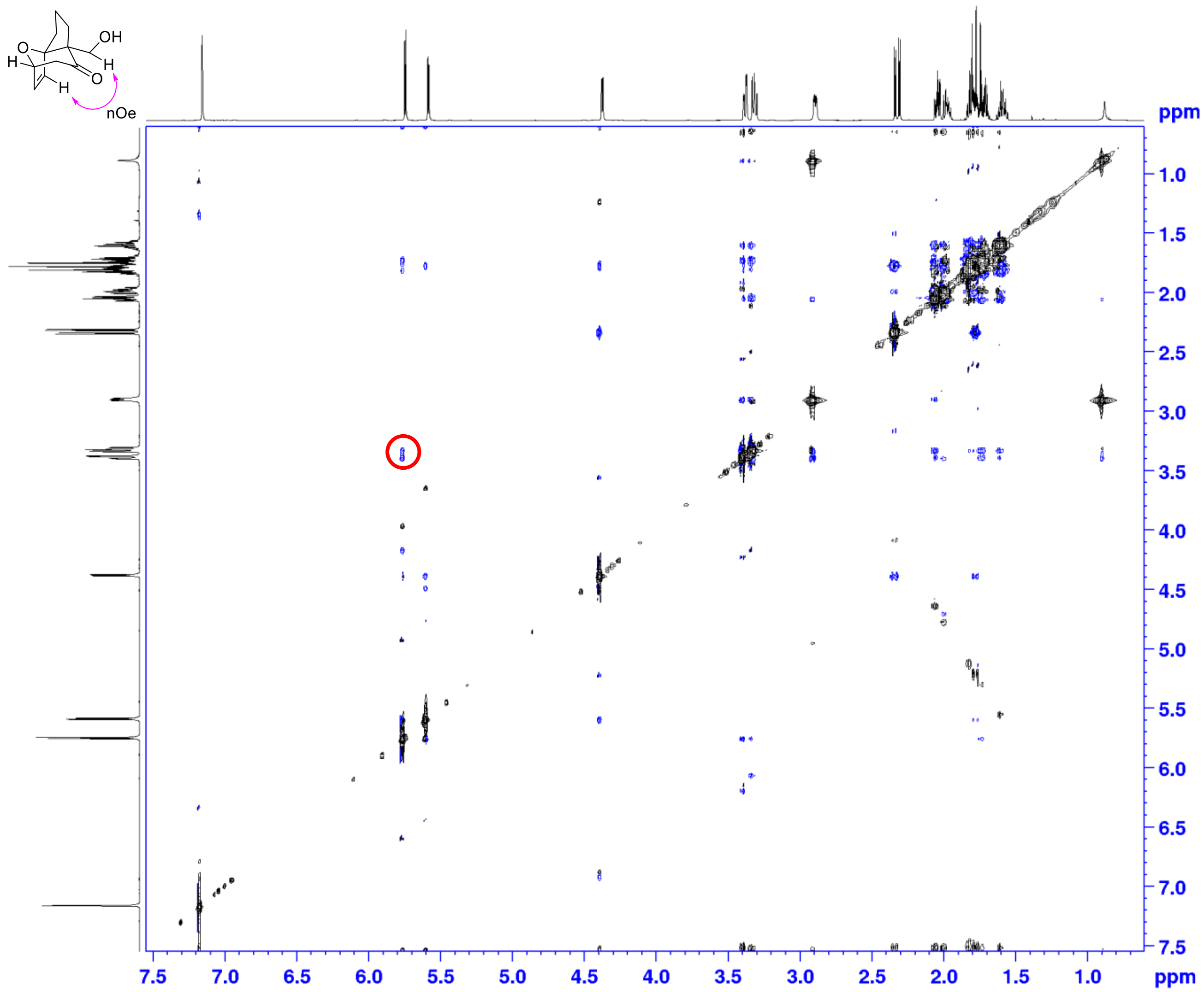

OH
H
O
H
O
H
nOe
ppm
1.0
1.5
2.0
2.5
3.0
3.5
4.0
4.5
5.0
5.5
6.0
6.5
7.0
7.5
7.5
7.0
6.5
6.0
5.5
5.0
4.5
4.0
3.5
3.0
2.5
2.0
1.5
1.0
ppm

15, $CDCl_3$, 600 MHz

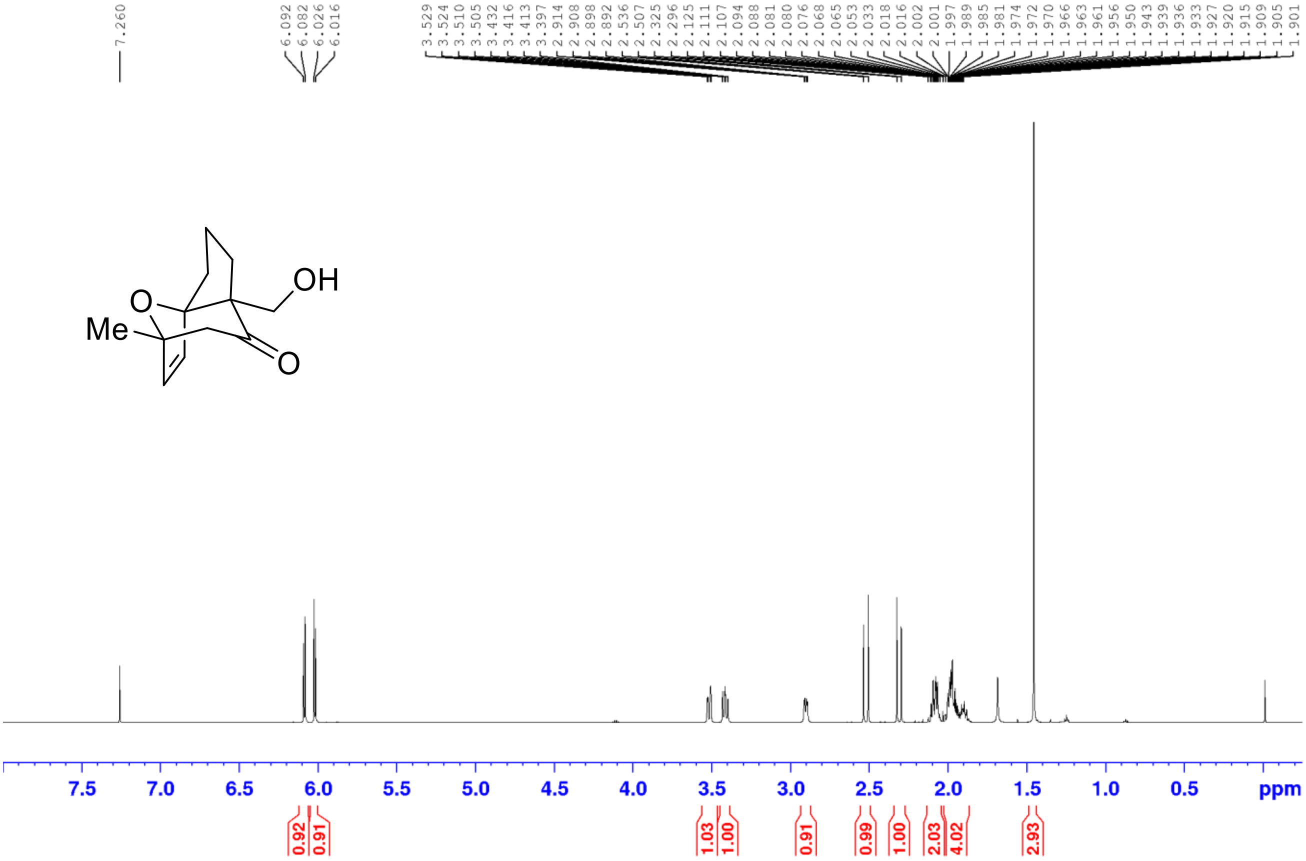

15, $CDCl_3$, 151 MHz

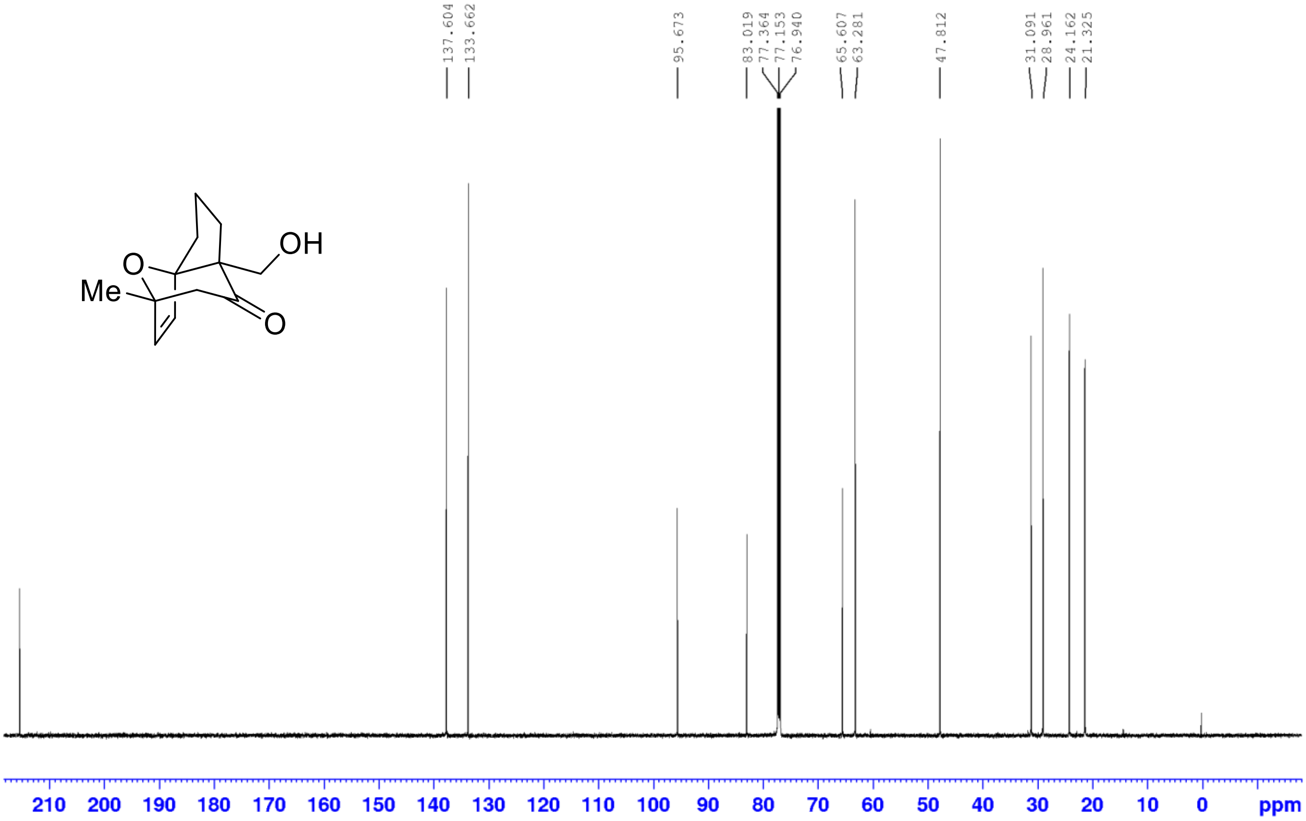

15, NOESY

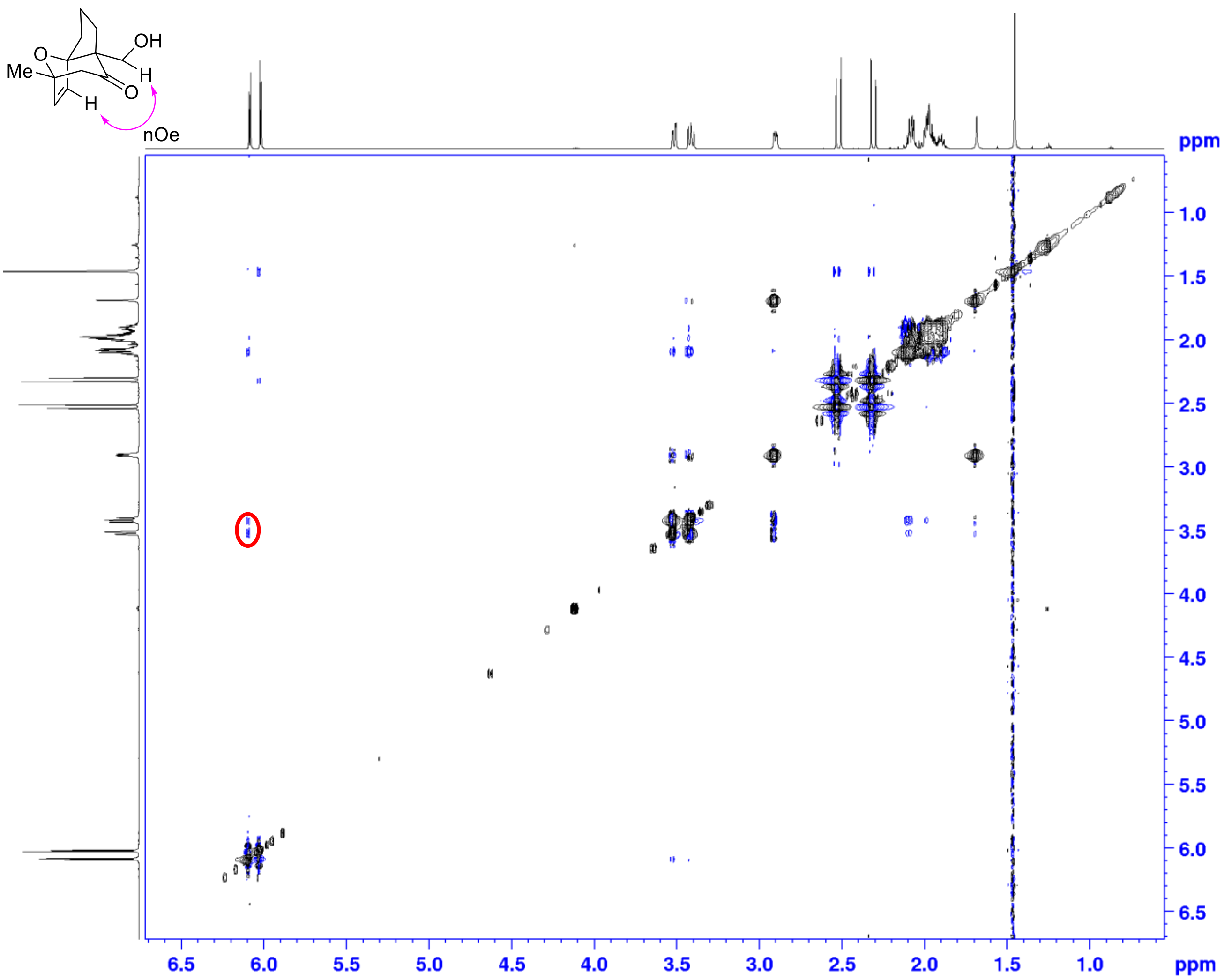
OH
O
Me
H
O
H
nOe
ppm
1.0
1.5
2.0
2.5
3.0
3.5
4.0
4.5
5.0
5.5
6.0
6.5
6.5
6.0
5.5
5.0
4.5
4.0
3.5
3.0
2.5
2.0
1.5
1.0
ppm

16, $CDCl_3$, 600 MHz

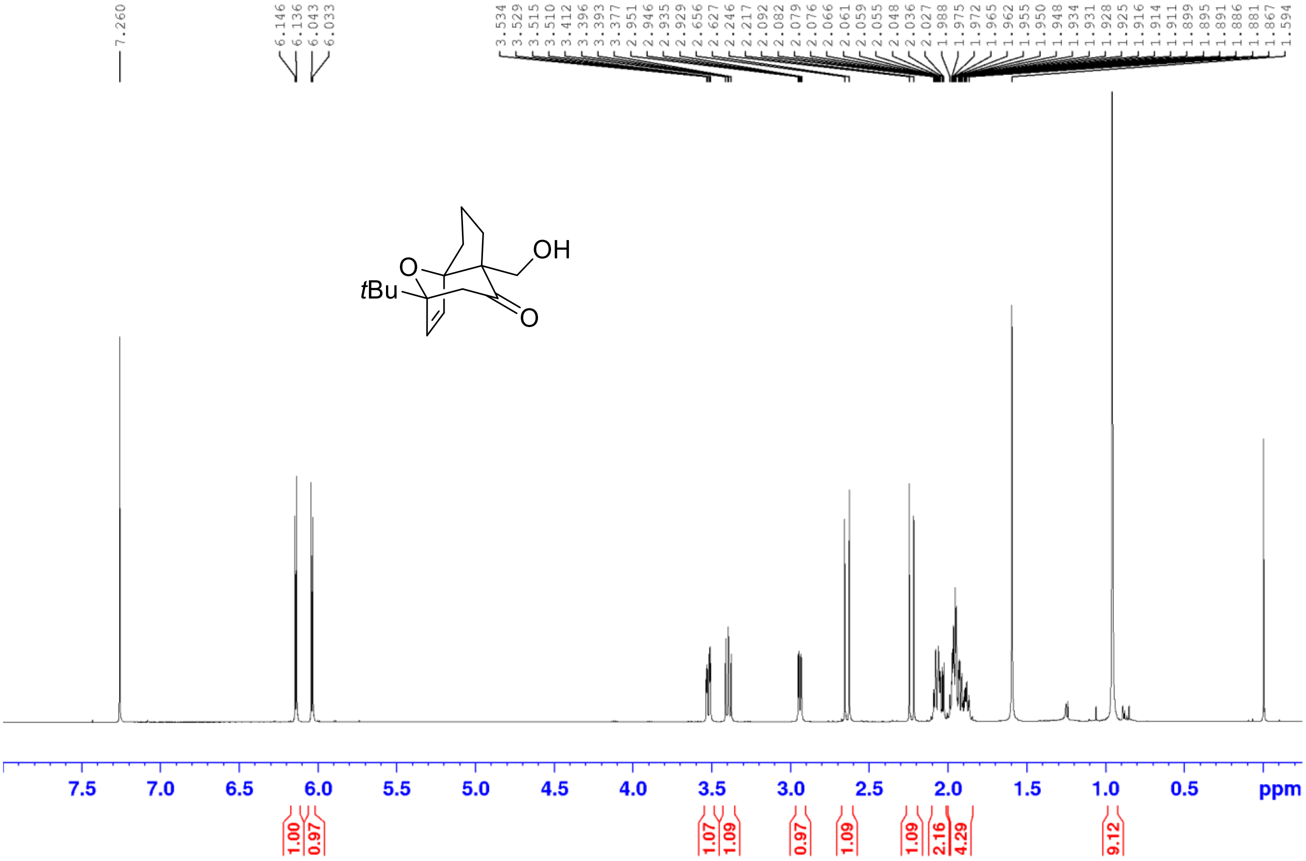

16, $CDCl_3$, 151 MHz

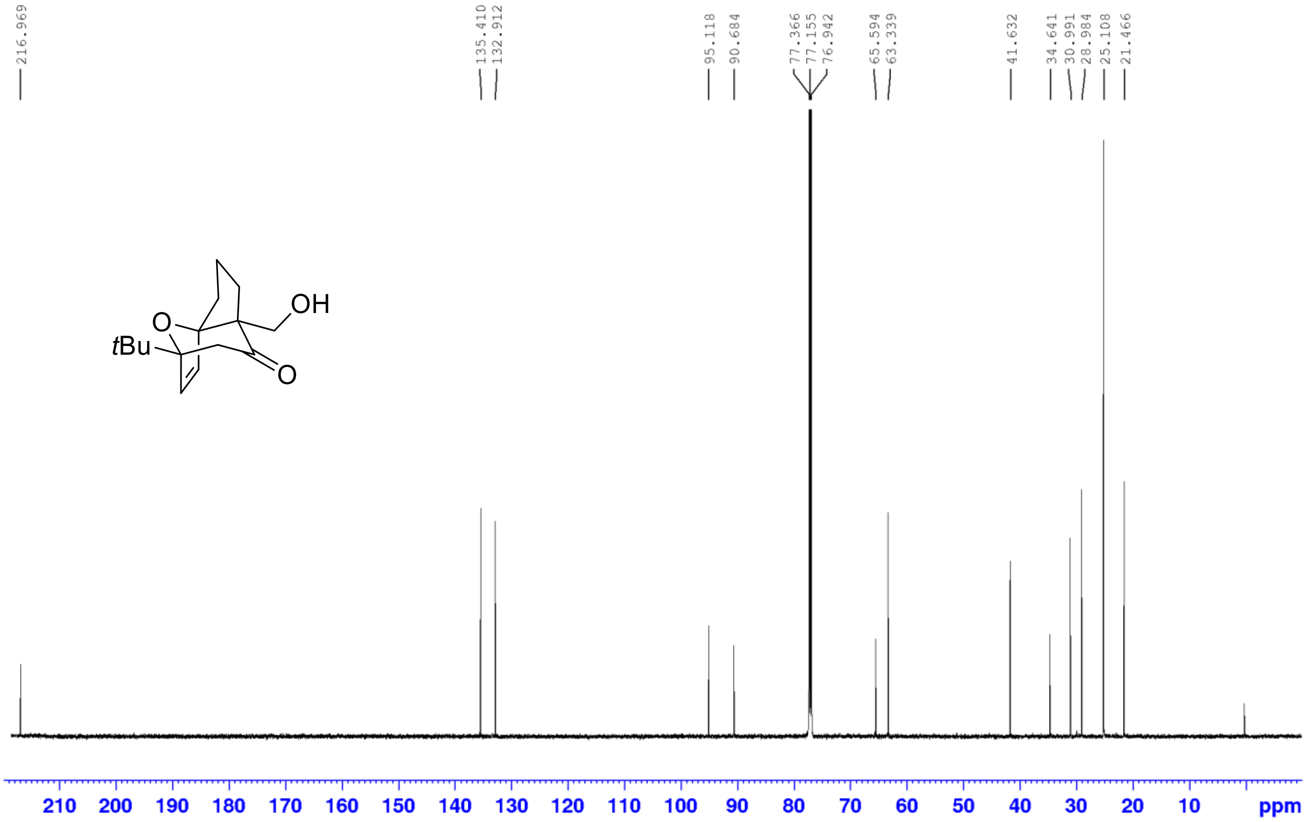

16, NOESY

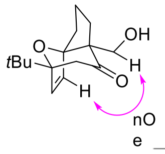
OH
O
tBu
H
O
H
nO
e

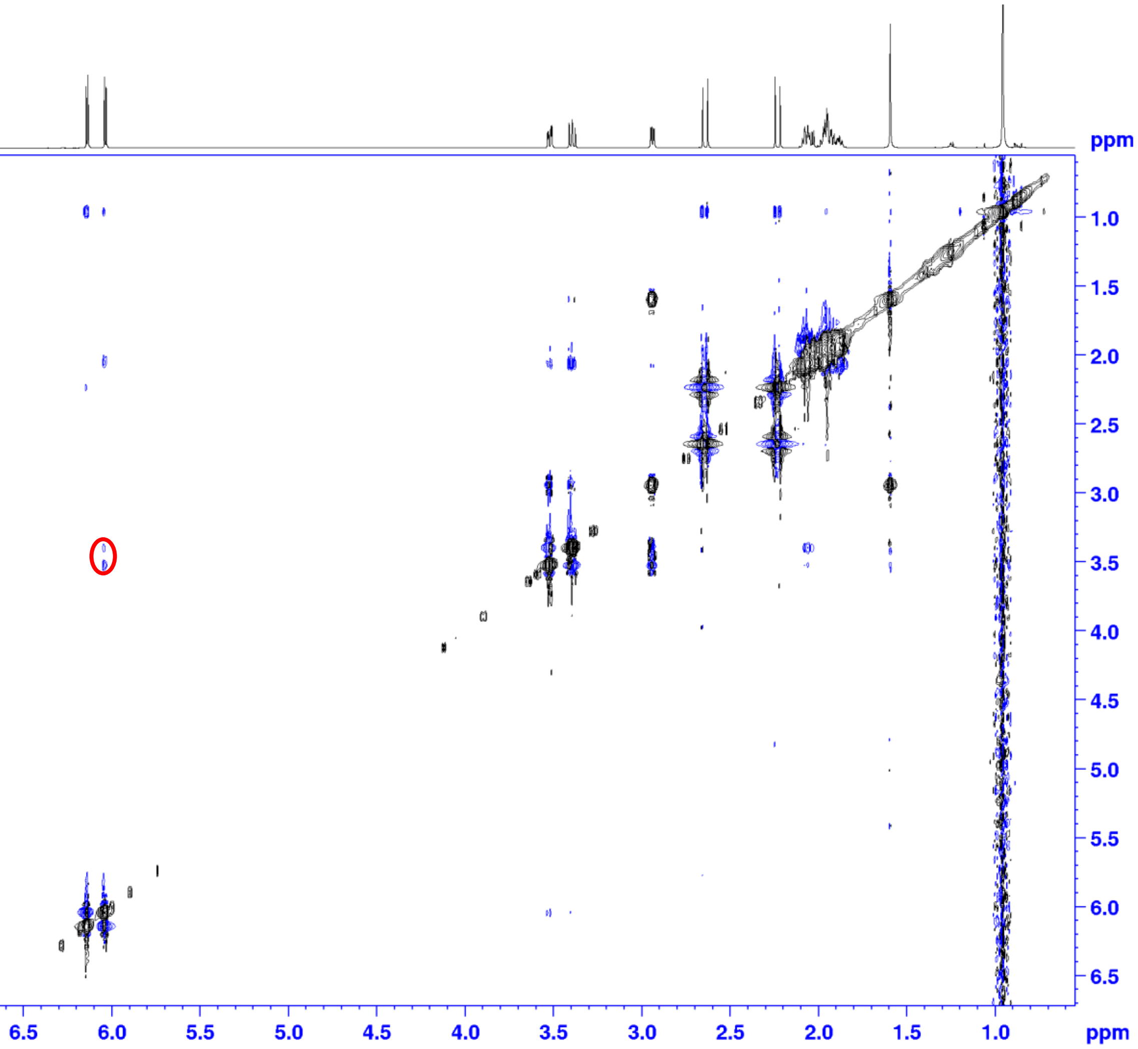
ppm
1.0
1.5
2.0
2.5
3.0
3.5
4.0
4.5
5.0
5.5
6.0
6.5
6.5 6.0 5.5 5.0 4.5 4.0 3.5 3.0 2.5 2.0 1.5 1.0 ppm

16’, C_6D_6, 400 MHz

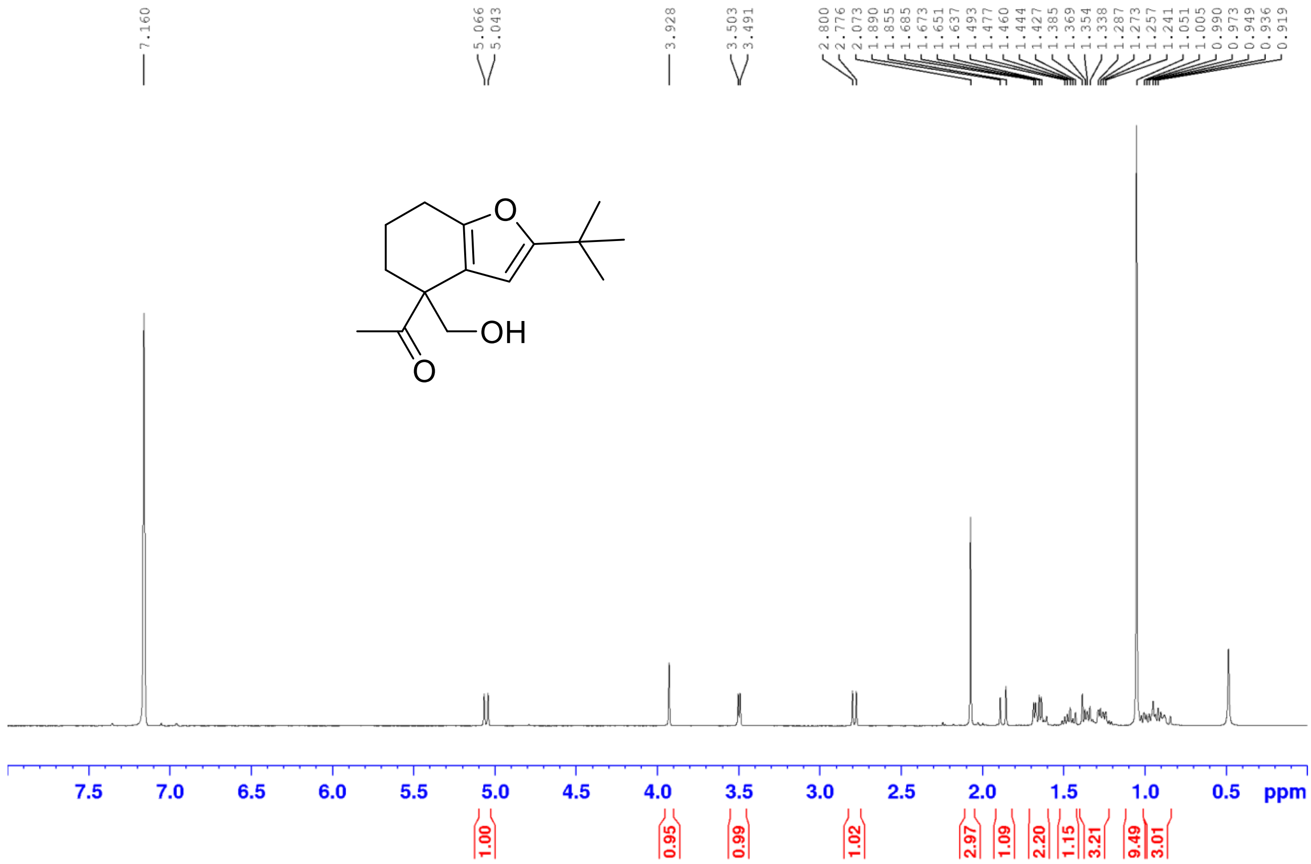

16’, C_6D_6, 100 MHz

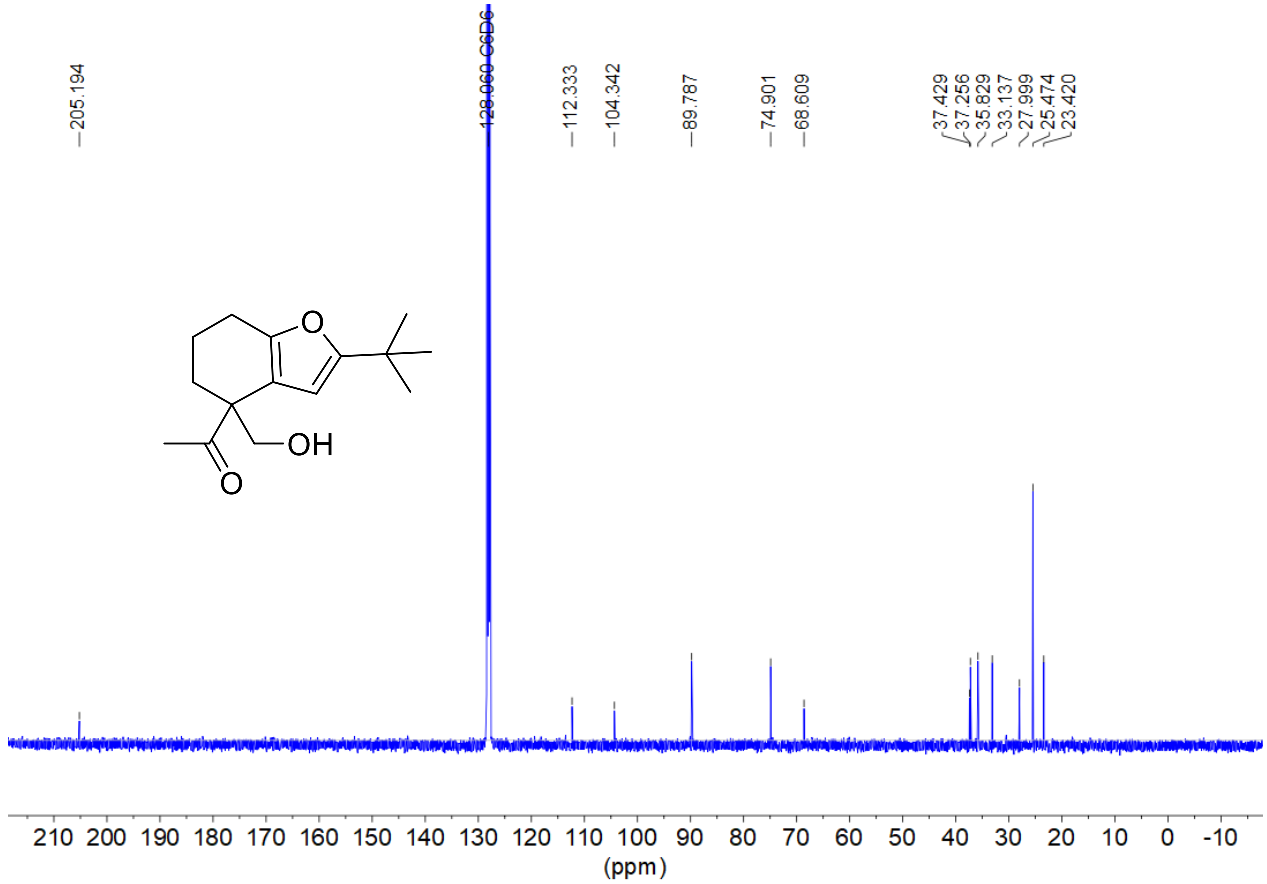

21b, $CDCl_3$, 500 MHz

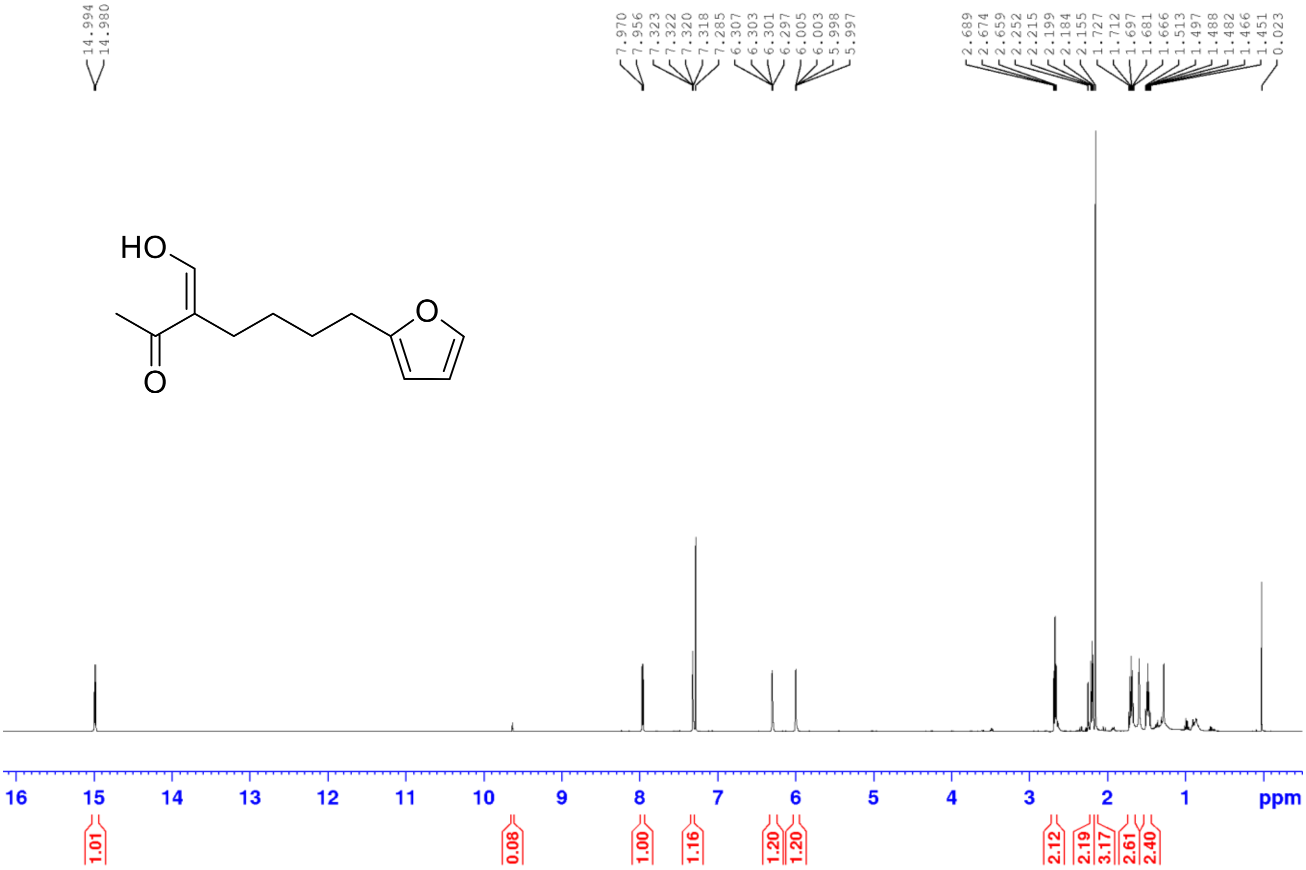

21b, $CDCl_3$, 125 MHz

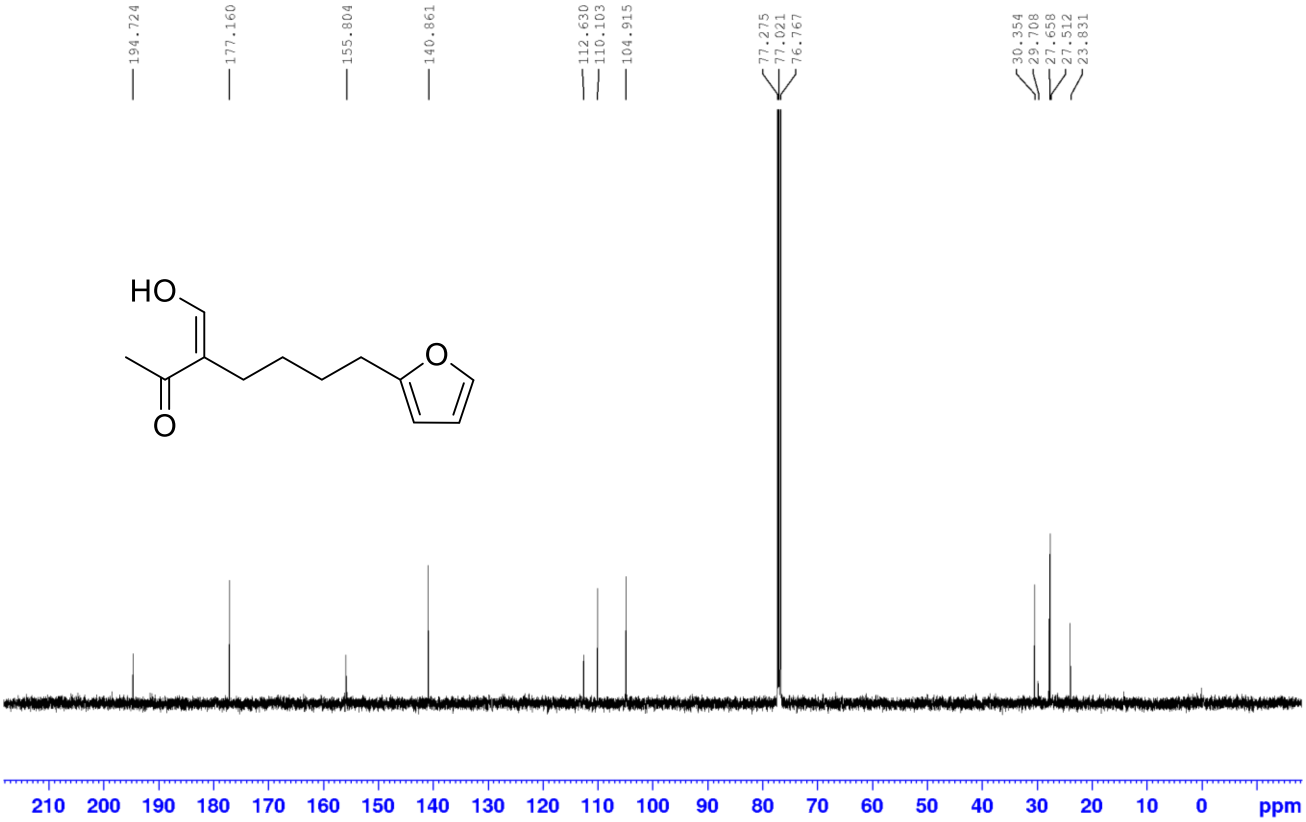

S6, $CDCl_3$, 400 MHz

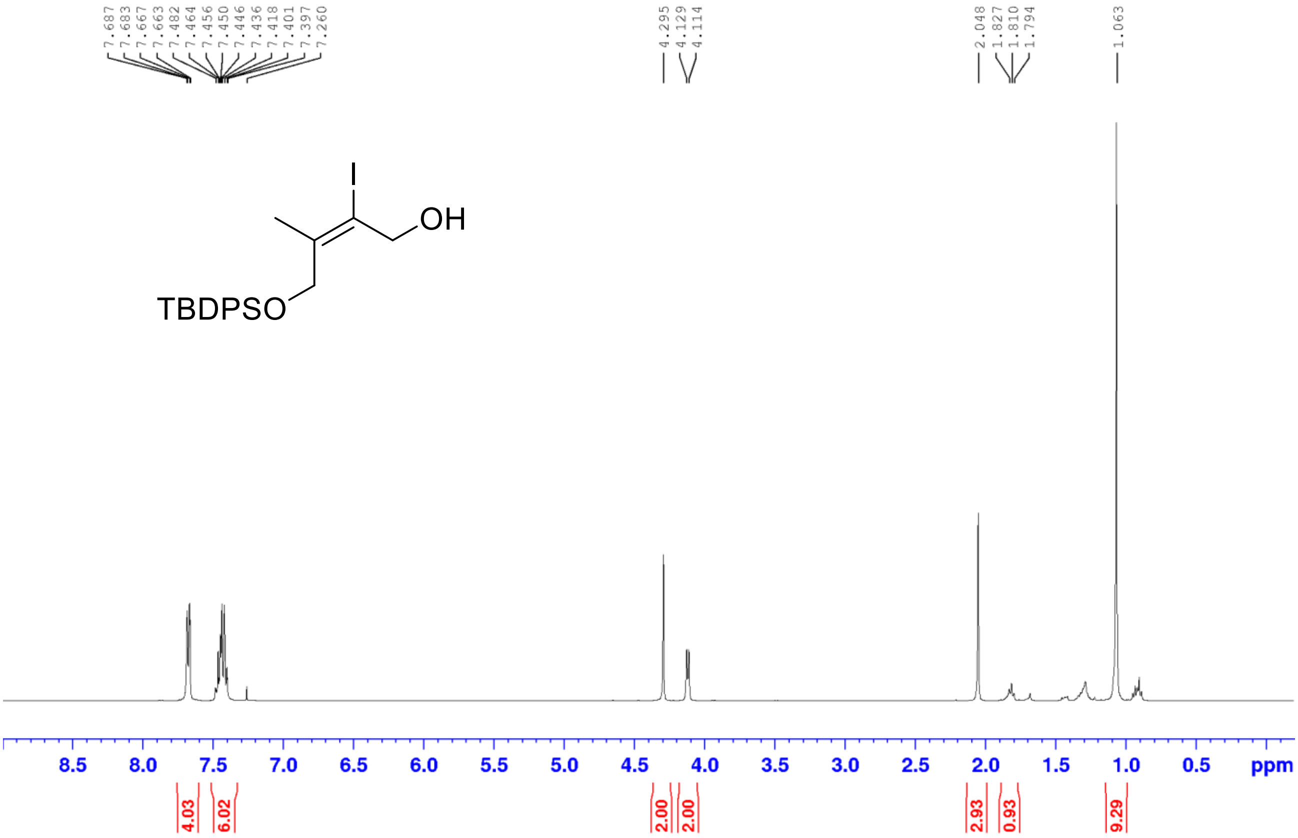

S6, $CDCl_3$, 100 MHz

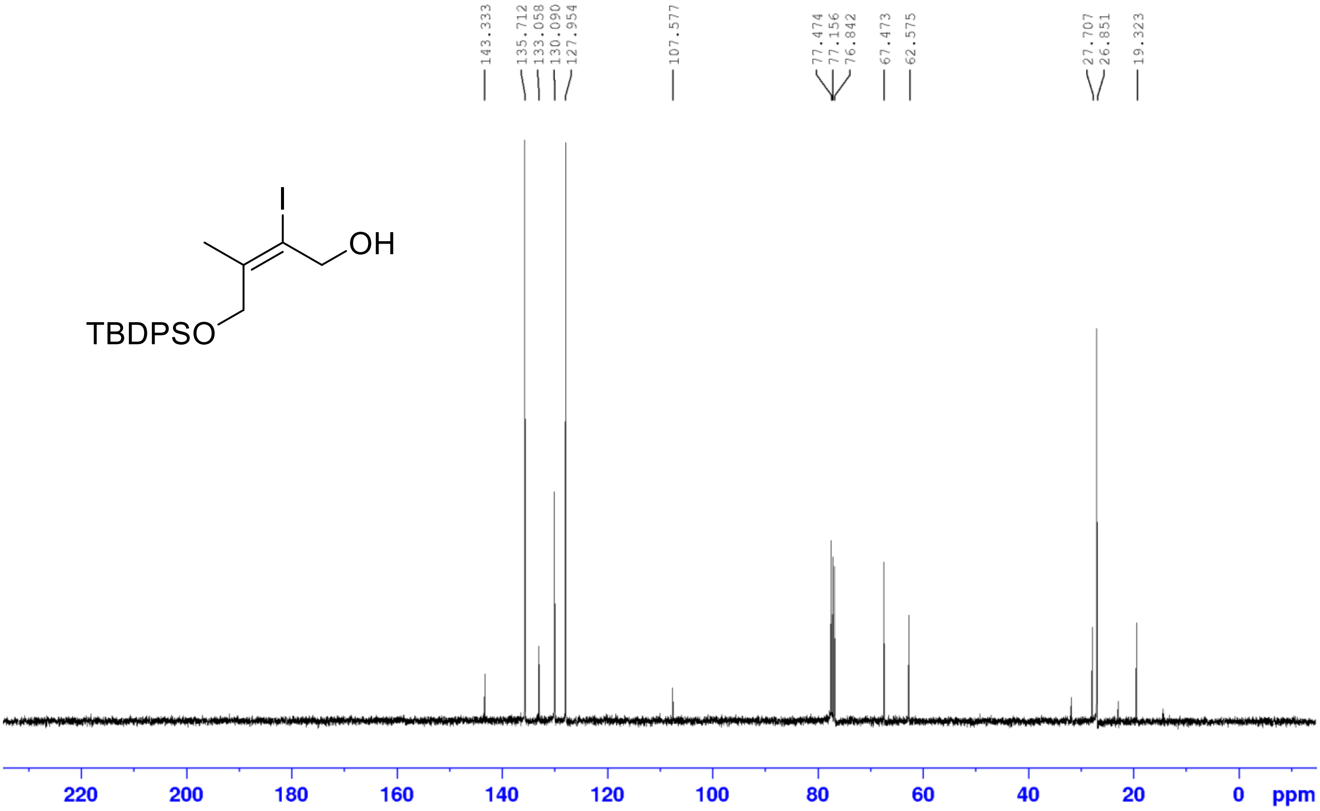

S7, $CDCl_3$, 400 MHz

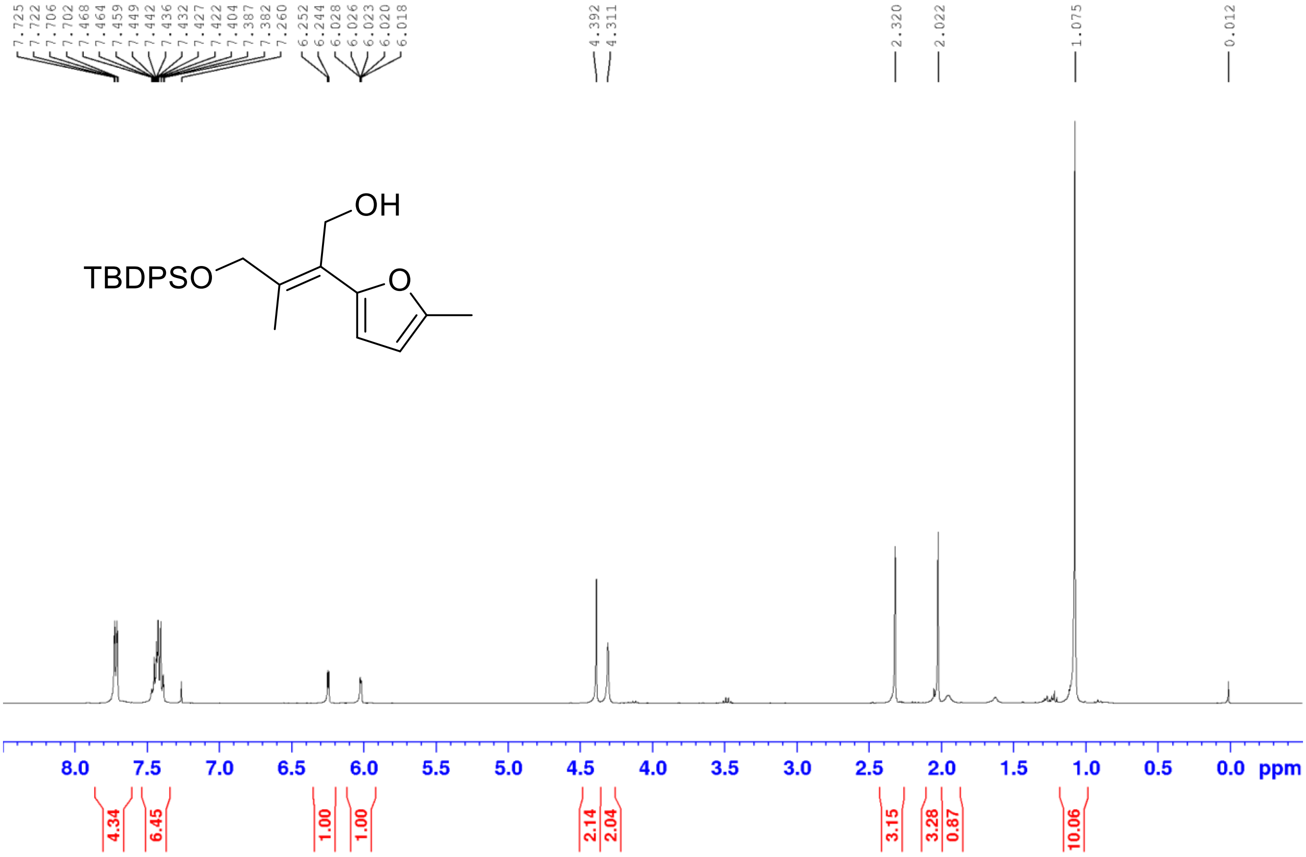

S7, $CDCl_3$, 100 MHz

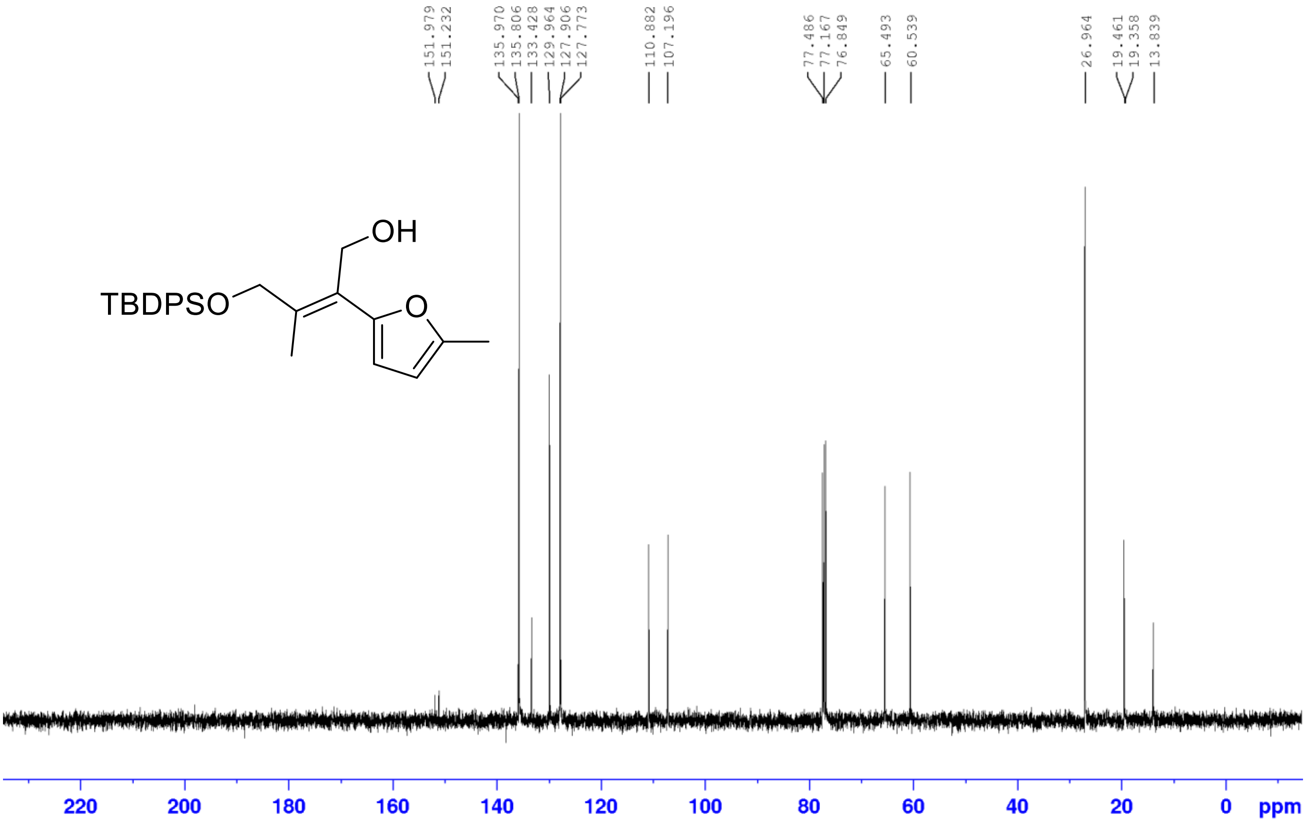

S10, $CDCl_3$, 400 MHz

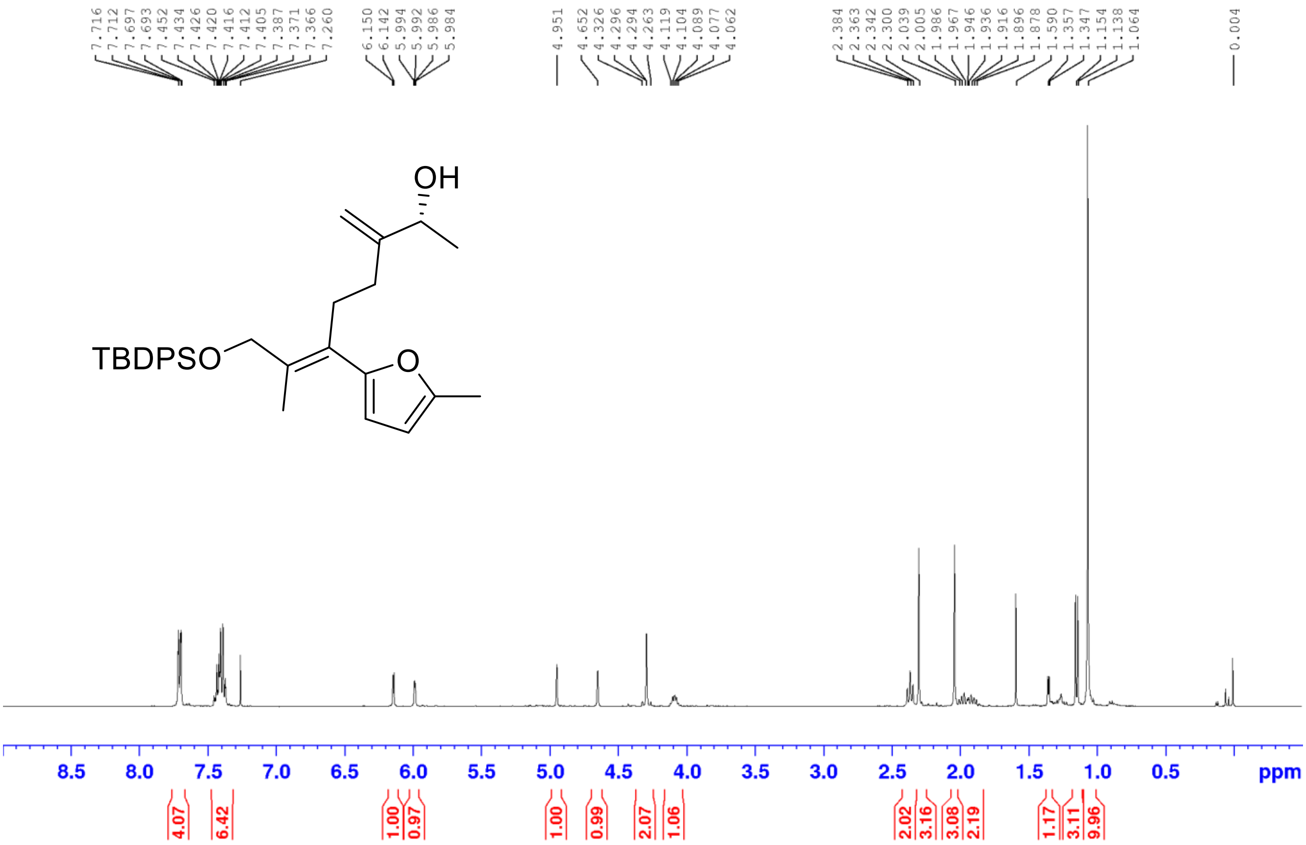

S10, $CDCl_3$, 100 MHz

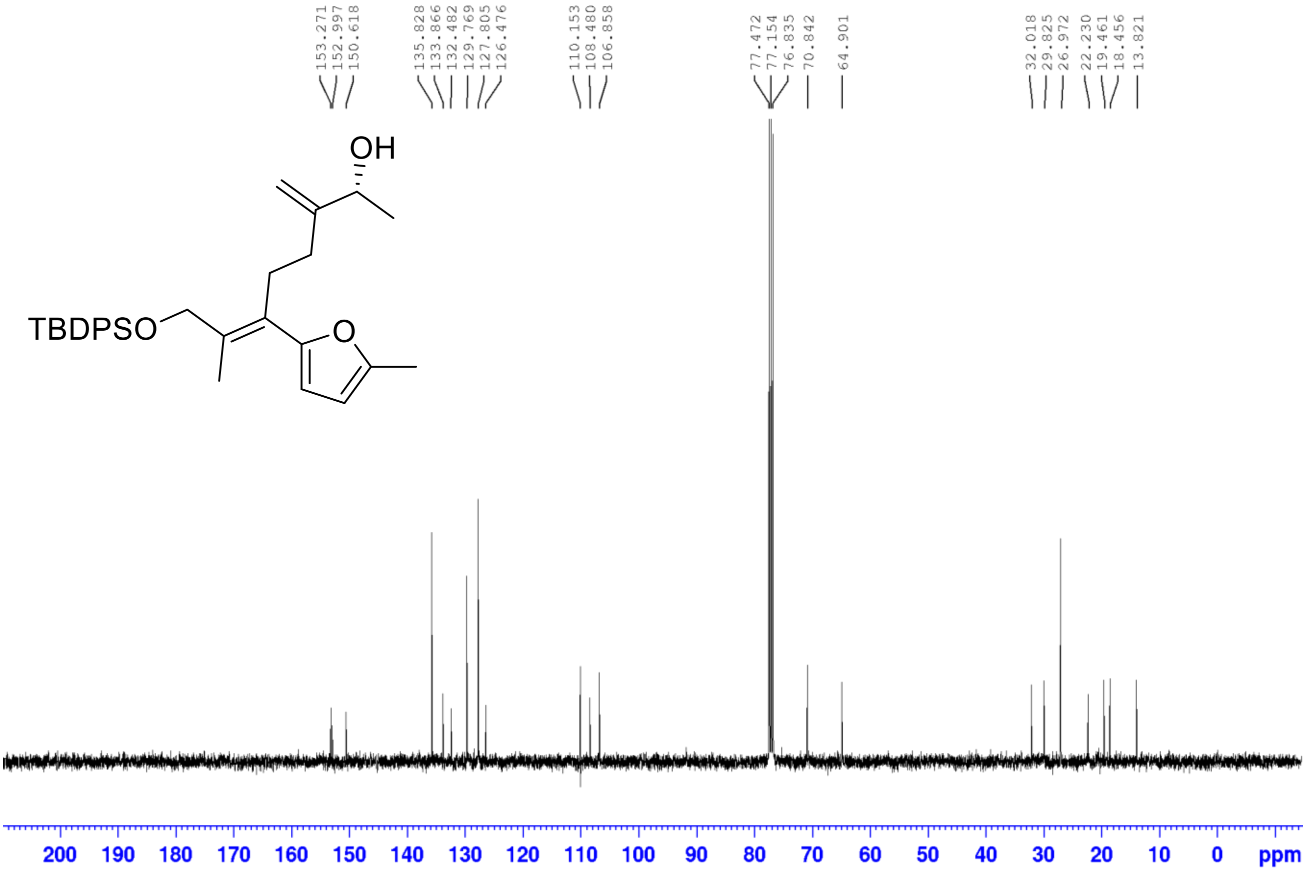

S11, $CDCl_3$, 400 MHz

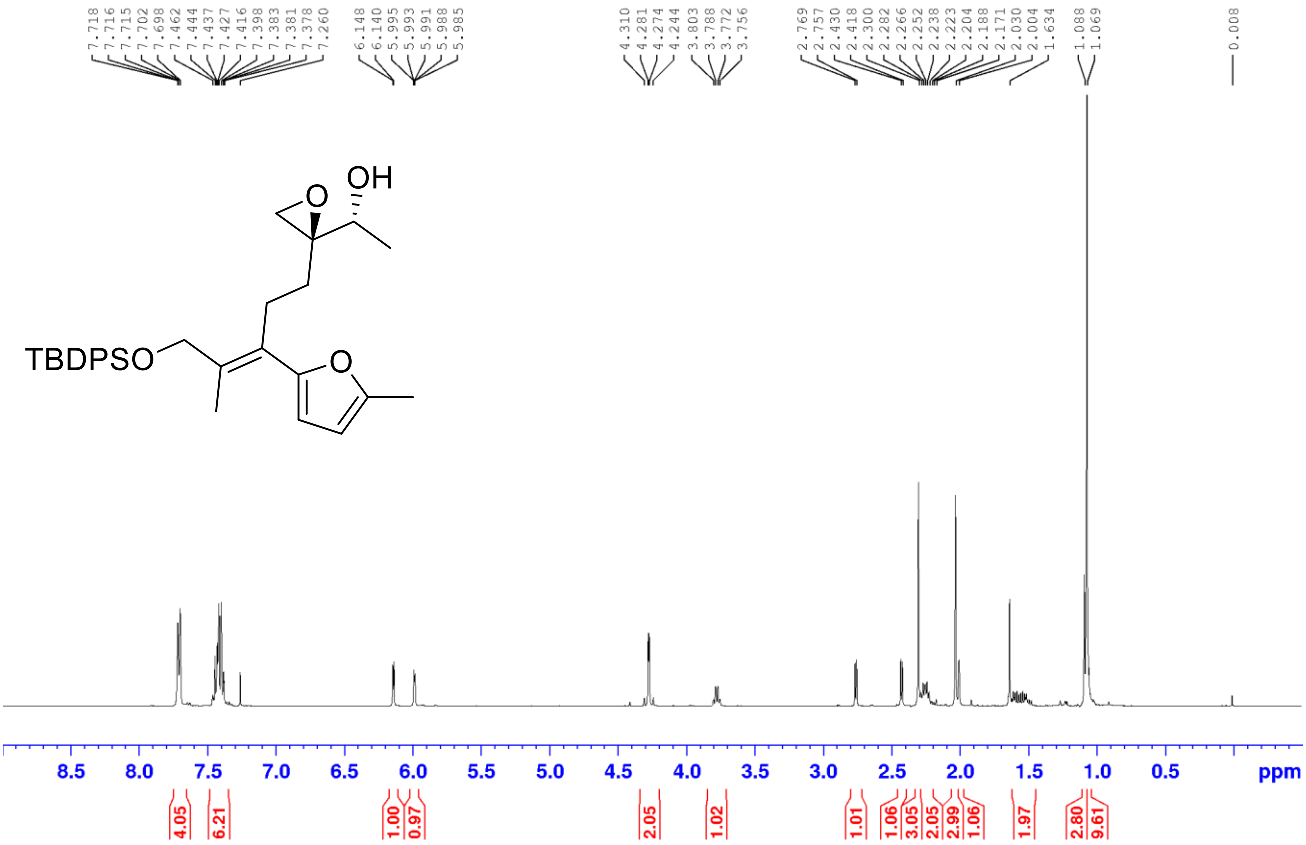

S11, $CDCl_3$, 100 MHz

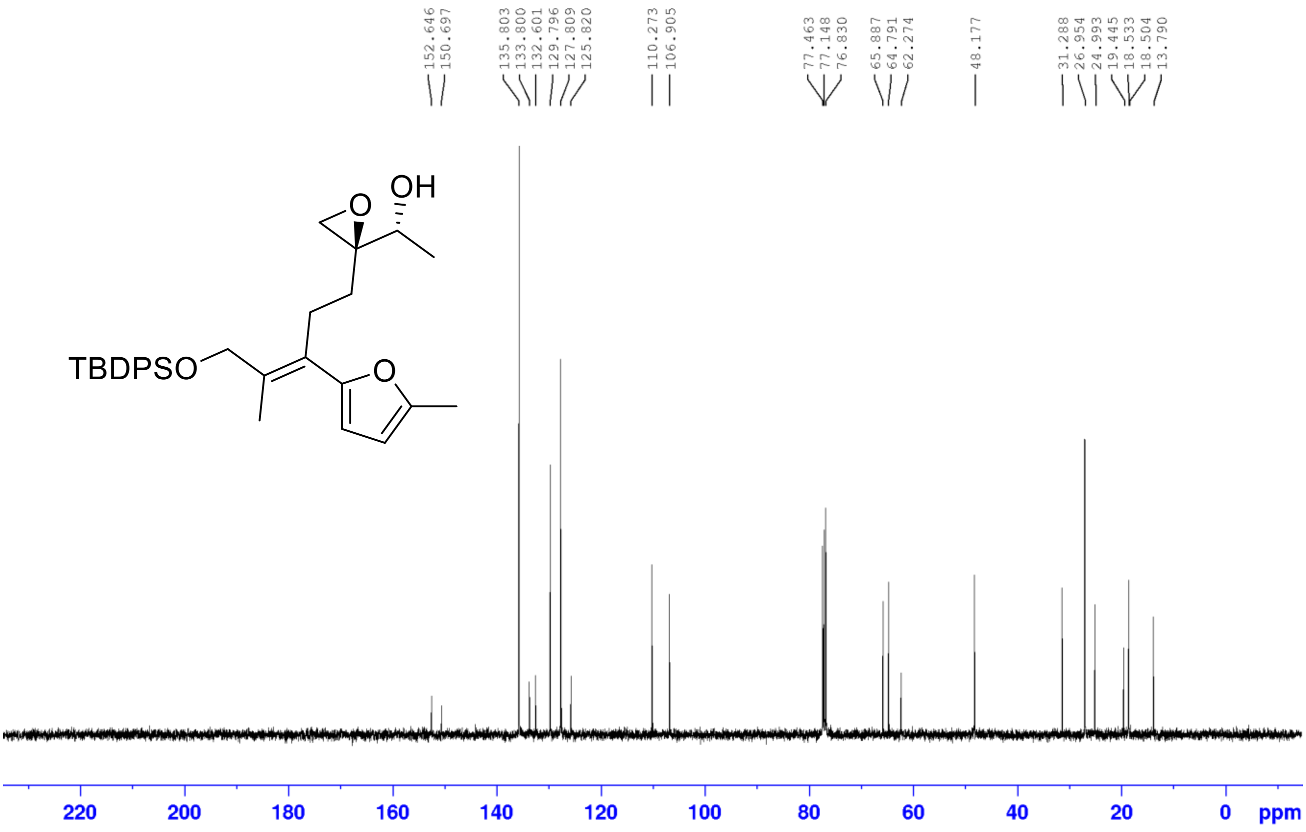

17, $CDCl_3$, 400 MHz

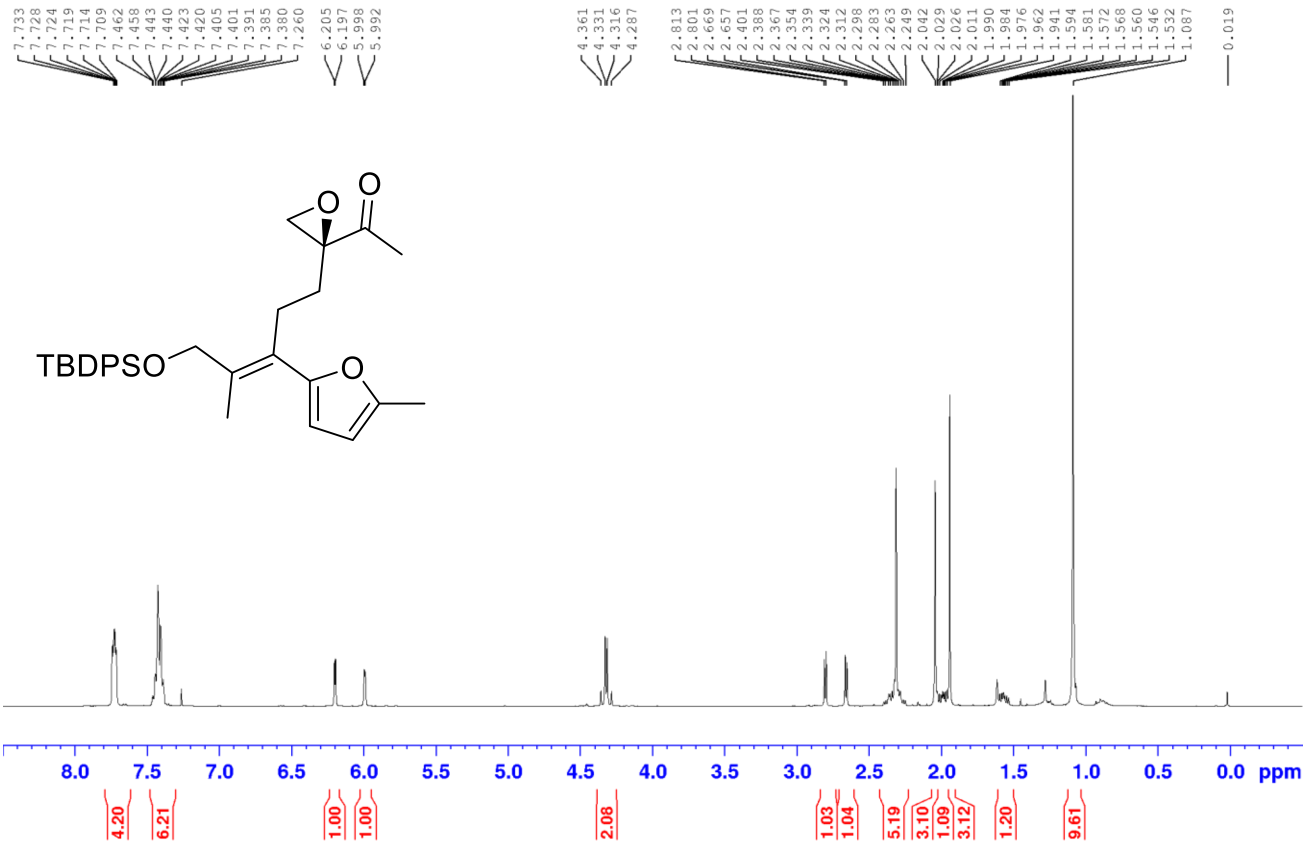

17, $CDCl_3$, 100 MHz

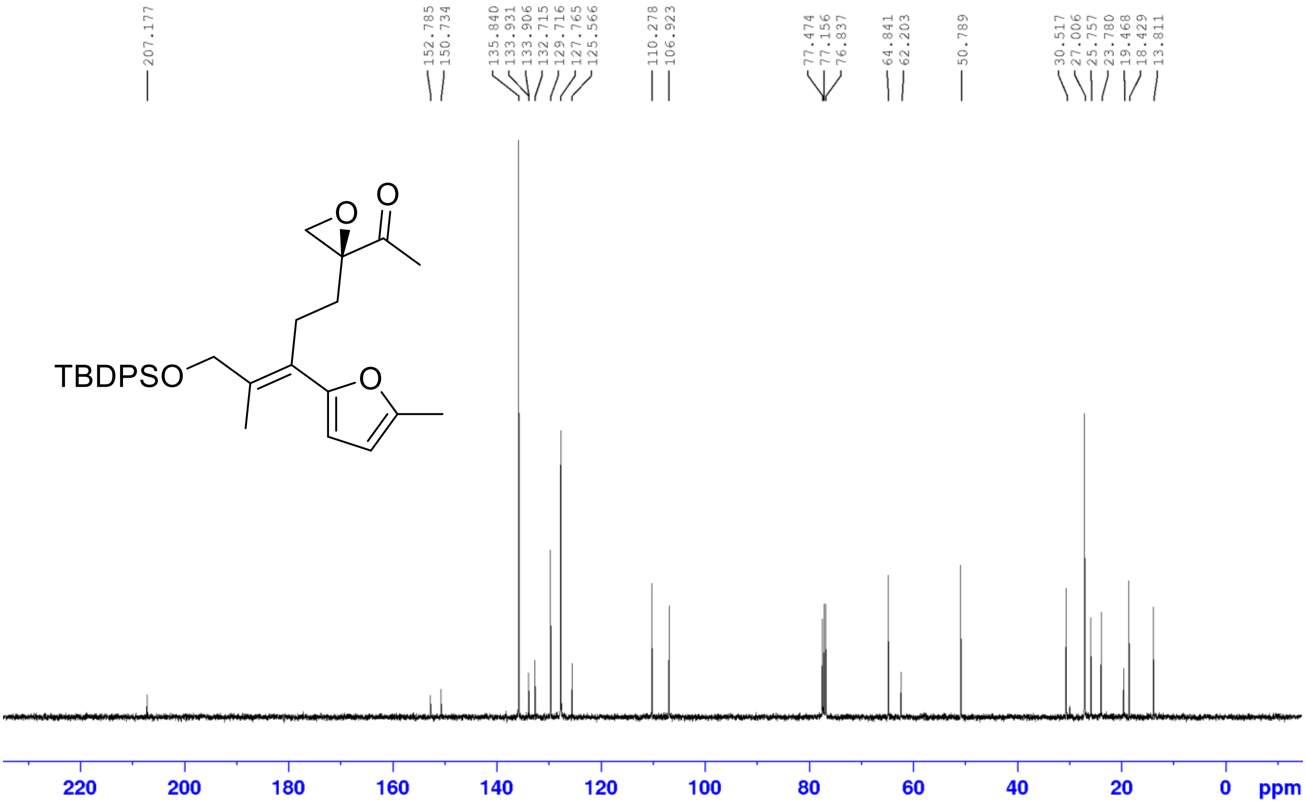

18, $CDCl_3$, 600 MHz

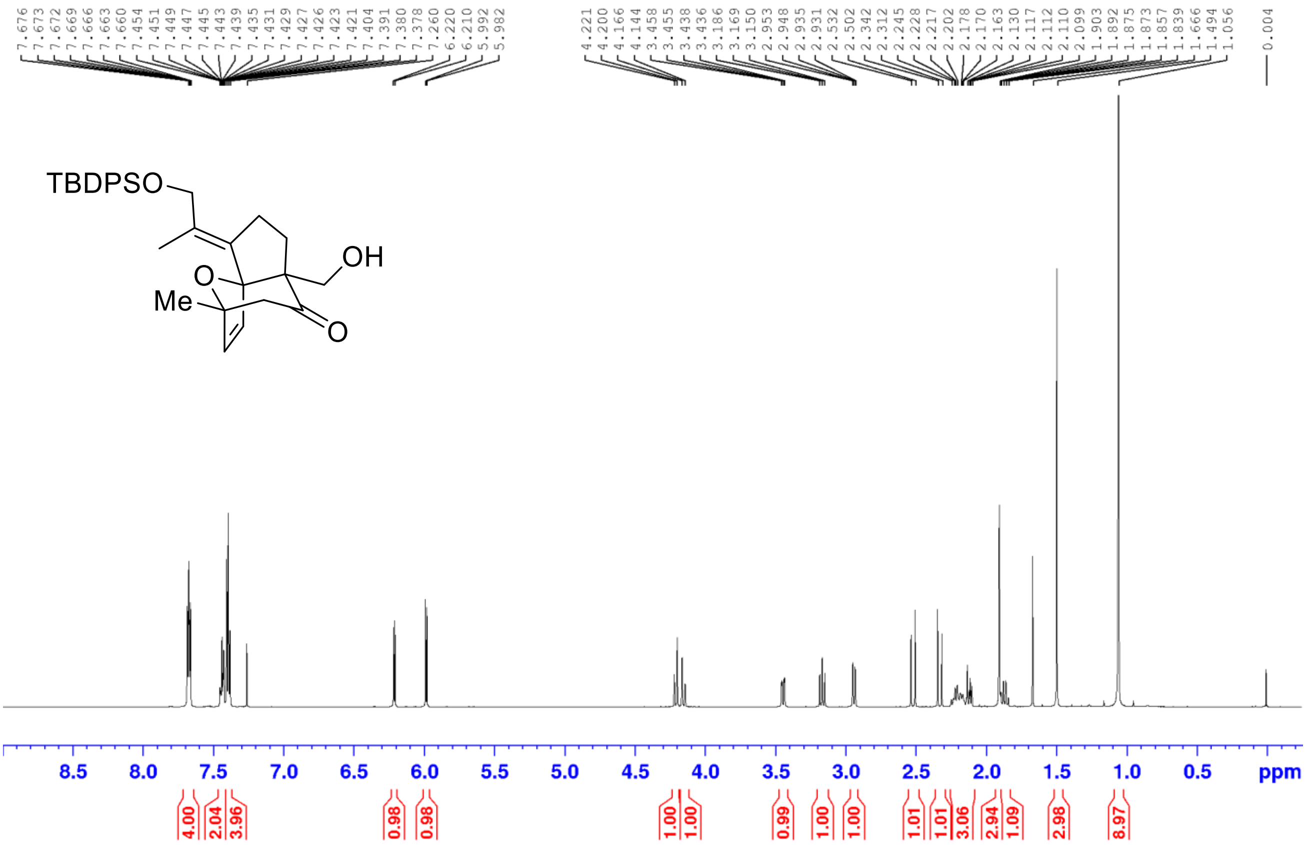

18, $CDCl_3$, 150 MHz

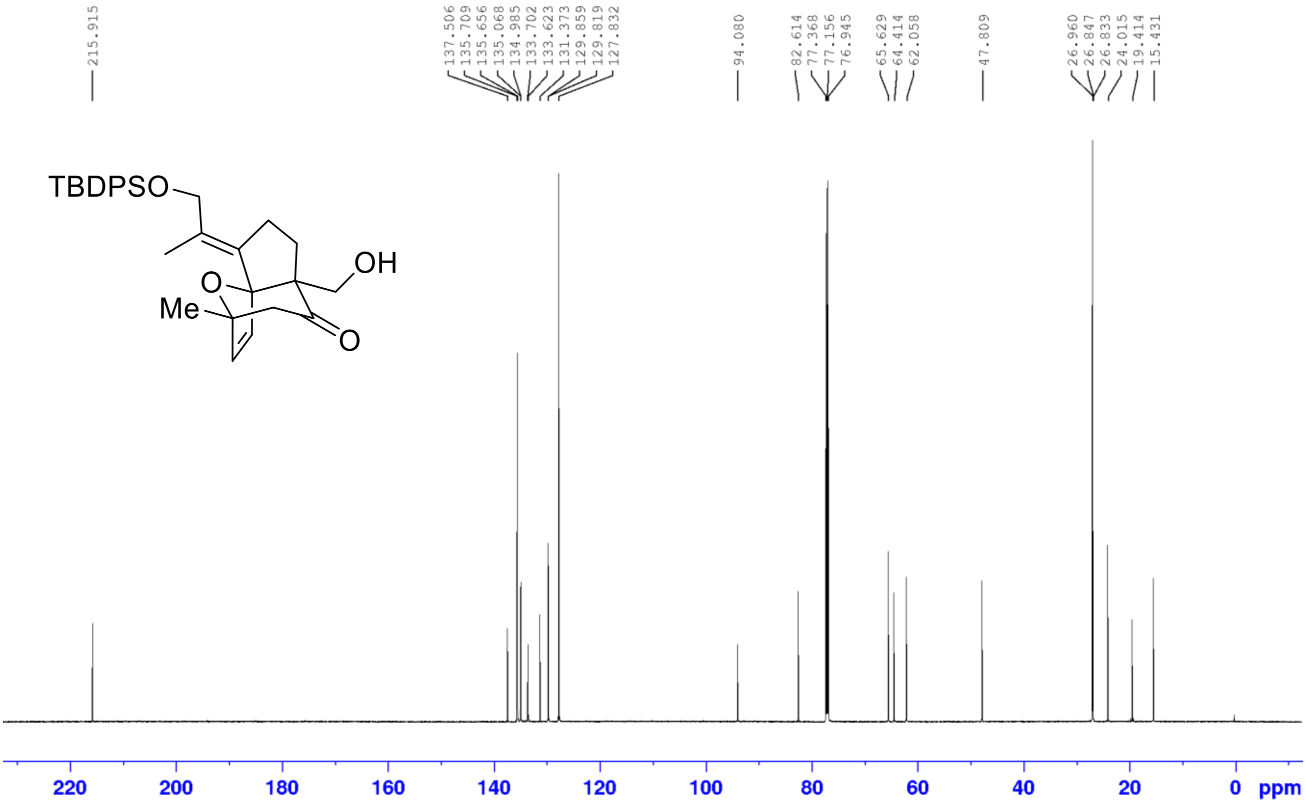

18, NOESY

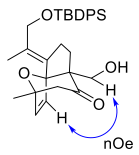
OTBDPS
OH
O
H
O
H
nOe

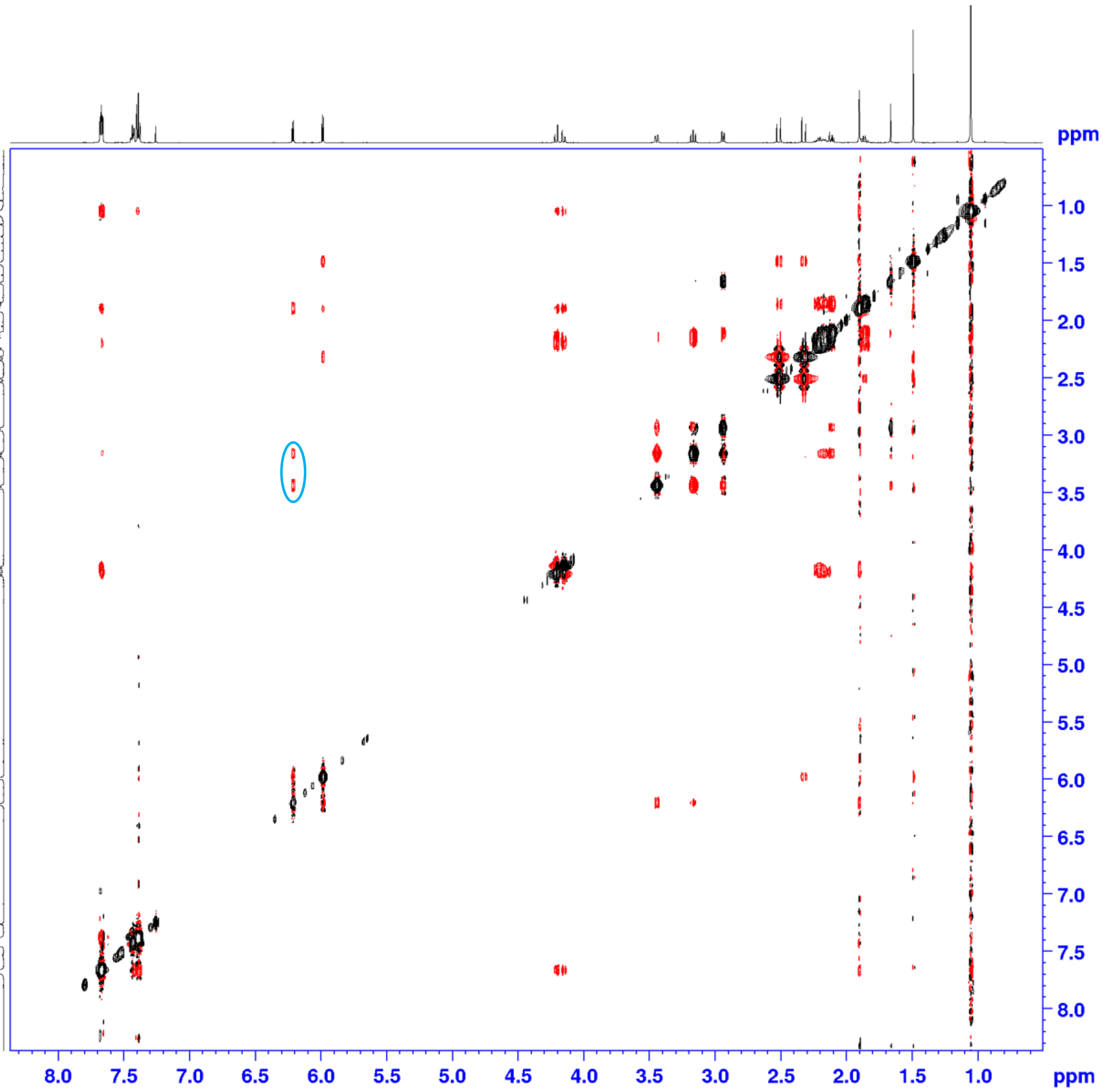
ppm
1.0
1.5
2.0
2.5
3.0
3.5
4.0
4.5
5.0
5.5
6.0
6.5
7.0
7.5
8.0
8.0
7.5
7.0
6.5
6.0
5.5
5.0
4.5
4.0
3.5
3.0
2.5
2.0
1.5
1.0
ppm

S14, $CDCl_3$, 500 MHz

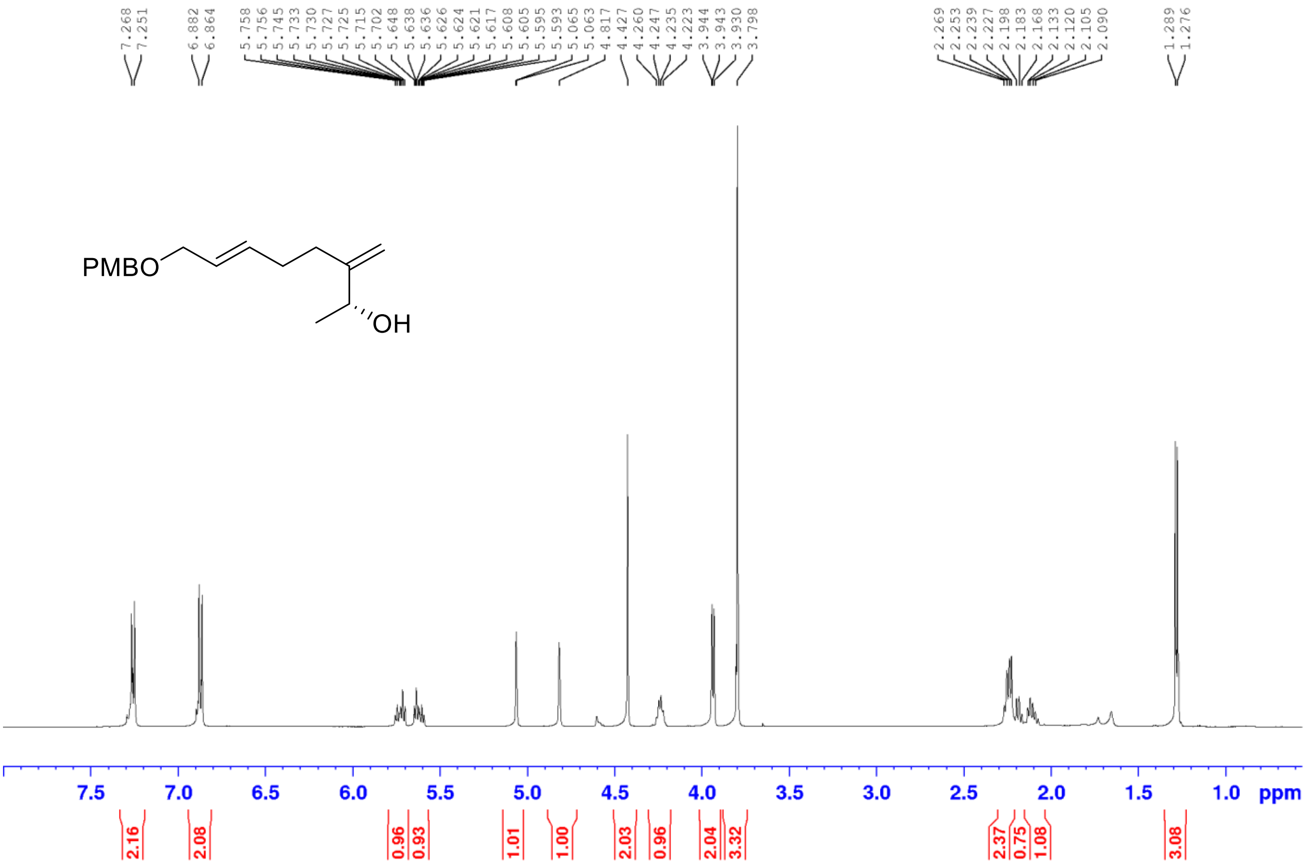

S14, $CDCl_3$, 126 MHz

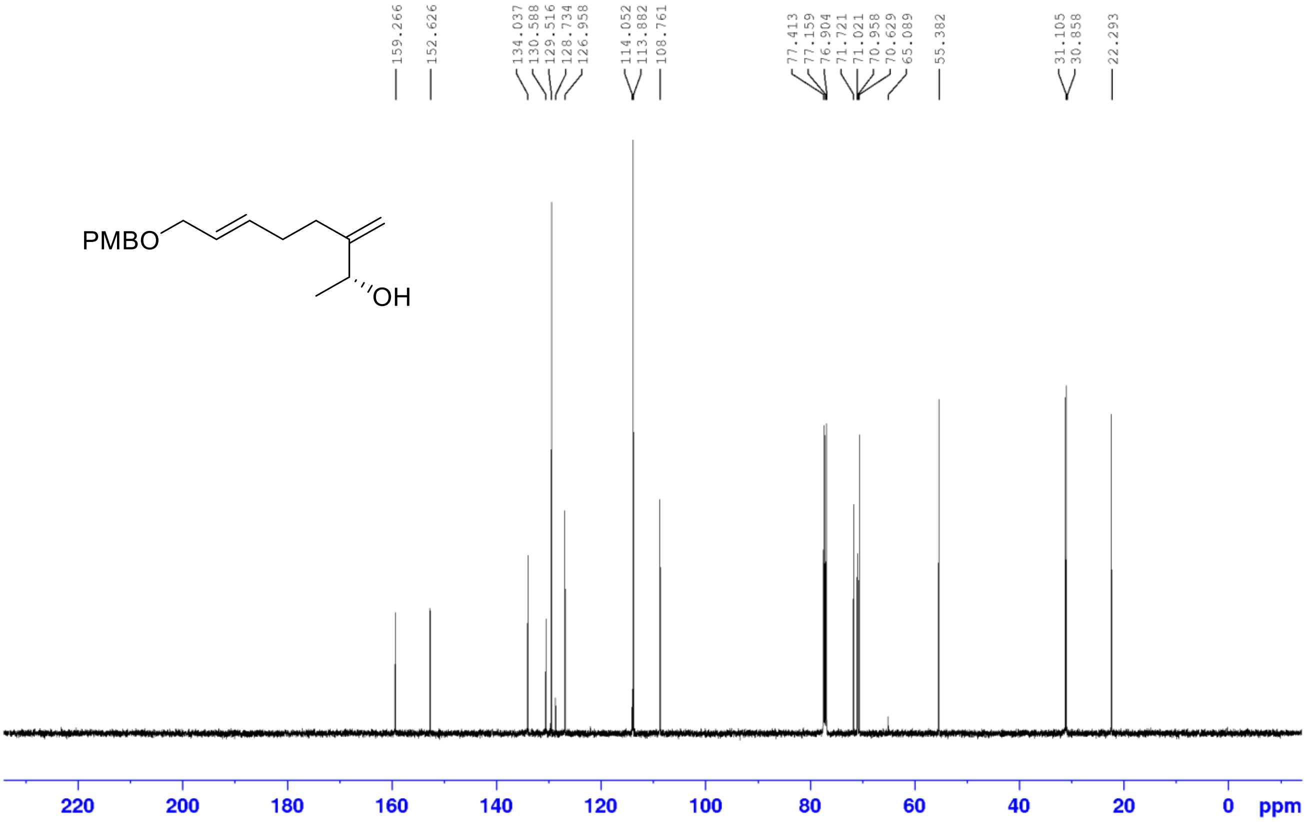

S15, $CDCl_3$, 500 MHz

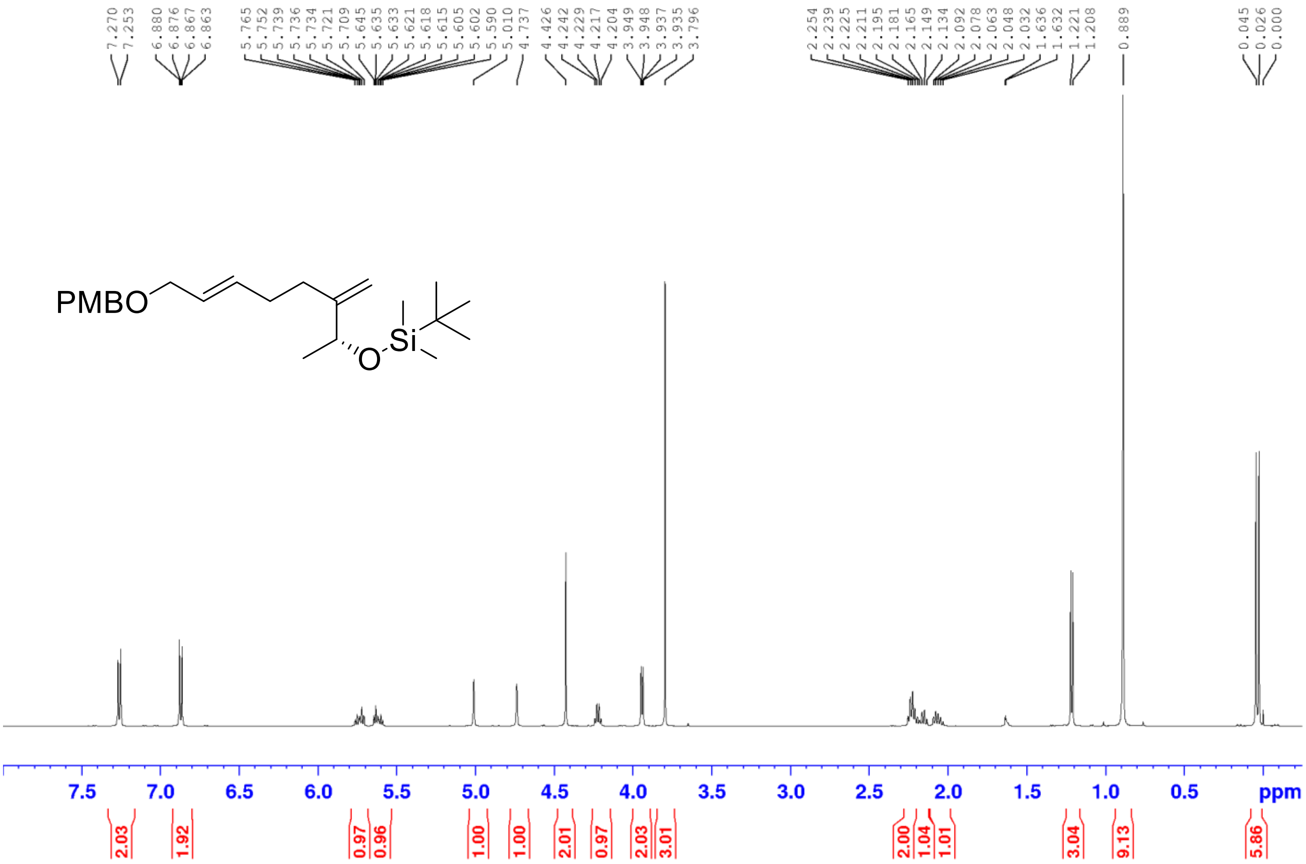

S15, $CDCl_3$, 126 MHz

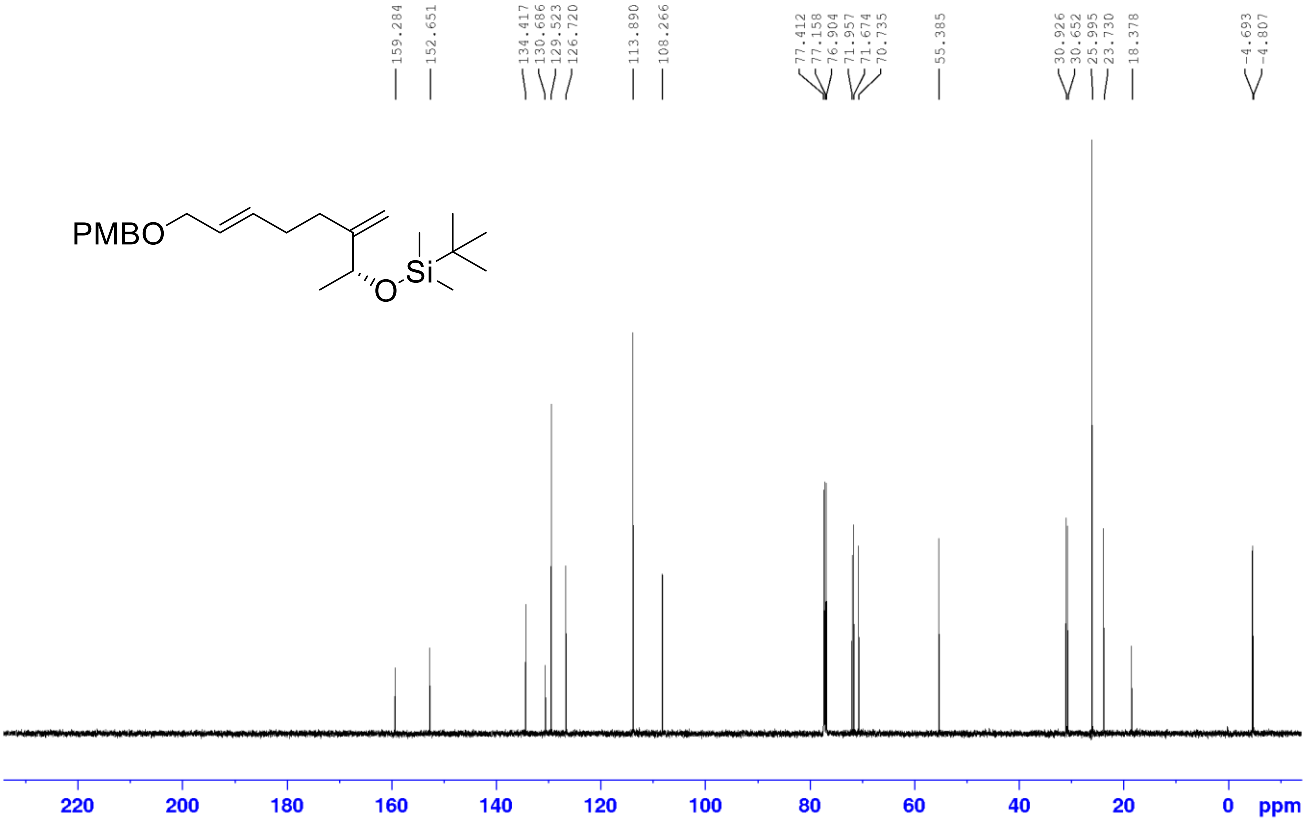

S16, $CDCl_3$, 500 MHz

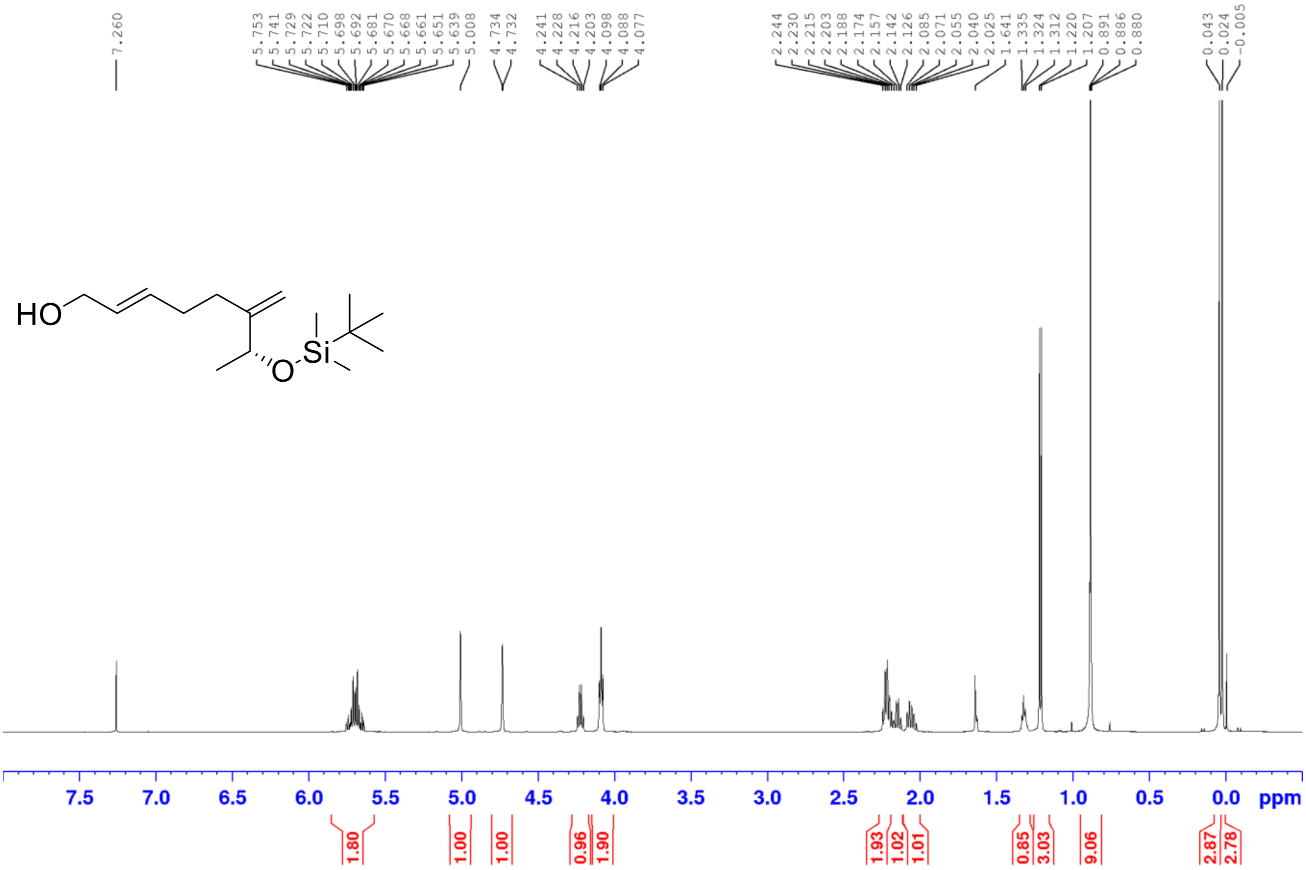

S16, $CDCl_3$, 126 MHz

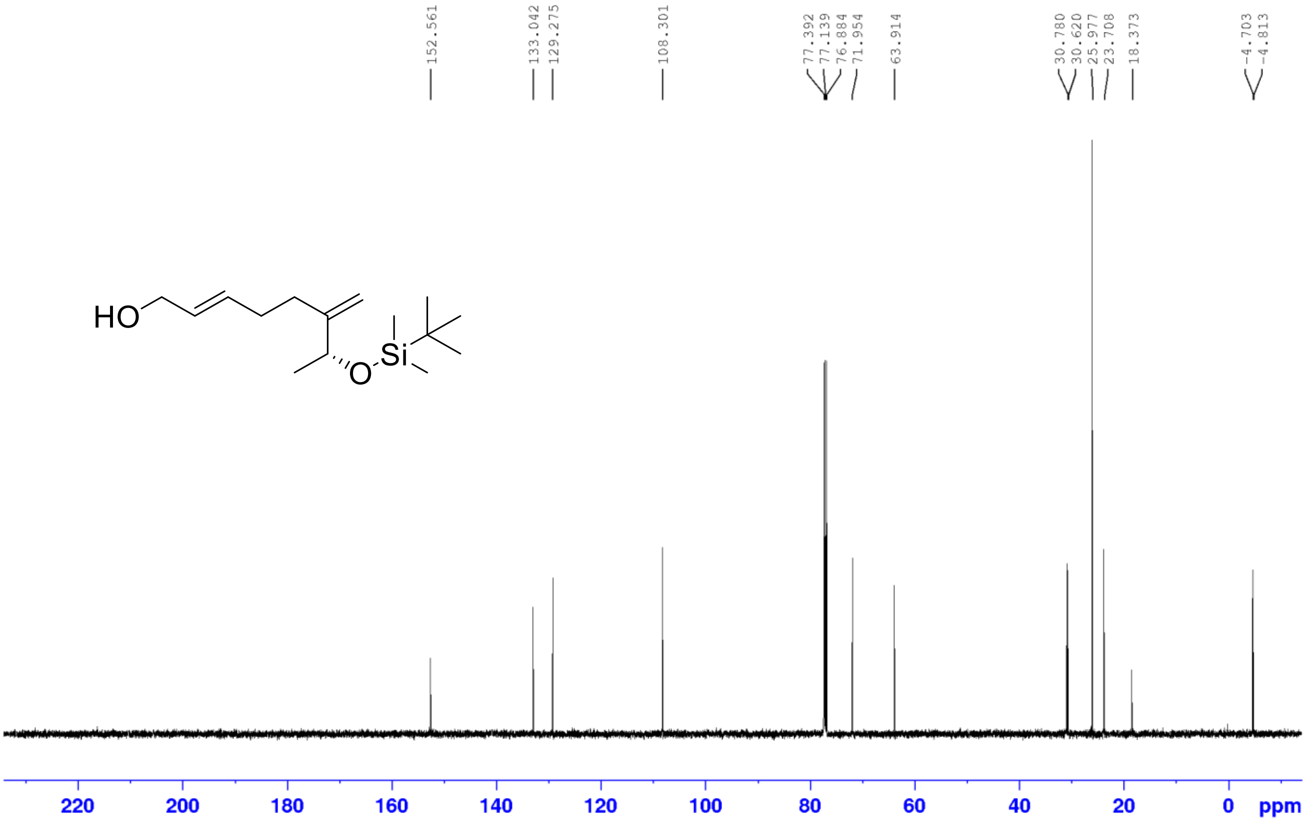

S17, $CDCl_3$, 500 MHz

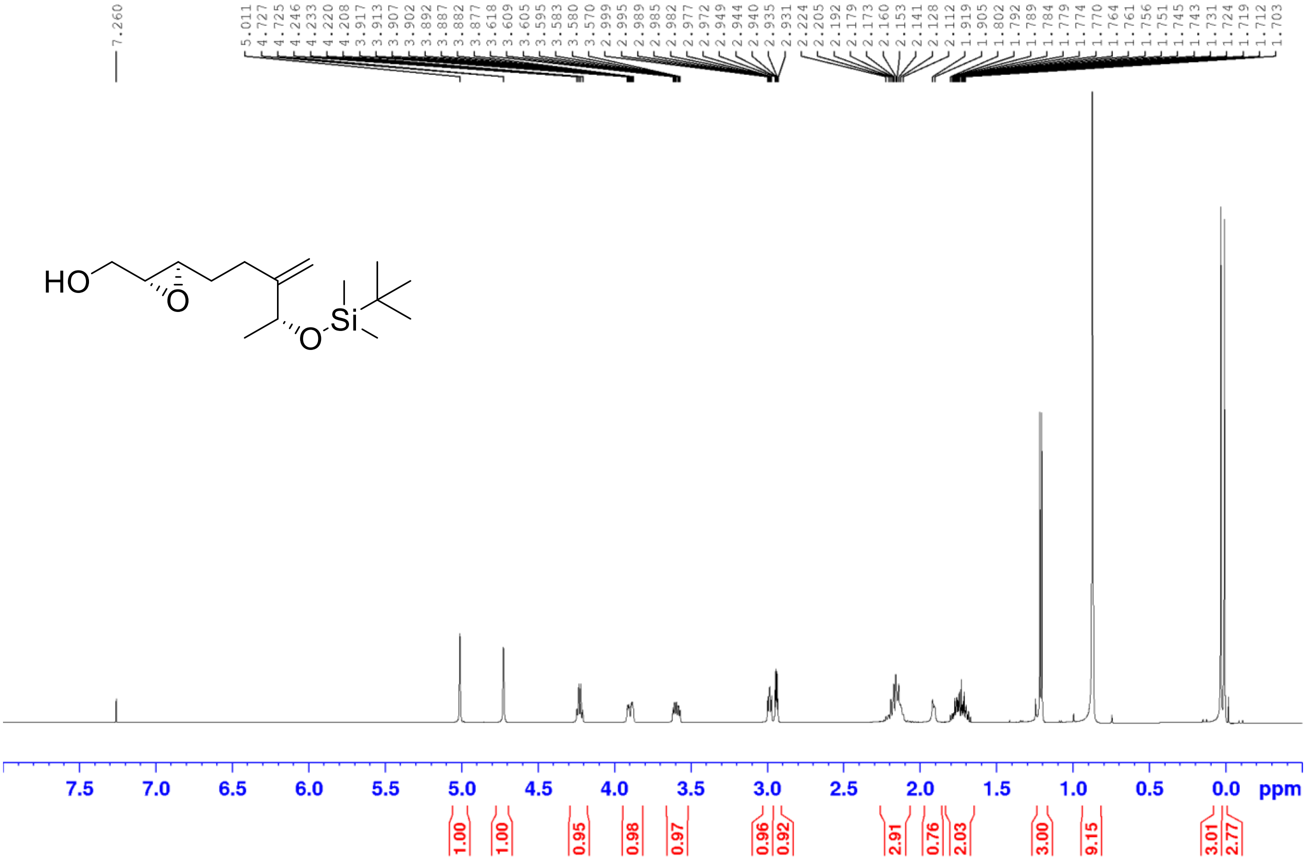

S17, $CDCl_3$, 126 MHz

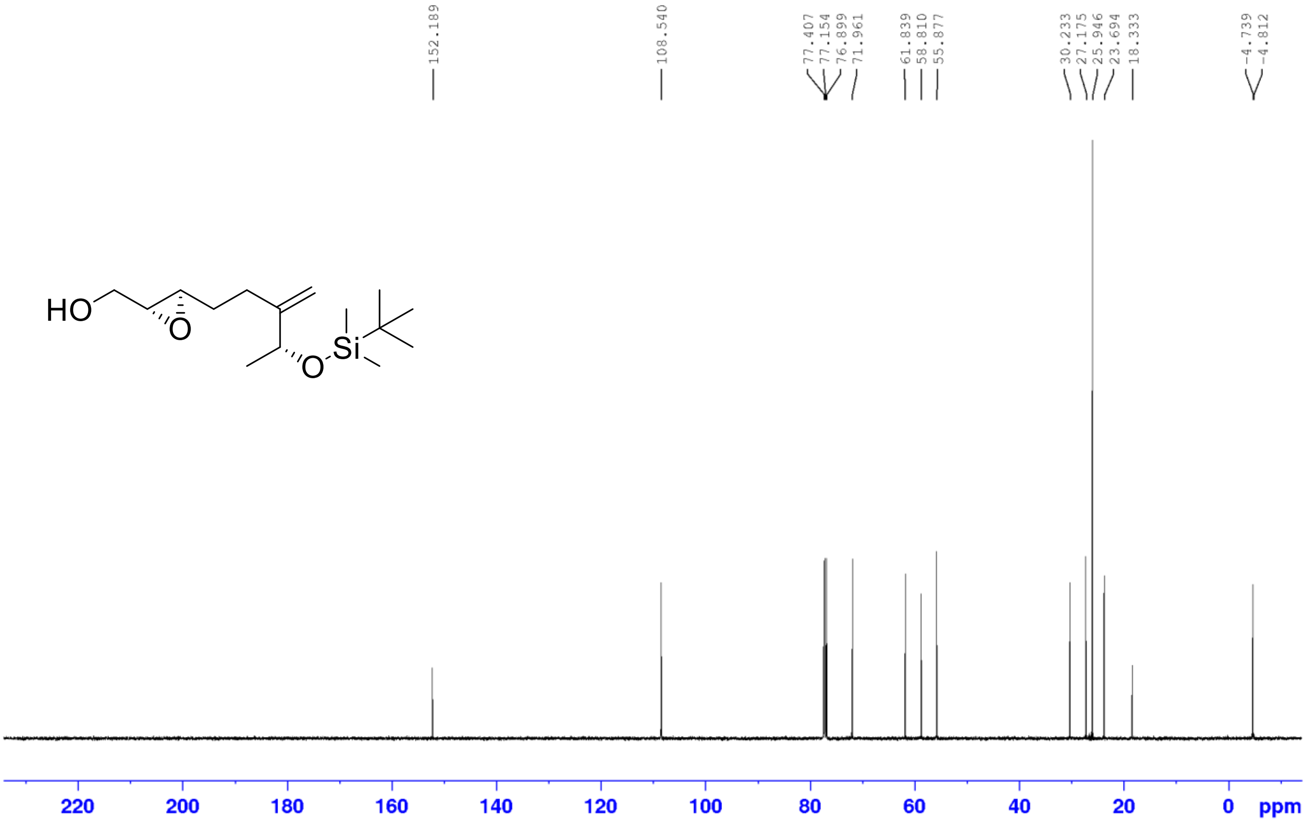

S18, $CDCl_3$, 500 MHz

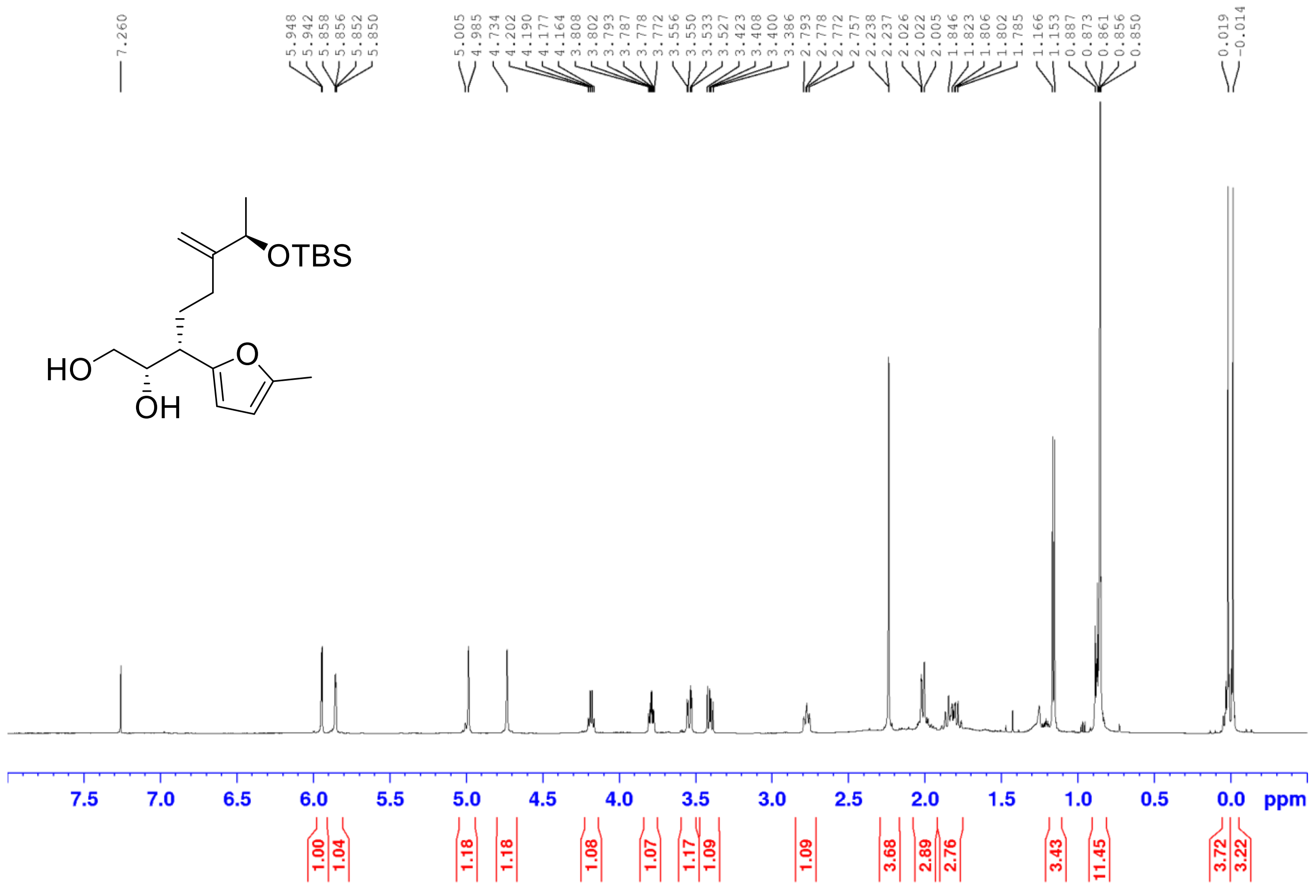

S18, $CDCl_3$, 126 MHz

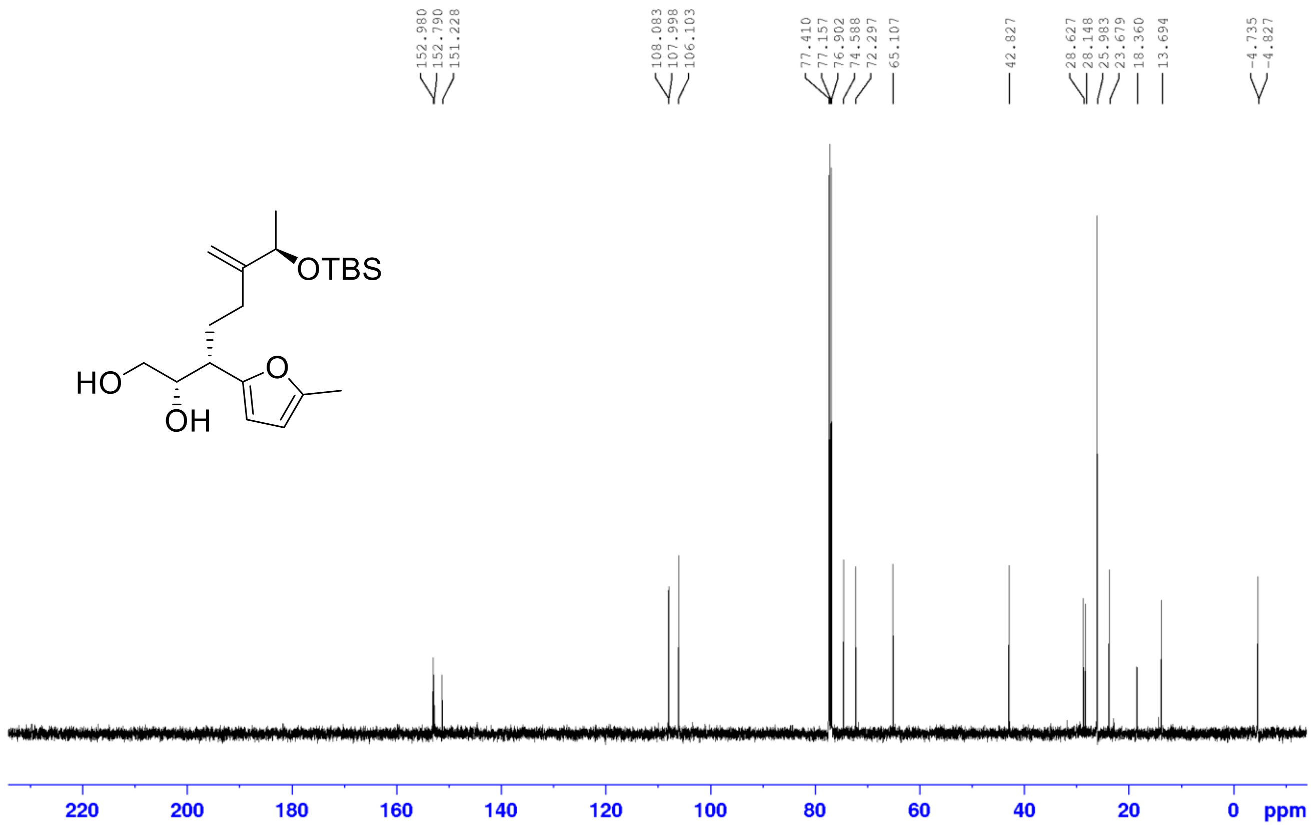

S19, $CDCl_3$, 500 MHz

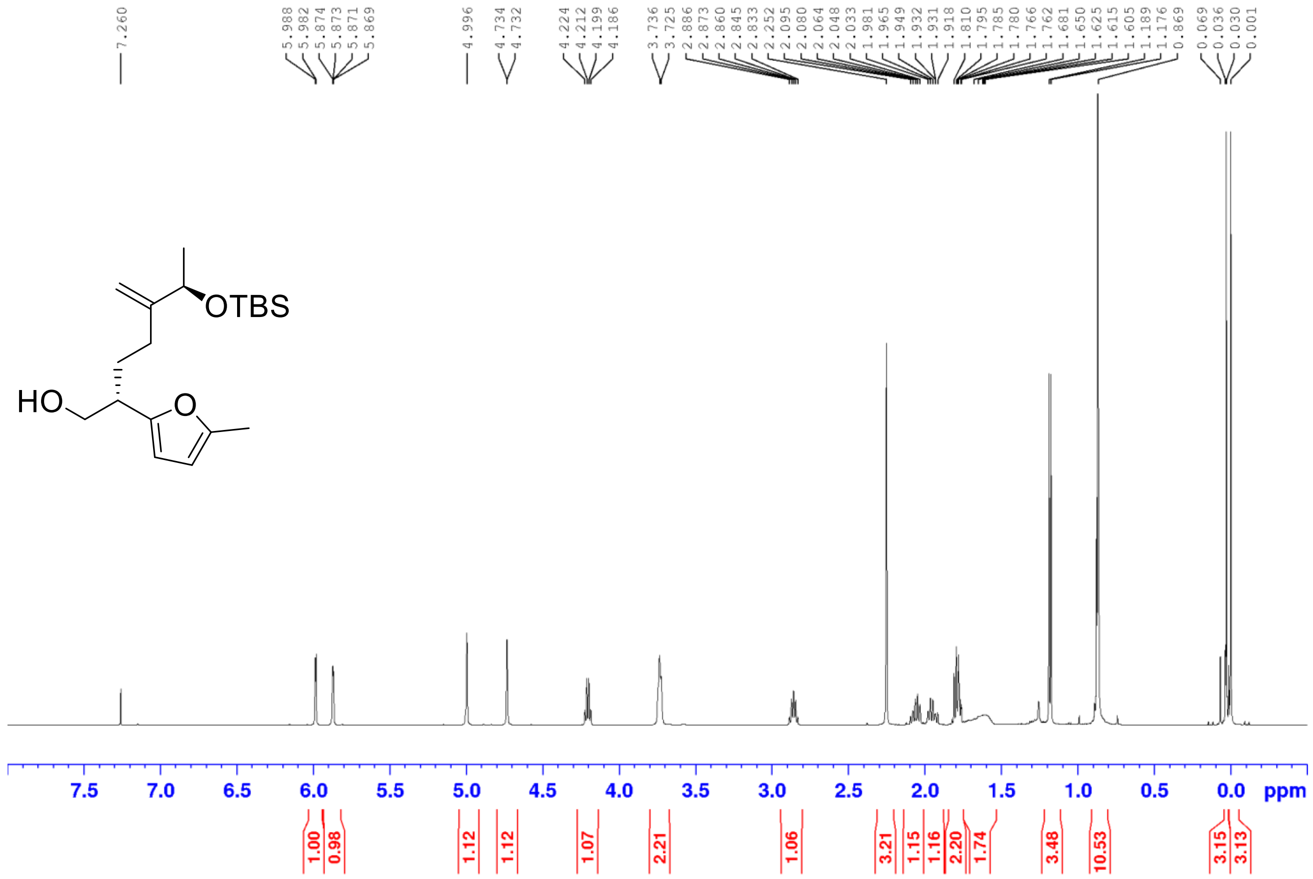

S19, $CDCl_3$, 126 MHz

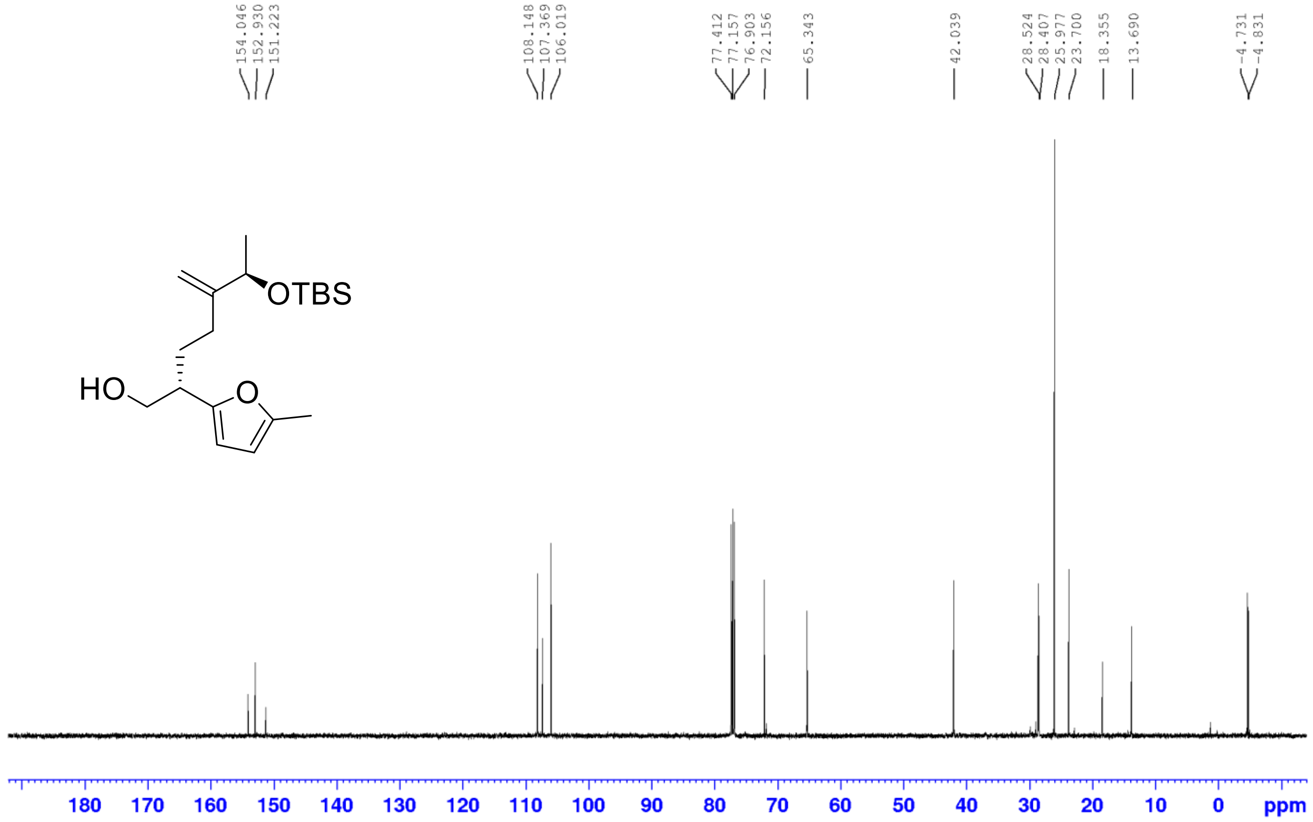

S20, $CDCl_3$, 500 MHz

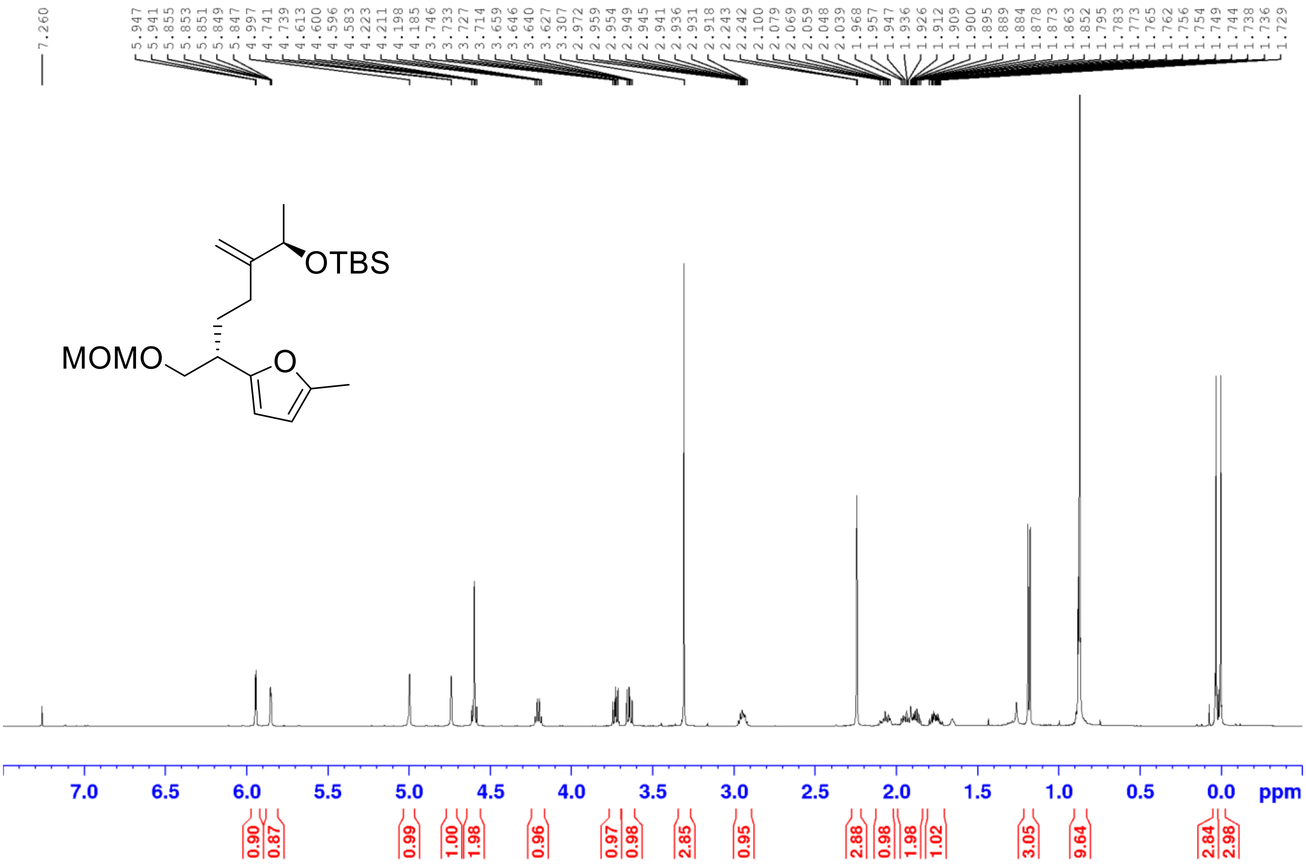

S20, $CDCl_3$, 126 MHz

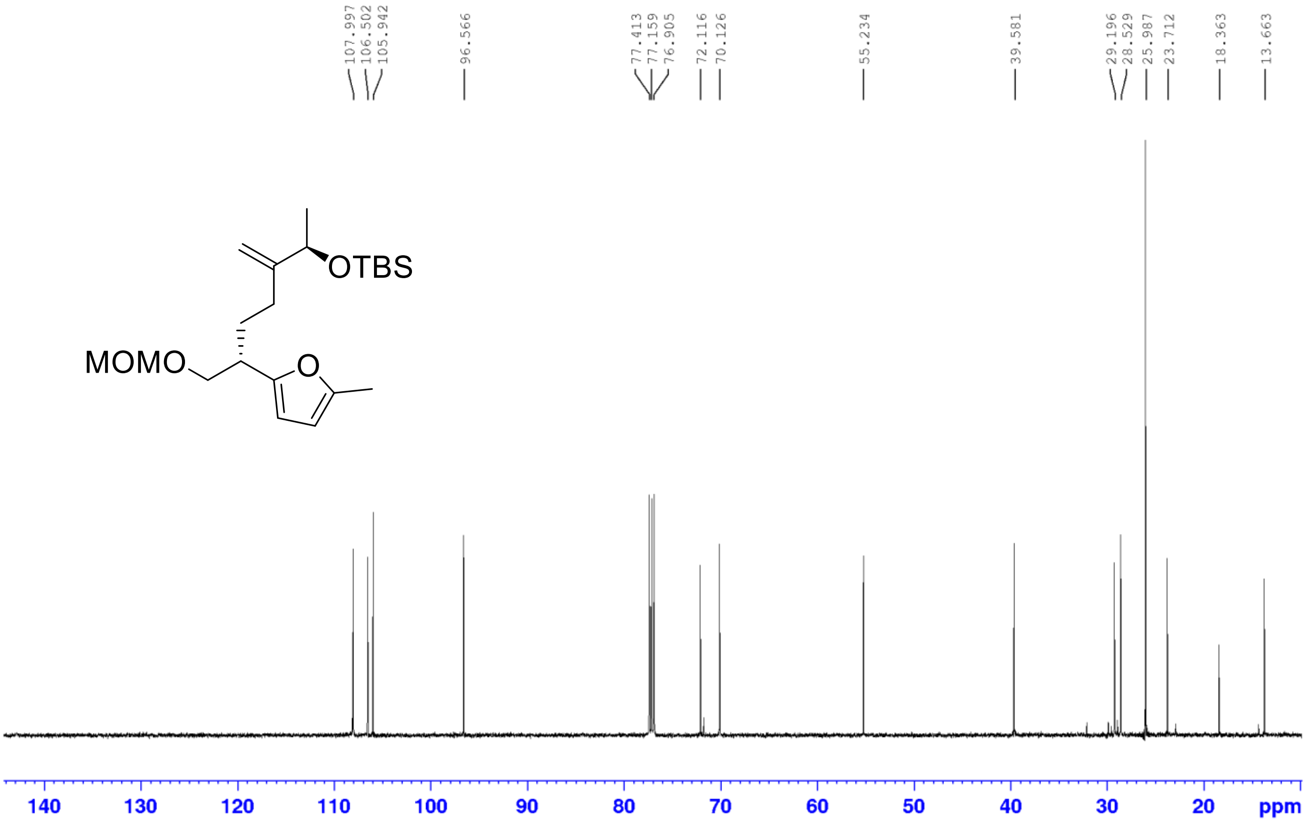

S21, $CDCl_3$, 400 MHz

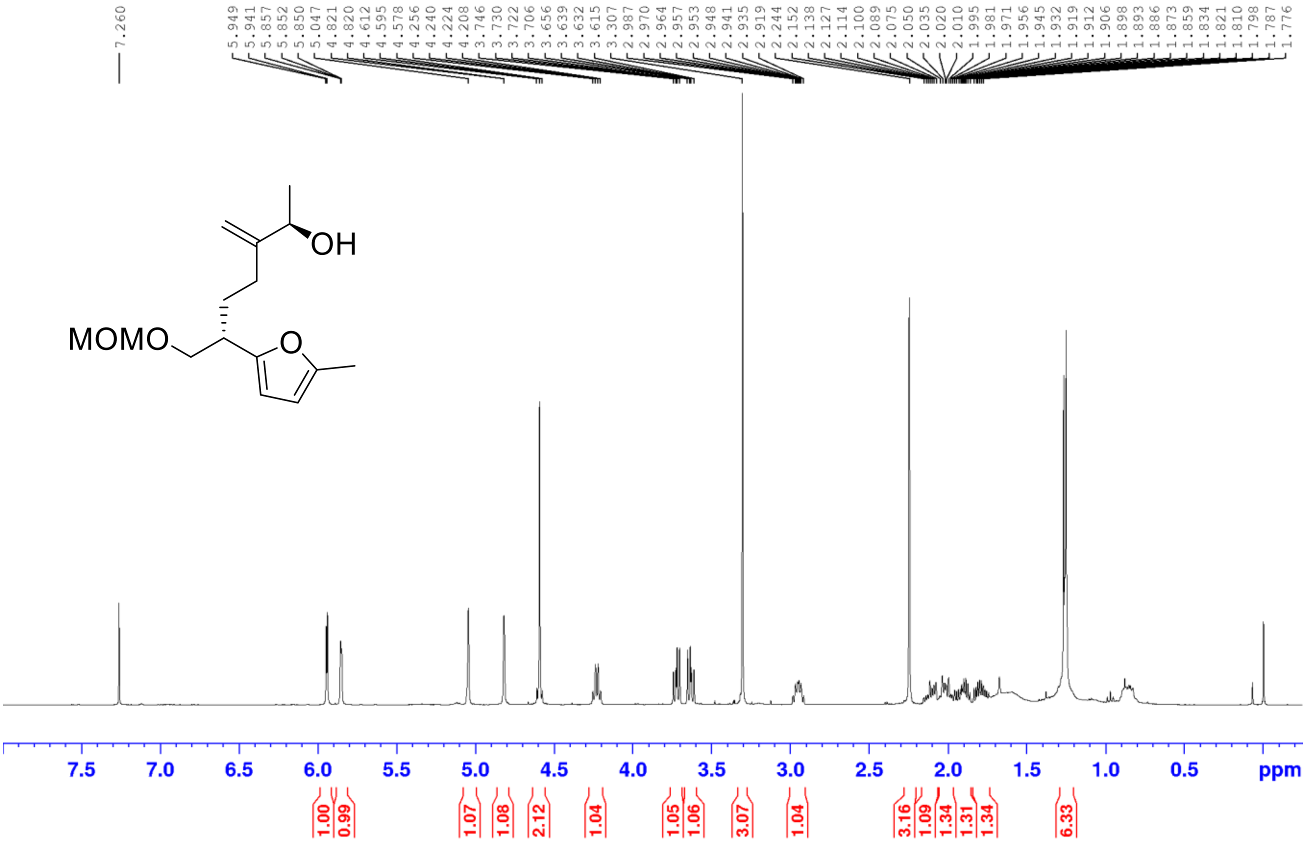

S21, $CDCl_3$, 100 MHz

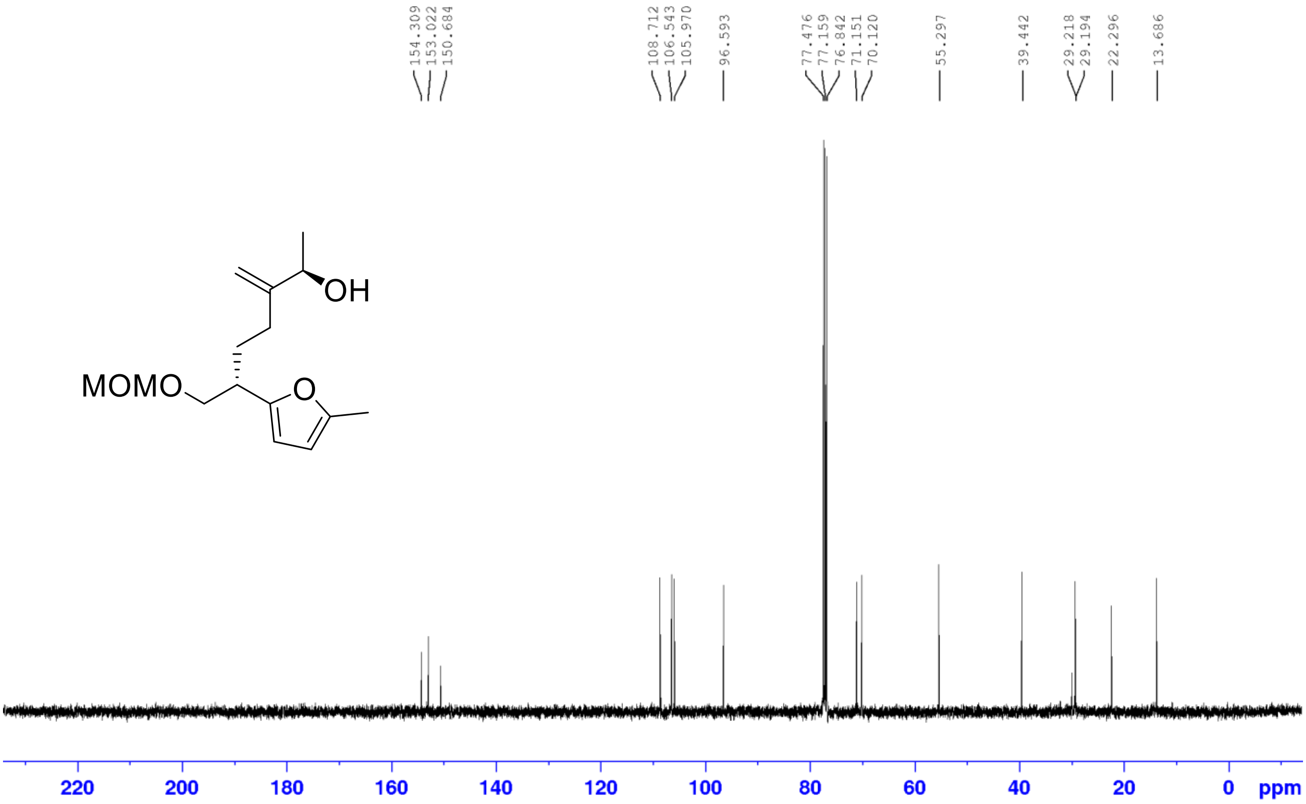

S22, $CDCl_3$, 500 MHz

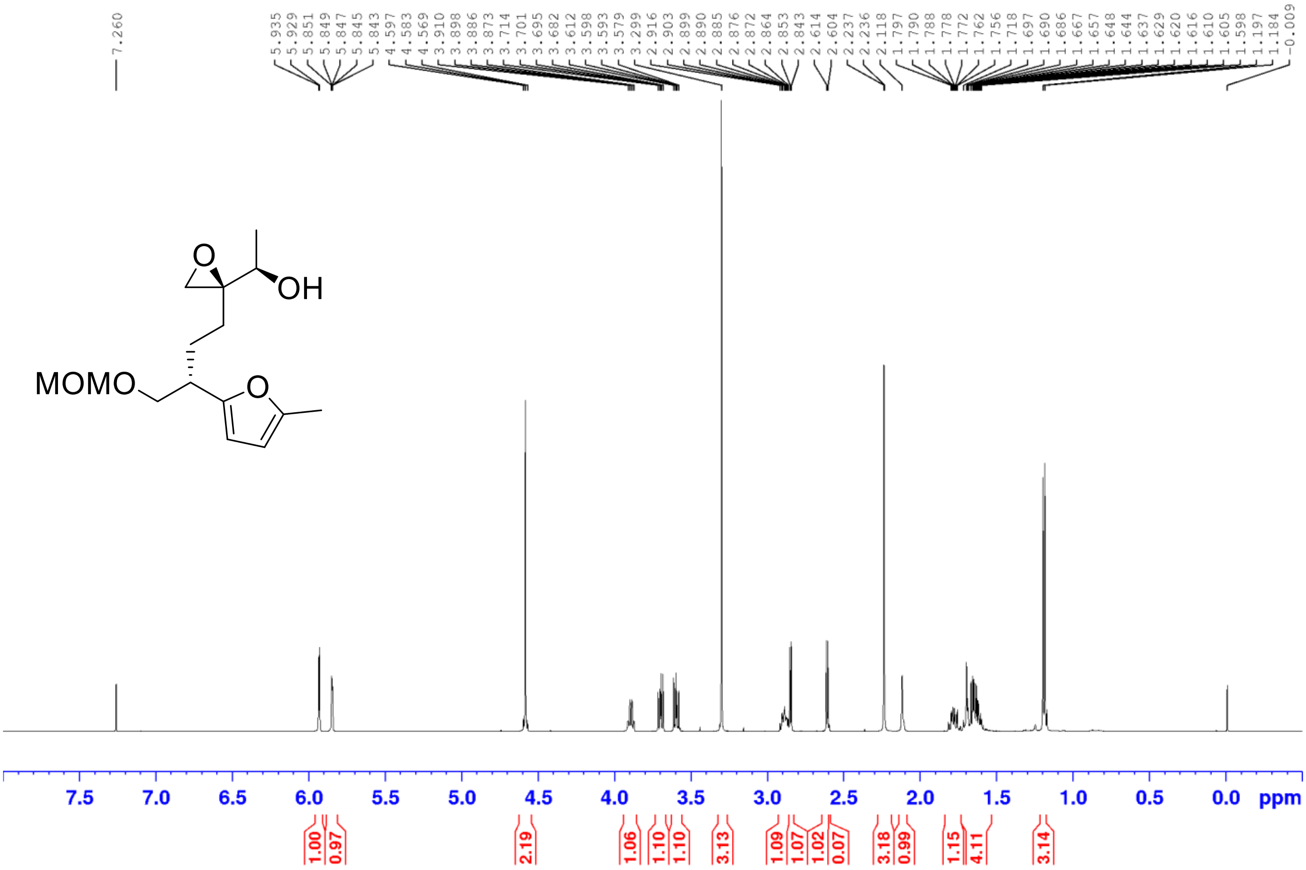

S22, $CDCl_3$, 126 MHz

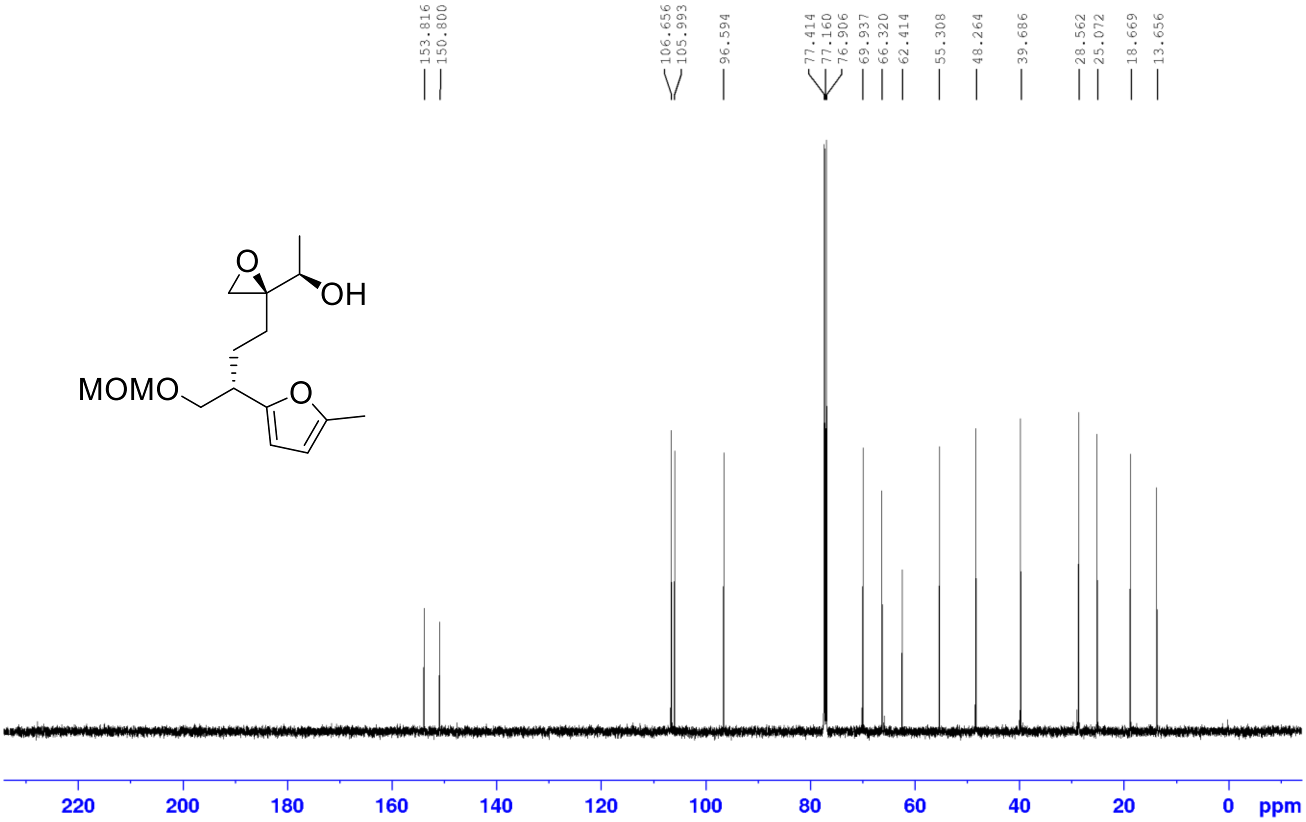

19, $CDCl_3$, 300 MHz

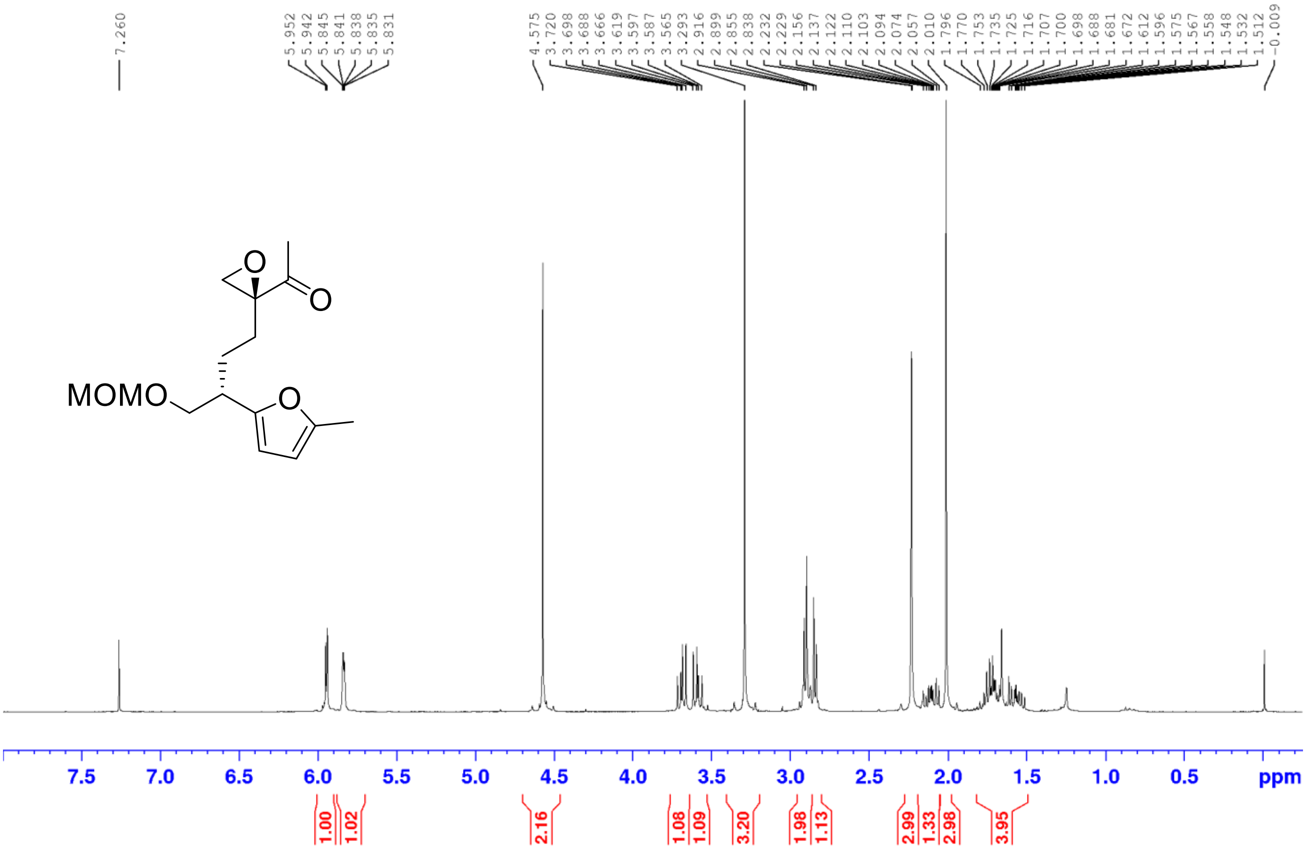

19, $CDCl_3$, 75 MHz

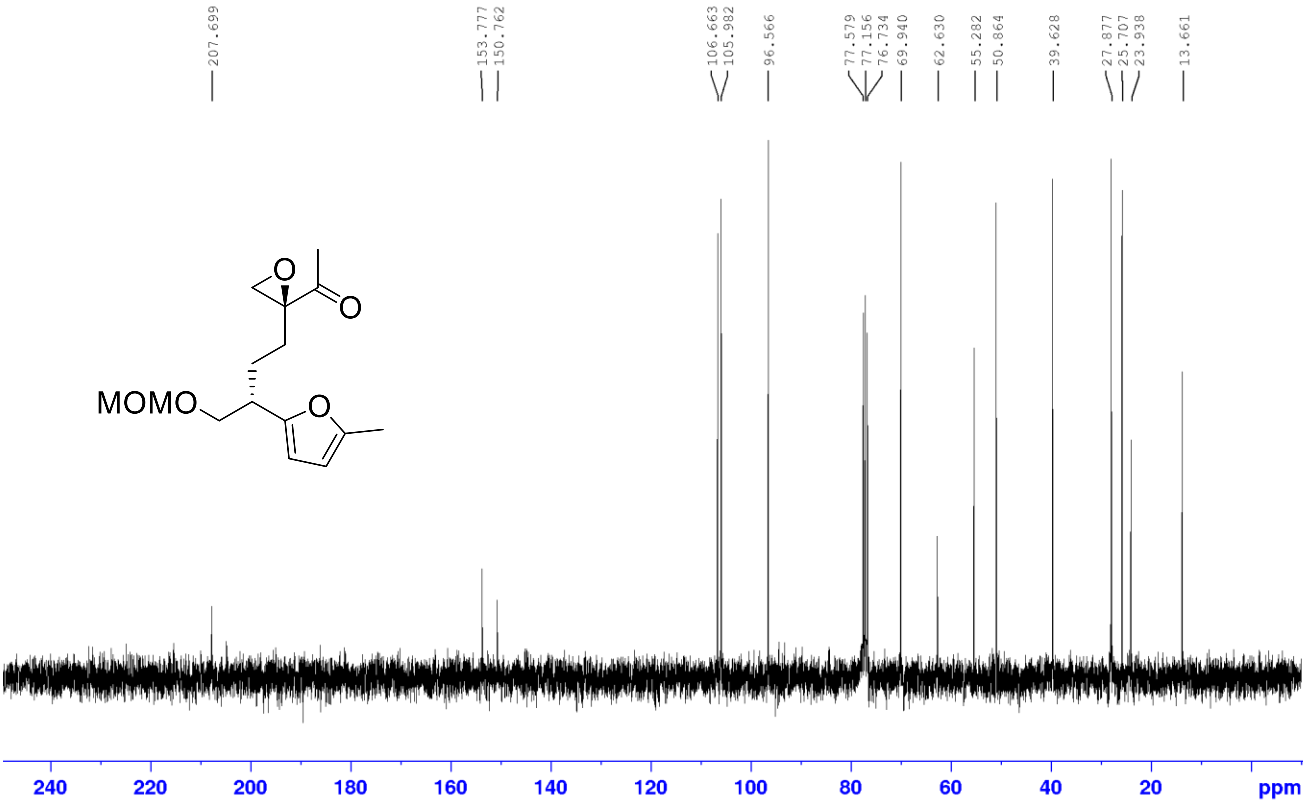

20, $CDCl_3$, 600 MHz

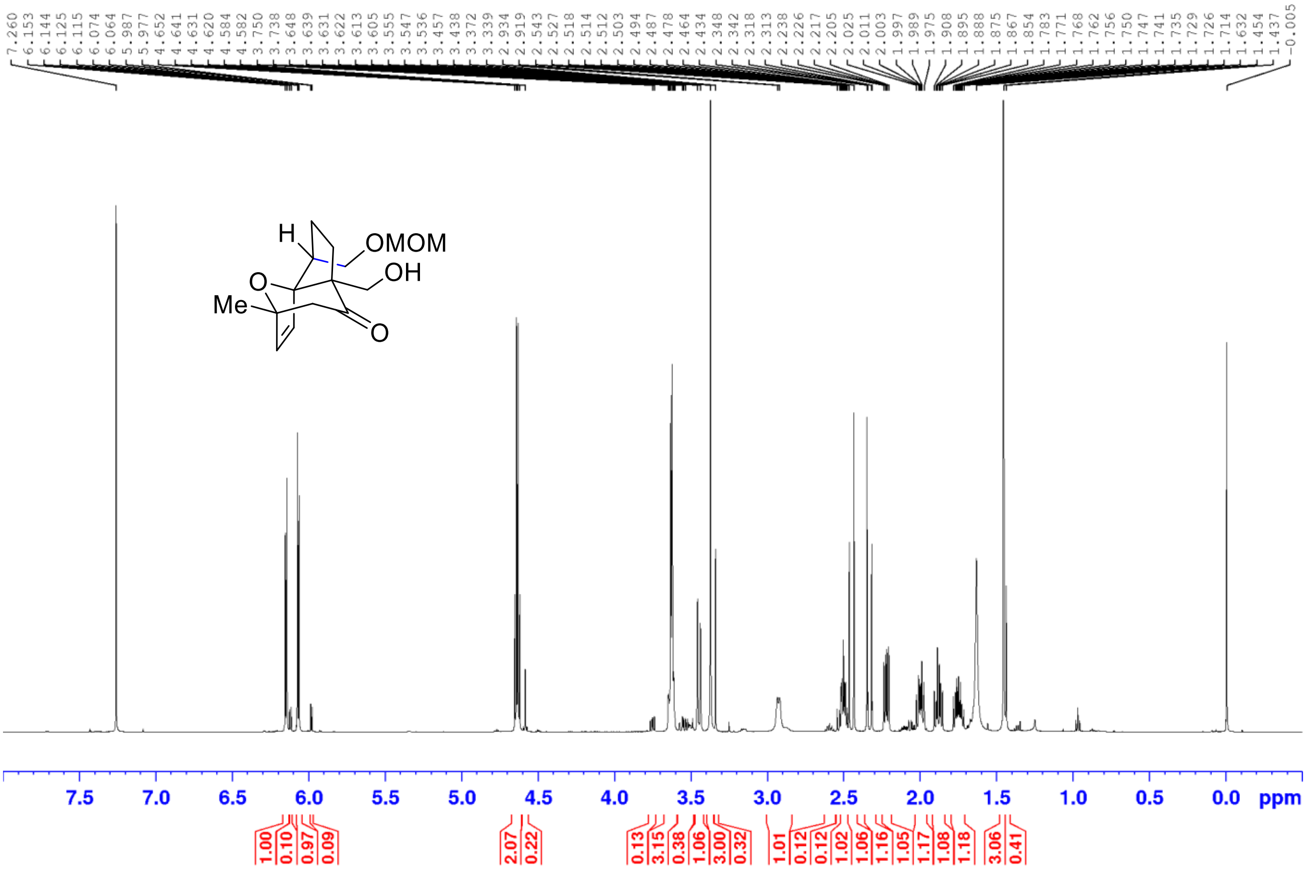

20, $CDCl_3$, 151 MHz

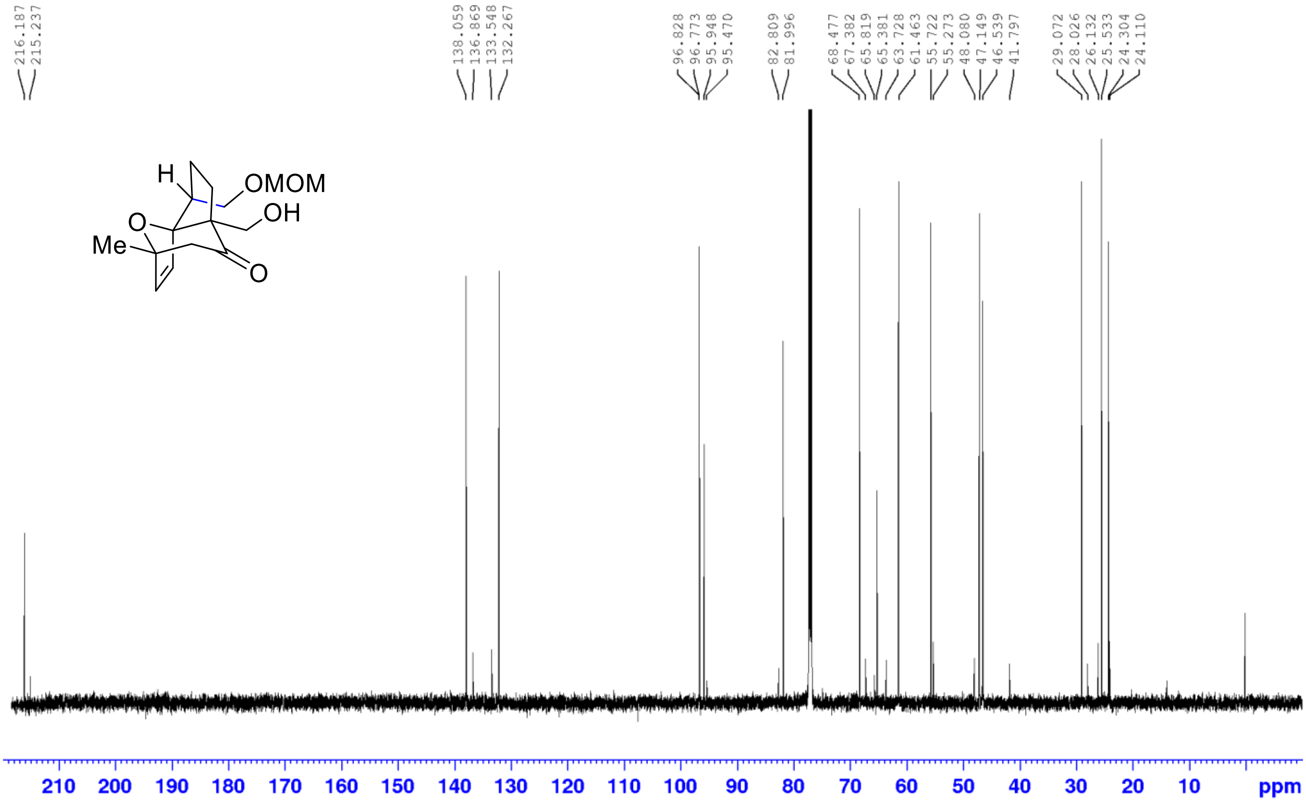

20, NOESY

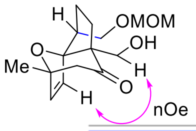
H
OMOM
OH
O
Me
H
O
H
nOe

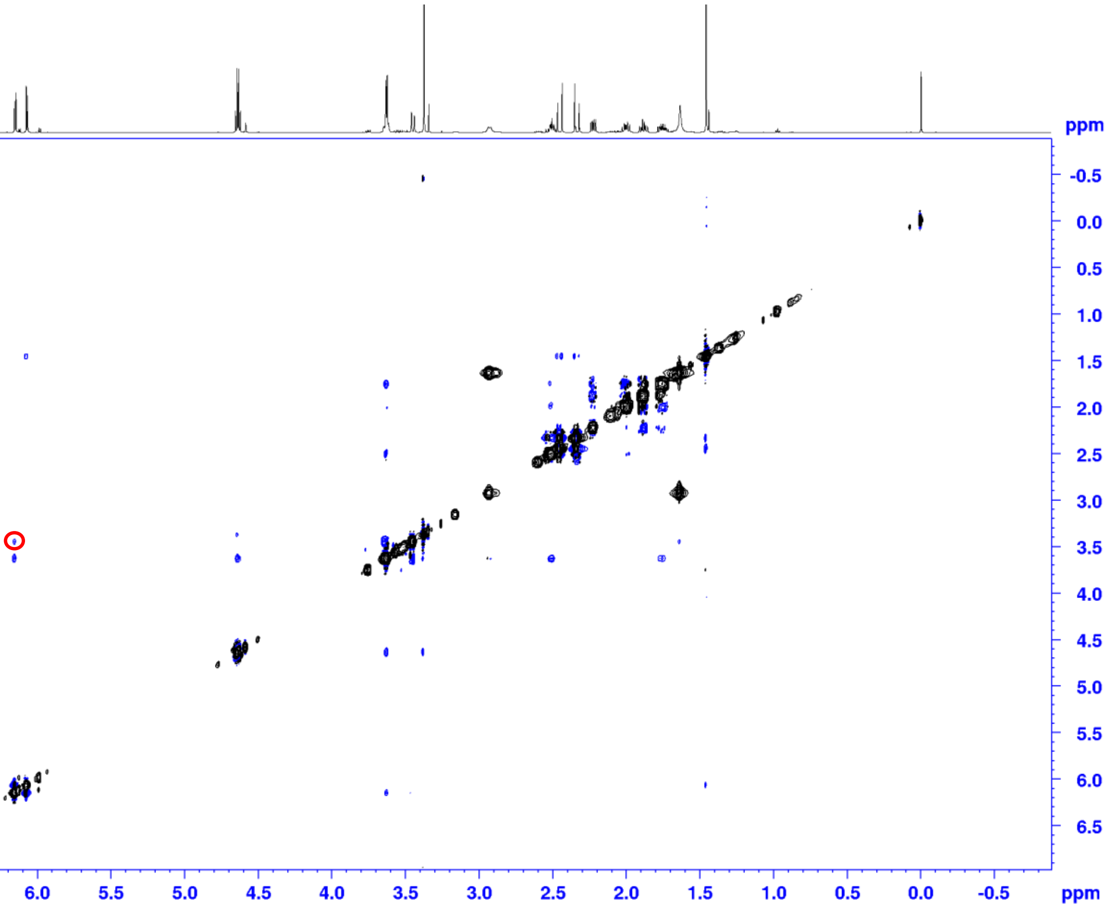
ppm
-0.5
0.0
0.5
1.0
1.5
2.0
2.5
3.0
3.5
4.0
4.5
5.0
5.5
6.0
6.5
6.5 6.0 5.5 5.0 4.5 4.0 3.5 3.0 2.5 2.0 1.5 1.0 0.5 0.0 -0.5
ppm

24, $CDCl_3$, 400 MHz

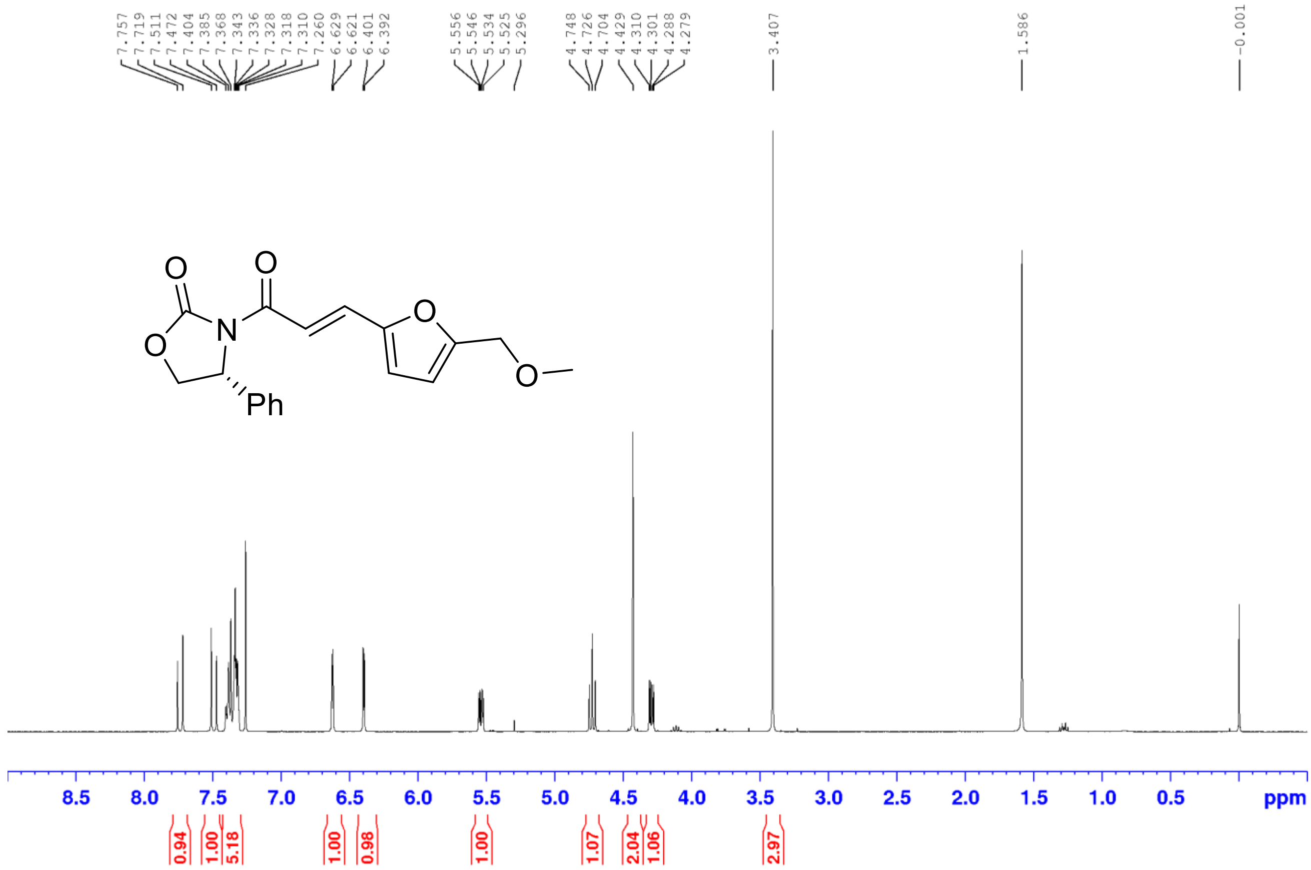

24, $CDCl_3$, 100 MHz

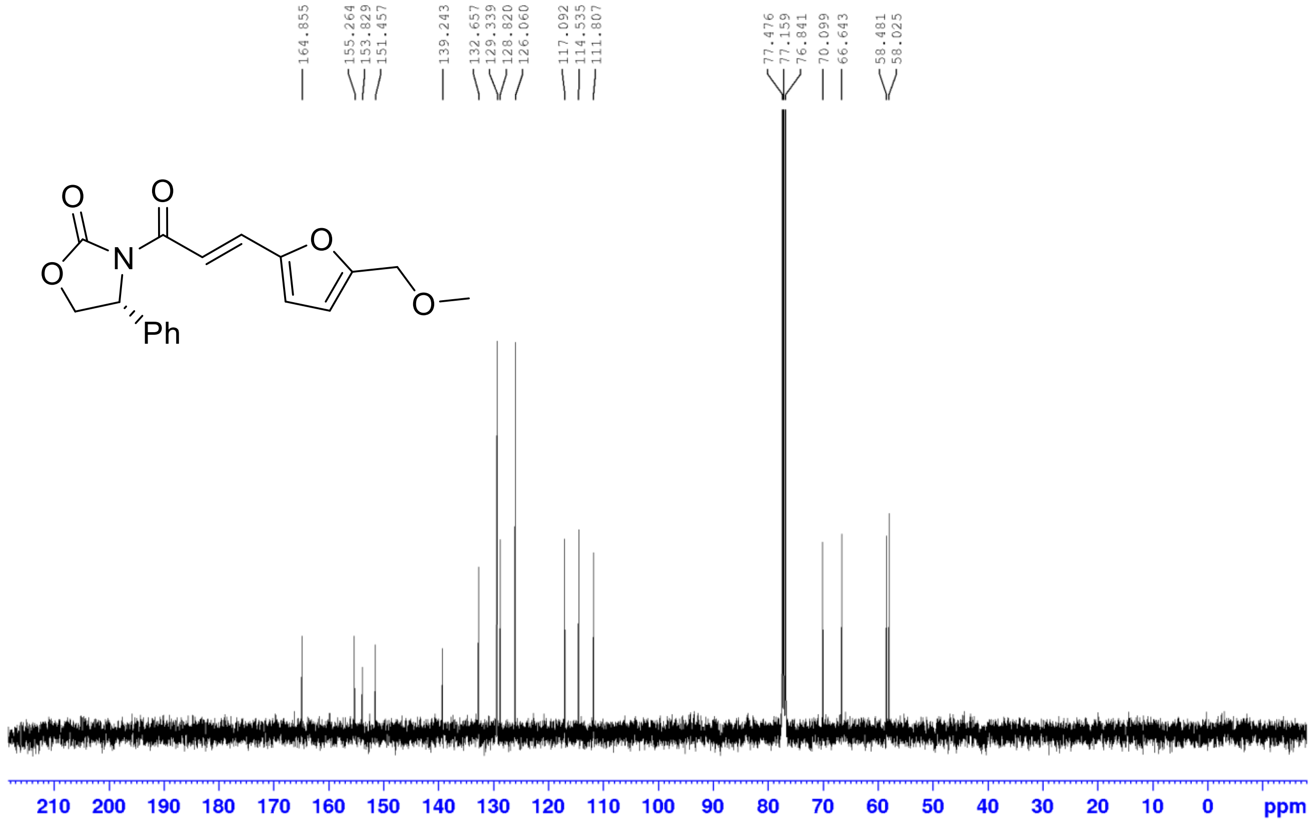

25, $CDCl_3$, 400 MHz

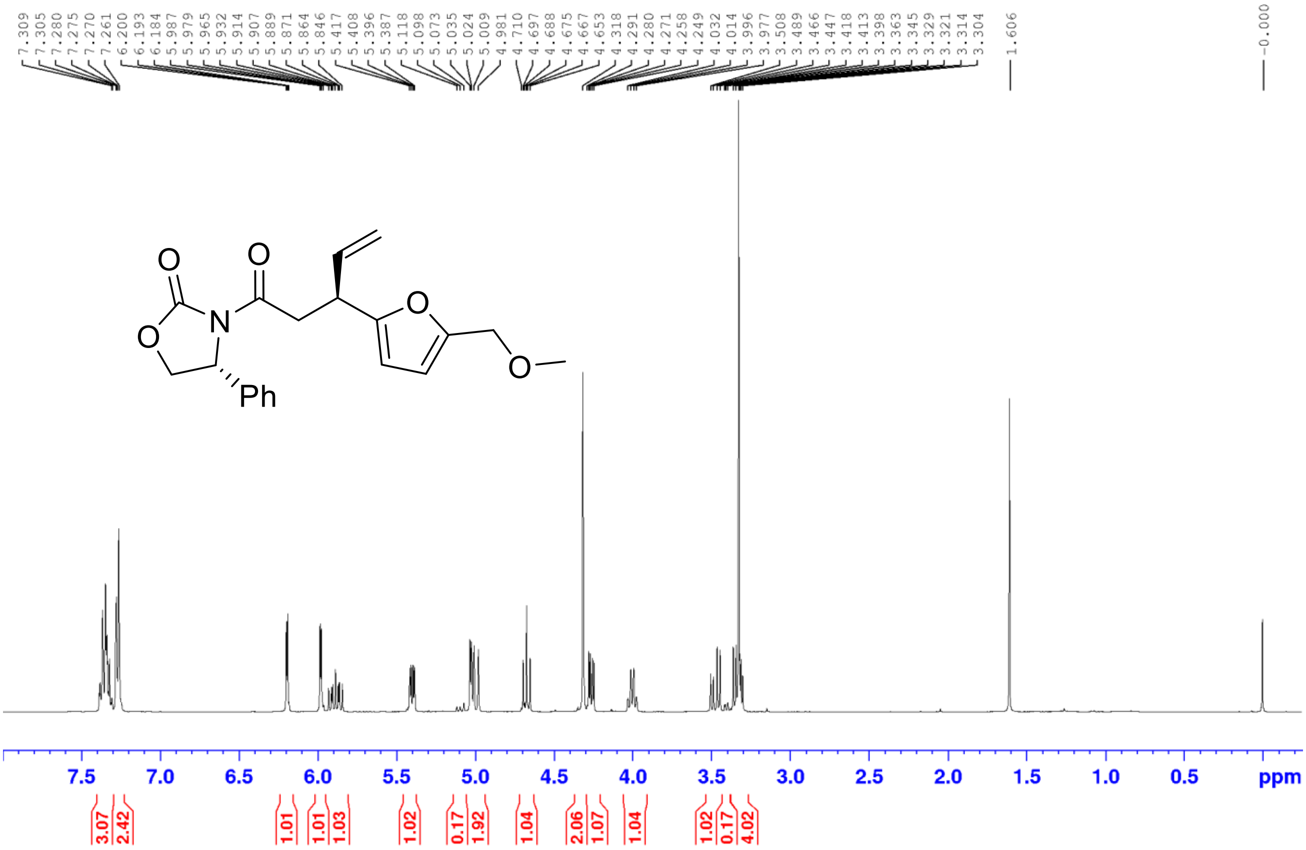

25, $CDCl_3$, 100 MHz

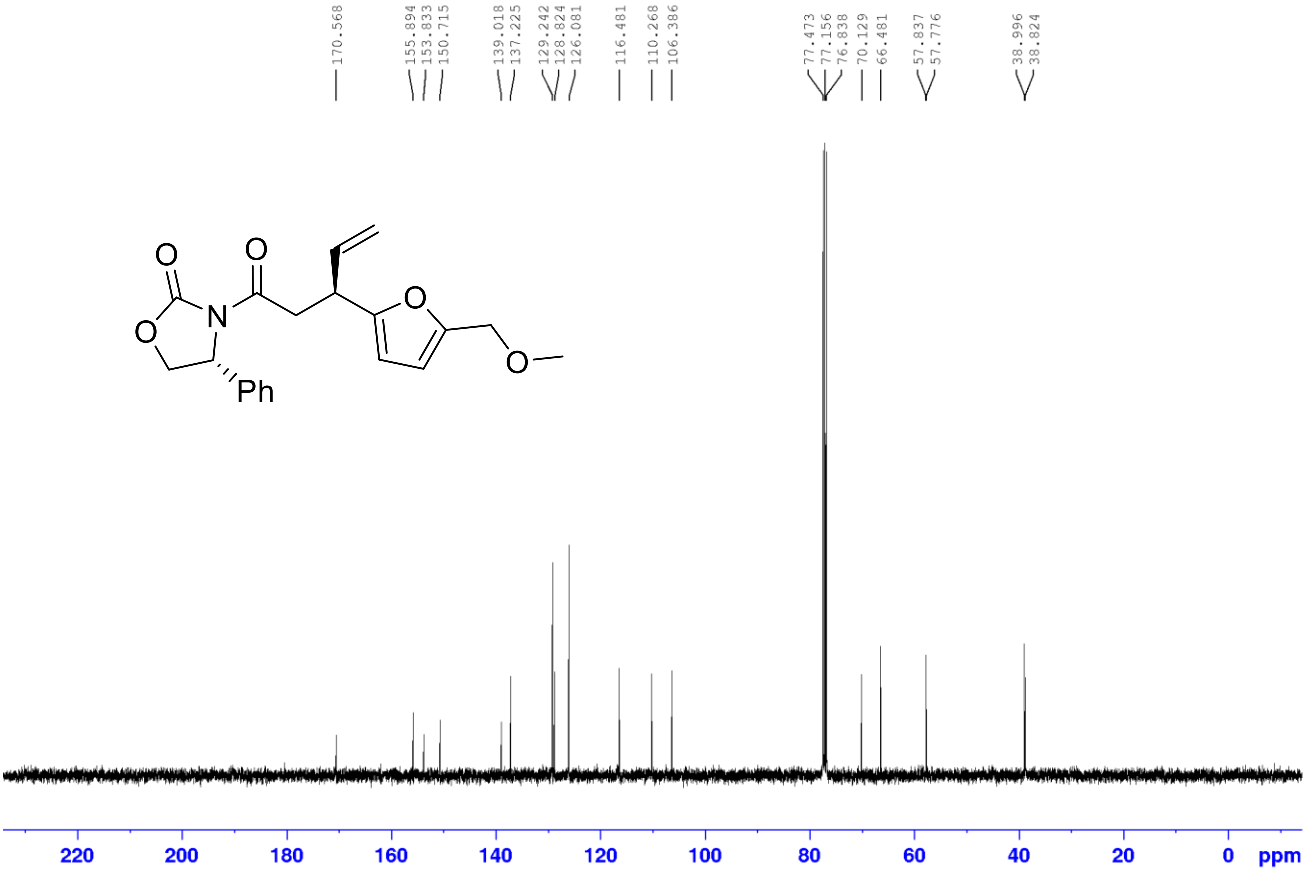

S23, $CDCl_3$, 400 MHz

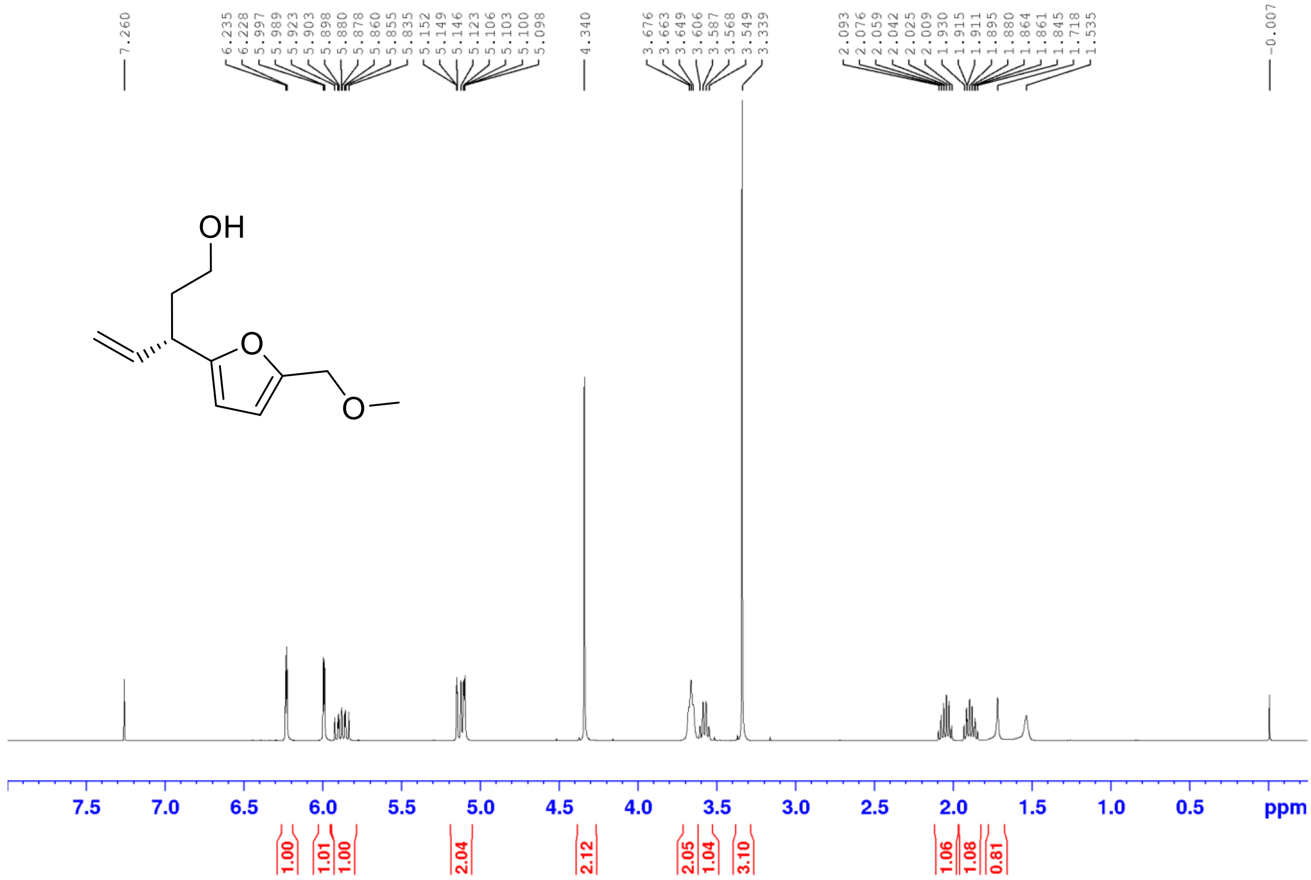

S23, $CDCl_3$, 100 MHz

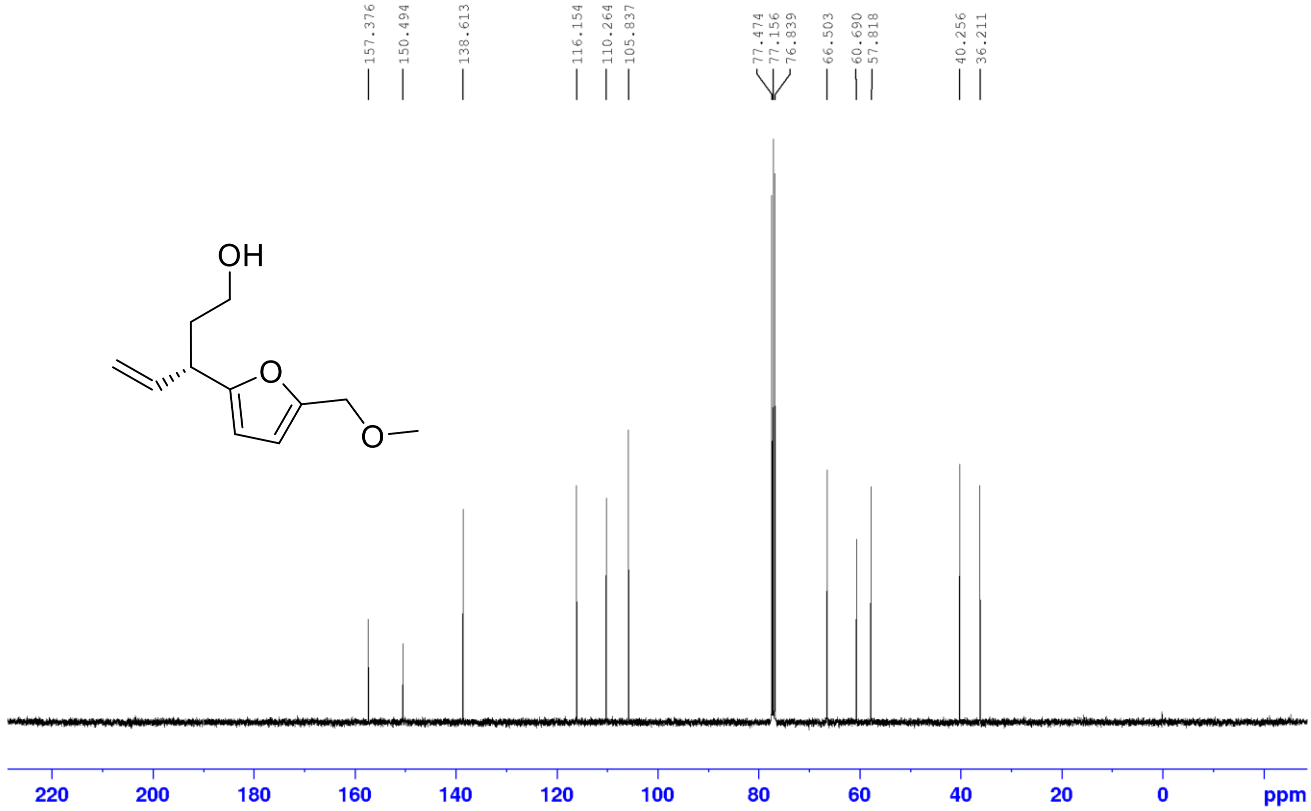

26, $CDCl_3$, 400 MHz

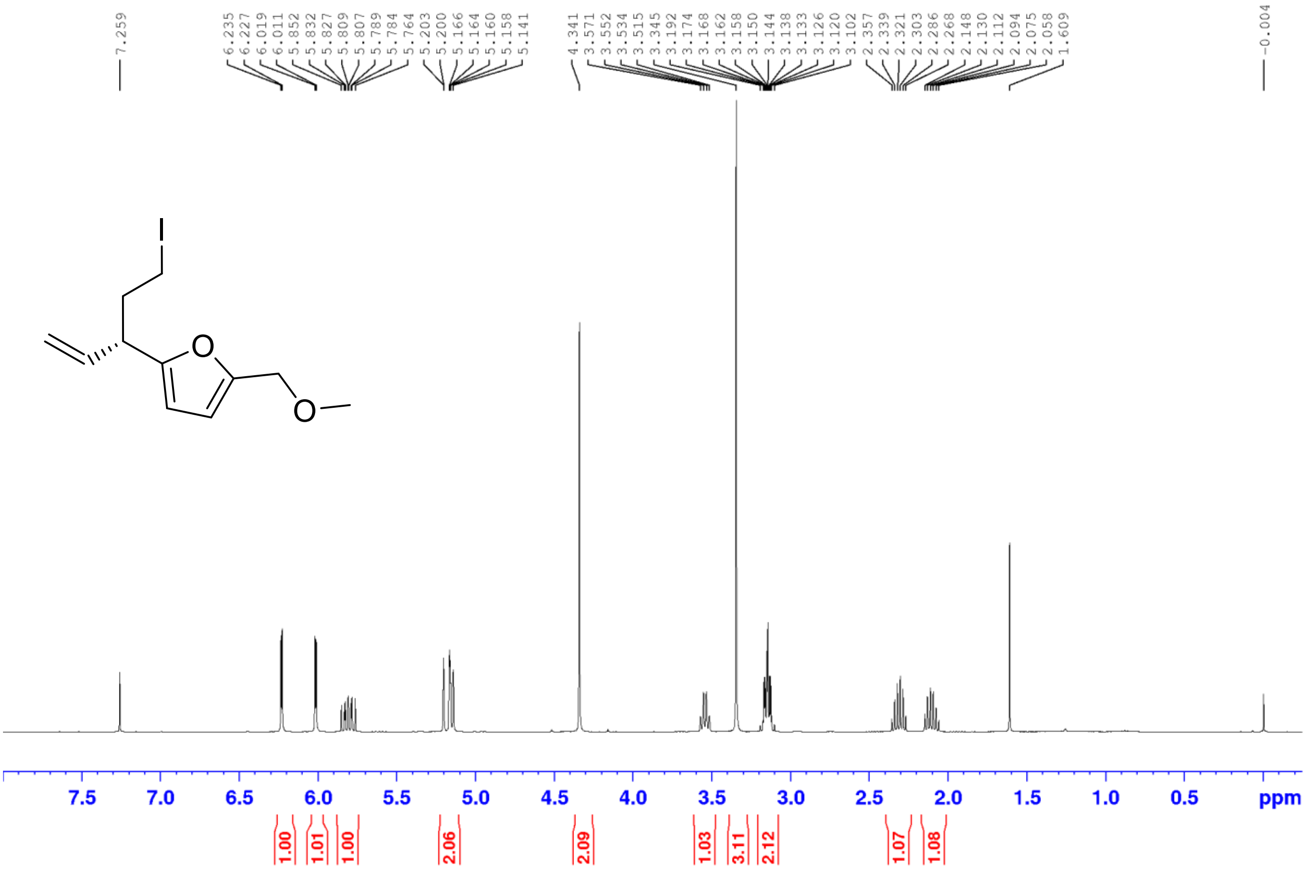

26, $CDCl_3$, 100 MHz

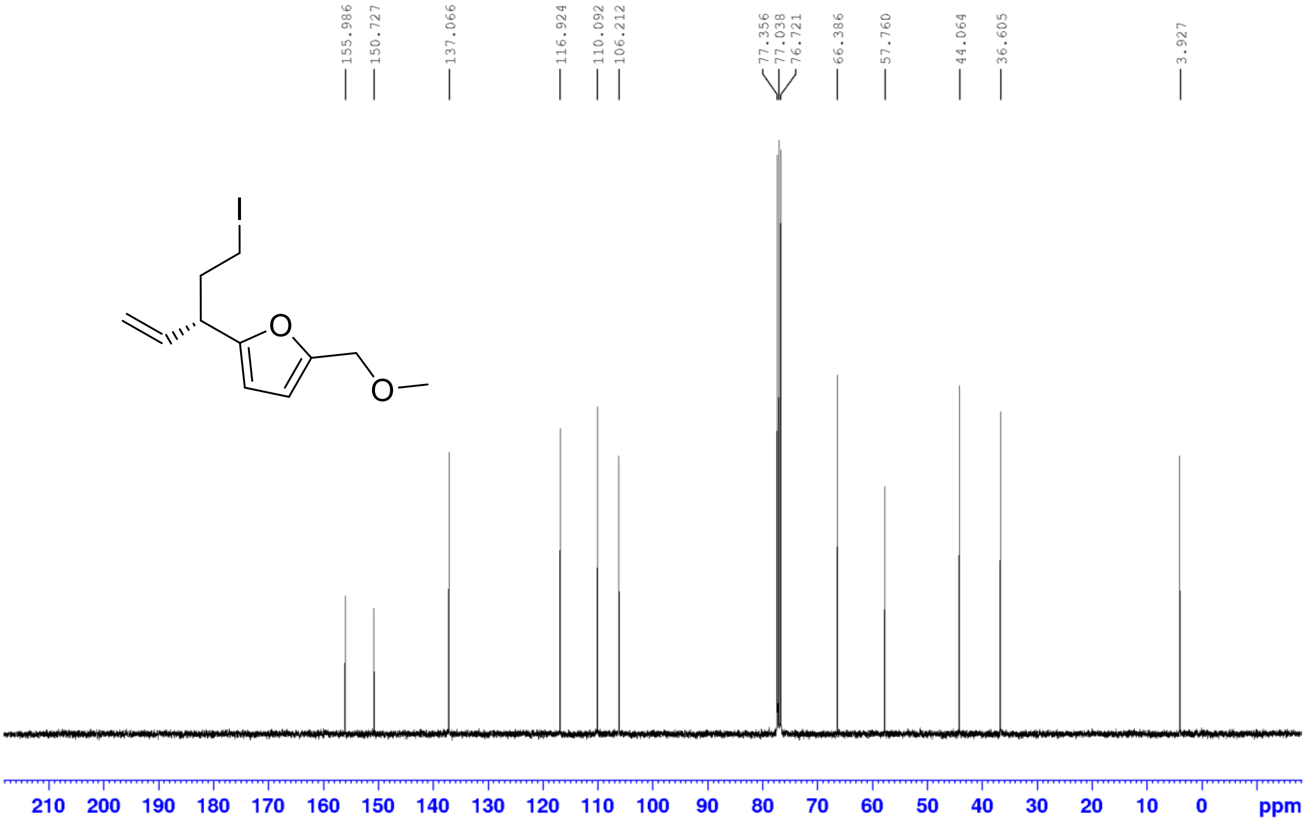

27, $CDCl_3$, 500 MHz

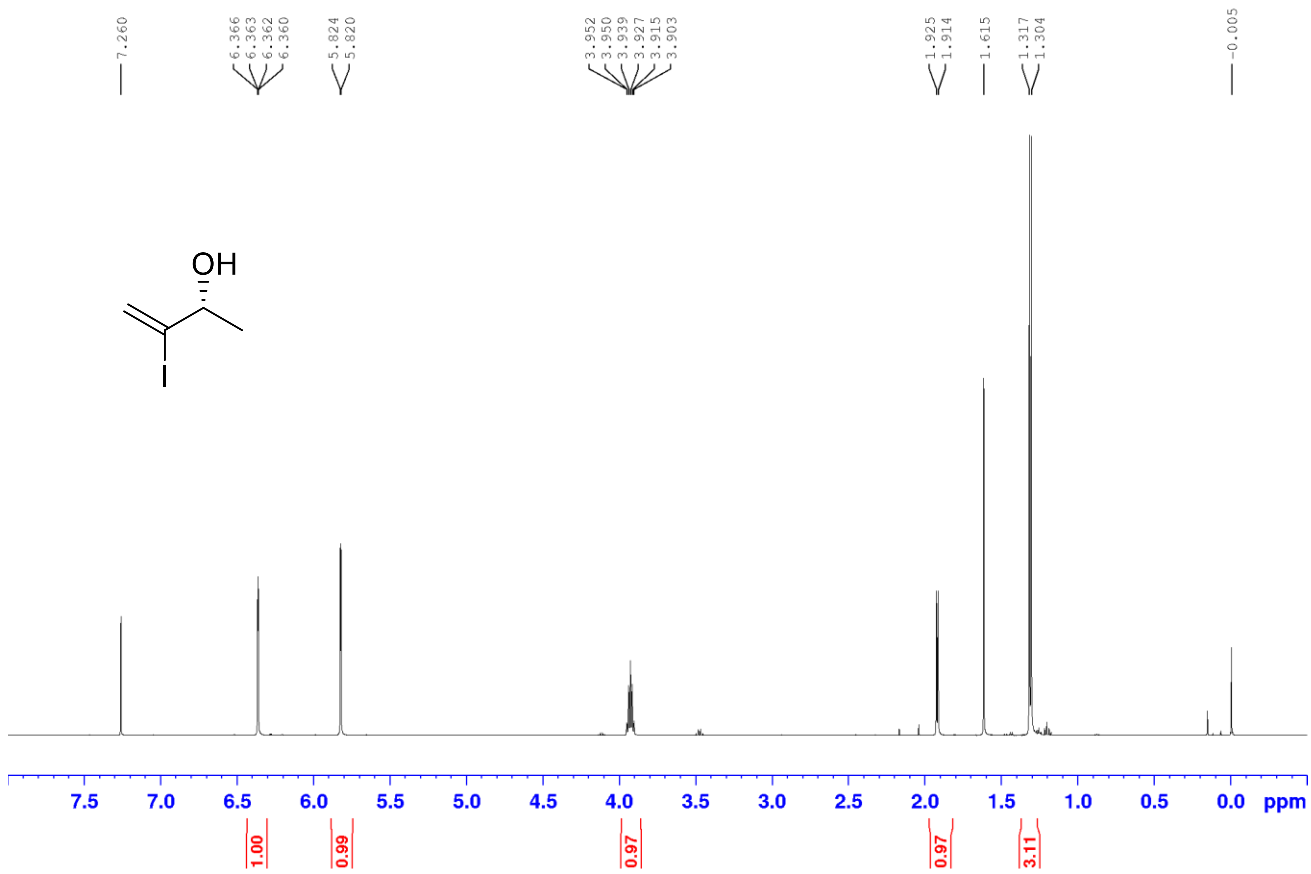

27, $CDCl_3$, 126 MHz

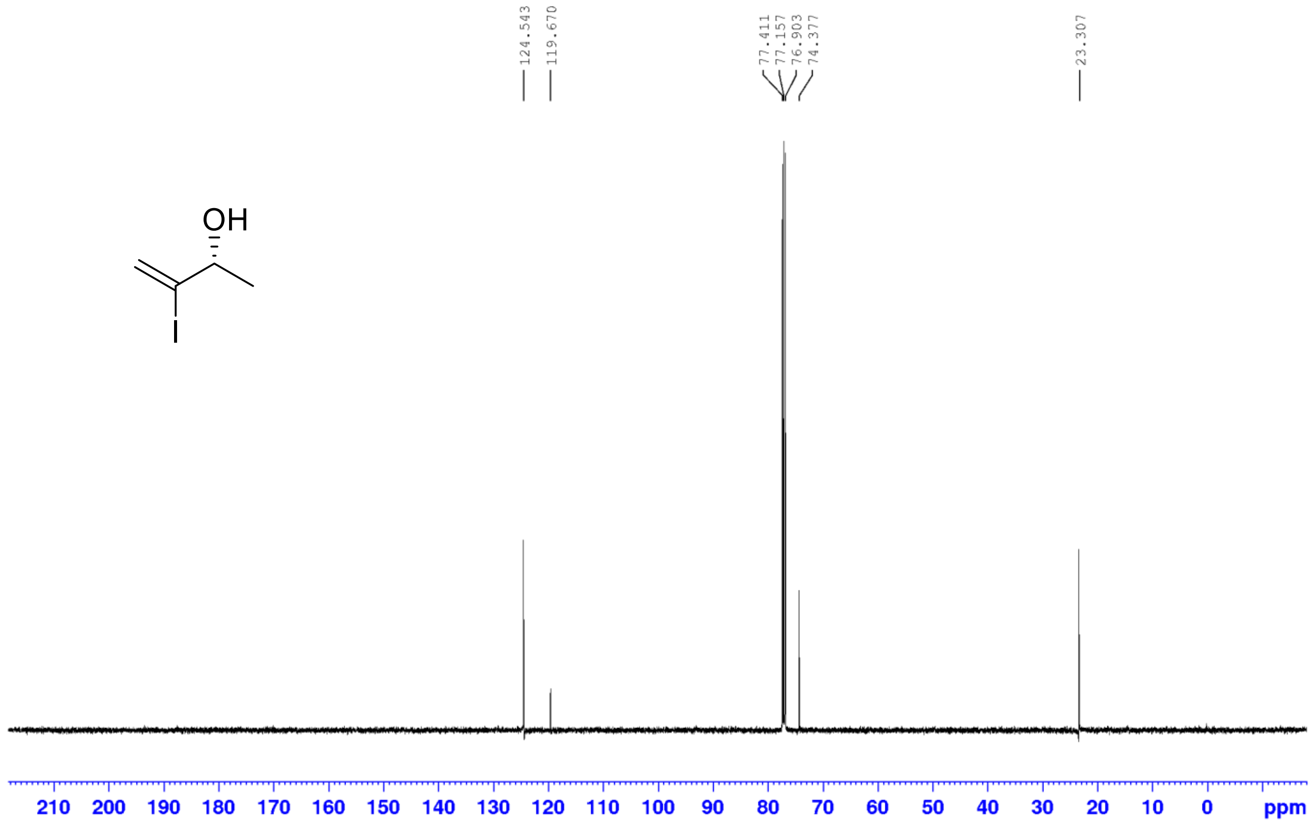

28, $CDCl_3$, 500 MHz

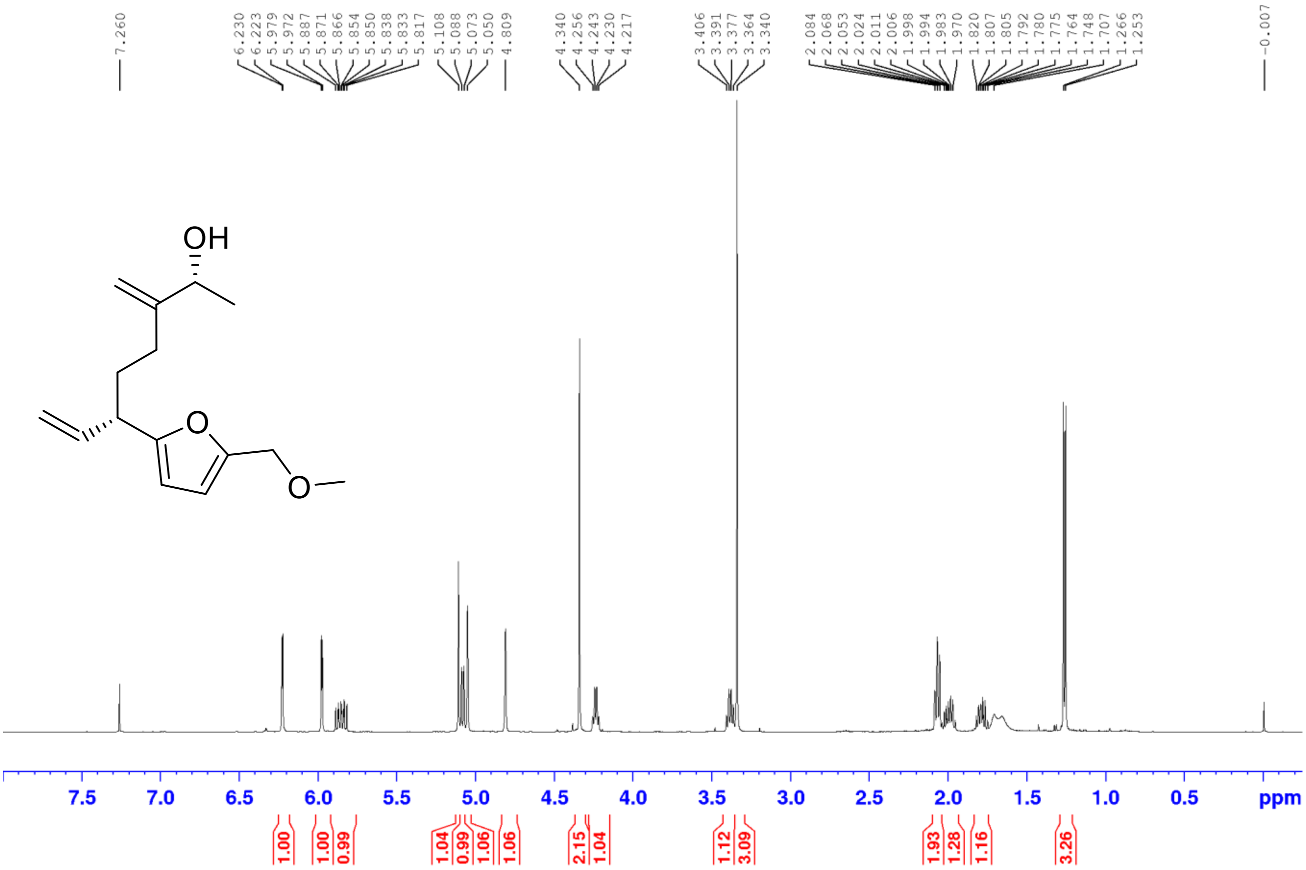

28, $CDCl_3$, 126 MHz

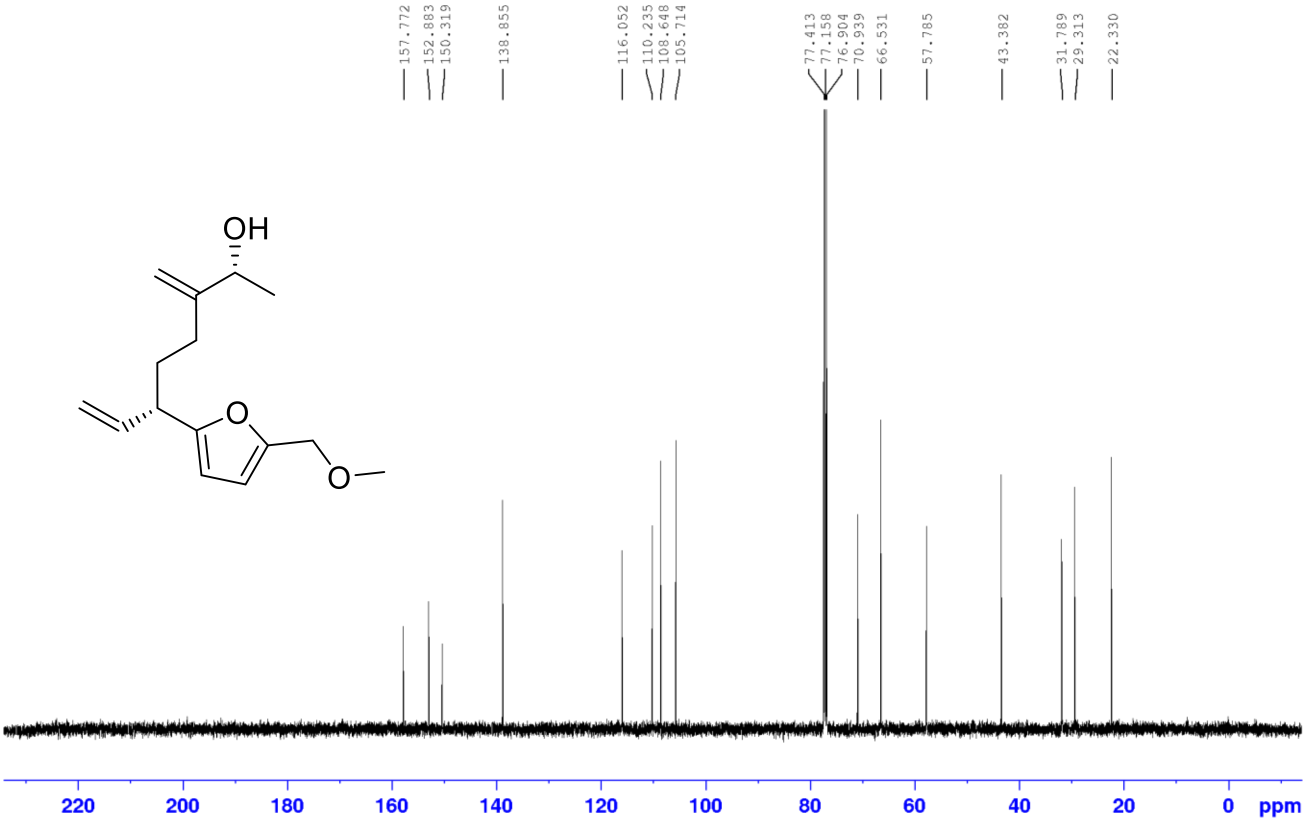

S25, $CDCl_3$, 400 MHz

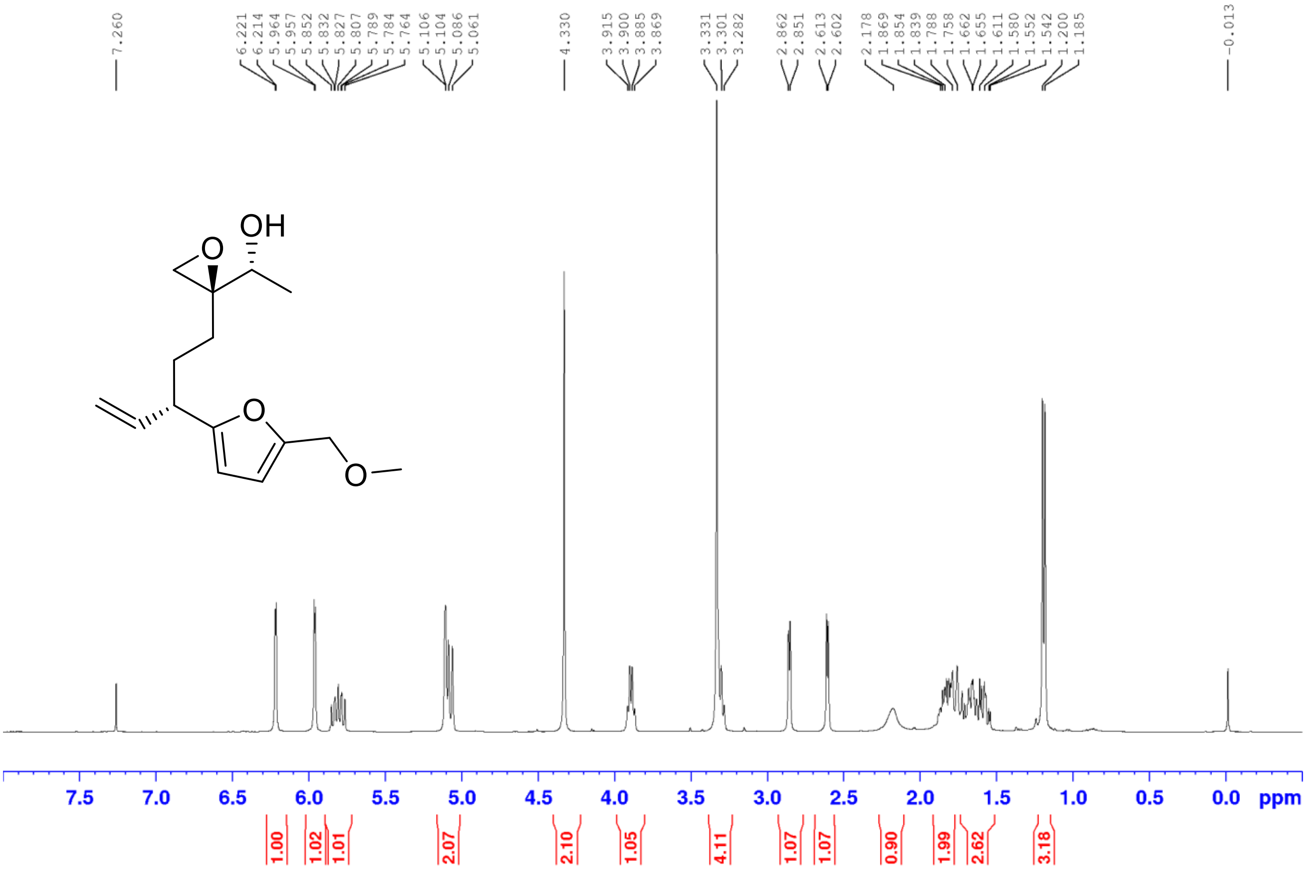

S25, $CDCl_3$, 100 MHz

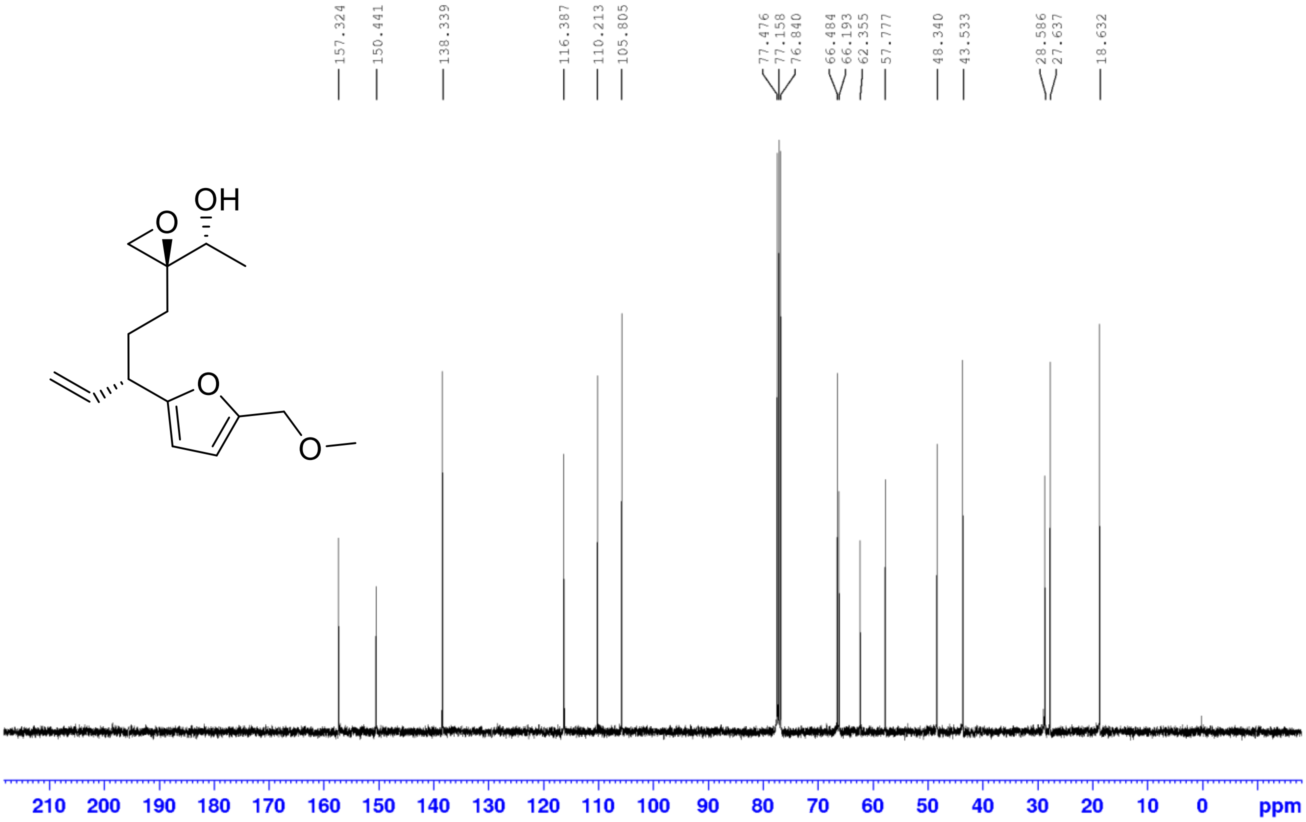

S26, $CDCl_3$, 400 MHz

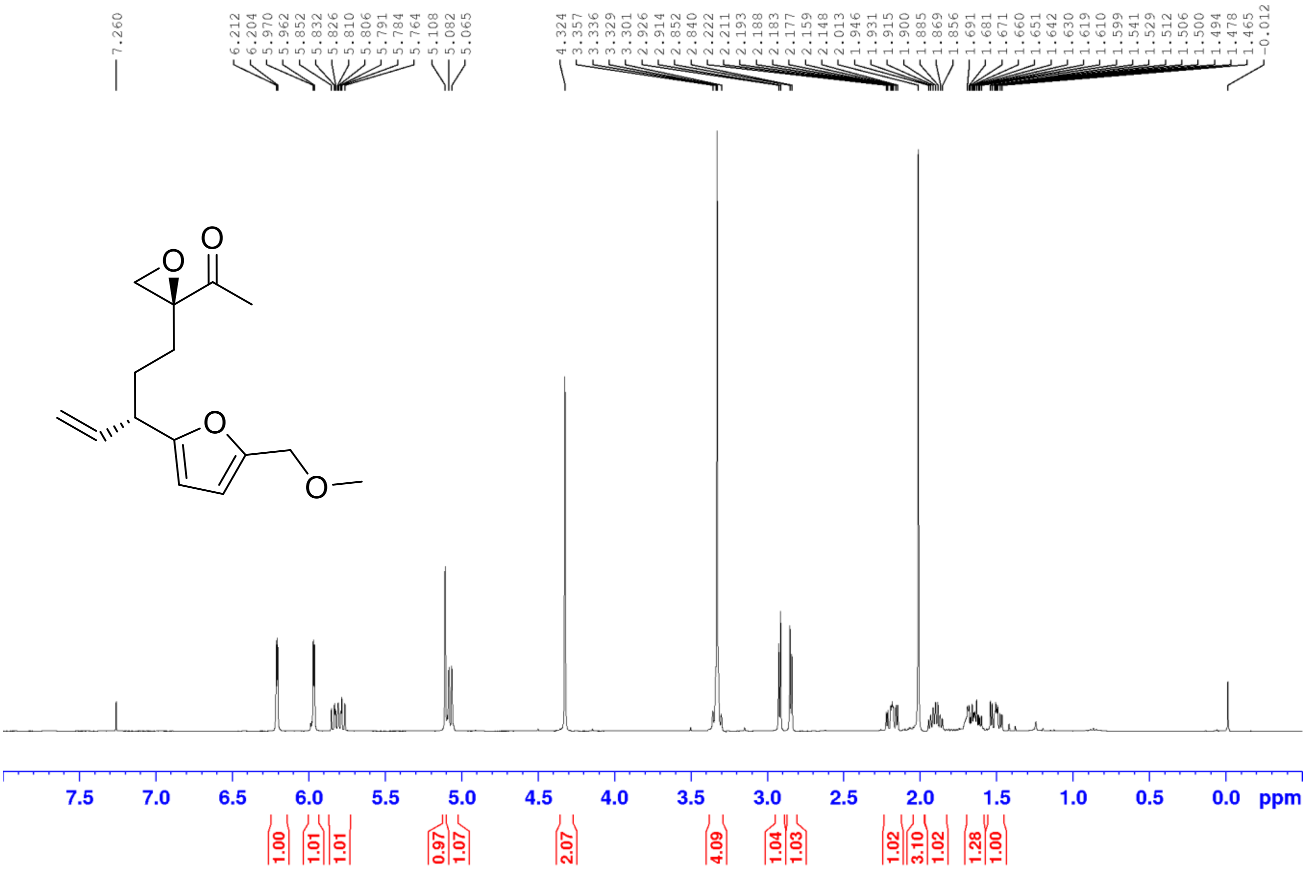

S26, $CDCl_3$, 100 MHz

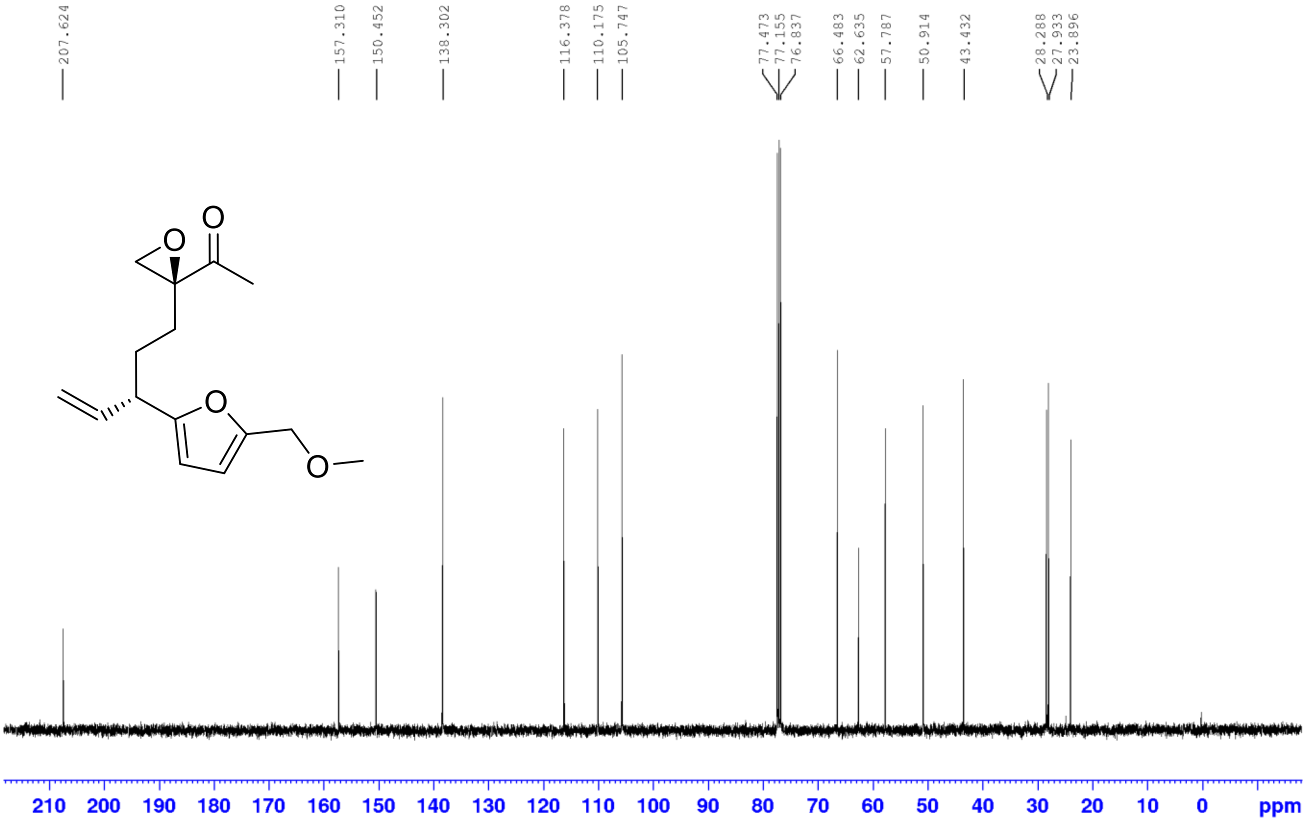

29, $CDCl_3$, 400 MHz

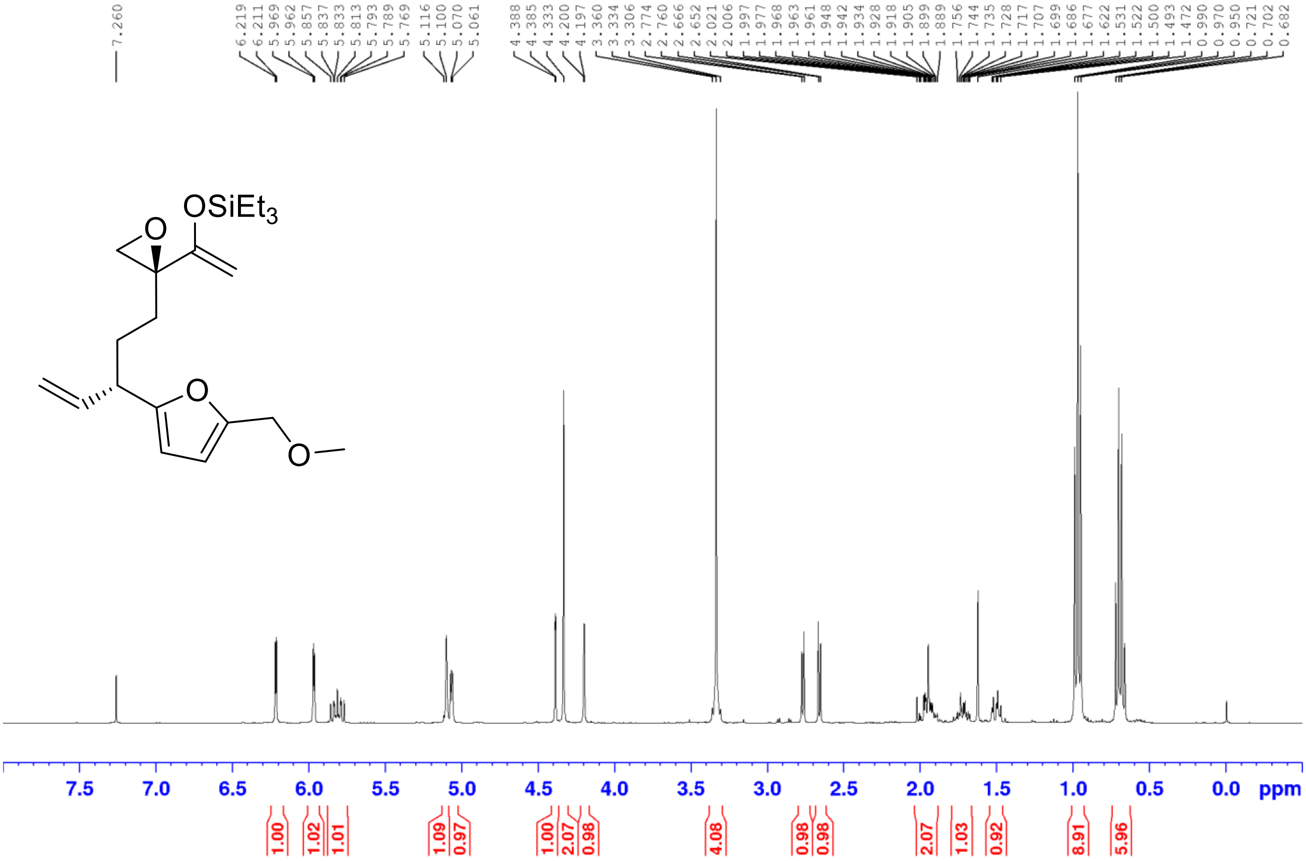

29, $CDCl_3$, 100 MHz

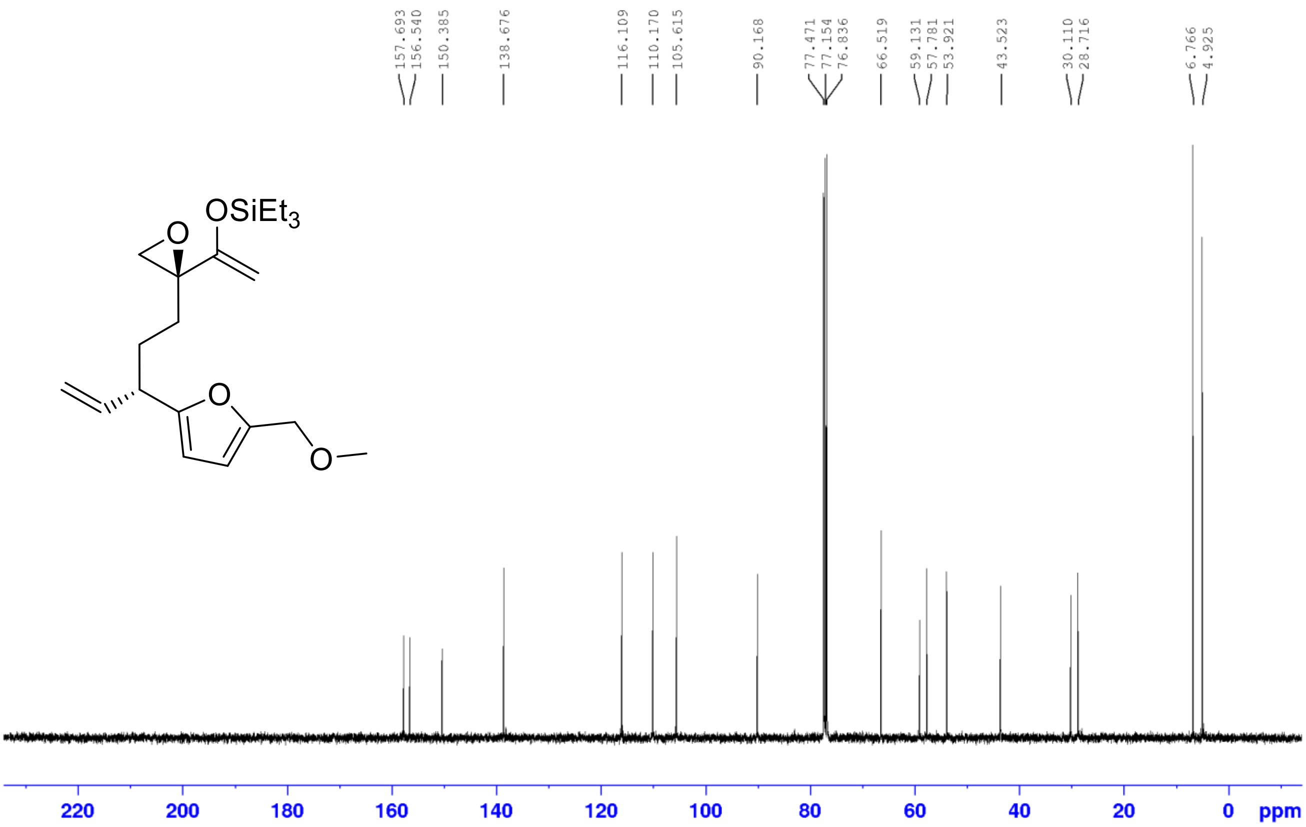

30, $CDCl_3$, 500 MHz

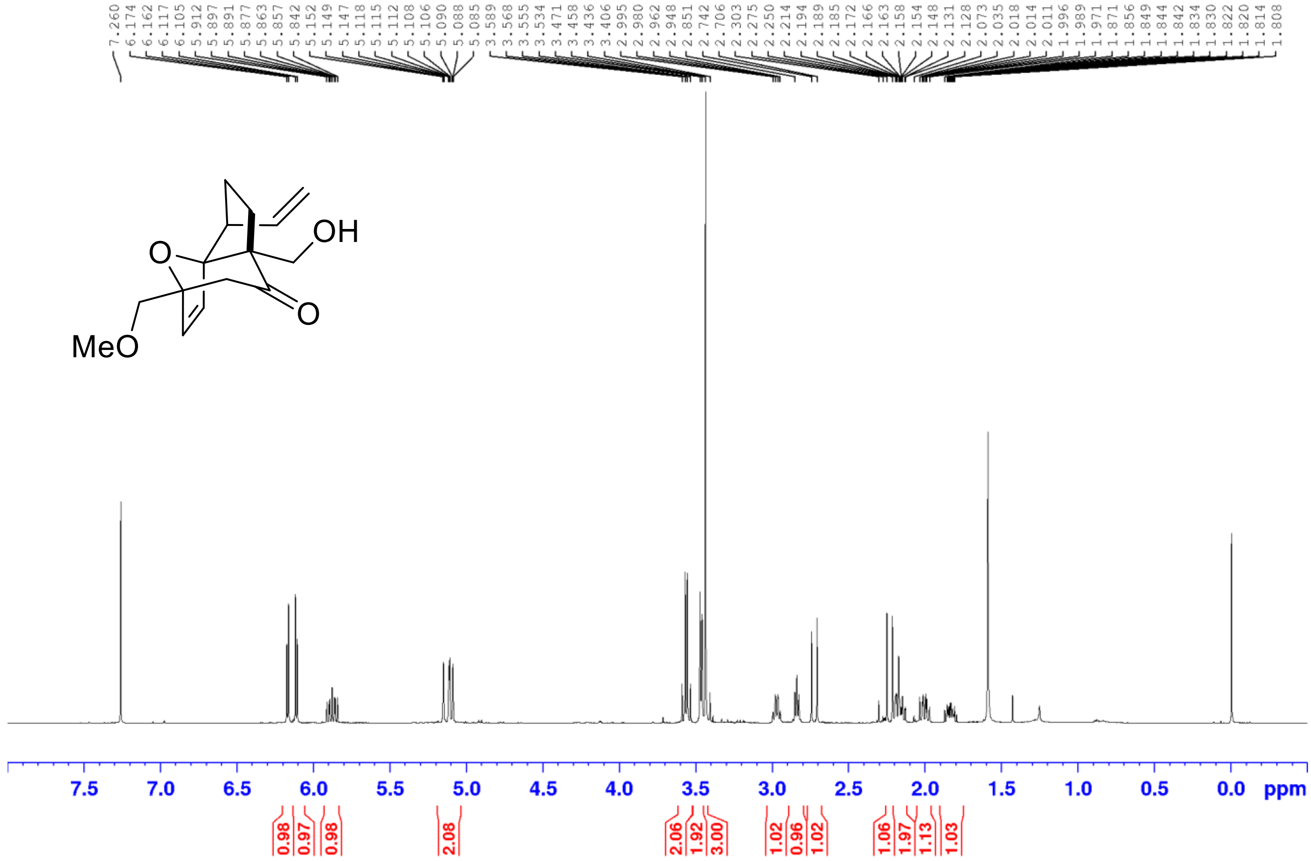

30, $CDCl_3$, 126 MHz

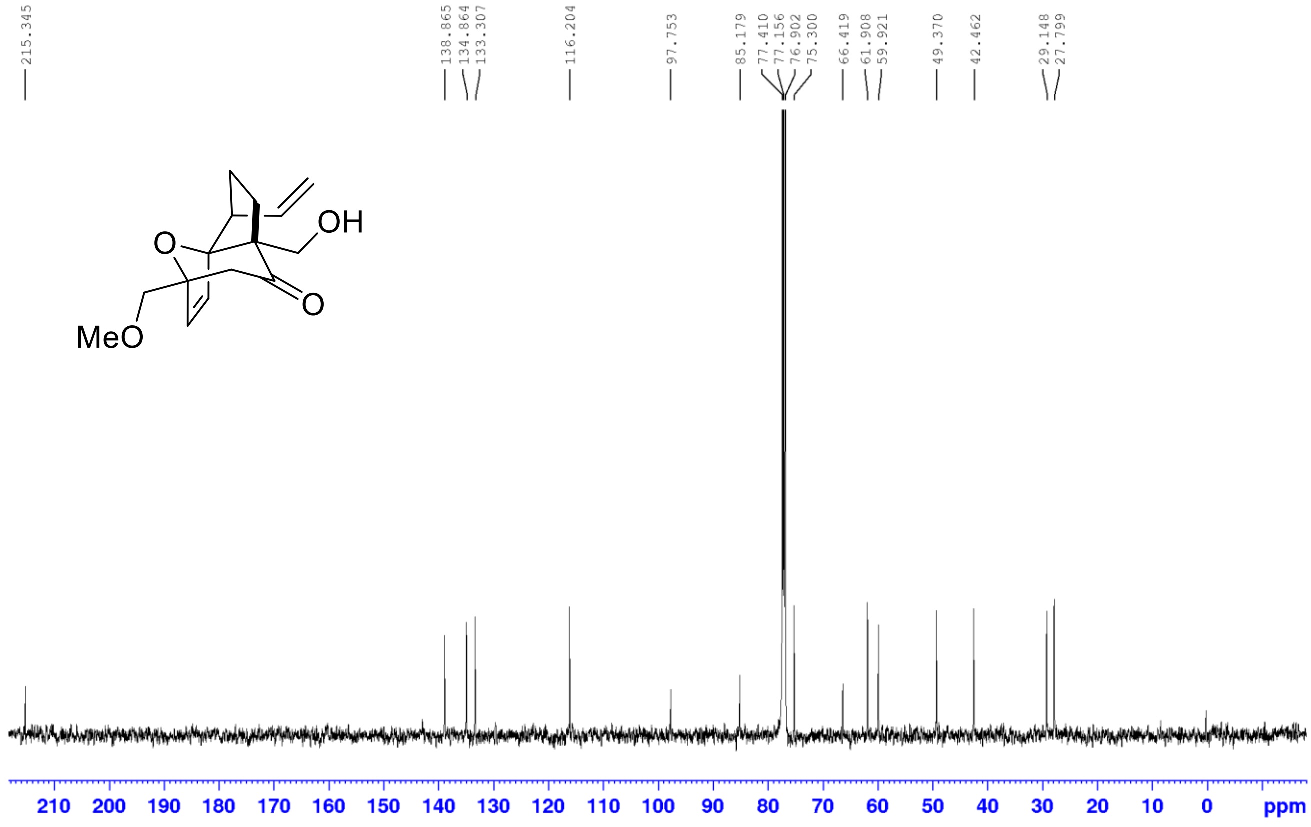

31, $CDCl_3$, 500 MHz

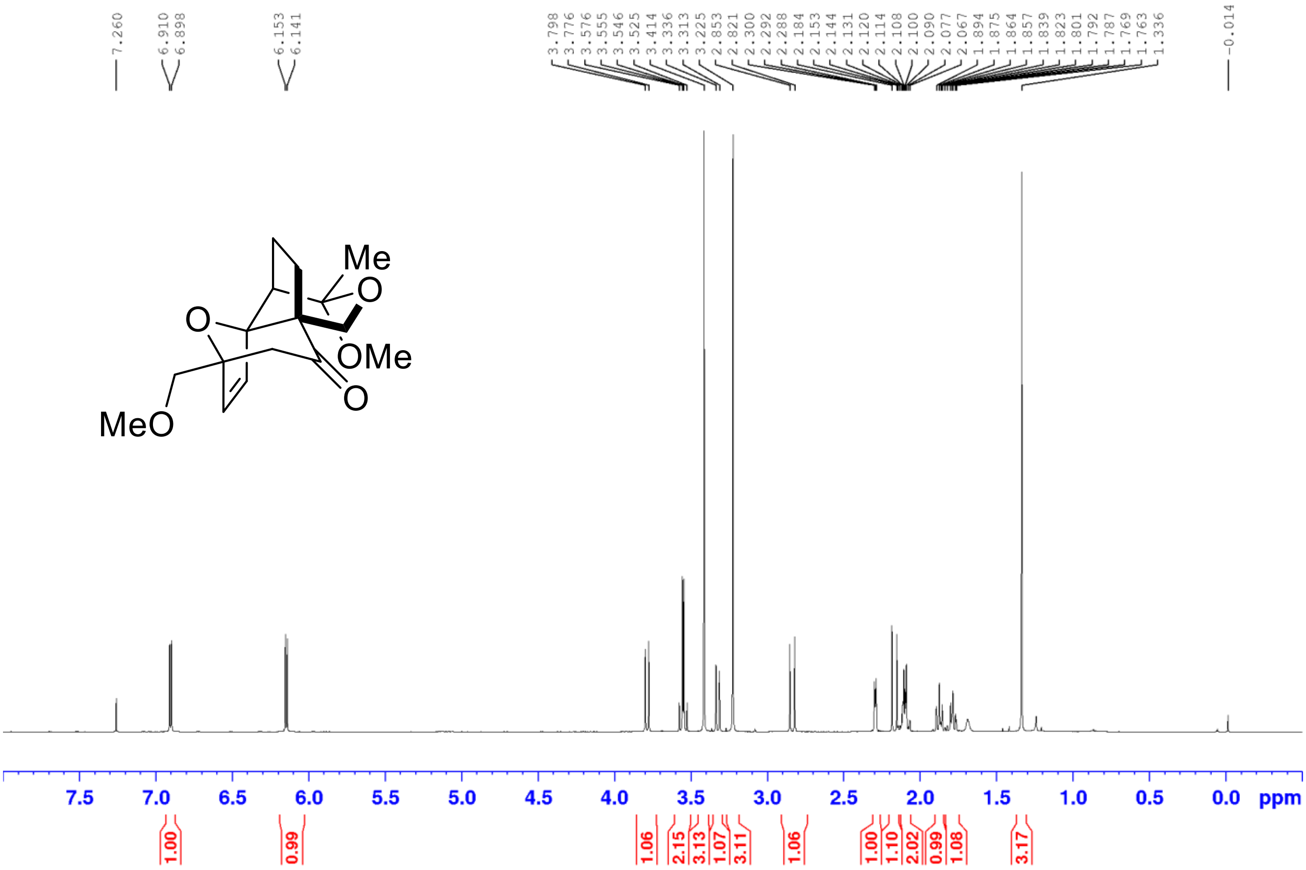

31, $CDCl_3$, 126 MHz

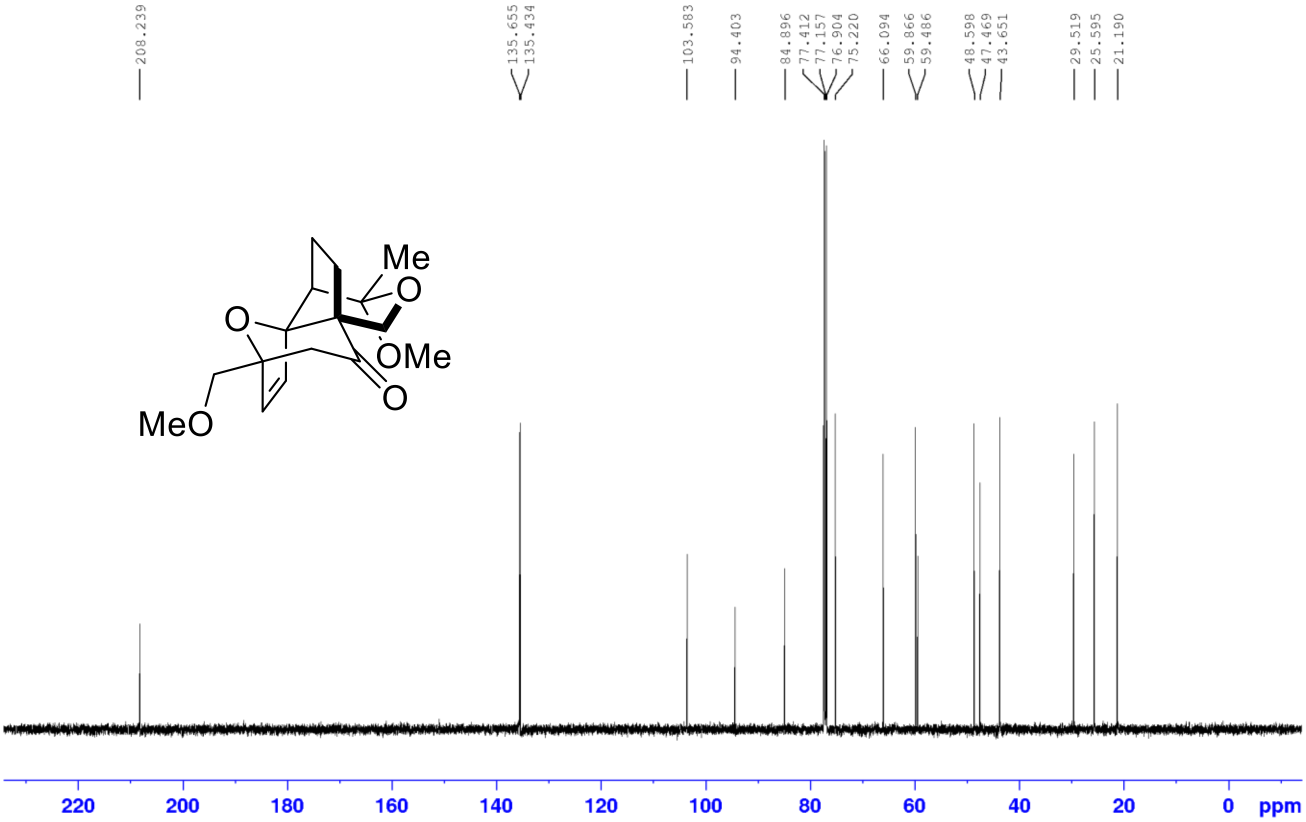

32, $CDCl_3$, 500 MHz

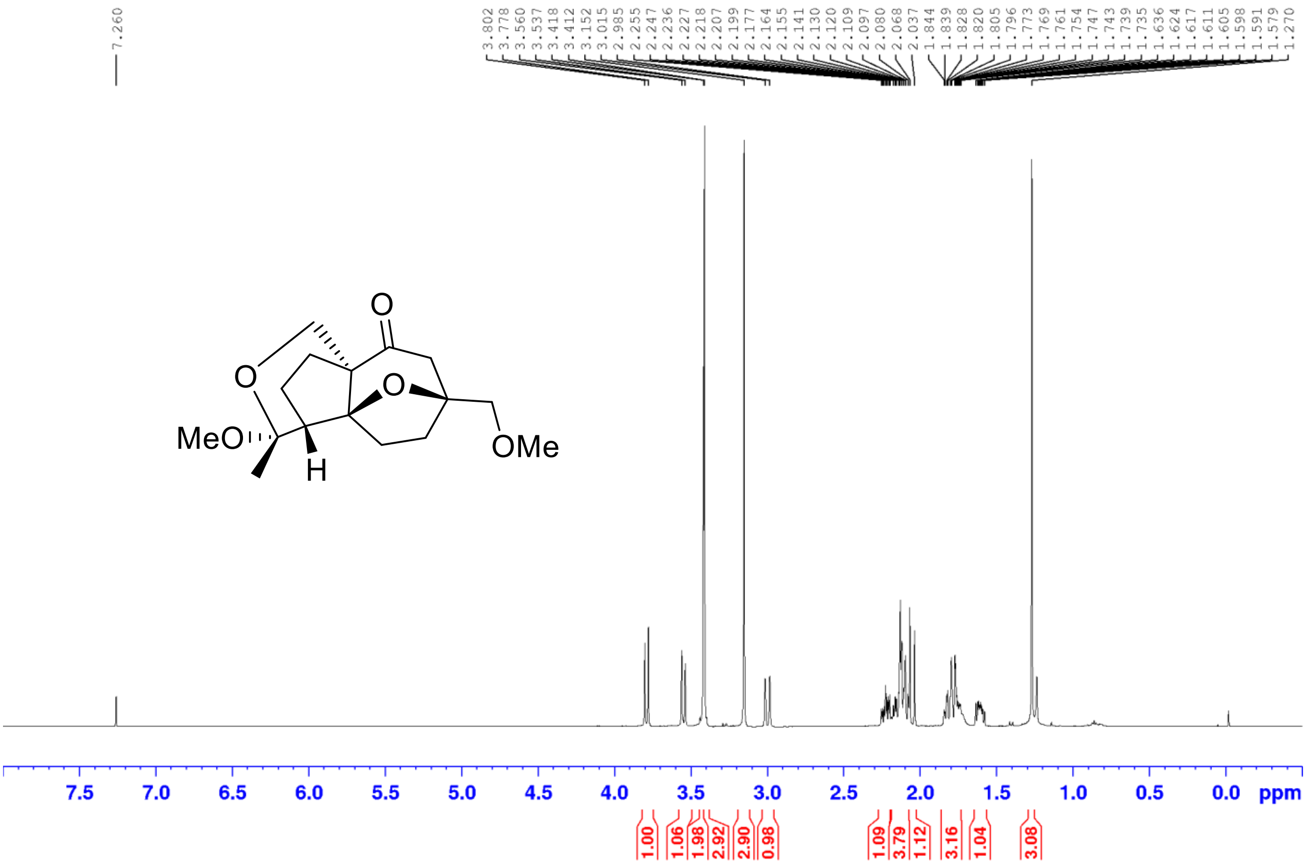

32, $CDCl_3$, 126 MHz

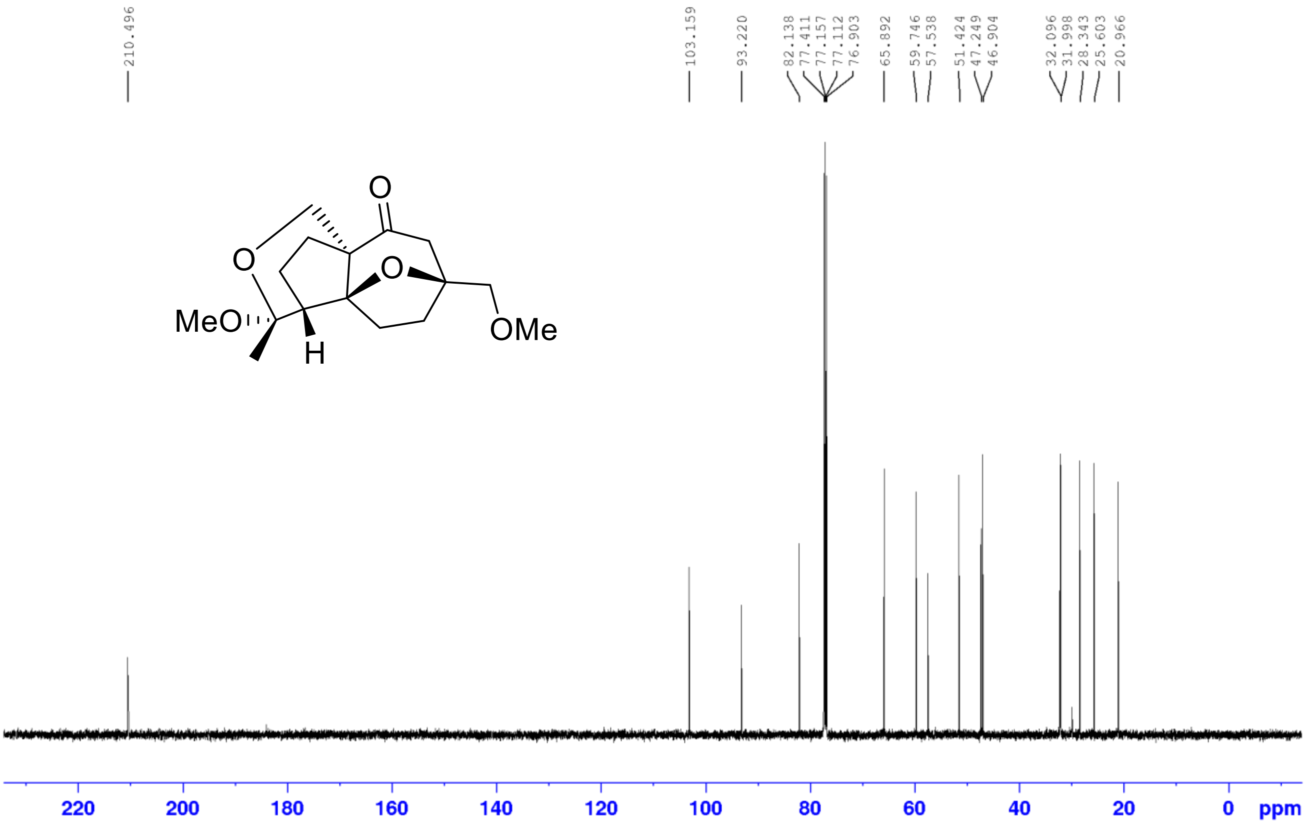

33, $CDCl_3$, 400 MHz

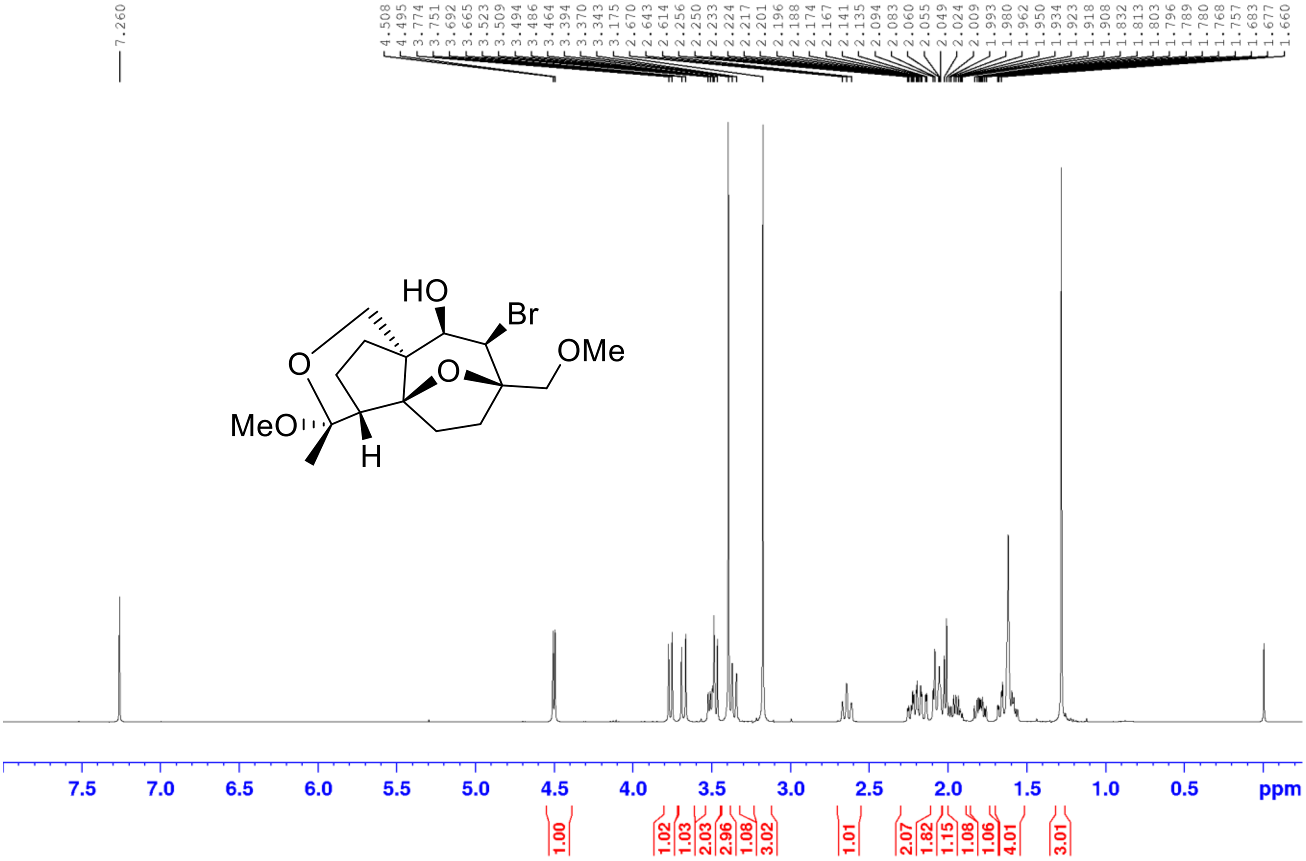

33, $CDCl_3$, 100 MHz

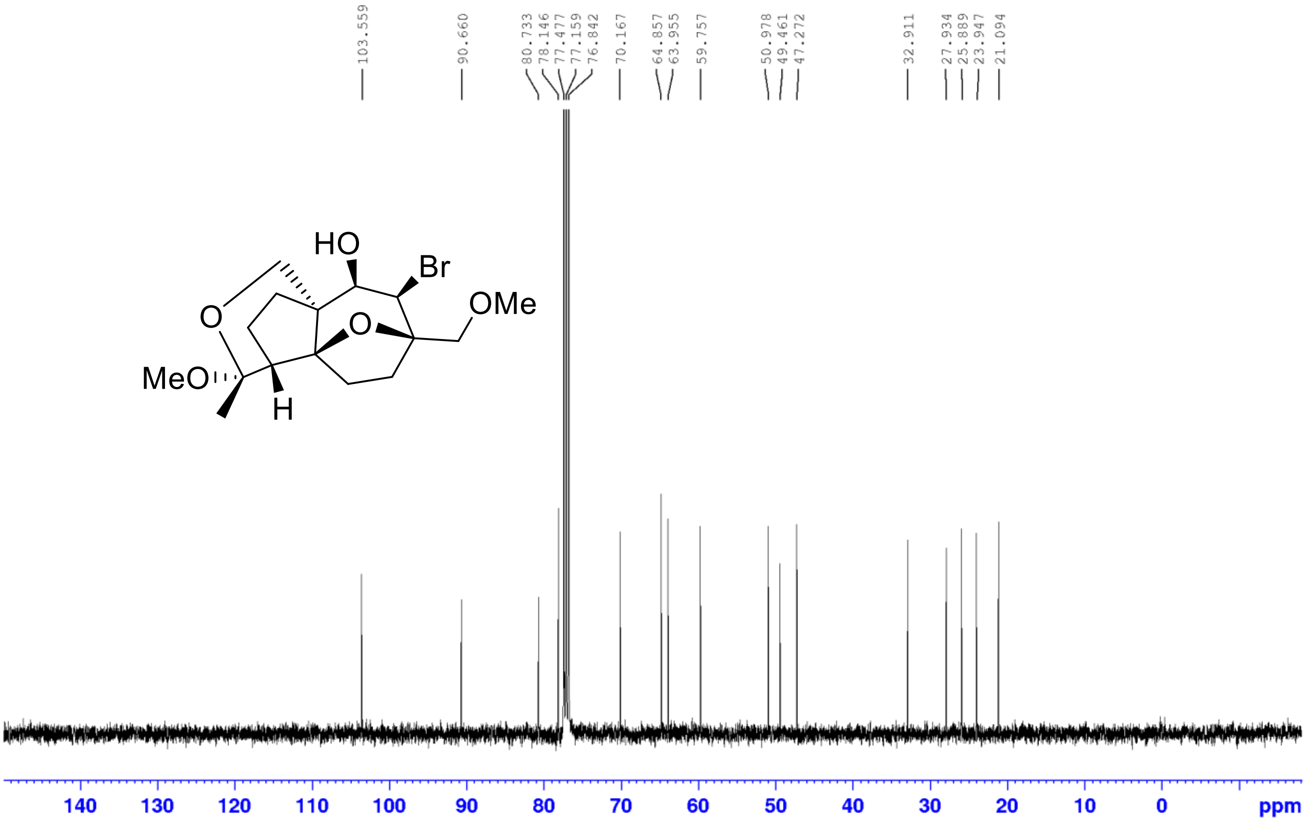

34, $CDCl_3$, 500 MHz

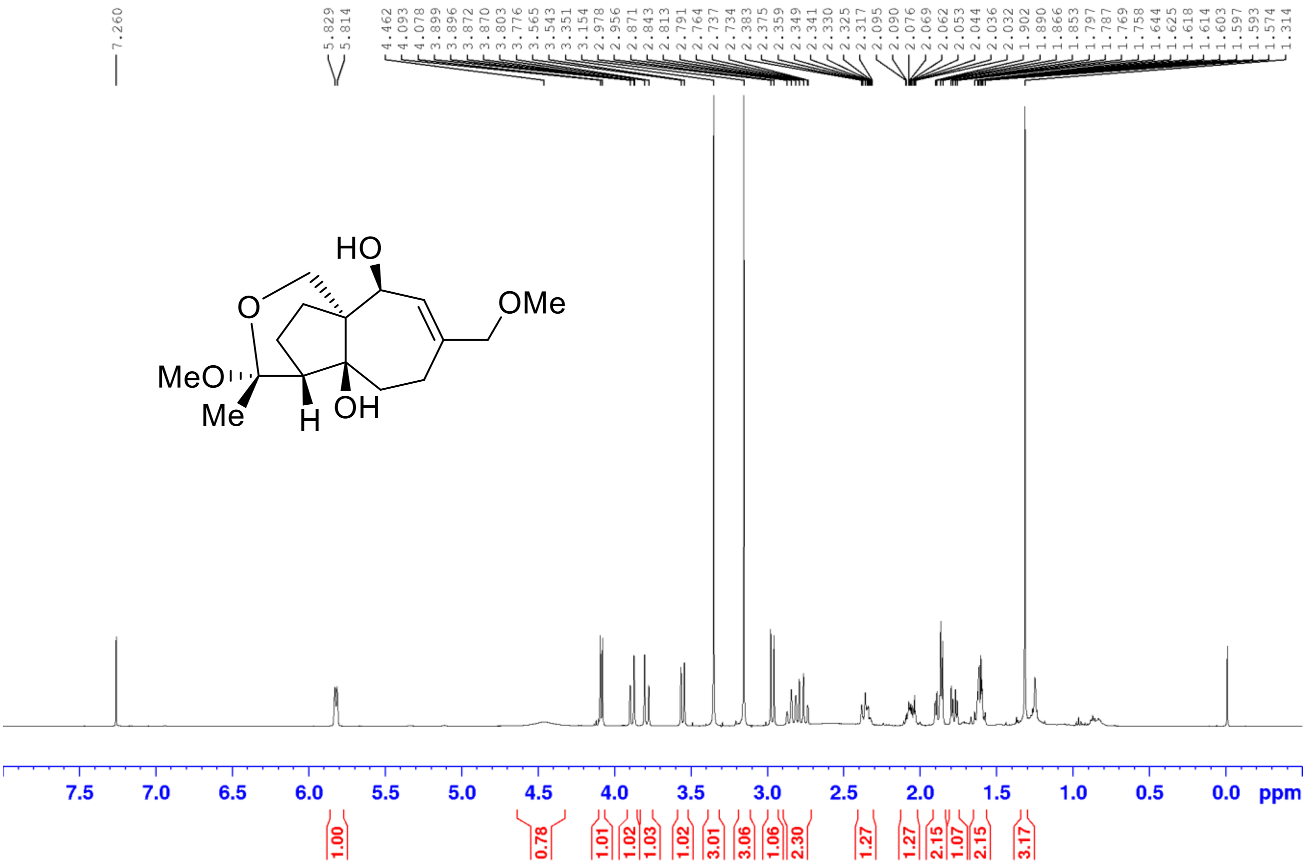

34, $CDCl_3$, 126 MHz

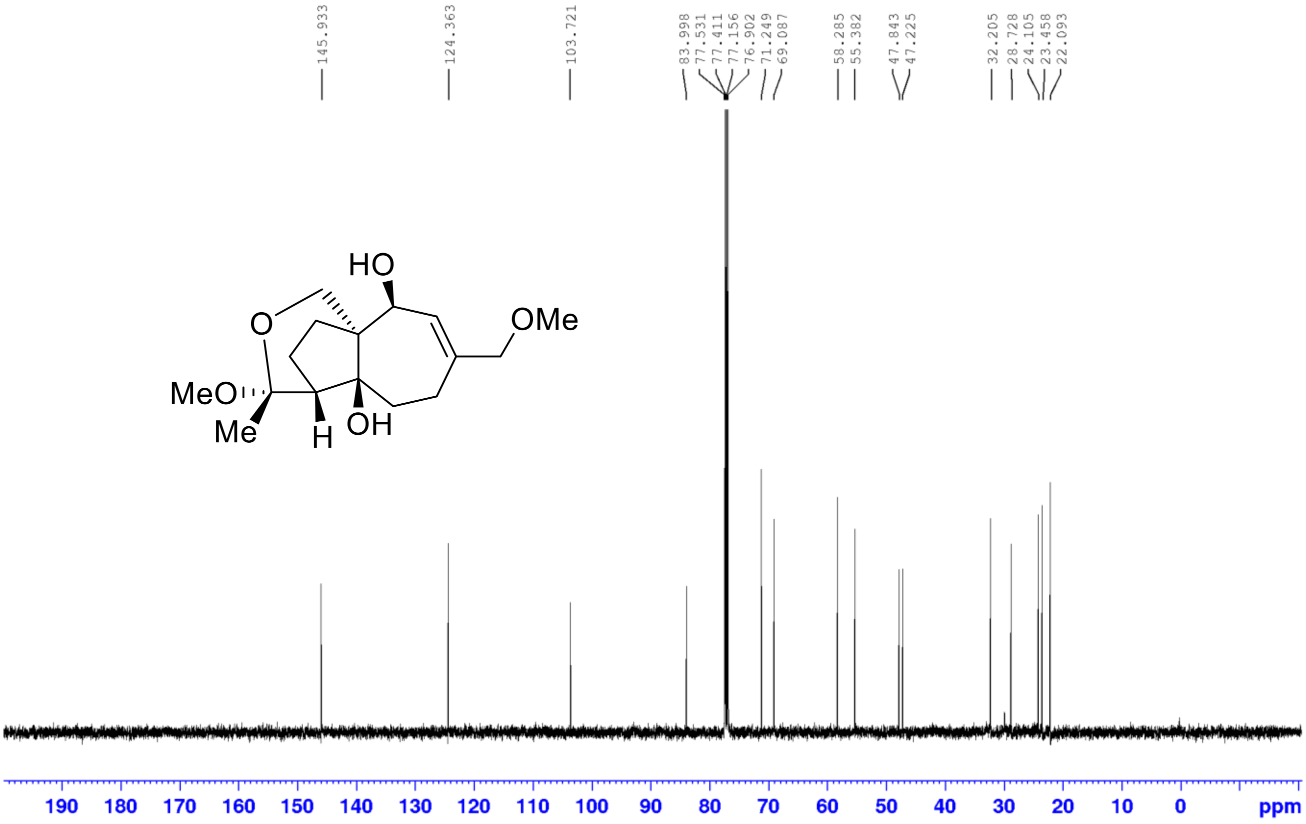

35, $CDCl_3$, 500 MHz

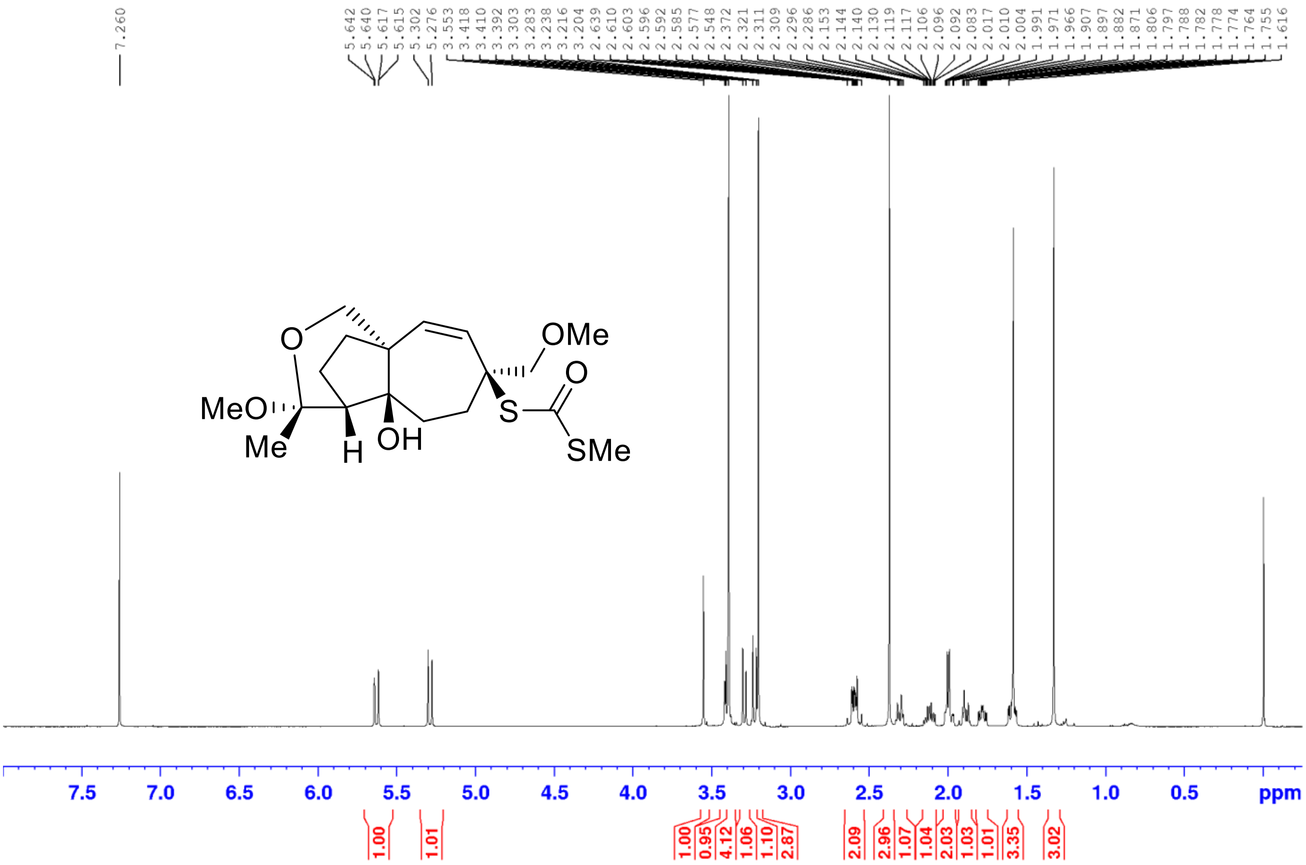

35, $CDCl_3$, 126 MHz

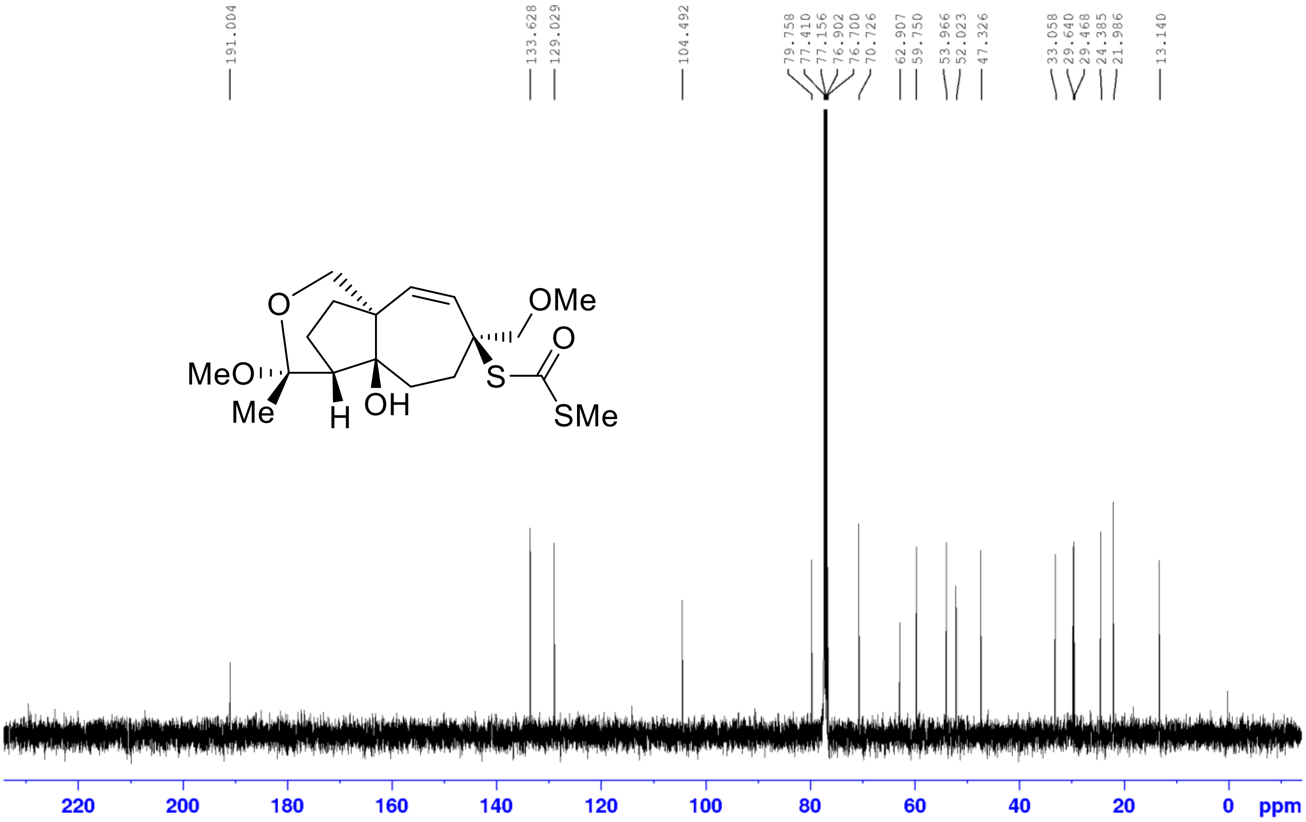

37, $CDCl_3$, 500 MHz

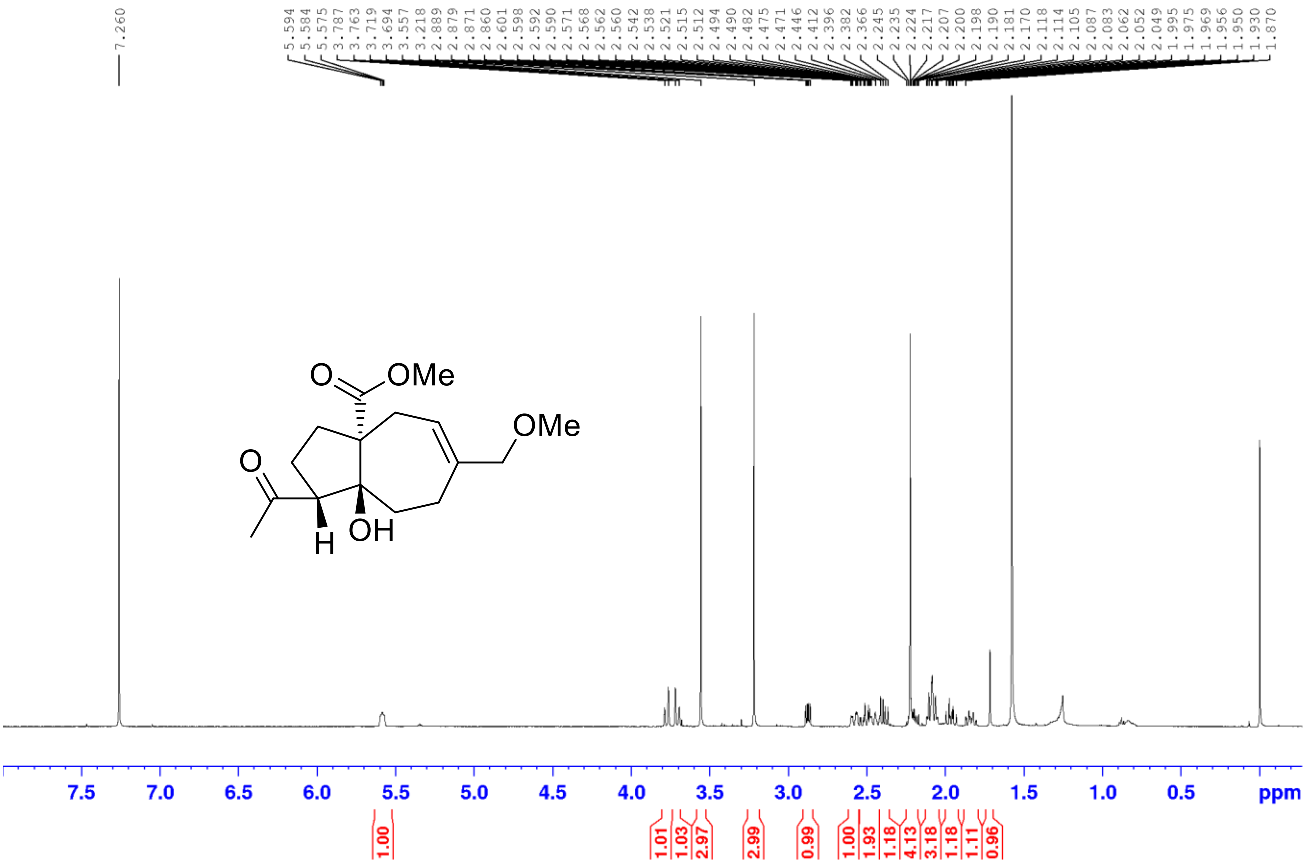

37, $CDCl_3$, 126 MHz

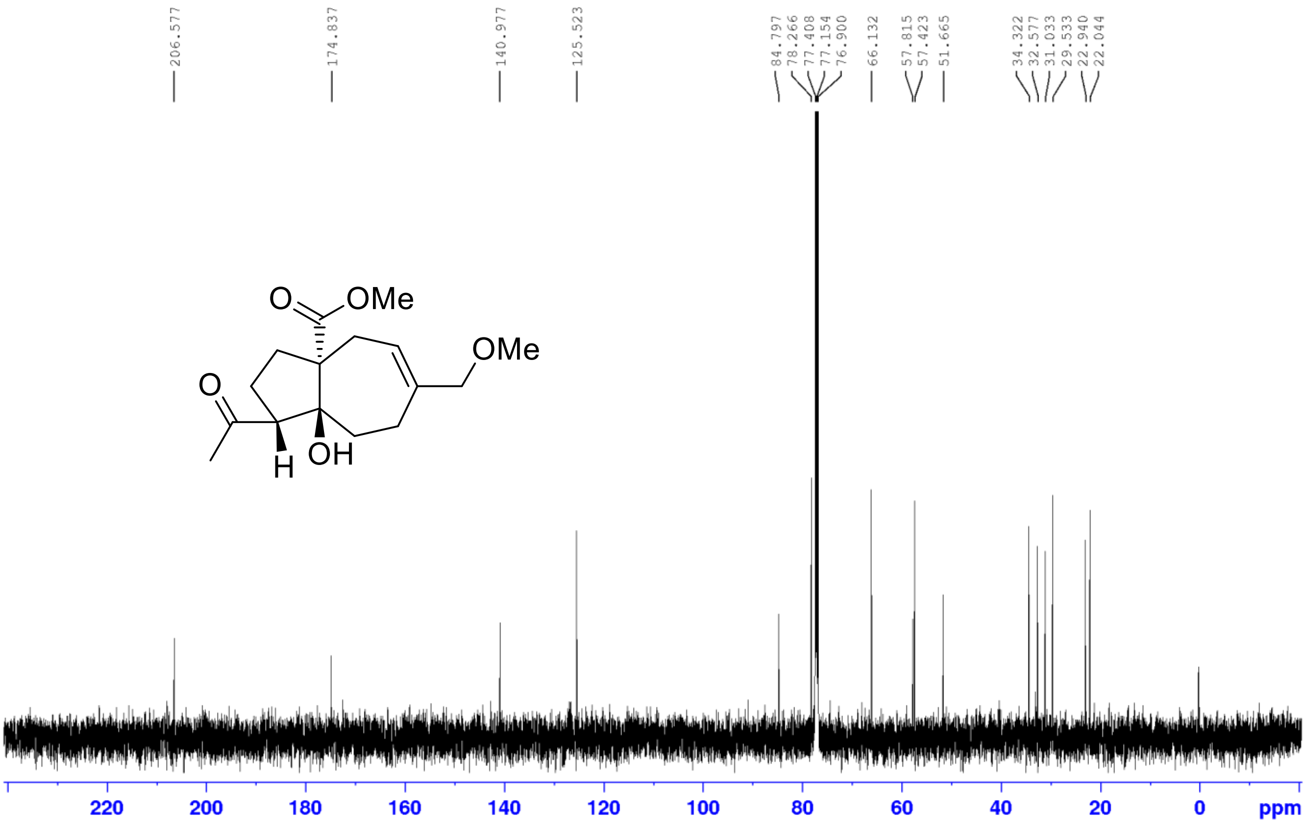

6, $CDCl_3$, 600 MHz

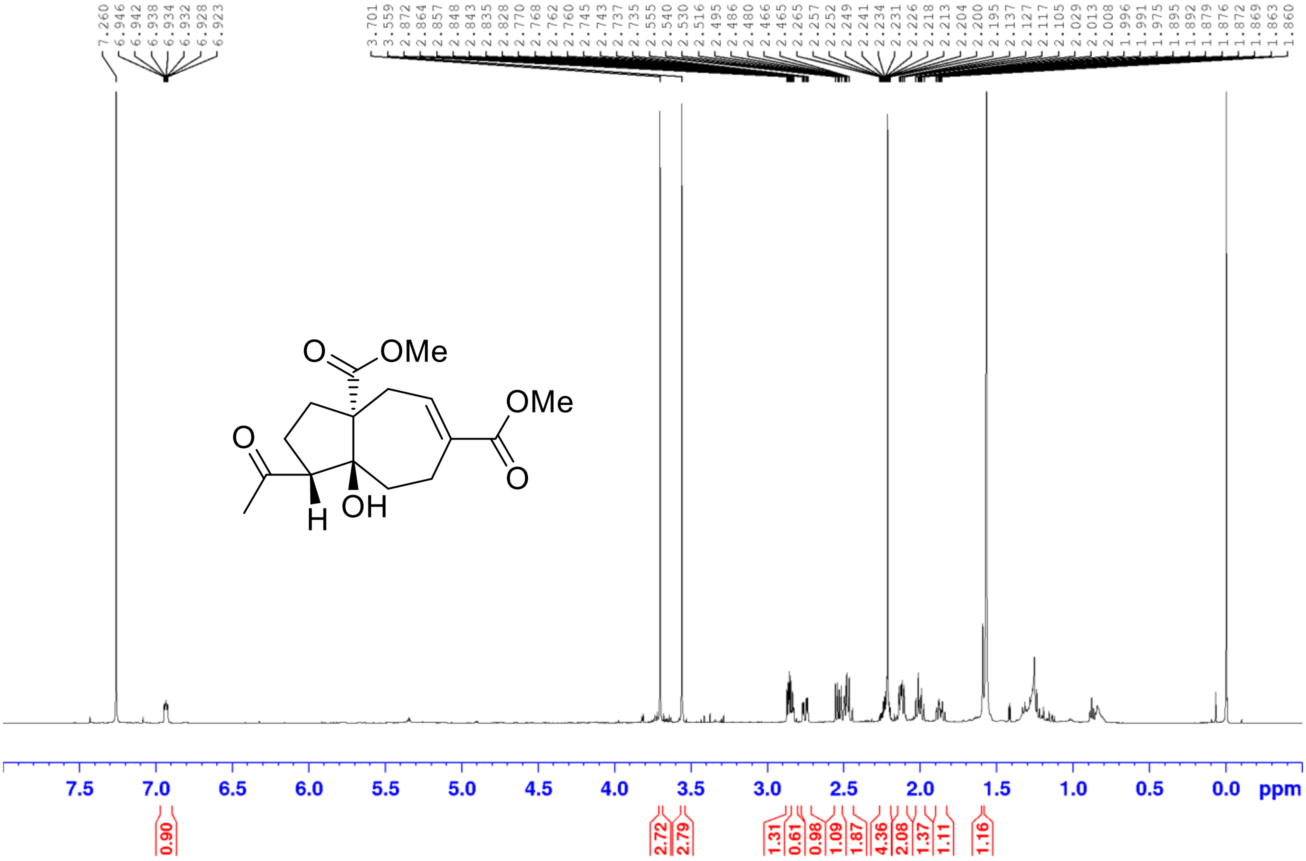

6, $CDCl_3$, 151 MHz

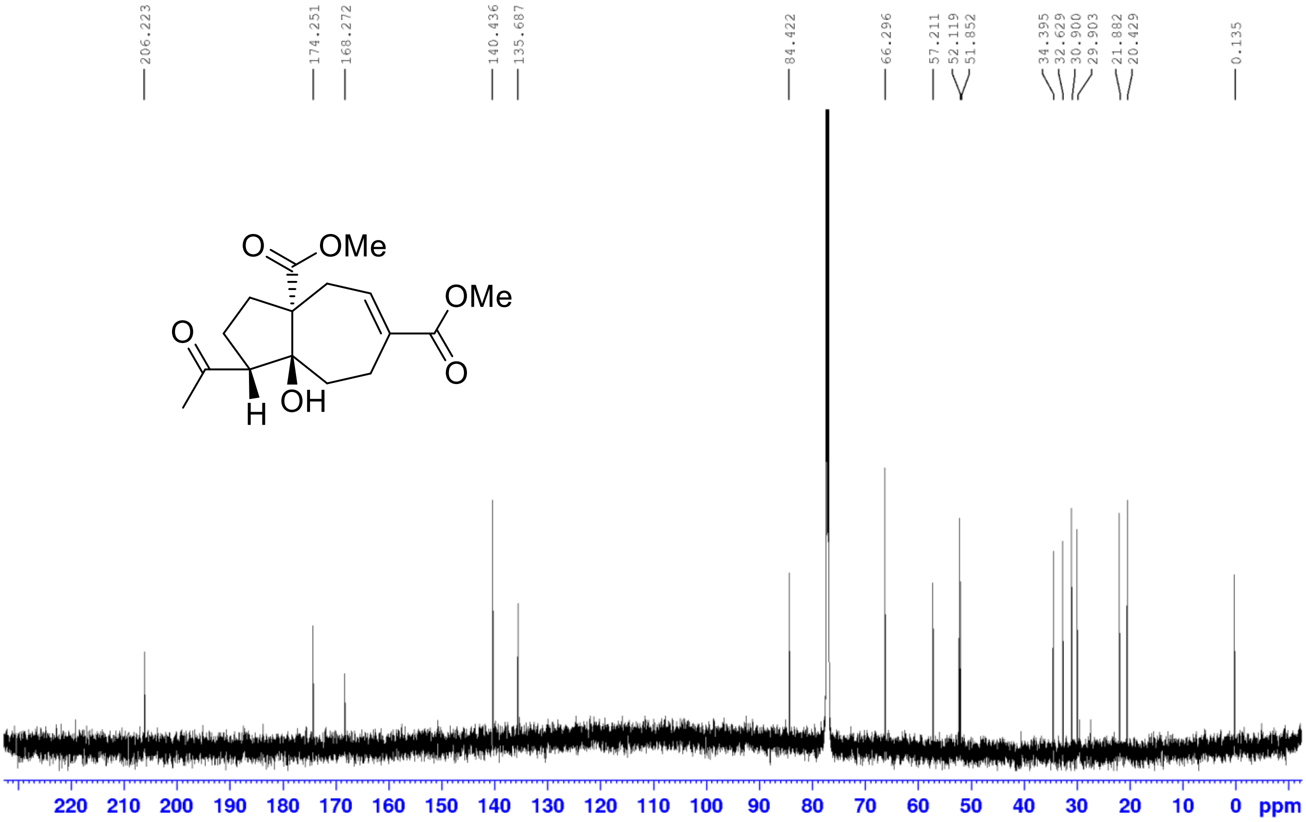

Supplement: Supplementary file 1 — Supporting Information [file ANIE-64-e202509650-s001.pdf]
